# Streamlined synthesis of functionalized dibenzo[*a*,*e*]pentalenes through potassium-mediated cyclization and late-stage thianthrenation

**DOI:** 10.1039/d6ra03886g

**Published:** 2026-06-08

**Authors:** Marcell M. Bogner, Bence Sóvári, Péter P. Kalapos, Péter J. Mayer, Márton Hegedűs, Gábor Turczel, Anna J. Kiss-Szemán, Veronika Harmat, Attila Kunfi, Gábor London

**Affiliations:** a Institute of Organic Chemistry, HUN-REN Research Centre for Natural Sciences 1117 Budapest Hungary london.gabor@ttk.hu; b Centre for Structural Science, HUN-REN Research Centre for Natural Sciences 1117 Budapest Hungary; c Laboratory of Structural Chemistry and Biology, Institute of Chemistry, Eötvös Loránd University Pázmány Péter sétány 1/A Budapest 1117 Hungary; d HUN-REN ELTE Protein Modelling Research Group Pázmány Péter sétány 1/A Budapest 1117 Hungary

## Abstract

Herein we report a simple and scalable synthetic platform for the dibenzo[*a*,*e*]pentalene (DBP) framework. *Via* potassium-mediated cyclization of TIPS-protected phenylacetylene we were able to produce multi-decagram-scale (>25 g) quantities of a 5,10-TIPS protected DBP key intermediate, which enabled further complexity-enhancing transformations. Halodesilylations on the pentalene core and aryl thianthrenation on the annulated benzene rings enabled highly modular synthetic pathways towards complex DBP-based structures.

## Abstract 2

Herein we report a simple and scalable synthetic platform for the dibenzo[*a*,*e*]pentalene (DBP) framework.

## Graphical abstract

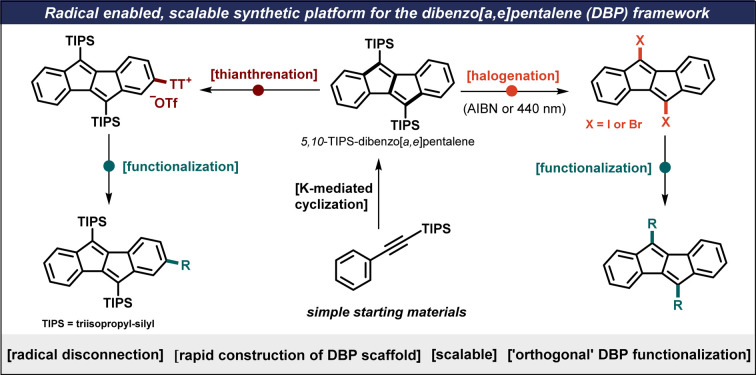

## Introduction

**Fig. 1:**
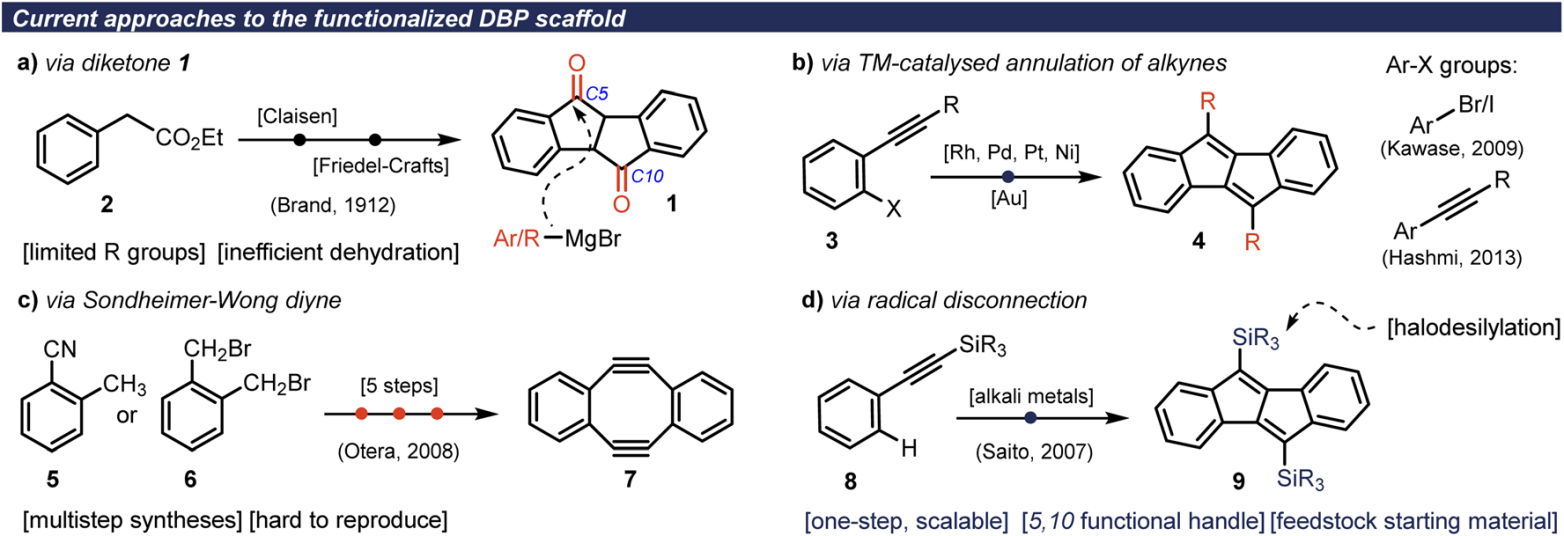
Context of the research. (A)–(D) Previous strategies to access the dibenzo[*a*,*e*]pentalene scaffold with various substitution patterns.

Dibenzo[*a*,*e*]pentalenes (DBPs) have received significant attention in recent years, owing to their potential in organic material science^1–3^ including single molecule electronics,^4–6^ covalent organic frameworks,^7,8^ nanohoops^9–14^ and organic optoelectronic devices.^15–18^ As a result, several distinct synthetic approaches have been developed to access the DBP framework with various substitution patterns.^19,20^ Key to these approaches is the assembly of the antiaromatic pentalene core.^1,21^ This has been accomplished *via* a number of strategies, all featuring their own benefits and drawbacks. The four most prominent pathways are summarized in Fig. 1.

Diketone 1 (Fig. 1a) was first described by Brand^22^ as a key intermediate en route to DBPs. This structure can be prepared in 2 steps (from 2) and a subsequent Grignard addition/dehydration sequence yields the 5,10-disubstituted DBP framework (4). Inherently, aryl (or R) groups that can be introduced are limited, while the dehydration step could compromise the yields.^23^ Development of this route has been put forward by the group of Esser recently.^14,23^ Alternatively, *ortho*-substituted aryl-acetylenes (3) can be converted to pentalenes *via* transition metal catalyzed homo-annulation^24–26^ and crossover-annulation^27,28^ processes (Fig. 1b). The dimerization of *ortho*-halogenated aryl-acetylenes was explored initially by Kawase and co-workers,^24^ and Levi and Tilley.^25^ Gold-catalyzed cyclization of *ortho*-diacetylenes, pioneered by Hashmi and co-workers, provides DBPs with non-substituted or aryl-substituted pentalene cores.^29,30^ Similarly, the strongly Lewis acidic B(C_6_F_5_)_3_ mediated transformation of *ortho*-diacetylenes reported by the groups of Erker and Yamaguchi furnishes DBPs with aryl-substituted pentalene subunits.^31^ Very recently, Ganesh and co-workers reported an approach to DBPs through a Pd-catalyzed twofold Suzuki coupling of *gem*-dibromo olefins and benzene-1,2-bisboronates.^32^ While these methodologies can be used to access DBPs, the 5,10-substitution sites are essentially locked after the assembly of the framework or lacking suitable functional groups for further derivatization. To allow for flexible 5,10-substitution, the preparation of dihalo-DBPs would be the most straightforward approach. Nevertheless, access to these highly valuable intermediates remains limited and labor-intensive. Developments by Otera and coworkers (Fig. 1c) leverage Sondheimer–Wong diyne 7, which requires a challenging multistep synthesis.^33,34^ Notably, diyne 7 can be converted to dihalo-DBPs in a single step using halogens (Br_2_ or I_2_) or interhalogens (IBr). Finally, based on the work of Saito and co-workers, simple aryl-acetylenes (8) can also be converted to dibenzopentalenes (Fig. 1d) in the presence of alkali metals.^35–38^ This reaction yields 5,10-trialkylsilyl-protected DBPs (9), which can be subjected to halodesilylative conditions to yield dihalo-DBPs.^38,39^ This simple, one-step route was selected to be the bedrock for our versatile DBP platform.

In this contribution, we aimed to provide a complete synthetic platform for the diverse functionalization of dibenzo[*a*,*e*]pentalenes leveraging efficient decagram scale framework assembly based on Saito's potassium-mediated DBP synthesis and late-stage aryl thianthrenation.

## Results and discussion

**Scheme 1:**
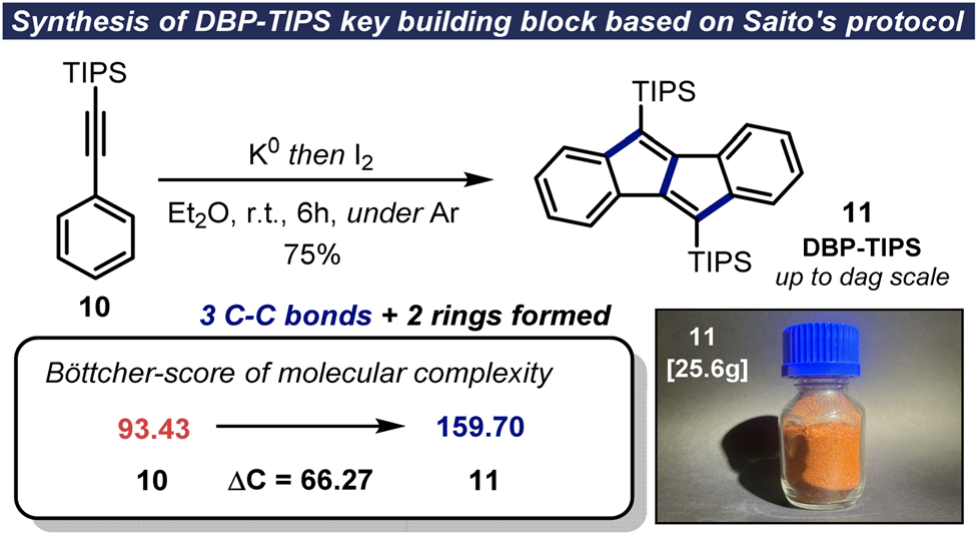
Decagram-scale synthesis of DBP-TIPS 11.

Major synthetic pathways for DBP reported to date lack the following key features: (a) high material throughput of key dihalo-DBP intermediates, and (b) diverse late-stage functionalization on the DBP framework. Through a key radical disconnection, 5,10-trialkylsilyl-dibenzopentalenes (9) can be simplified into the corresponding silyl-protected phenylacetylenes (8). These simple starting materials (of which some are commercially available) can easily be prepared on decagram-scale *via* Sonogashira coupling or silyl protection of phenylacetylenes (Section S3.2, SI). In a simple operation, reported by Saito and co-workers, these internal acetylenes can yield the DBP framework in the presence of alkali metals in aprotic media (Scheme 1).^35,37,38^ This special reductive cyclization – involving a formal Birch reduction step – (for mechanistic details see Section S3.2.3, SI) results in the formation of 3 bonds and 2 rings in a single step, highly increasing molecular complexity early in the synthetic pathway (illustrated by the Böttcher-score (C),^40,41^Scheme 1). Notably, an alternative reaction mechanism has been proposed, also involving alkali metal induced radical transformations.^35,37^ Reaction conditions were optimized^37^ and advanced to a preparative level by screening alkali metals, solvents and silyl protecting groups (see Section S3.2.1, SI), with metallic potassium and triisopropyl-silyl group found to be the most efficient combination for our further studies. As an advanced building block, 11 could be prepared on 25 gram scale (from 10) enabling the streamlined synthesis of several complex derivatives.

**Scheme 2:**
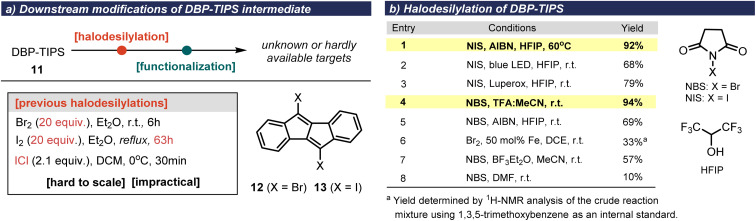
(a) Downstream modification on the pentalene core. (b) Halodesilylation conditions.

Although the bulky TIPS groups facilitated the high-yielding preparation of 11, subsequent halodesilylation proved to be a significant challenge due to the same steric feature. Previously reported conditions for these halogenations involved either vast excess of halogens^38^ (20 equiv. of Br_2_ or I_2_) combined with multiday reaction times or interhalogen compounds^39^ (typically ICl or IBr) (Scheme 2a). To ensure safe and practical gram-scale syntheses of dibromo (12) and diiodo (13) derivatives we searched for conditions leveraging more conventional *N*-halosuccinimide reagents (NXS) (Scheme 2b). Extensive screening of conditions (see Section S3.3, SI) revealed that iodination was most efficient using radical initiation (chemical or photochemical), whereas bromination favored strongly acidic media. Notably, both reactions could be performed on gram-scale, with >90% yields, and deemed operationally very simple (“dump-and-stir”, max. 6 h reaction time, easy work-up and purification).

**Fig. 2:**
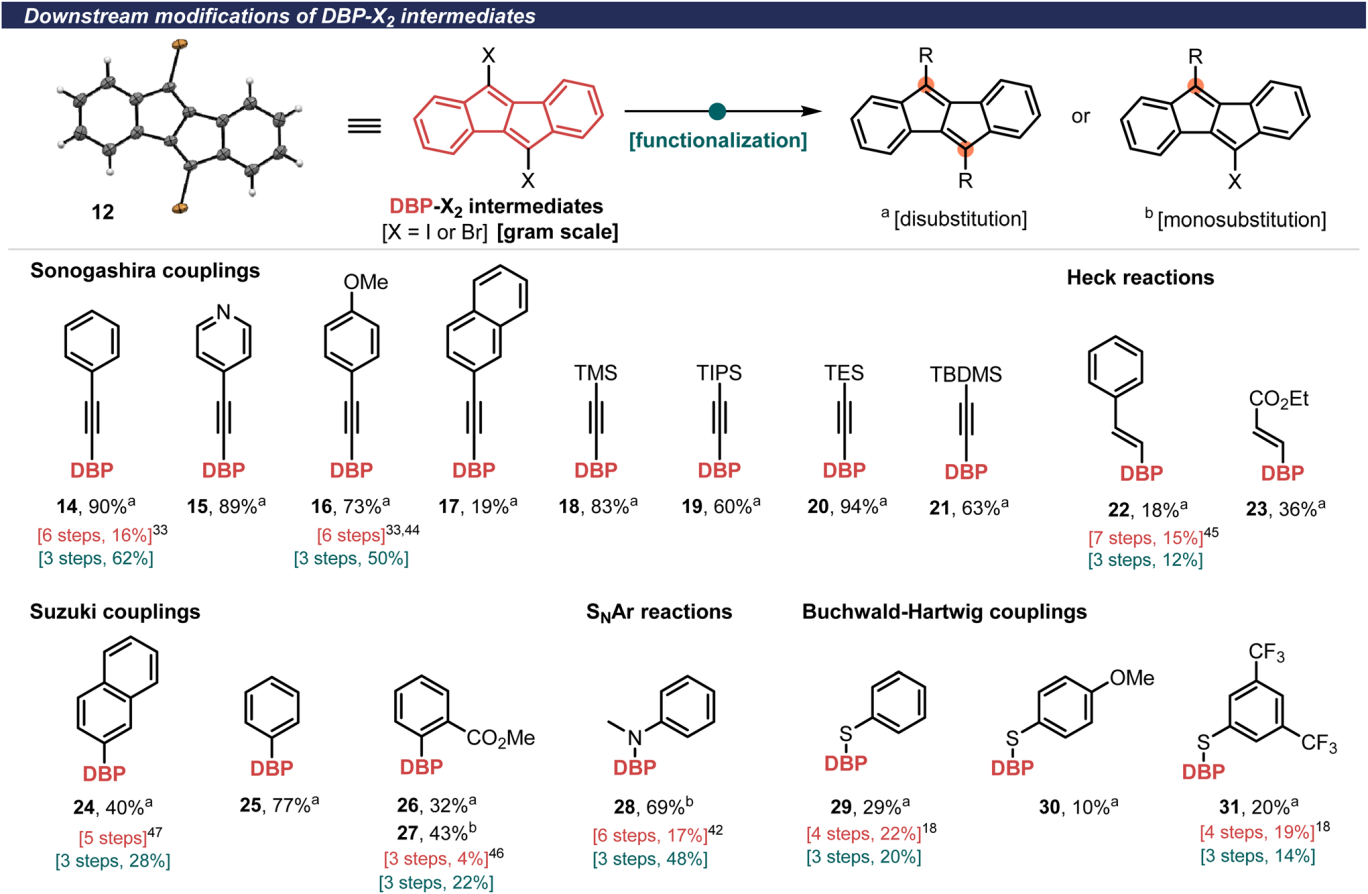
Scope of transformations on the pentalene core.

Dihalo-DBPs are known to be highly useful intermediates and have been used in several studies to prepare valuable DBP-based structures.^33,42,43^ However, provided that the synthesis of halides 12 and 13 required long multistep synthesis (5 steps) or impractical conditions previously,^33,37,38^ limiting material throughput severely. Given the ease of access to these two compounds (12 and 13) enabled by our current approach, we were able to simplify the syntheses of known DBP-based molecules studied in the field of singlet-fission chromophores (14, 16, 22)^44,45^ and organic electronics (26, 29, 31).^18,46^ In addition, new structures and reactivities were also investigated (Fig. 2). The key functional handles in the 5,10-positions enabled multiple types of cross-coupling to form C–C and C-heteroatom bonds directly on the pentalene core. Sonogashira couplings of aryl and silyl-acetylenes (14–21) proceeded smoothly to yield known (14, 16)^44^ and potentially interesting singlet-fission chromophores (17–21). Notably, in case of compound 14 the previous 6-step synthesis was simplified into a 3-step procedure (16% *vs.* 62% overall yield, respectively). We also investigated Heck reactions to prepare DBP-based polyene type structures 22 and 23. Compound 22 has previously been studied as highly efficient singlet-fission chromophore,^45^ however, the reported 7-step synthesis limited all-round structure–property relationship screening. Similarly, Suzuki couplings were performed to give DBP-based compounds 24–27.^47^ For compound 27 monosubstitution was proved to be viable with careful tuning of reaction conditions and subsequent purification simplifying access to non-symmetric aryl-functionalized DBPs. Carbon-heteroatom bonds could be introduced *via* nucleophilic aromatic substitution or Buchwald–Hartwig couplings. This approach allowed the straightforward preparation of tertiary amine 28,^42^ and aryl-sulfides 29–31^18^ bearing functional groups with diverse electronic properties (both EWG and EDG).

In summary, we completed diverse functionalization of the 5,10-positions, enabled by the gram-scale access to building blocks 12 and 13. To further broaden our synthetic platform, in the following, we report the first thianthrenation of an antiaromatic ring system, allowing late-stage functionalization on the annulated benzene rings. Overall, the reported set of transformations enables diverse functionalization of the DBP scaffold both on the pentalene subunit and the terminal benzene rings.

**Scheme 3:**
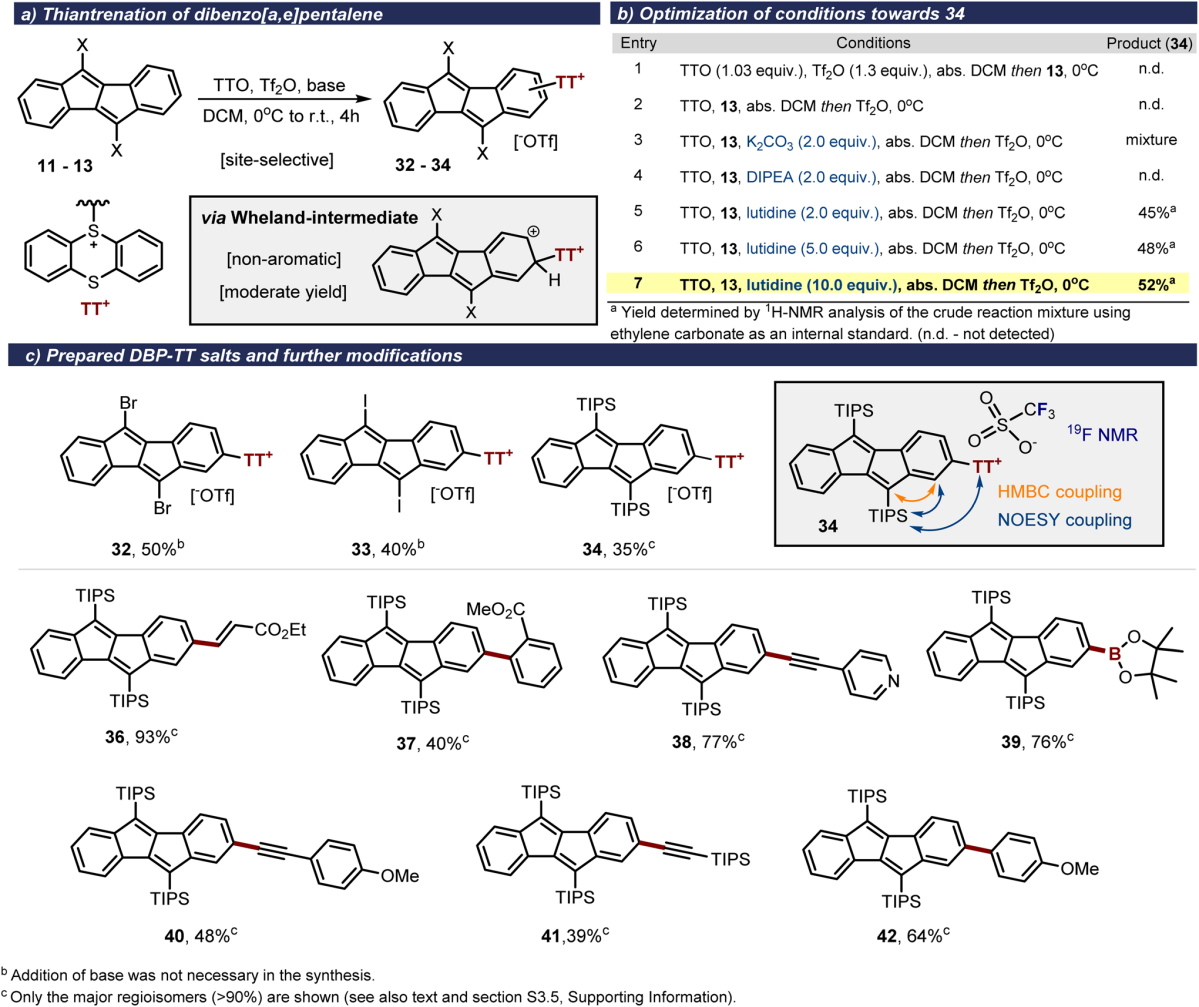
(a) Aryl-thianthrenation of dibenzopentalenes. (b) Optimization of DBP thianthrenation. (c) Functionalization of DBP-thianthrenium salts.

Previously, we attempted multiple different transformations on the annulated benzene rings, such as electrophilic brominations, formylations and Friedel–Crafts type acylation/alkylations. Unfortunately, these attempts all failed, despite the relatively electron-rich nature of the aromatic rings. Inspired by the recent work of Dumele and coworkers,^48^ we turned to aryl thianthrenation of our substrate candidates. This state-of-the-art tool for aryl and alkene functionalization requires the formation of thianthrenium salts (TT^+^-salt) (Scheme 3a) before subsequent downstream modification. Using carefully optimized conditions, based on Ritter's protocol,^49–51^ (Scheme 3b) we achieved monothianthrenation on the DBP-benzene rings, bearing halogens (32, 33) or TIPS groups (34) in the 5,10-positions (Scheme 3c). It has been previously shown that stabilization of the Wheland intermediate is closely related with the efficiency of thianthrenation.^47^ In case of DBP, one finds three non-aromatic charged, and one antiaromatic charged resonance structures (Scheme 3a), resulting in moderate yields for this transformation (up to 52% NMR yield).

Protecting group tolerance was a key challenge during the optimization of conditions as the highly acidic media (during either the reaction or aqueous work-up) resulted in the loss of TIPS and generally in complex crude reaction mixtures. This challenge could be overcome by addition of an internal base (lutidine) and avoidance of aqueous work-up. Preserving the TIPS groups was also crucial for subsequent functionalizations, as we experienced no/very low selectivity for TT^+^ on the benzene rings in the presence of halogens at 5,10-positions in the attempted cross-coupling reactions. This was quite unexpected as previous reports suggest higher reactivity for TT^+^ than for halides in such reactions.^52^ Accordingly, we demonstrated the synthetic versatility of the TIPS-protected DBP-TT^+^ salt 34, which was prepared on gram scale (>1.5 g) and enabled access to several novel DBP structures (Scheme 3c). Notably, thianthrenation of DBP-TIPS was not completely selective; a minor regioisomer (<10%) formed along the major product. These regioisomers could not be separated and used as a mixture in the subsequent transformations (see also Section S3.5, SI). Therefore, yields are reported throughout for the combined mixtures. Unless otherwise noted, the ratio of the major and minor regioisomers is approximately 9 : 1 (Scheme 3a, only major products are shown). The thianthrenation of halogenated DBP derivatives proceeded with complete selectivity.

Pd-catalyzed cross coupling reactions, such as Suzuki, Sonogashira or Heck reactions, all proceeded smoothly on thianthrenium salt 34 (Scheme 3c). Operationally simple, practical conditions allowed the introduction of various substituted aromatic and heteroaromatic groups.^52^ Carbon-heteroatom bond construction was also successful, as conventional Pd-catalyzed Miyaura borylation conditions yielded aryl boronate 39 from the corresponding TT^+^-salt. Importantly, our scope includes all novel structures, several of which can serve as valuable intermediate in further synthesis. Furthermore, this strategy allows for the direct functionalization of the aromatic rings of DBP, thereby eliminating the need to incorporate substituents on these sites at an early stage of the synthetic sequence.

## Conclusions

In conclusion, the study outlined herein described an extensive synthetic platform for diversly substituted dibenzo[*a*,*e*]pentalene derivatives. Rapid, multi-decagram scale construction of the DBP scaffold was achieved from simple starting materials in a reductive radical cyclization reaction. Triisopropyl-silyl groups in the 5,10-pentalene core positions served as surrogates for later halodesilylation, which was first described using conventional reagents under practical conditions (radical, photochemical, acidic). This simple 2-step sequence allowed the preparation of scope of 5,10-functionalized DBPs. Finally, aryl thianthrenation on the annulated benzene rings enabled our platform approach to yield complex, even trisubstituted DBP derivatives.

## Author contributions

Marcell M. Bogner, Bence Sóvári, Péter P. Kalapos, Péter J. Mayer, Márton Hegedűs and Attila Kunfi performed the syntheses. Gábor Turczel performed NMR characterizations. Anna J. Kiss-Szemán and Veronika Harmat determined the crystal structures. Gábor London conceptualized the research. Marcell M. Bogner, Bence Sóvári and Gábor London wrote the manuscript. All authors contributed to the final version of the manuscript.

## Conflicts of interest

The authors declare no conflicts of interest.

## Supplementary Material

RA-016-D6RA03886G-s001

RA-016-D6RA03886G-s002

## Data Availability

CCDC 2503542 (11) and 2503560 (12) contain the supplementary crystallographic data for this paper.^53*a*,*b*^ The data supporting this article have been included as part of the supplementary information (SI). Supplementary information: experimental details and characterization data for products (copies of ^1^H, and ^13^C{^1^H} NMR, and crystal data). See DOI: https://doi.org/10.1039/d6ra03886g.
