# Supplementary material for: Streamlined synthesis of functionalized dibenzo[a,e]pentalenes through potassium-mediated cyclization and late-stage thianthrenation
Source: RSC Adv. 2026 Jun 8;16(33):30958–64. doi: 10.1039/d6ra03886g (PMC13247826; doi:10.1039/d6ra03886g)
Supplement: RA-016-D6RA03886G-s001 [file RA-016-D6RA03886G-s001.pdf]

Supplementary Information (SI) for RSC Advances.
This journal is © The Royal Society of Chemistry 2026

Supporting Information

Streamlined synthesis of functionalized dibenzo[a,e]pentalenes through potassium-mediated cyclization and late-stage thianthrenation

Marcell M. Bogner[1], Bence Sóvári[1], Péter P. Kalapos[1], Péter J. Mayer[1], Márton Hegedűs[1], Gábor Turczel[2], Attila Kunfi[1], Anna J. Kiss-Szemán[3], Veronika Harmat[3,4], Gábor London*[1]

[1]Institute of Organic Chemistry, HUN-REN Research Centre for Natural Sciences, 1117 Budapest, Hungary
[2]Centre for Structural Science, HUN-REN Research Centre for Natural Sciences, 1117 Budapest, Hungary
[3]Laboratory of Structural Chemistry and Biology, Institute of Chemistry, Eötvös Loránd University, Pázmány Péter sétány 1/A, 1117, Budapest, Hungary
[4]HUN-REN ELTE Protein Modelling Research Group, Pázmány Péter sétány 1/A, 1117, Budapest, Hungary
*Corresponding Author Email: london.gabor@ttk.hu

Table of Contents

S1 Materials and methods 3

S2 Summary of previous synthetic routes towards 5,10-disubstituted dibenzo[a,e]pentalenes 5

S3 Experimental protocols and product characterization 6

S3.1 Preparation of aryl acetylenes 6

S3.2 Preparation of diareno[*a*,*e*]pentalenes 15

S3.2.1 Reaction optimization 16

S3.2.2 Limitations 17

S3.2.3 Mechanistic overview 17

S3.2.4 Attempted molten metal synthesis 18

S3.3 Halodesilylation – Primary product valorisation 19

S3.3.1 Halodesilylation – Iodination 19

S3.3.2 Halodesilylation – Bromination 21

S3.4 Functionalization of dihalo-DBPs - Secondary product valorisation 23

S3.5 Thianthrenation 35

S3.5.1 Optimization of reaction conditions 35

S3.5.1.1 Formation of regioisomeric products upon thianthrenation of **11** and subsequent functionalizations 36

S3.5.2 Experimental procedures and product characterization 37

S3.5.3 Thianthrenium salt functionalization 39

S3.6 Further attempted transformations of dihalo-DBPs 45

S4 X-ray crystallographic data 46

S5 NMR spectra 49

S6 References 65

S1 Materials and methods

Reagents and solvents

Commercial reagents, solvents, and catalysts (Sigma-Aldrich, Fluorochem, and VWR) of reagent grade were purchased and used without further purification. Solvents for extraction or column chromatography were of a technical quality. Organic solutions were concentrated by rotary evaporation at 40 °C, unless otherwise stated.

Chromatographic methods

Thin-layer chromatography was carried out on "Merck silica gel 60 F_{254}" or "Merck aluminium oxide 60 F_{254} neutral" type UV-active silica or alumina sheets. Compounds were visualized using UV light (254 nm or 365 nm) or TLC stain solutions (e.g., $KMnO_4$, cerium ammonium molybdate (CAM) or 2,4-dinitrophenylhydrazine (DNPH)). Column chromatography was performed using a Teledyne Isco CombiFlash Rf+ automated flash chromatographer with "RediSep R_f GOLD" silica gel or basic alumina column at 25 (± 1)°C. The cartridge was filled with Zeochem "ZEOprep 60 25–40 µm" silica gel or EcoChrom "MP Alumina B - Super I" basic alumina.

High performance liquid chromatography

Analytical RP-HPLC-UV/vis-MS measurements were carried out using a Shimadzu LCMS 2020 instrument applying a Gemini C18 column (100 mm × 2.00 mm I.D.) in which the stationary phase was 5 µm silica with a pore size of 110 Å. The chromatograms were recorded with a UV–vis diode array (190–800 nm) and an ESI-MS detector. The following linear gradient elution profile was applied for the LC–MS measurements: 0% → 100% B in 6.5 min then 100% → 0% B in 0.5 min, then 0% B for 1 min with eluents A (2% HCOOH, 5% CH_3CN and 93% water) and B (2% HCOOH, 80% CH_3CN and 18% water) at a flow rate of 0.8 mL/min at 40°C.

Gas chromatography-mass spectrometry

Gas chromatography–mass spectrometry (GC-MS) analyses were performed on a Shimadzu GC-2010 instrument equipped with a GCMS-QP2010 Ultra detector, using EI ionization mode. The analytical method used a Zebron ZB-5MSi column (30 m, ID: 0.25 mm, df: 0.25 µm), helium as a carrier gas, and an oven temperature profile as follows: 0 min.: 80°C; 11 min.: 250°C; 16 min.: 250°C. Further parameters: injector temperature: 250°C; gas flow rate: 27.7 mL/min.; split ratio: 1/20, detector temperature: 280°C.

NMR spectroscopy

NMR spectra were acquired on a Varian 600 (^{1}H 600 MHz, ^{13}C 151 MHz) Varian 500 (^{1}H 500 MHz, ^{13}C 126 MHz) or a Varian 300 (^{1}H 300 MHz, ^{13}C 75 MHz, ^{19}F 282 MHz) NMR spectrometer. The residual solvent peaks were used as the internal reference. Chemical shifts (δ) are reported in ppm. The following abbreviations are used to indicate the multiplicity in ^{1}H NMR spectra: s, singlet; d, doublet; t, triplet; m, multiplet. ^{13}C NMR spectra were acquired in a broadband decoupled mode. All NMR spectra were recorded at 30 °C.

High resolution mass spectroscopy

High-resolution mass spectrometry measurements were performed on a Sciex TripleTOF 5600+ high-resolution tandem mass spectrometer equipped with a DuoSpray ion source. APCI or ESI ionization was applied in positive ion detection mode. Samples were dissolved in acetonitrile and flow injected into the acetonitrile/water 1:1 flow. The flow rate was 0.2 mL/min. The resolution of the mass spectrometer was 35,000.

S2 Summary of previous synthetic routes towards 5,10-disubstituted dibenzo[a,e]pentalenes

The following scheme (Scheme S1) details the synthetic campaigns[1-5] featured in Figure 1. in the main text.

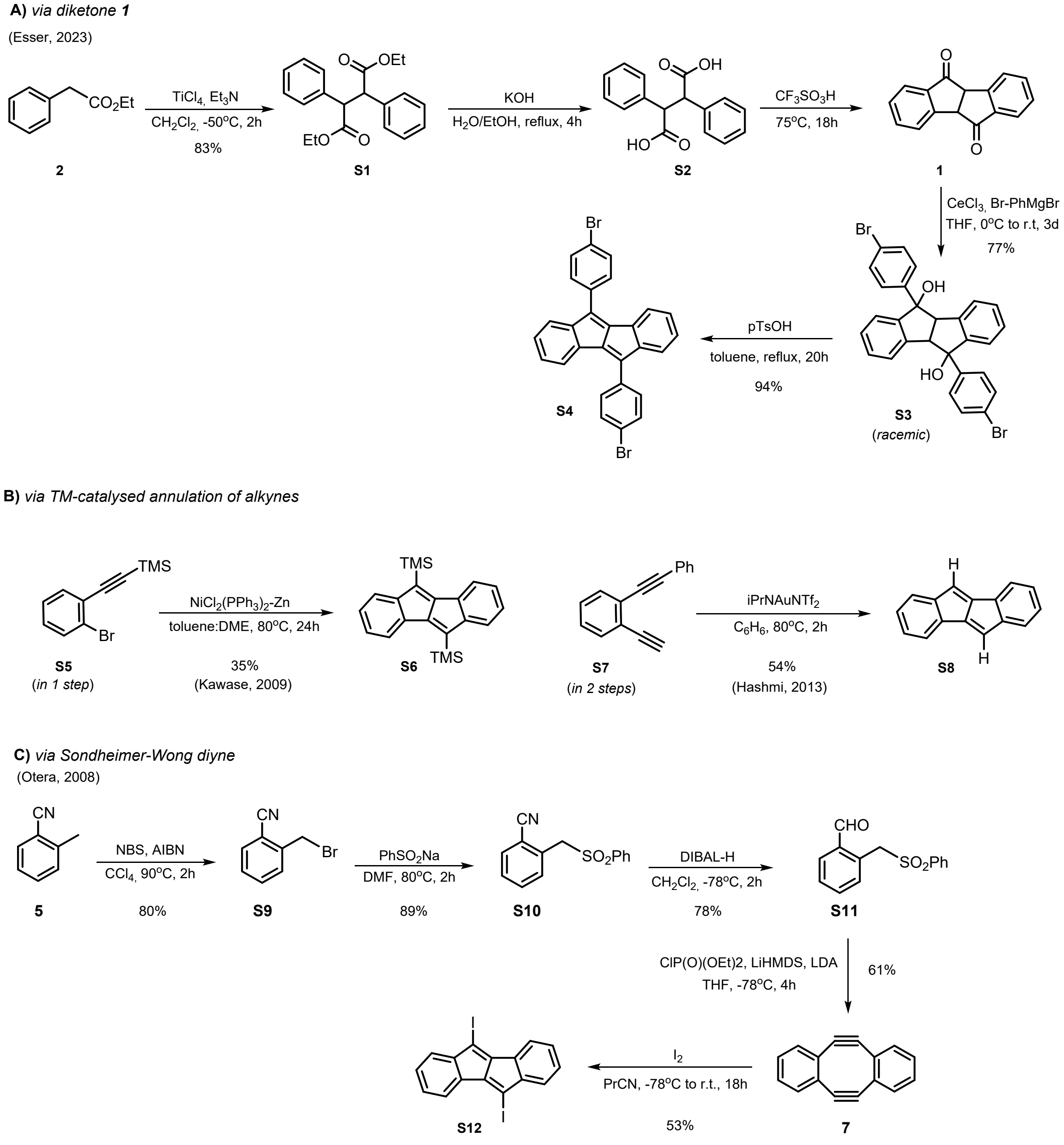

Scheme S1. Previously reported routes to *5,10*-disubstituted dibenzo[*a,e*]pentalenes.

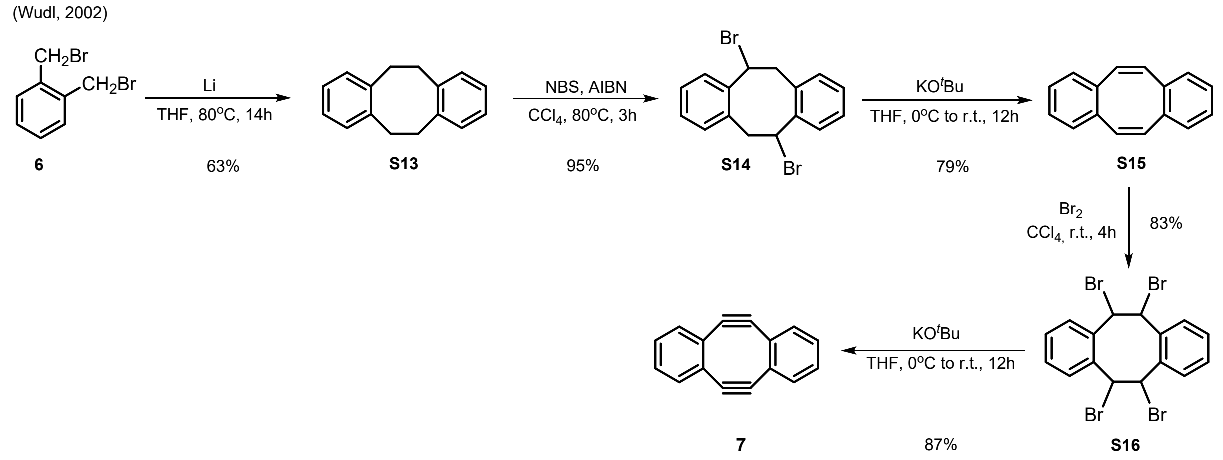

Scheme S1. (cont) Previously reported routes to *5,10*-disubstituted dibenzo[*a,e*]pentalenes.

S3 Experimental protocols and product characterization

S3.1 Preparation of aryl acetylenes

To explore the scope of the potassium mediated dimerization we prepared a set of aryl acetylenes with different substituents and (hetero)aromatic ring structures (Figure S1).

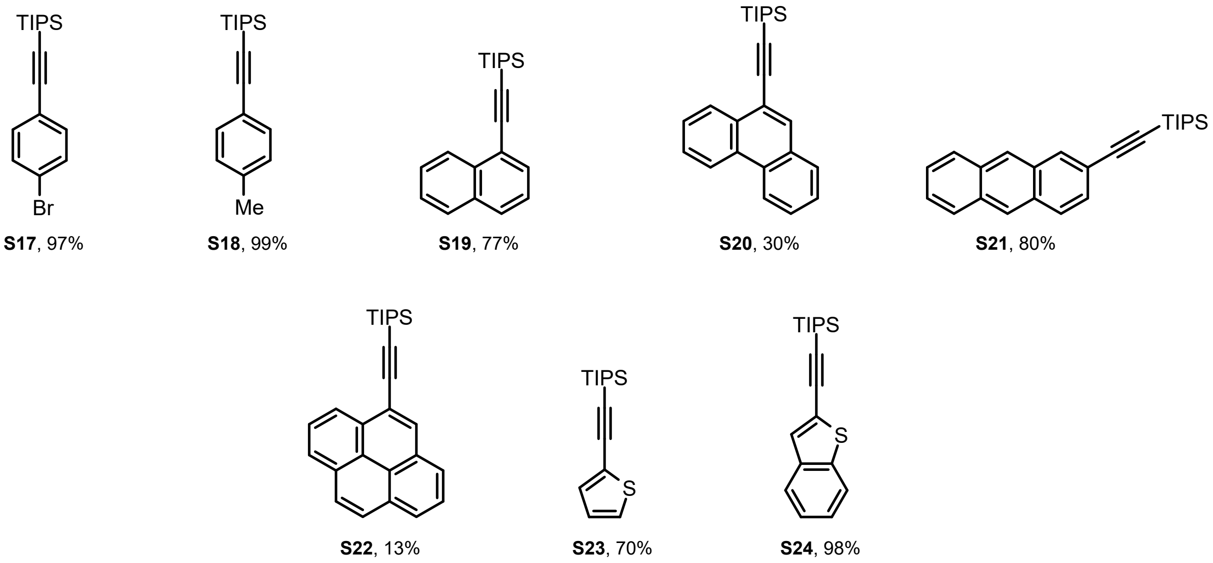

Figure S1. Aryl acetylenes prepared *via* Sonogashira coupling.

Aryl acetylenes **S17** – **S24** were prepared via Sonogashira coupling of the corresponding aryl bromide/iodide and (triisopropylsilyl)acetylene. A typical procedure for the preparation of **S17** is shown below:

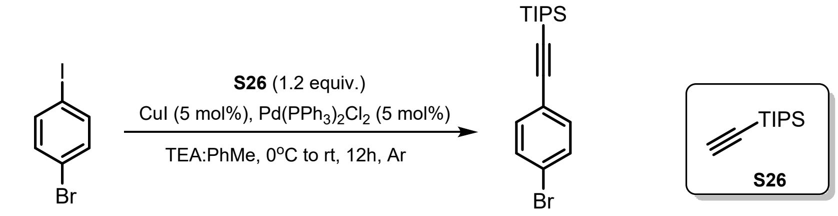

S25 (1.0 equiv.) **S17**

General Procedure A: An oven-dried 60 mL scintillation vial was charged with Pd-catalyst (5 mol%), CuI (5 mol%) (also aryl-halide if solid) and purged with argon. Subsequently, the solid components were dissolved in a mixture of degassed THF and triethylamine (TEA, V/V = 1:1), and trialkylsilyl acetylene (1.2 equiv.) (also aryl-halide if liquid) was added via syringe. The resulting mixture was stirred at room temperature overnight. After completion (monitored by TLC analysis) the mixture was diluted with EtOAc, filtered over a pad of celite and concentrated under reduced pressure. The crude product was further purified by column chromatography (SiO_2, hexanes) affording the desired products as colorless oils.

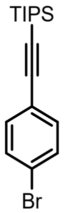

S17

Compound S17: product obtained according to General Procedure A; as a colorless oil. **Yield:** 2.90 g (97%).

TLC: $R_f = 0.77$ (hexanes)

^{1}H NMR (CDCl$_3$, 500 MHz): δ = 7.43 (d, J = 8.50 Hz, 2H), 7.33 (d, J = 8.49 Hz, 2H), 1.12 (s, 21H) ppm.

^{13}C NMR (CDCl$_3$, 126 MHz): δ = 133.6, 131.6, 122.67, 122.65, 106.0, 92.2, 18.8, 11.5 ppm.

MS (EI): m/z (rel. int.) = 336.1 ($[M]^+$, 11); 295.0 (100); 293.0 (99); 224.95 (58).

HRMS (APCI-TOF): m/z calcd. for $[C_{14}H_{18}SiBr]^+$ ($[M-^iPr]^+$) 293.0355, found 293.0348.

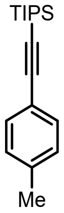

S18

Compound S18: product obtained according to General Procedure A; as a colorless oil. **Yield:** 620 mg (99%).

TLC: $R_f = 0.73$ (hexanes)

^{1}H NMR (CDCl$_3$, 500 MHz): δ = 7.38 (d, J = 8.13 Hz, 2H), 7.11 (d, J = 8.06 Hz, 2H), 2.35 (s, 3H), 1.14 (s, 21H) ppm.

^{13}C NMR (CDCl$_3$, 126 MHz): δ = 138.5, 132.1, 129.1, 123.6, 120.7, 107.5, 89.7, 21.6, 18.8, 11.5 ppm.

MS (EI): *m/z* (rel. int.) = 272.15 ([M]$^+$, 10); 229.05 (100); 159.05 (60); 173.05 (41).

HRMS (APCI-TOF): *m/z* calcd. for [$C_{15}H_{21}Si$]$^+$ ([M-^{i}Pr]$^+$) 229.1407, found 229.1396.

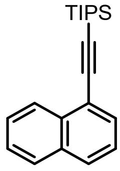

S19

Compound S19: product obtained according to General Procedure A; as a colorless oil. **Yield:** 1.18 g (77%).

TLC: R$_f$ = 0.72 (hexanes) (purple fluorescence under 254 nm)

^{1}H NMR (CDCl$_3$, 500 MHz): δ = 8.42 (d, J = 8.5 Hz, 1H), 7.84 (dd, J = 12.2, 8.2 Hz, 2H), 7.74 (dd, J = 7.1, 1.3 Hz, 1H), 7.59 (t, J = 7.6 Hz, 1H), 7.54 – 7.51 (m, 1H), 7.42 (t, J = 7.7 Hz, 1H), 1.23 (s, 21H) ppm.

^{13}C NMR (CDCl$_3$, 121 MHz): δ = 133.7, 133.3, 131.2, 128.9, 128.4, 127.0, 126.48, 126.46, 125.3, 121.4, 105.2, 95.9, 18.9, 11.6 ppm.

MS (EI): *m/z* (rel. int.) = 308.15 ([M]$^+$, 39); 309.15 ([M+H]$^+$, 11); 265.10 (100); 195.05 (66); 223.05 (45).

HRMS (APCI-TOF): *m/z* calcd. for [$C_{21}H_{28}Si$]$^+$ ([M]$^+$) 308.1960, found 308.1949.

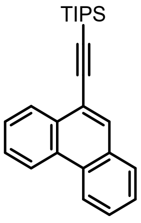

S20

Compound S20: product obtained according to General Procedure A; as a colorless oil. **Yield:** 209 mg (30%).

TLC: R$_f$ = 0.63 (hexanes)

^{1}H NMR (CDCl$_3$, 500 MHz): δ = 8.68 (m, 1H); 8.65 (d, J = 8.3 Hz, 1H); 8.51 (m, 1H); 8.04 (s, 1H); 7.85 (d, J = 7.8 Hz, 1H); 7.71-7.64 (m, 3H); 7.59 (t, J = 7.2 Hz, 1H); 1.24 (s, 21H) ppm.

^{13}C NMR ($CDCl_3$, 126 MHz): δ = 132.6, 131.5, 131.4, 130.5, 130.2, 128.6, 127.6, 127.23, 127.21, 127.14, 127.06, 122.9, 122.8, 120.1, 105.3, 95.7, 19.0, 11.6 ppm.

HRMS (APCI-TOF): *m/z* calcd. for $[C_{25}H_{30}Si]^+$ ($[M]^+$) 358.2111, found 358.2106.

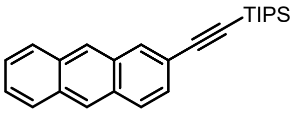

S21

Compound S21: product obtained according to General Procedure A; as a pale-yellow amorphous solid. **Yield:** 560 mg (80%).

TLC: R_f = 0.51 (hexanes)

^{1}H NMR ($CDCl_3$, 500 MHz): δ = 8.37 (s, 2H), 8.20 (s, 1H), 8.02 – 7.97 (m, 2H), 7.93 (d, J = 8.7 Hz, 1H), 7.52 – 7.47 (m, 3H), 1.27 – 1.21 (m, 21H) ppm.

^{13}C NMR ($CDCl_3$, 126 MHz): δ = 132.5, 132.3, 132.2, 131.1, 130.9, 128.4, 128.3, 128.2, 128.1, 126.4, 126.3, 125.9, 125.8, 120.4, 108.0, 91.8, 18.9, 11.6 ppm.

HRMS (APCI-TOF): *m/z* calcd. for $[C_{25}H_{31}Si]^+$ ($[M+H]^+$) 359.2189, found 359.2182.

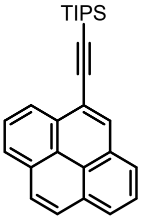

S22

Compound S22: product obtained according to General Procedure A; as a green-yellow crystalline solid. **Yield:** 88.0 mg (13%).

TLC: R_f = 0.41 (hexanes) (green fluorescence under 366 nm)

^{1}H NMR ($CDCl_3$, 500 MHz): δ = 8.63 (d, J = 9.06 Hz, 1H); 8.23-8.15 (m, 4H); 8.08 (d, J = 9.61 Hz, 2H); 8.03 (m, 2H); 1.27 (s, 21H) ppm.

^{13}C NMR ($CDCl_3$, 126 MHz): δ = 132.4, 131.42, 131.39, 131.2, 130.3, 128.6, 128.3, 127.4, 126.3, 125.8, 125.72, 125.67, 124.6, 124.50, 124.45, 118.3, 106.2, 96.9, 19.0, 11.7 ppm.

HRMS (APCI-TOF): *m/z* calcd. for $[C_{27}H_{31}Si]^+$ ($[M+H]^+$) 383.2189, found 383.2204.

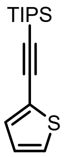

S23

Compound S23: product obtained according to General Procedure A; as a colorless oil. **Yield:** 280 mg (70%).

TLC: $R_f = 0.74$ (hexanes)

^{1}H NMR ($CDCl_3$, 500 MHz): δ = 7.24-7.21 (m, 2H); 6.95 (t, J = 3.7 Hz, 1H); 1.14 (s, 21H) ppm.

^{13}C NMR ($CDCl_3$, 126 MHz): δ = 132.5, 127.1, 126.9, 123.9, 99.5, 95.5, 18.8, 11.5 ppm.

The NMR spectra are in accordance with literature data.[6]

MS (EI): *m/z* (rel. int.) = 264.15 ($[M]^+$, 15); 221.05 (100); 151.0 (51); 165.05 (29).

HRMS (APCI-TOF): *m/z* calcd. for $[C_{12}H_{17}SiS]^+$ ($[M\text{-}^i Pr]^+$) 221.0814, found 221.0806.

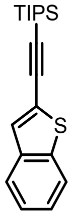

S24

Compound S24: product obtained according to General Procedure A; as a pale-yellow oil. **Yield:** 360 mg (98%).

TLC: $R_f = 0.69$ (hexanes)

^{1}H NMR ($CDCl_3$, 500 MHz): δ = 7.76-7.71 (m, 2H); 7.46 (s, 1H); 7.37-7.33 (m, 2H); 1.16 (s, 21H) ppm.

^{13}C NMR ($CDCl_3$, 126 MHz): δ = 140.3, 139.2, 129.3, 125.6, 124.8, 123.9, 123.7, 122.1, 99.8, 98.0, 18.8, 11.5 ppm.

MS (EI): *m/z* (rel. int.) = 314.15 ($[M]^+$, 44); 315.15 ($[M+H]^+$, 12); 271.10 (100); 201.0 (70); 229.0 (46).

HRMS (APCI-TOF): *m/z* calcd. for $[C_{16}H_{19}SiS]^+$ ($[M\text{-}^i Pr]^+$) 271.0971, found 271.0964.

Aryl acetylenes **S27** – **S32** were prepared via protection of the corresponding aryl acetylene (Figure S2).

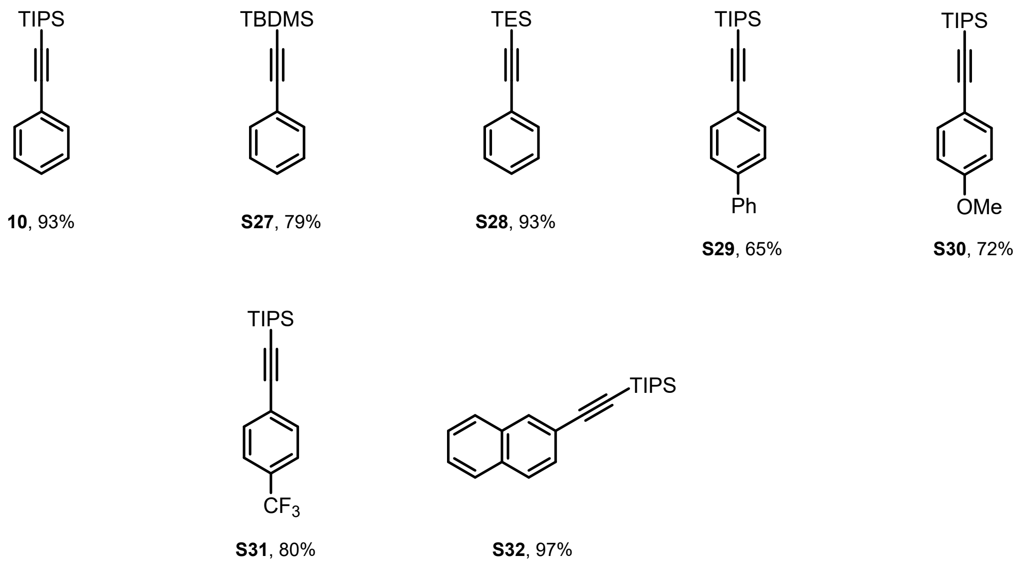

Figure S2. Aryl acetylenes prepared *via* protection of terminal acetylenes.

A typical procedure for the preparation of **10** is shown below:

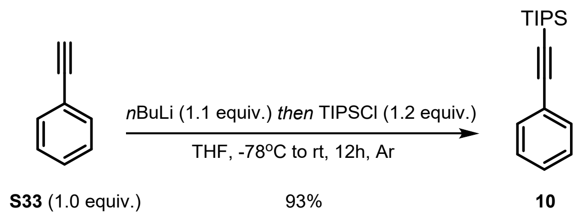

General Procedure B: An oven-dried 60 mL scintillation vial was purged with dry nitrogen and the phenylacetylene (1.0 equiv.) was added either via a syringe or as a solid under continuous nitrogen flow. Subsequently, anhydrous THF was added, and the resulting solution was cooled to -78 °C. Under these conditions *n*-BuLi (1.1 equiv) (1.6 M in hexanes) was added dropwise and the mixture was stirred for 30 minutes. Next, triisopropylsilyl chloride (1.2 equiv.) was added, and the mixture was left to warm to room temperature overnight. The reaction was quenched with sat. NH_4Cl solution and the phases were separated. The aqueous phase was extracted with ethyl acetate, and the combined organic phase was washed with brine and dried over anhydrous $MgSO_4$. Subsequent filtration and evaporation of the solvent under reduced pressure yielded the crude products. The crude (often yellowish discoloured) products were filtered through a pad of silica using hexane as eluent. The solutions were concentrated under reduced pressure yielding the desired products as colourless oils.

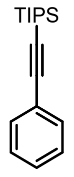

10

Compound 10: product obtained according to General Procedure B; as a colorless oil. **Yield:** 23.6 g (93%).

TLC: R_f = 0.82 (hexanes)

^{1}H NMR (CDCl$_3$, 500 MHz): δ = 7.48 (m, 2H), 7.30 (m, 3H), 1.14 (s, 21H) ppm.

^{13}C NMR (CDCl$_3$, 126 MHz): δ = 132.10, 128.35, 128.24, 123.68, 107.26, 90.46, 18.75, 11.44 ppm.

MS (EI): *m/z* (rel. int.) = 258.15 ([M]$^+$, 8); 215.10 (100); 145.10 (60); 159.05 (50).

HRMS (APCI-TOF): *m/z* calcd. for [$C_{14}H_{19}Si$]$^+$ ([M-^{i}Pr]$^+$) 215.1250, found 215.1243.

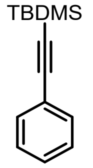

S27

Compound S27: product obtained according to General Procedure B; as a colorless oil. **Yield:** 1.68 g (79%).

TLC: R_f = 0.64 (hexanes)

^{1}H NMR (CDCl$_3$, 500 MHz): δ = 7.47 (m, 2H), 7.30 (m, 2H), 1.00 (s, 9H), 0.18 (s, 6H) ppm.

^{13}C NMR (CDCl$_3$, 126 MHz): δ = 132.2, 128.6, 128.3, 123.5, 105.9, 92.6, 26.3, 16.9, -4.4 ppm.

MS (EI): *m/z* (rel. int.) = 216.10 ([M]$^+$, 20); 217.1 ([M+H]$^+$, 4); 159.15 (100); 160.05 (81); 129.05 (18).

HRMS (APCI-TOF): *m/z* calcd. for [$C_{10}H_{11}Si$]$^+$ ([M-^{t}Bu]$^+$) 159.0624, found 159.0619.
m/z calcd. for [$C_{14}H_{20}Si$]$^+$ ([M]$^+$) 216.1328, found 216.1319.

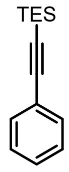

S28

Compound S28: product obtained according to General Procedure B; as a colorless oil. **Yield:** 1.96 g (93%).

TLC: R_f = 0.67 (hexanes)

^{1}H NMR (CDCl$_3$, 500 MHz): δ = 7.47 (m, 2H), 7.30 (m, 3H), 1.05 (t, J = 7.90 Hz, 9H), 0.68 (q, J = 7.90 Hz, 6H) ppm.

^{13}C NMR (CDCl$_3$, 126 MHz): δ = 132.2, 128.5, 128.3, 123.6, 106.6, 91.7, 7.6, 4.6 ppm.

MS (EI): *m/z* (rel. int.) = 216.10 ([M]$^+$, 10); 187.05 (100); 159.05 (96); 131.05 (77).

HRMS (APCI-TOF): *m/z* calcd. for [$C_{12}H_{15}Si$]$^+$ ([M-Et]$^+$) 187.0937, found 187.0932.

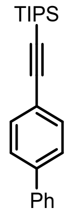

S29

Compound S29: product obtained according to General Procedure B; as a colorless oil. **Yield:** 618 mg (65%).

TLC: R_f = 0.50 (hexanes)

^{1}H NMR (CDCl$_3$, 500 MHz): δ = 7.56 (m, 6H), 7.45 (t, J = 7.74 Hz, 2H), 7.36 (m, 1H), 1.15 (s, 21H) ppm.

^{13}C NMR (CDCl$_3$, 126 MHz): δ = 141.2, 140.6, 132.6, 129.0, 127.8, 127.2, 127.0, 122.7, 107.2, 91.4, 18.8, 11.5 ppm.

HRMS (APCI-TOF): *m/z* calcd. for [$C_{23}H_{30}Si$]$^+$ ([M]$^+$) 334.2111, found 334.2106.

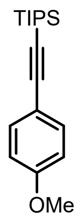

S30

Compound S30: product obtained according to General Procedure B; as a colorless oil. **Yield:** 1.18 g (72%).

TLC: R_f = 0.73 (hexanes / EtOAc, 5 : 1 v/v); R_f = 0.21 (hexanes)

^{1}H NMR (CDCl$_3$, 500 MHz): δ = 7.41 (d, J = 9.06 Hz, 2H), 6.82 (d, J = 9.06 Hz, 2H), 3.81 (s, 3H), 1.12 (s, 21H) ppm.

^{13}C NMR (CDCl$_3$, 126 MHz): δ = 159.8, 133.7, 116.0, 114.0, 107.3, 88.8, 55.5, 18.8, 11.6 ppm.

HRMS (APCI-TOF): *m/z* calcd. for $[C_{18}H_{29}OSi]^+$ ($[M+H]^+$) 289.1982, found 289.1985.

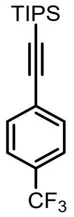

S31

Compound S31: product obtained according to General Procedure B; as a colorless oil. **Yield:** 770 mg (80%).

TLC: R_f = 0.86 (hexanes)

^{1}H NMR (CDCl$_3$, 500 MHz): δ = 7.60-7.53 (m, 4H), 1.15 (s, 21H) ppm.

^{13}C NMR (CDCl$_3$, 126 MHz): δ = 132.4, 127.5, 125.3 (q, J = 4.0 Hz, 2C), 105.7, 94.0, 18.8, 11.4 ppm.

^{19}F NMR (CDCl$_3$, 282 MHz): δ = -62.9 (s) ppm.

MS (EI): *m/z* (rel. int.) = 326.10 ($[M]^+$, 8); 283.05 (100); 212.95 (97); 227.0 (89).

HRMS (APCI-TOF): *m/z* calcd. for $[C_{15}H_{18}F_3Si]^+$ ($[M-{}^iPr]^+$) 283.1124, found 283.1118.

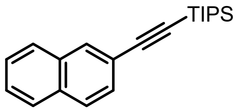

S32

Compound S32: product obtained according to General Procedure B; as a colorless oil. **Yield:** 1.98 g (97%).

TLC: R_f = 0.83 (hexanes)

^{1}H NMR (CDCl$_3$, 500 MHz): δ = 8.00 (s, 1H), 7.80 (td, J = 9.67, 6.40, 3.44 Hz, 2H), 7.76 (d, J = 8.47 Hz, 1H), 7.52 (dd, J = 8.44, 1.61 Hz, 1H), 7.48 (m, 2H), 1.17 (s, 21H) ppm.

^{13}C NMR (CDCl$_3$, 126 MHz): δ = 133.1, 133.0, 132.0, 129.0, 128.0, 127.88, 127.87, 126.8, 126.6, 121.0, 107.6, 91.1, 18.9, 11.6 ppm.

MS (EI): *m/z* (rel. int.) = 308.20 ($[M]^+$, 31); 309.15 ($[M+H]^+$, 9); 265.15 (100); 195.05 (68); 209.05 (51).

HRMS (APCI-TOF): *m/z* calcd. for $[C_{18}H_{21}Si]^+$ ($[M-^iPr]^+$) 265.1407, found 265.1398.

S3.2 Preparation of diareno[*a*,*e*]pentalenes

Representative reaction conditions for the preparation of dibenzo[*a*,*e*]pentalenes:

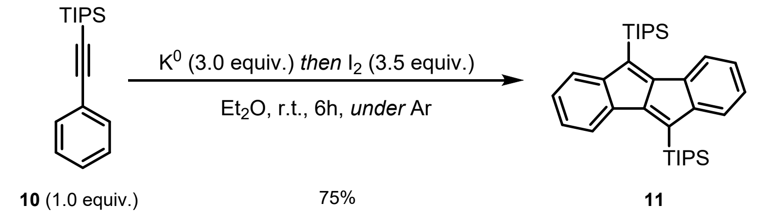

General Procedure C: An oven-dried 50 mL round-bottom flask was charged with metallic potassium under argon and anhydrous diethyl ether was added via syringe. Subsequently, trialkylsilyl-protected phenylacetylene was added and the resulting mixture was stirred at room temperature overnight. After completion (monitored by TLC analysis) iodine (0.4 M in Et_2O) was added to the mixture and the dark red solution was stirred for a further 60 minutes. Finally, the reaction mixture was transferred into an Erlenmeyer-flask and was quenched with an aqueous solution of Na_2SO_3 (1.0 M). The phases were separated, the aqueous phase was extracted with diethyl ether, and the combined organic phase was washed with brine. This solution was dried over $MgSO_4$ and concentrated under reduced pressure to give the crude product as a dark red tar.

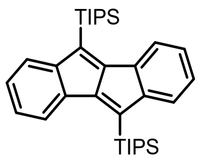

11

Compound 11: product obtained according to General Procedure C; as a red solid. **Yield:** 2.25 g, (75%); (25.6 g, 50% on *large scale, recrystallized*)

TLC: $R_f = 0.68$ (hexanes)

^{1}H NMR (CDCl$_3$, 500 MHz): $\delta = 7.29$ (d, $J = 7.3$ Hz, 2H); 7.15 (d, $J = 7.3$ Hz, 2H); 6.91-6.81 (m, 4H); 1.70 (hept, $J = 7.5$ Hz, 6H); 1.24 (d, $J = 7.6$ Hz, 36H) ppm.

^{13}C NMR (CDCl$_3$, 126 MHz): $\delta = 162.0$, 156.3, 140.2, 136.5, 127.9, 126.2, 125.1, 123.5, 19.5, 13.4 ppm.

HRMS (APCI-TOF): *m/z* calcd. for $[C_{34}H_{50}Si_2]^+$ ($[M]^+$) 514.3445, found 514.3449.

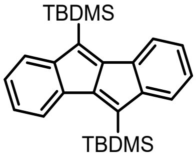

S33

Compound S33: product obtained according to General Procedure C; as a red solid. **Yield:** 117.5 mg (12%).

TLC: $R_f = 0.51$ (hexanes)

^{1}H NMR (CDCl$_3$, 500 MHz): δ = 7.23 (d, J = 7.18 Hz, 1H), 7.01 (d, J = 7.27 Hz, 1H), 6.84 (td, J = 7.49, 7.46, 1.33 Hz, 1H), 6.80 (td, J = 7.49, 7.46, 1.25 Hz, 1H), 1.03 (s, 9H), 0.41 (s, 6H) ppm.

^{13}C NMR (CDCl$_3$, 126 MHz): δ = 160.5, 156.1, 140.1, 135.9, 128.1, 126.3, 125.3, 123.7, 27.3, 19.2, -2.1 ppm.

HRMS (APCI-TOF): *m/z* calcd. for $[C_{28}H_{38}Si_2]^+$ ($[M]^+$) 430.2506, found 430.2504.

S3.2.1 Reaction optimization

Table S1. Optimization of alkali metal reagent.[Note 1]

Entry	Alkali metals[Note 2]	Product [%]
1	magnesium (Mg) [Note 3]	n.d.
2	lithium (Li) [Note 4]	trace[Note 5]
3	sodium (Na)	n.d.
4	potassium (K)	75

[Note 1]: Optimization was performed using (triisopropylsilyl)phenylacetylene (**10**).
[Note 2]: All metals were used in 3 equivalents, unless otherwise noted.
[Note 3]: Magnesium (Mg) was used in the form of turnings.
[Note 4]: Lithium was applied in 10 equivalents and reaction time was 3 days.
[Note 5]: Reaction with lithium (Li) resulted in complex, inseparable product mixture, trace desired product was detected in NMR.

Table S2. Optimization of trialkylsilyl protecting groups.

Entry	Protecting group (-SiR$_3$)	Product [%]
1	trimethylsilyl (TMS)	n.d. [Note 1]
2	triisopropyl (TIPS)	75
3	triethylsilyl (TES)	n.d. [Note 2]
4	*tert*-butyldimethylsilyl (TDBDMS)	12

[Note 1]: DBP-TMS reacted with iodine, to give DBP-I$_2$ (**13**), therefore isolated yield could not be determined (n.d.). Overall yield for 2 steps is 10%.
[Note 2]: DBP-TES reacted with iodine, to give DBP-I$_2$ (**13**), therefore isolated yield could not be determined (n.d.). Overall yield for 2 steps is 5%.

Table S3. Optimization of solvent. [Note 1], [Note 2]

Entry	Solvents	Product [%]
1	tetrahydrofuran (THF)	approx. 60
2	diethyl ether (Et_2O)	75[Note 3]

[Note 1]: Solvents were freshly distilled from sodium, low water content was indicated by benzophenone.
[Note 2]: Protic and halogenated solvents should be avoided when working with elemental potassium!
[Note 3]: Use of anhydrous diethyl ether (Et_2O) resulted in cleaner reaction profile and slightly increased yields.

S3.2.2 Limitations

Substrates **S17** – **S32** failed to give the desired cyclized pentalene products, with several different outcomes:

1. Substrate is unreactive, remains mostly intact, starting material could be recovered. (**S19 – S24, S29, S32**).
2. Substrate showed alternative reactivity (e.g. reductive pathway) (**S30**) (Scheme S2).
3. Complex, inseparable product mixture was formed, desired product could not be obtained/identified. **(S17, S18, S31)**

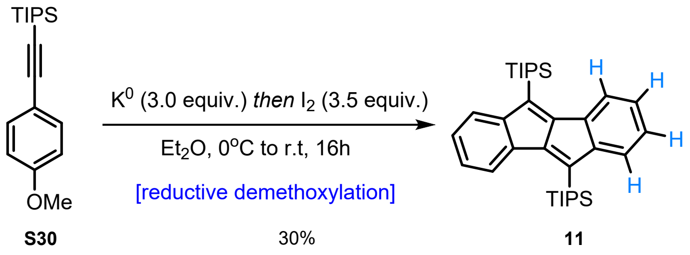

Scheme S2. Reactivity of compound **S30** in the presence of potassium.

S3.2.3 Mechanistic overview

Proposed mechanism of potassium mediated acetylene dimerization was first described by Saito *et. al.*[7] depicted below in Scheme S3. The first step involves a formal Birch reduction of the ethynyl motif to from radical anion **S34**. Subsequent dimerization of this species (**S34**) forms tricyclic intermediate **S35**, which undergoes hydrogen atom abstraction, formally a loss of H_2. Finally, isomerization of the central eight-membered ring produces potassium-pentalenide salt **S36**, which is later quenched using iodine to afford the dibenzo[*a,e*]pentalene scaffold.

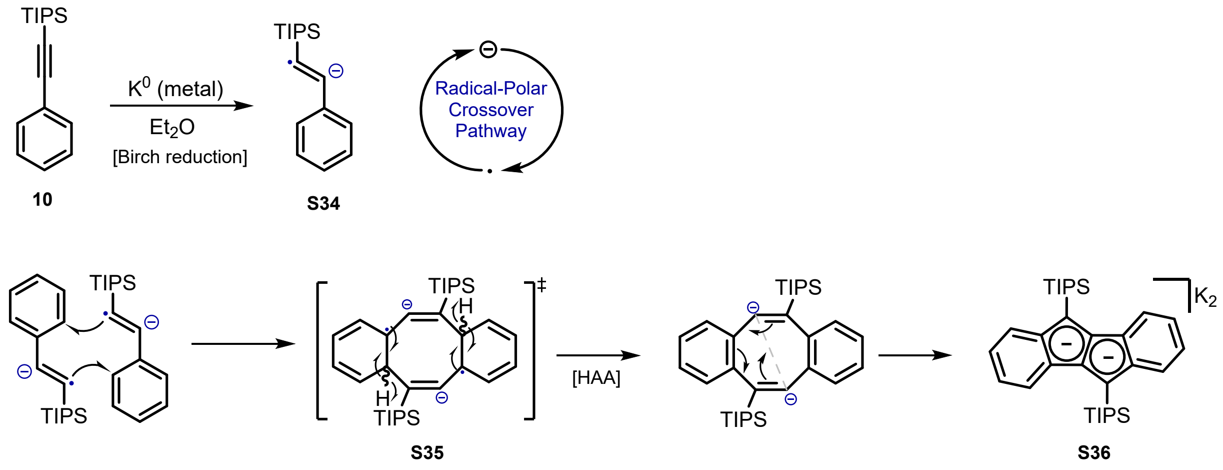

Scheme S3. Plausible mechanism of the acetylene dimerization reaction proposed by Saito and co-workers.[7]

S3.2.4 Attempted molten metal synthesis

We were inspired by the very recent work of Docherty and co-workers[8] on the construction of nanographenes enabled using elemental potassium. In their communication they described a rapid approach to a wide range of polyaromatic hydrocarbons (PAHs) by grinding the starting material with molten potassium metal. Using this method, they could efficiently construct 5-, and 6-membered rings leveraging this alkali mediated cyclodehydrogenation. Previously, our studies only involved solid potassium in heterogenous alkali metal-solution mixtures, but after this "demonstration" we explored this method for the synthesis of dibenzo[*a,*e]pentalenes from corresponding aryl-acetylenes.

Procedure[Note 1]: An oven-dried 25 mL Schlenk-tube was charged with metallic potassium (226 mg, 5.8 mmol, 6.0 equiv.) and **10** (250 mg, 0.97 mmol, 1.0 equiv.) under a continuous nitrogen flow. Subsequently, the tube was slowly heated (over the course of 1 hour) to 100 °C and was incubated at this temperature for 6 hours. After the addition of 2 mL of anhydrous THF the mixture was subjected to TLC analysis that indicated no conversion of the starting material. However, with the addition of solvent the mixture quickly turned to a dark-red coloured suspension, that is the distinct sign of dibenzopentalenide formation. Accordingly, the mixture was allowed to react overnight in 6 mL of dry THF at 65 °C, so that potassium could remain in liquid form (mp.[K^0] = 63.5 °C). After the mixture cooled to room temperature, a solution of iodine (810 mg, 3.2 mmol, 3.3 equiv.) in THF (8 mL, 0.4 molar) was added and stirred for 2 hours.
Based on subsequent NMR analyses, the reaction at elevated temperature resulted in less desirable crude product mixture and so we decided not to further explore these conditions.

[Note 1]: Use a PTFE-coated stir bar should be avoided, when working with molten potassium. Homogeneity was ensured by solvent reflux after addition of THF.

S3.3 Halodesilylation – Primary product valorisation

S3.3.1 Halodesilylation – Iodination

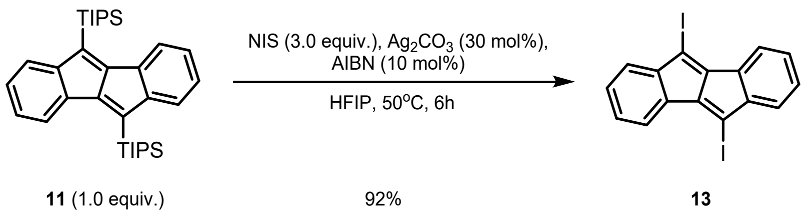

General Procedure D[Note 1] (*chemical initiation*): A 20 mL scintillation vial was charged with **11** (50.0 mg, 0.1 mmol, 1.0 equiv.), *N*-iodosuccinimide (65.5 mg, 0.3 mmol, 3.0 equiv.), AIBN[Note 2] (31.9 mg, 0.2 mmol, 2.0 equiv.) and silver carbonate (8.0 mg, 0.03 mmol, 30 mol%). Under air HFIP (4 mL) was added and the mixture was stirred at 50 °C overnight (6 h). After the reaction completed[Note 3] the mixture was diluted with dichloromethane (40 mL) and washed with Na_2SO_3 (1M, 20 mL) and brine (20 mL). The organic phase was separated, dried over anhydrous $MgSO_4$, filtered and the solvents were removed under reduced pressure. The resulting dark-red tar was further purified by column chromatography (SiO_2, eluting in hexanes) affording the desired product (**13**) as a light-brown solid.

[Note 1]: Reaction monitoring is challenging by TLC analysis as the product and the starting material are hard to separate.
[Note 2]: Notably, dibenzoyl peroxide (Luperox A75) can also be used for radical initiation, resulting in similar yields.
[Note 3]: The reaction when complete affords a "homogeneous", red-brown suspension.

General Procedure E (*photochemical initiation*): A 20 mL scintillation vial was charged with **11** (50.0 mg, 0.1 mmol, 1.0 equiv.), *N*-iodosuccinimide (65.5 mg, 0.3 mmol, 3.0 equiv.) and silver carbonate (8.0 mg, 0.03 mmol, 30 mol%). Under air HFIP (4 mL) was added and the mixture was irradiated (blue LED, $\lambda = 400 \pm 40$ nm) for 4 hours, while vigorously stirred at ambient temperature. After the reaction completed the mixture was diluted with dichloromethane (40 mL) and washed with Na_2SO_3 (1M, 20 mL) and brine (20 mL). The organic phase was separated, dried over anhydrous $MgSO_4$, filtered and the solvents were removed under reduced pressure. The resulting dark-red tar was further purified by column chromatography (SiO_2, eluting in hexanes) affording the desired product (**13**) as a light-brown solid.

Table S4. Optimization of iodinating reagent and conditions.

Entry	Iodinating reagent	Product [%]
1	I_2 (large excess, >10 equiv.)	n.d.
2	ICl (5.0 equiv) [Note 1]	n.d.
3	ICl (2.0 equiv.), TBAF (2.0 equiv.)	n.d.
4	NIS, CsF	n.d.
5	NIS, radical initiation	68-92
6	NIS, TFA:MeCN	complex mixture

[Note 1]: Iodine monochloride was purchased from Sigma-Aldrich and used as received.

Table S5. Radical initiation.[Note 1]

Entry	Initiator	Product [%]
1	no initiator (dark, overnight)	86
2	AIBN (2.0 equiv.)[Note 2]	92
3	AIBN (0.1 equiv.)	81
4	blue LED (λ = 440 nm)	68
5	Luperox A75 (2.0 equiv.)	70
6	Luperox A75 (0.1 equiv.)	79

[Note 1]: 4 hours of reaction time, unless otherwise noted.
[Note 2]: In case of AIBN the mixture was stirred at an elevated temperature of 50 ^{o}C to promote radical formation.

Table S6. Additive, base, fluoride source.

Entry	Additive	Product [%]
1	no additive	59
2	CsF	63
3	Ag_2CO_3 (30 mol%)[Note 1]	92

[Note 1]: 30 mol% Ag_2CO_3 results in cleaner reaction profile.

Table S7. Optimization of solvent. [Note 1]

Entry	Solvents	Product [%]
1	tetrahydrofuran (THF)	n.d.
2	methanol (MeOH)	n.d.
3	diethyl ether (Et_2O)	n.d.
4	dichloromethane (CH_2Cl_2)	n.d.
5	hexafluoroisopropanol (HFIP)[Note 2]	92
6	HFIP / benzene (1:1)	complex mixture

[Note 1]: Intriguingly, the reaction showed remarkable solvent dependency, the desired transformation could only be achieved in HFIP.
[Note 2]: HFIP does not dissolve the substrate completely, the reaction mixture is heterogeneous.

Scale-up of iododesilylation:

Procedure[Note 1]: A 100 mL round-bottom flask was charged with **11** (500.0 mg, 0.97 mmol, 1.0 equiv.), N-iodosuccinimide (655.3 mg, 3.0 mmol, 3.0 equiv.), AIBN (15.9 mg, 0.1 mmol, 0.1 equiv.) and silver-carbonate (83 mg, 0.3 mmol, 30 mol%). Under air HFIP (40 mL) [Note 2] was added and the mixture was stirred at 50 °C overnight (16 h). After the reaction completed the mixture was diluted with dichloromethane (80 mL) and washed with Na_2SO_3 (1M ,80 mL) and brine (80 mL). The organic phase was separated, dried over anhydrous $MgSO_4$, filtered and the solvents were removed under reduced pressure. The resulting dark-red tar was further purified by column chromatography (SiO_2, eluting in hexanes) and recrystallization (The crude product was dissolved in hot hexanes, and methanol was added to induce crystallization. The mixture was then stored at −20 °C) affording the desired product (**13**) as a light-brown solid (320 mg, 72%).

[Note 1]: The procedure was scaled up to 1.75 mmol (900 mg) without further modifications.
[Note 2]: HFIP could be recycled by rotary evaporation and used again for this reaction without loss of efficiency.

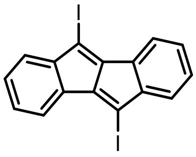

13

Compound 13: product obtained according to General Procedure D/E; as a red-brown solid. **Yield:** 40.6 mg (92%).

TLC: R_f = 0.50 (hexanes) (stains yellow on silica)

^{1}H NMR ($CDCl_3$, 500 MHz): δ = 7.42 (d, J = 7.2 Hz, 2H); 7.07 (td, J = 7.5, 1.2 Hz, 2H); 7.01 (td, J = 7.5, 1.2 Hz, 2H); 6.84 (d, J = 7.30 Hz, 2H) ppm.

^{13}C NMR ($CDCl_3$, 126 MHz): δ = 150.1, 149.8, 132.9, 129.2, 128.6, 124.1, 121.0, 93.1 ppm.

HRMS (ESI-TOF): *m/z* calcd. for $[C_{16}H_8I_2]^+$ ($[M]^+$) 453.8710, found 453.8708.

[Note 1]: In case of lower resolution NMR acquisition triplets at 7.07 and 7.02 ppm tend to form a multiplet.

S3.3.2 Halodesilylation – Bromination

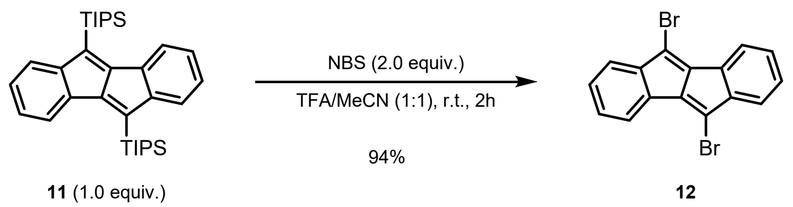

11 (1.0 equiv.) **12**

General Procedure F: A 20 mL scintillation vial was charged with **11** (250.0 mg, 0.49 mmol, 1.0 equiv.) and *N*-bromosuccinimide (172.8 mg, 0.97 mmol, 2.0 equiv.), which were dissolved in a mixture of MeCN (5 mL) and trifluoroacetic acid (TFA, 5 mL). The resulting mixture was stirred for 4 hours at room temperature. After completion the reaction mixture was filtered, and the red-brown precipitate was washed with acetonitrile, dried under reduced pressure to afford title compound **12** (160 mg, 91%).

Table S8. Screened conditions from bromination.

Entry	Conditions	Product [%][Note 1]
1	NBS (3.0 equiv.), AIBN (2.0 equiv.), HFIP, r.t., 16h	**12**; 69%
2	Fe (50 mol%), Br_2 (3.0 equiv.), DCE, r.t., 16h	**12**; 33%[Note 2]
4	NBS (2.2 equiv.), HFIP, dark, r.t., 16h	**12**; 20%
5	NBS (2.0 equiv.), DMF, r.t., 16h	**12**; 10%
6	NBS (2.0 equiv.), mandelic acid (0.4 equiv.), MeCN, r.t., 6h	mixture
7	NBS (2.0 equiv.) S_8 (0.2 equiv.), MeCN, r.t., 6h	mixture
8	NBS (2.0 equiv.), $BF_3{\cdot}Et_2O$ (2.0 equiv.), MeCN, r.t., 6h	**12**; 57%
9	NBS (2.0 equiv.), TFA/MeCN (1:1), r.t., 2h[Note 3]	**12**; 94%
10	NBS (1.0 equiv.), TFA/MeCN (1:1), r.t., 2h	**12**:**10**; 1:1 mixture (71%)

[Note 1]: Title compound was isolated by recrystallization from hexanes / MeOH mixture.
[Note 2]: NMR yield against 1,3,5-trimethoxybenzene internal standard.
[Note 3]: Addition of TFA slightly warms the mixture.

After succeeding with *radical based* iododesilylation, we attempted to perform "orthogonal" – *polar disconnection based* – electrophilic halogenation on the annulated benzene rings. For this purpose, we used Lewis basic conditions to pre-form Br^+ species for subsequent S_EAr bromination. The screened conditions exclusively resulted in desilylative halogenations in *5,10* positions with varying yields and efficiency.
Notably, conditions described in *Entry 9*, Table S8 showed high efficiency combined with ease of operation, which allowed fast and reasonably high material throughput.

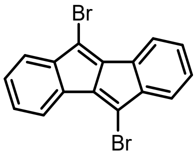

12

Compound 12: product obtained according to General Procedure F; as a red-brown solid. **Yield:** 160.0 mg (94%).

TLC: $R_f = 0.63$ (hexanes)

^{1}H NMR ($CDCl_3$, 500 MHz): $\delta = 7.35$ (dd, $J = 6.6$, 1.0 Hz, 2H); 7.07-7.00 (m, 4H); 6.97 (dd, $J = 7.0$, 1.0 Hz, 2H) ppm.

^{13}C NMR (CDCl$_3$, 126 MHz): δ = 147.6, 144.4, 133.0, 128.9, 128.8, 122.25, 122.23, 120.5 ppm.

HRMS (APCI-TOF): *m/z* calcd. for $[C_{16}H_8Br_2]^+$ ($[M]^+$) 357.8987, found 357.8989.

S3.4 Functionalization of dihalo-DBPs - Secondary product valorisation

Structural diversity accessible from the dihalogenated intermediates (**12** and **13**) was demonstrated via the preparation of a scope of *5,10*-disubstituted dibenzo[*a,e*]pentalenes. Synthetic procedures and product characterization are detailed below:

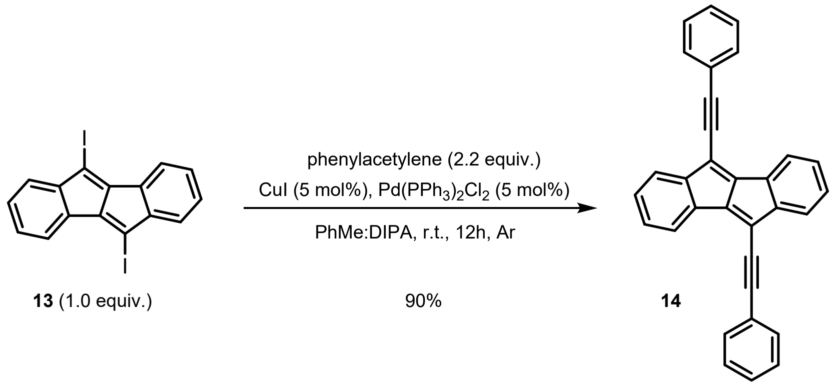

Procedure: A 4 mL scintillation vial was charged with **13** (50.0 mg, 0.11 mmol, 1.0 equiv.), copper(I) iodide (1.0 mg, 5.5 µmol, 5 mol%), tetrakis(triphenylphosphine)palladium(0) (6.3 mg, 5.5 µmol, 5 mol%). The vial was purged with argon, and the degassed solvent mixture (toluene : DIPA, V/V = 1 : 1, 2 mL) was added via syringe. Subsequently, phenylacetylene (24.6 mg, 0.24 mmol, 2.2 equiv.) was added and the resulting mixture was stirred at room temperature overnight. Once complete, the mixture was diluted with DCM, filtered over a pad of celite and concentrated under reduced pressure. The crude residue was further purified via column chromatography (SiO_2, hexanes) affording the desired product as a dark greenish solid.

Compound 14: product obtained as a dark green solid. **Yield:** 40.0 mg (90%).

TLC: R_f = 0.17 (hexanes) (stains orange)

^{1}H NMR (CDCl$_3$, 500 MHz): δ = 7.62 (m, 4H); 7.41 (m, 6H); 7.38 (m, 2H); 7.14 (m, 2H); 7.01 (m, 4H) ppm.

^{13}C NMR (CDCl$_3$, 126 MHz): δ = 149.3, 148.6, 133.5, 132.2, 129.4, 128.8, 128.7, 128.2, 123.0, 122.8, 122.2, 120.4, 103.3, 84.4 ppm.

HRMS (APCI-TOF): *m/z* calcd. for $[C_{32}H_{19}]^+$ ($[M+H]^+$) 403.1481, found 403.1487.

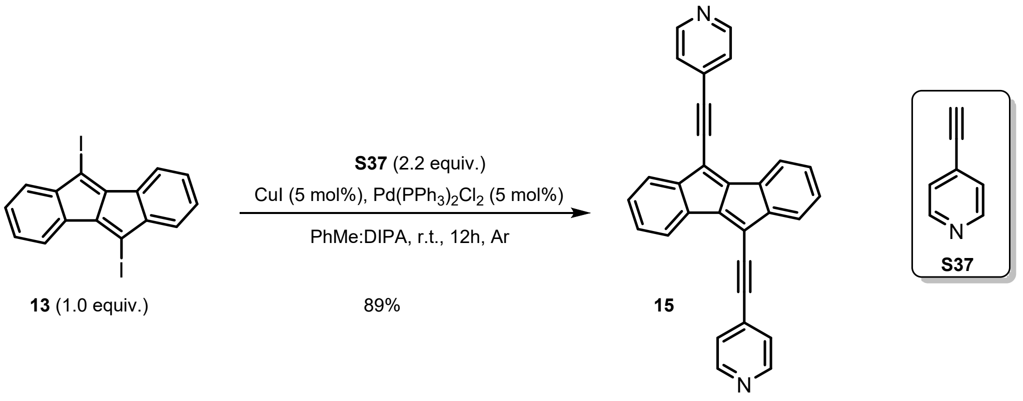

Procedure: A 4 mL scintillation vial was charged with **13** (50.0 mg, 0.11 mmol, 1.0 equiv.), copper(I) iodide (1.0 mg, 5.5 μmol, 5 mol%), tetrakis(triphenylphosphine)palladium(0) (6.3 mg, 5.5 μmol, 5 mol%), and 4-ethynylpyridine (**S37**) (24.9 mg, 0.24 mmol, 2.2 equiv.). The vial was purged with argon, and the degassed solvent mixture (toluene / DIPA, V/V = 1 : 1, 2 mL) was added via syringe. The resulting mixture was stirred at room temperature overnight. Once complete, the mixture was diluted with DCM, filtered over a pad of celite and concentrated under reduced pressure. The crude residue was further purified via column chromatography (SiO_2, hexane/ethyl acetate) affording the desired product as a dark red solid.

Compound 15: product obtained as a dark red solid. **Yield:** 39.7 mg (89%).

TLC: R_f = 0.50 (EtOAc, 1% TEA V/V)

^{1}H NMR ($CDCl_3$, 500 MHz): δ = 8.68 (d, J = 6.0 Hz, 4H); 7.45 (d, J = 6.0 Hz, 4H); 7.32 (d, J = 6.1 Hz, 2H); 7.10 (d, J = 6.5 Hz, 2H); 7.03 (m, 4H) ppm.

^{13}C NMR ($CDCl_3$, 126 MHz): δ = 150.9, 150.2, 148.3, 132.9, 130.7, 129.5, 128.6, 125.7, 123.2, 122.4, 120.0, 99.8, 87.8 ppm.

HRMS (APCI-TOF): *m/z* calcd. for $[C_{30}H_{17}N_2]^+$ ($[M+H]^+$) 405.1386, found 405.1393.

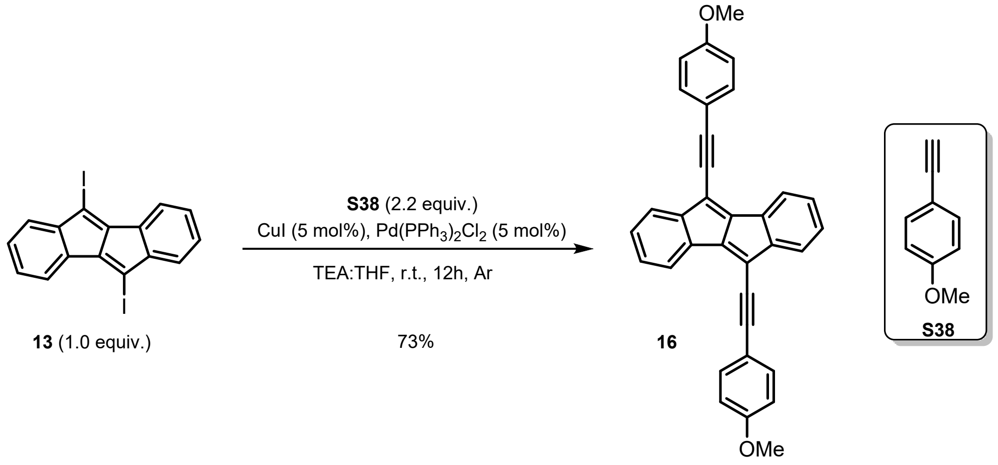

Procedure: A 4 mL scintillation vial was charged with **13** (50.0 mg, 0.11 mmol, 1.0 equiv.), copper(I) iodide (1.0 mg, 5.5 μmol, 5 mol%), bis(triphenylphosphine)palladium dichloride (3.8 mg, 5.5 μmol, 5 mol%). The vial was purged with argon, and the degassed solvent mixture

(TEA / THF, V/V = 1 : 1, 2 mL) was added via syringe. Subsequently, 4-ethynylanisole (**S38)** (31.9 mg, 0.24 mmol, 2.2 equiv.) was added and the resulting mixture was stirred at room temperature overnight. Once complete, the mixture was diluted with DCM, filtered over a pad of celite and concentrated under reduced pressure. The crude residue was further purified via column chromatography (SiO_2, hexane/ethyl acetate) affording the desired product as a dark red solid.

Compound 16: product obtained as a dark red solid. **Yield:** 37.0 mg (73%).

TLC: R_f = 0.42 (hexanes / EtOAc, 9 : 1 V/V)

^{1}H NMR ($CDCl_3$, 500 MHz): δ = 7.55 (d, J = 8.6 Hz, 4H); 7.37 (m, 2H); 7.14 (m, 2H); 7.00 (m, 4H); 6.93 (d, J = 8.4 Hz, 4H) ppm.

^{13}C NMR ($CDCl_3$, 126 MHz): δ = 160.7, 148.6, 148.5, 133.9, 133.7, 128.5, 128.0, 122.6, 122.1, 120.4, 115.2, 114.4, 103.7, 83.8, 55.6 ppm.

HRMS (APCI-TOF): *m/z* calcd. for $[C_{34}H_{23}O_2]^+$ ($[M+H]^+$) 463.1692, found 463.1694.

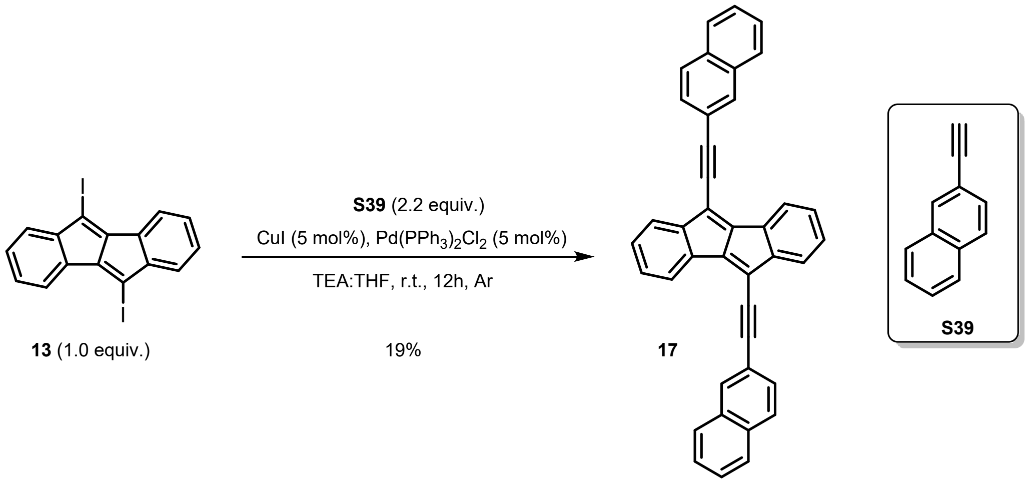

Procedure: A 4 mL scintillation vial was charged with **13** (50.0 mg, 0.11 mmol, 1.0 equiv.), copper(I) iodide (1.0 mg, 5.5 µmol, 5 mol%), bis(triphenylphosphine)palladium dichloride (3.8 mg, 5.5 µmol, 5 mol%), 2-ethynylnaphthalene (**S39**) (50.0 mg, 0.33 mmol, 3.0 equiv.). The vial was purged with argon, and a degassed solvent mixture (TEA / THF, V/V = 1 : 1, 2 mL) was added via a syringe and the resulting mixture was stirred at room temperature overnight. Once complete, the mixture was diluted with DCM, filtered over a pad of celite and concentrated under reduced pressure. The crude residue was further purified via column chromatography (SiO_2, hexane/ethyl acetate) affording the desired product as a dark red solid.

Compound 17: product obtained as a dark red solid. **Yield:** 10.3 mg (19%).

TLC: R_f = 0.49 (hexanes / EtOAc, 9 : 1 V/V)

^{1}H NMR (CD_2Cl_2, 500 MHz): δ = 8.19 (s, 2H); 7.93-7.87 (m, 4H); 7.68 (d, J = 8.3 Hz, 2H); 7.59-7.55 (m, 4H); 7.51-7.47 (m, 2H); 7.26-7.23 (m, 2H); 7.11-7.06 (m, 4H) ppm.

^{13}C NMR (CDCl$_3$, 126 MHz): δ = 149.4, 148.6, 133.5, 133.4, 133.2, 132.5, 128.8, 128.6, 128.5, 128.2, 128.1, 127.4, 127.0, 122.8, 122.3, 120.4, 120.2, 103.9, 84.9 ppm.
(Due to low solubility ^{13}C signals are weak.)

HRMS (APCI-TOF): *m/z* calcd. for $[C_{40}H_{23}]^+$ ($[M+H]^+$) 503.1794, found 503.1798.

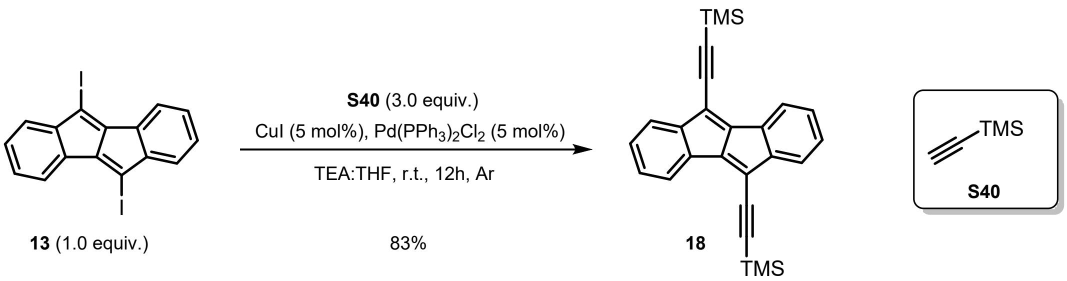

13 (1.0 equiv.) 83% **18**

Procedure: A 4 mL scintillation vial was charged with **13** (50.0 mg, 0.11 mmol, 1.0 equiv.), copper(I) iodide (1.0 mg, 5.5 μmol, 5 mol%), bis(triphenylphosphine)palladium dichloride (3.8 mg, 5.5 μmol, 5 mol%). The vial was purged with argon, and the degassed solvent mixture (TEA / THF, V/V = 1 : 1, 2 mL) was added via syringe. Subsequently, (trimethylsilyl)acetylene (**S40**) (32.3 mg, 0.33 mmol, 3.0 equiv.) was added and the resulting mixture was stirred at room temperature overnight. Once complete, the mixture was diluted with DCM, filtered over a pad of celite and concentrated under reduced pressure. The crude residue was further purified via column chromatography (SiO_2, hexanes) affording desired product as a dark red solid.

Compound 18: product obtained as a dark red solid. **Yield:** 36 mg (83%).

TLC: R_f = 0.36 (hexanes)

^{1}H NMR (CDCl$_3$, 500 MHz): δ = 7.24-7.21 (m, 2H); 7.02-6.93 (m, 6H); 0.31 (s, 18H) ppm.

^{13}C NMR (CDCl$_3$, 126 MHz): δ = 150.1, 148.5, 133.1, 128.9, 128.2, 122.8, 122.1, 120.4, 110.1, 0.1 ppm.

HRMS (APCI-TOF): *m/z* calcd. for $[C_{26}H_{26}Si_2]^+$ ($[M]^+$) 394.1567, found 394.1575.

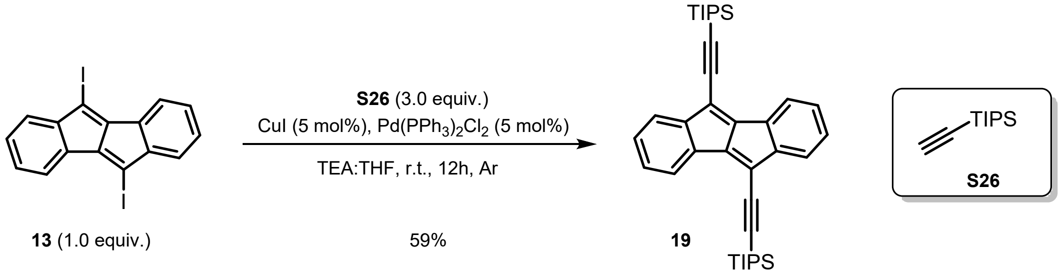

13 (1.0 equiv.) 59% **19**

Procedure: A 4 mL scintillation vial was charged with **13** (50.0 mg, 0.11 mmol, 1.0 equiv.), copper(I) iodide (1.0 mg, 5.5 μmol, 5 mol%), bis(triphenylphosphine)palladium dichloride (3.8 mg, 5.5 μmol, 5 mol%). The vial was purged with argon, and the degassed solvent mixture (TEA / THF, V/V = 1 : 1, 2 mL) was added via syringe. Subsequently, (triisopropylsilyl)acetylene (**S26**) (59.9 mg, 0.33 mmol, 3.0 equiv.) was added and the resulting

mixture was stirred at room temperature overnight. Once complete, the mixture was diluted with DCM, filtered over a pad of celite and concentrated under reduced pressure. The crude residue was further purified via column chromatography (SiO_2, hexanes) affording the desired product as a dark red solid.

Compound 19: product obtained as a dark red solid. **Yield:** 36.9 mg (59%).

TLC: $R_f = 0.65$ (hexanes)

^{1}H NMR (CD_2Cl_2, 500 MHz): δ = 7.28 (d, J = 6.4 Hz, 2H); 7.04 (m, 2H); 7.01-6.96 (m, 4H); 1.19 (s, 42H) ppm.

^{13}C NMR (CD_2Cl_2, 126 MHz): δ = 149.9, 149.0, 133.4, 129.3, 128.6, 123.0, 122.4, 120.9, 107.8, 101.0, 18.9, 11.8 ppm.

HRMS (APCI-TOF): *m/z* calcd. for $[C_{38}H_{51}Si_2]^+$ ($[M+H]^+$) 563.3523, found 563.3527.

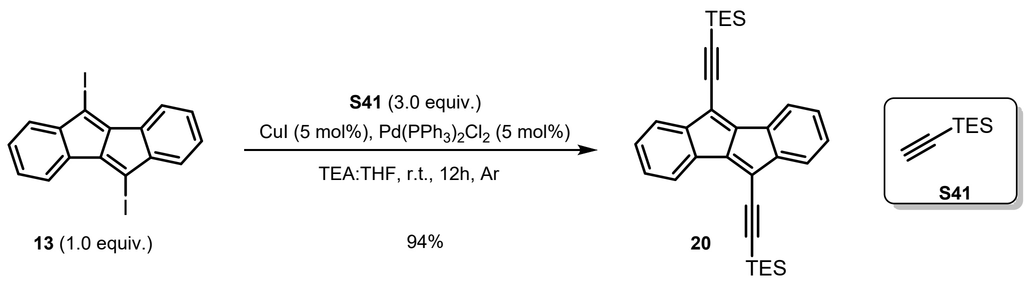

Procedure: A 4 mL scintillation vial was charged with **13** (50.0 mg, 0.11 mmol, 1.0 equiv.), copper(I) iodide (1.0 mg, 5.5 µmol, 5 mol%), bis(triphenylphosphine)palladium dichloride (3.8 mg, 5.5 µmol, 5 mol%). The vial was purged with argon, and the degassed solvent mixture (TEA / THF, V/V = 1 : 1, 2 mL) was added via syringe. Subsequently, (triethylsilyl)acetylene (**S41**) (46.1 mg, 0.33 mmol, 3.0 equiv.) was added and the resulting mixture was stirred at room temperature overnight. Once complete, the mixture was diluted with DCM, filtered over a pad of celite and concentrated under reduced pressure. The crude residue was further purified via column chromatography (SiO_2, hexanes) affording the desired product as a red-brown solid.

Compound 20: product obtained as a red-brown solid. **Yield:** 49.7 mg (94%).

TLC: $R_f = 0.46$ (hexanes)

^{1}H NMR ($CDCl_3$, 500 MHz): δ = 7.25 (m, 2H); 7.01-6.98 (m, 2H); 6.98-6.93 (m, 4H); 1.09 (t, J = 7.90 Hz, 16H); 0.74 (q, J = 7.90 Hz, 12H) ppm.

^{13}C NMR ($CDCl_3$, 126 MHz): δ = 149.9, 148.7, 133.2, 128.9, 128.2, 122.8, 122.1, 120.5, 107.9, 100.1, 7.71, 4.56 ppm.

HRMS (APCI-TOF): *m/z* calcd. for $[C_{32}H_{38}Si_2]^+$ ($[M]^+$) 478.2506, found 478.2509.

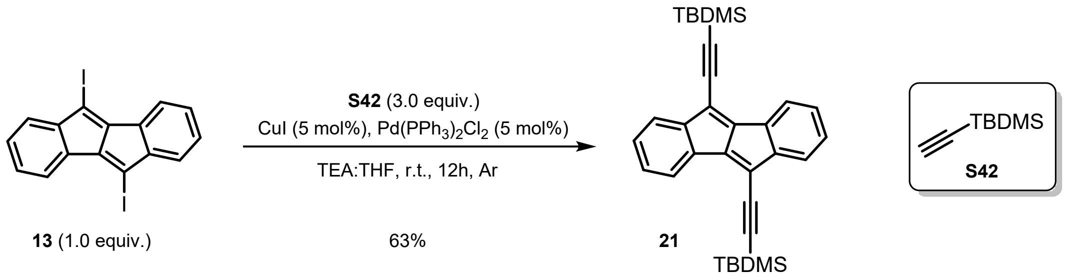

Procedure: A 4 mL scintillation vial was charged with **13** (50.0 mg, 0.11 mmol, 1.0 equiv.), copper(I) iodide (1.0 mg, 5.5 μmol, 5 mol%), bis(triphenylphosphine)palladium dichloride (3.8 mg, 5.5 μmol, 5 mol%). The vial was purged with argon, and the degassed solvent mixture (TEA / THF, V/V = 1 : 1, 2 mL) was added via syringe. Subsequently, (tert-butyldimethylsilyl)acetylene (**S42**) (46.1 mg, 0.33 mmol, 3.0 equiv.) was added and the resulting mixture was stirred at room temperature overnight. Once complete, the mixture was diluted with DCM, filtered over a pad of celite and concentrated under reduced pressure. The crude residue was further purified via column chromatography (SiO_2, hexanes) affording the desired product as a dark red solid.

Compound 21: product obtained as a dark red solid. **Yield:** 33.2 mg (63%).

TLC: $R_f = 0.44$ (hexanes)

^{1}H NMR ($CDCl_3$, 500 MHz): δ = 7.24 (m, 2H); 7.02-6.93 (m, 6H); 1.04 (s, 18H); 0.25 (s, 12H) ppm.

^{13}C NMR ($CDCl_3$, 126 MHz): δ = 150.0, 148.6, 133.2, 128.9, 128.2, 122.8, 122.1, 120.4, 108.7, 99.5, 26.4, 16.8, -4.4 ppm.

HRMS (APCI-TOF): *m/z* calcd. for $[C_{32}H_{39}Si_2]^+$ ($[M+H]^+$) 479.2584, found 479.2577.

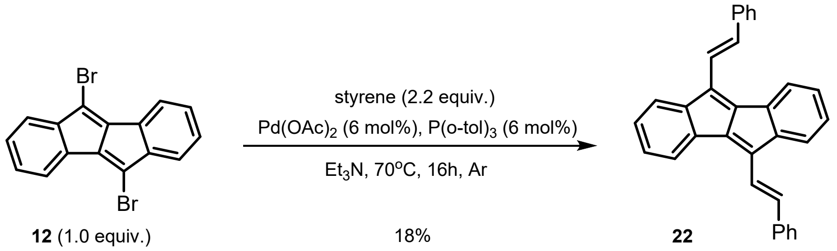

Procedure: A 4 mL scintillation vial was charged with **12** (50.0 mg, 0.14 mmol, 1.0 equiv.), palladium acetate (1.0 mg, 4.2 μmol, 6 mol%), tris(o-tolyl)phosphine (2.5 mg, 8.3 μmol, 5 mol%). The vial was purged with argon, and styrene (31.9 mg, 0.3 mmol, 2.2 equiv.; 99%, stabilized by 10-15 ppm 4-*tert*-butyl-catechol) was added in triethylamine (TEA, 1 mL) via syringe, then the resulting mixture was stirred at 70°C overnight. Once complete, the mixture was diluted with DCM, filtered over a pad of celite and concentrated under reduced pressure. The crude residue was further purified via column chromatography (SiO_2, hexanes/ethyl acetate) affording the desired product as a dark amorphous solid.

Compound 22: product obtained as a dark solid. **Yield:** 10.2 mg (18%).

TLC: R_f = 0.60 (hexanes / EtOAc, 9 : 1 V/V)

^{1}H NMR ($CDCl_3$, 500 MHz): δ = 7.59 (d, J = 7.58 Hz, 4H); 7.44-7.32 (m, 12H); 7.01 (m, 4H) ppm.

^{13}C NMR ($CDCl_3$, 126 MHz): δ = 148.4, 144.4 137.4, 136.6 135.8, 135.1, 129.1, 128.8, 127.6, 127.5, 127.1, 123.0, 122.8, 121.4 ppm.

HRMS (APCI-TOF): *m/z* calcd. for $[C_{32}H_{23}]^+$ ($[M+H]^+$) 407.1794, found 407.1784.

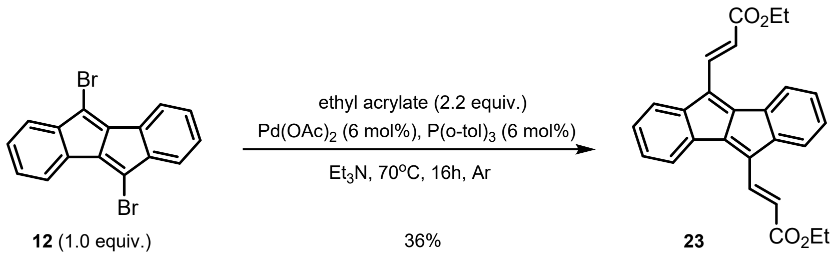

12 (1.0 equiv.) 36% **23**

Procedure: A 4 mL scintillation vial was charged with **12** (50.0 mg, 0.14 mmol, 1.0 equiv.), palladium acetate (1.0 mg, 4.2 µmol, 6 mol%), tris(o-tolyl)phosphine (2.5 mg, 8.3 µmol, 5 mol%). The vial was purged with argon, and ethyl acrylate (30.6 mg, 0.3 mmol, 2.2 equiv.) was added in triethylamine (TEA, 1 mL) via syringe, then the resulting mixture was stirred at 70°C overnight. Once complete, the mixture was diluted with DCM, filtered over a pad of celite and concentrated under reduced pressure. The crude residue was further purified via column chromatography (SiO_2, hexane/ethyl acetate) affording the desired product as a dark red/black solid.

Compound 23: product obtained as a dark solid. **Yield:** 20.0 mg (36%).

TLC: R_f = 0.42 (hexanes / EtOAc, 9 : 1 V/V)

^{1}H NMR ($CDCl_3$, 500 MHz): δ = 7.78 (d, J = 16.2 Hz, 2H); 7.31 (m, 2H); 7.21 (m, 2H); 6.99 (m, 4H); 6.60 (d, J = 16.2 Hz, 2H); 4.31 (q, J = 7.2 Hz, 4H); 1.37 (t, J = 7.1 Hz, 6H) ppm.

^{13}C NMR ($CDCl_3$, 126 MHz): δ = 166.8, 149.0, 147.9, 135.5, 135.2, 134.4, 129.1, 128.2, 124.0, 123.3, 61.1, 14.5 ppm.

HRMS (APCI-TOF): *m/z* calcd. for $[C_{26}H_{22}O_4]^+$ ($[M]^+$) 398.1512, found 398.1516.

Table S9. Screening of reaction conditions to access compound **23**.

Entry	Conditions	Product [%]
1	$Pd(OAc)_2$ (5 mol%), $P(o\text{-}tol)_3$ (10 mol%), EtOAc (5 equiv.), TEA:PhMe (1:1), 90°C	<5%
2	$Pd(OAc)_2$ (6 mol%), $P(o\text{-}tol)_3$ (12 mol%), EtOAc (2.2 equiv.), TEA, 70°C	36%

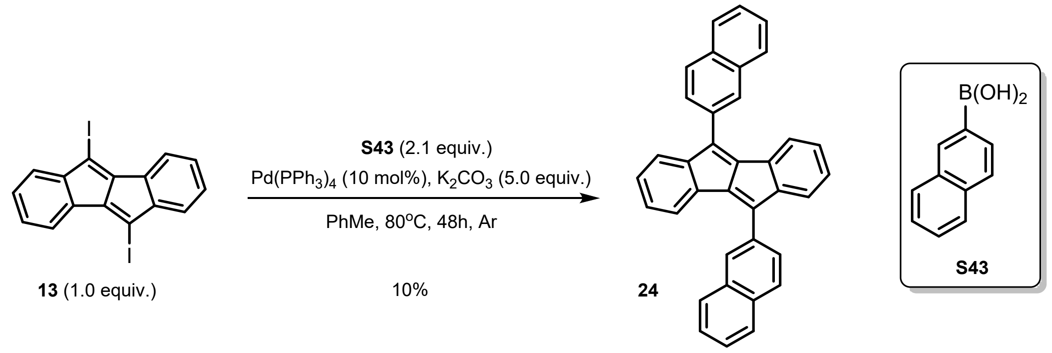

Procedure: A 20 mL scintillation vial was charged with **13** (100.0 mg, 0.22 mmol, 1.0 equiv.), naphtanlen-2-ylboronic acid (**S43**) (79.6 mg, 0.46 mmol, 2.1 equiv.), potassium carbonate (152.0 mg, 1.10 mmol, 5.0 equiv.), tetrakis(triphenylphosphine)palladium(0) (25.5 mg, 22 µmol, 10 mol%) and was purged with argon. Toluene (4 mL) was added using a syringe and the reaction mixture was stirred at 80°C for 48 hours. After completion, the mixture was diluted with DCM, filtered over a pad of celite and concentrated under reduced pressure. The crude residue was further purified via column chromatography (SiO_2, hexane/ethyl acetate) affording the desired product as an orange amorphous solid.

Compound 24: product obtained as an orange solid. **Yield:** 9.8 mg (10%).

TLC: R_f = 0.59 (hexanes / EtOAc, 9 : 1 V/V)

^{1}H NMR ($CDCl_3$, 500 MHz): δ = 8.20 (s, 2H); 8.03 (d, J = 8.43 Hz, 2H); 7.96 (m, 4H); 7.82 (d, J = 8.38 Hz, 2H); 7.59 (m, 4H); 7.27 (d, J = 7.40 Hz, 2H); 7.15 (d, J = 7.40 Hz, 2H); 6.96 (t, J = 7.46 Hz, 2H); 6.88 (t, J = 7.46 Hz, 2H) ppm.

^{13}C NMR ($CDCl_3$, 126 MHz): δ = 149.9, 143.7, 140.9, 135.4, 133.6, 133.5, 131.7, 128.5, 128.4, 128.04, 128.97, 127.7, 126.8, 126.7, 126.5, 122.8, 122.2 ppm. Due to low solubility the carbon

HRMS (APCI-TOF): *m/z* calcd. for $[C_{36}H_{23}]^+$ ($[M+H]^+$) 455.1794, found 455.1798.

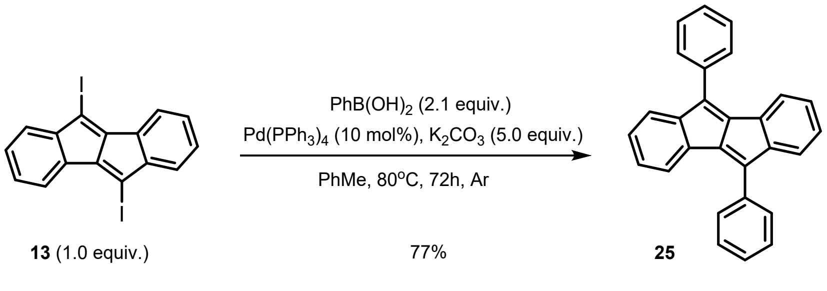

Procedure: A 20 mL scintillation vial was charged with **13** (50.0 mg, 0.11 mmol, 1.0 equiv.), phenylboronic acid (28.2 mg, 0.23 mmol, 2.1 equiv.), potassium carbonate (76.0 mg, 0.55 mmol, 5.0 equiv.), tetrakis(triphenylphosphine)palladium(0) (12.7 mg, 11 µmol, 10 mol%) and was purged with argon. Toluene (2 mL) was added using a syringe and the reaction mixture was stirred at 80°C for 72 hours. After completion, the mixture was diluted with DCM, filtered over a pad of celite and concentrated under reduced pressure. The crude residue was further

purified via column chromatography (SiO_2, hexane/ethyl acetate) affording the desired product as an orange amorphous solid.

Compound 25: product obtained as an orange solid. **Yield:** 30.0 mg (77%).

TLC: R_f = 0.23 (hexanes)

^{1}H NMR (CDCl$_3$, 500 MHz): δ = 7.67 (d, J = 6.98 Hz, 4H); 7.52 (t, J = 7.68 Hz, 4H); 7.45 (t, J = 7.41 Hz, 2H); 7.21 (d, J = 7.26 Hz, 2H); 7.02 (d, J = 7.35 Hz, 2H); 6.90 (t, J = 7.52 Hz, 2H); 6.84 (t, J = 7.44 Hz, 2H) ppm.

^{13}C NMR (CDCl$_3$, 126 MHz): δ = 150.0, 143.5, 141.1, 135.6, 134.2, 129.3, 129.1, 128.9, 128.3, 127.9, 122.9, 122.3 ppm.

HRMS (APCI-TOF): *m/z* calcd. for $[C_{28}H_{19}]^+$ ($[M+H]^+$) 355.1481, found 355.1472.

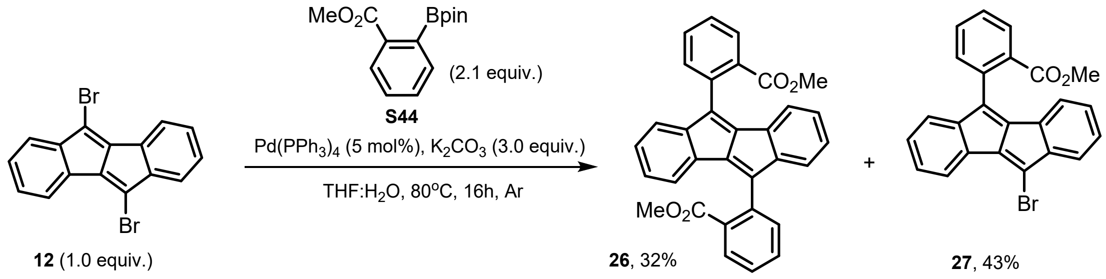

Procedure: A 20 mL scintillation vial was charged with **12** (50.0 mg, 0.14 mmol, 1.0 equiv.), boronate **S44** (28.2 mg, 0.23 mmol, 2.1 equiv.), potassium carbonate (57.6 mg, 0.42 mmol, 3.0 equiv.), tetrakis(triphenylphosphine)palladium(0) (8.0 mg, 7.0 µmol, 5 mol%) and was purged with argon. A mixture of THF / water (5 mL : 0.1 mL) was added using a syringe and the reaction mixture was stirred at 80°C overnight. After completion, the mixture was diluted with DCM, filtered over a pad of celite and concentrated under reduced pressure. The crude residue was further purified via column chromatography (SiO_2, hexane/ethyl acetate) affording the desired product as an orange amorphous solid.
Notably, the monosubstituted product (**27**) could be isolated as a major side product of the reaction.

Compound 26: product obtained as an orange amorphous solid. **Yield:** 21.3 mg (32%).

TLC: R_f = 0.34 (hexanes / EtOAc, 9 : 1 V/V)

^{1}H NMR (CDCl$_3$, 500 MHz): δ = 8.07 (d, J = 7.9 Hz, 2H); 7.71-7.64 (m, 4H); 7.61 (d, J = 7.7 Hz, 1H); 7.56 (t, J = 7.8 Hz, 2H); 7.38-7.32 (m, 1H); 7.30-7.24 (m, 1H); 6.86-6.74 (m, 3H), 6.57 (t, J = 6.7 Hz, 1H); 3.70 (s, 3H); 3.67 (s, 3H) ppm.

^{13}C NMR (CDCl$_3$, 126 MHz): δ = 167.9, 167.8, 161.9, 151.1, 132.8, 132.7, 131.12, 131.08, 130.9, 130.5, 129.08, 129.05, 128.30, 128.26, 127.4, 122.24, 122.20, 121.8, 52.6, 52.5 ppm.

HRMS (APCI-TOF): *m/z* calcd. for $[C_{32}H_{23}O_4]^+$ ($[M+H]^+$) 471.1590, found 471.1580.

Compound 27: product obtained as a red amorphous solid. **Yield:** 28.0 mg (43%).

TLC: R_f = 0.64 (hexanes / EtOAc, 9 : 1 v/v)

^{1}H NMR (CD_2Cl_2, 500 MHz): δ = 8.06 (dd, J = 8.1, 1.4 Hz, 1H); 7.66 (td, J = 7.6, 1.4 Hz, 1H); 7.56 (m, 2H); 7.45 (d, J = 7.3 Hz, 1H); 6.99 (m, 3H); 6.93 (td, J = 7.5, 1.2 Hz, 1H); 6.86-6.81 (m, 1H); 6.74 (d, J = 7.3 Hz, 1H); 6.58 (d, J = 7.3 Hz, 1H); 3.66 (s, 3H) ppm.

^{13}C NMR (CD_2Cl_2, 126 MHz): δ = 167.6, 150.6, 148.4, 145.9, 142.3, 141.4, 134.4, 134.0, 133.8, 132.8, 131.2, 131.1, 130.5, 129.3, 129.0, 128.7, 128.6, 128.0, 122.9, 122.20, 122.17, 121.9, 119.9, 52.6 ppm.

HRMS (APCI-TOF): *m/z* calcd. for $[C_{24}H_{16}O_2Br]^+$ ($[M]^+$) 415.0328, found 415.0318.

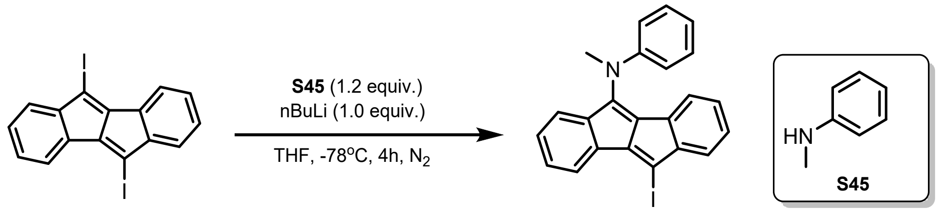

13 (1.0 equiv.) 69% **28**

Procedure: A 30 mL scintillation vial was charged with *N*-methylaniline (**S45**) (39.0 mg, 0.36 mmol, 1.1 equiv.) dissolved in anhydrous THF (1.5 mL) and was purged with nitrogen gas. The solution was cooled to -78 °C and *n*-butyllithium (21.0 mg, 0.33 mmol, 1.6 M in hexanes, 206 µL, 1.0 equiv.) was added dropwise and the resulting solution was stirred at this temperature for 30 minutes. Subsequently, a solution of **13** (150.0 mg, 0.33 mmol, 1.0 equiv.) in anhydrous THF (6 mL) was added and the mixture was allowed to warm to room temperature over the course of 3 hours. After completion, the reaction mixture was quenched with water and was extracted with DCM. Phases were separated and the combined organic phase was washed with brine, dried over anhydrous $MgSO_4$ and concentrated under reduced pressure. The crude residue was further purified via column chromatography (SiO2, hexanes/ethyl acetate) affording the desired product as a dark purple-black amorphous solid.

Compound 28: product obtained as a black amorphous solid. **Yield:** 99.0 mg (69%).

TLC: R_f = 0.71 (hexanes / EtOAc, 9 : 1 V/V)

^{1}H NMR ($CDCl_3$, 300 MHz): δ = 7.65 (d, J = 7.4 Hz, 1H); 7.41-7.32 (m, 2H); 7.25-7.13 (m, 3H); 7.03-6.94 (m, 3H); 6.87 (ddd, J = 7.5, 6.8, 1.9 Hz, 1H); 6.69 (d, J = 7.6 Hz, 1H); 6.27 (d, J = 7.6 Hz, 1H); 3.74 (s, 3H) ppm.

^{13}C NMR ($CDCl_3$, 75 MHz): δ = 150.0, 148.4, 147.2, 145.8, 145.0, 136.4, 132.6, 129.5, 128.1, 127.5, 126.6, 126.5, 125.5, 124.4, 124.0, 123.2, 123.0, 120.3, 82.0, 43.0 ppm.

HRMS (ESI-TOF): *m/z* calcd. for $[C_{23}H_{16}NI]^+$ ($[M]^+$) 433.0327, found 433.0313.

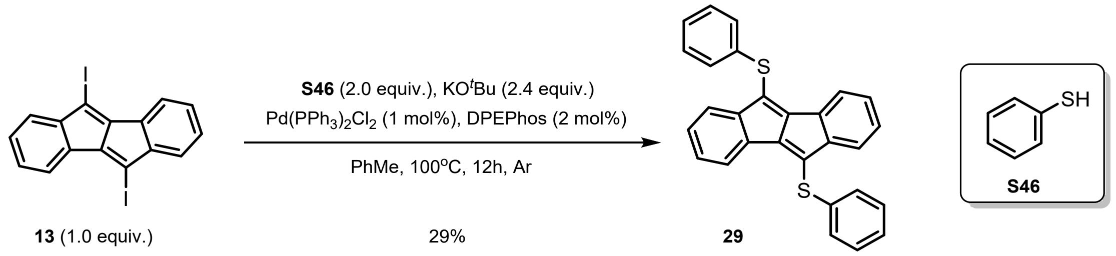

Procedure: Under an argon atmosphere tris(dibenzylideneacetone)dipalladium(0) (1.0 mg, 1.1 μmol, 1 mol%) and DPEPhos (1.2 mg, 2.2 μmol, 2 mol%) was dissolved in anhydrous toluene (1 mL) and stirred for 10 minutes at ambient temperature. To the resulting solution were added **13** (50.0 mg, 0.11 mmol, 1.0 equiv.), thiophenol (**S46**) (24.3 mg, 0.22 mmol, 2.0 equiv.), and potassium *tert*-butoxide (14.8 mg, 0.13 mmol, 1.2 equiv.) and the reaction was heated at 110°C overnight. After cooling, the crude mixture was diluted with DCM, filtered over a pad of celite and washed with NaOH solution (1M). The organic layers were separated, dried over anhydrous $MgSO_4$ and concentrated under reduced pressure. The crude residue was further purified via column chromatography (SiO_2, hexane/ethyl acetate) affording the desired product as a bright orange amorphous solid.

Compound 29: product obtained as an orange solid. **Yield:** 13.0 mg (29%).

TLC: R_f = 0.59 (hexanes / EtOAc, 9 : 1 V/V)

^{1}H NMR (CDCl$_3$, 500 MHz): δ = 7.50 (d, J = 7.1 Hz, 4H); 7.33-7.30 (m, 4H); 7.28-7.24 (m, 1H); 6.83-6.76 (m, 8H); 6.69-6.64 (m, 2H) ppm.

^{13}C NMR (CDCl$_3$, 126 MHz): δ = 148.3, 146.5, 134.0, 133.4, 132.7, 130.7, 129.5 128.0, 127.7, 127.4, 123.4, 122.4 ppm.

HRMS (APCI-TOF): *m/z* calcd. for $[C_{28}H_{19}S_2]^+$ ($[M+H]^+$) 419.0922, found 419.0917.

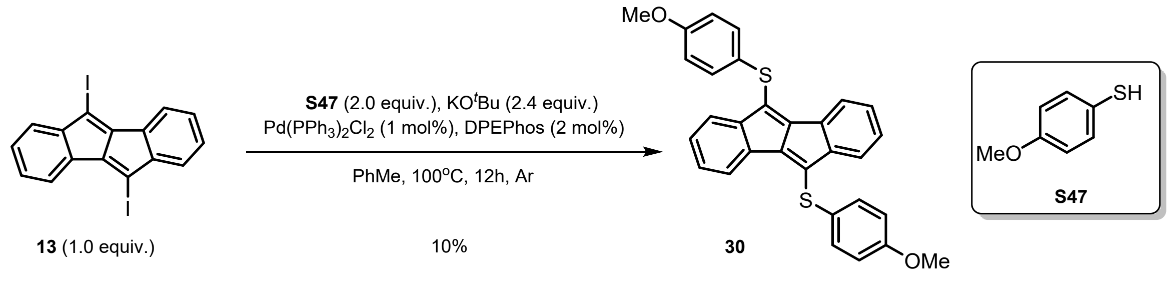

Procedure: Under an argon atmosphere tris(dibenzylideneacetone)dipalladium(0) (1.0 mg, 1.1 μmol, 1 mol%) and DPEPhos (1.2 mg, 2.2 μmol, 2 mol%) was dissolved in anhydrous toluene (1 mL) and stirred for 10 minutes at ambient temperature. To the resulting solution were added **13** (50.0 mg, 0.11 mmol, 1.0 equiv.), 4-methoxybenzenethiol (**S47**) (30.9 mg, 0.22 mmol, 2.0 equiv.), and potassium *tert*-butoxide (29.7 mg, 0.26 mmol, 2.4 equiv.) and the reaction was heated at 110 °C overnight. After cooling, the crude mixture was diluted with DCM, filtered over a pad of celite and washed with NaOH solution (1M). The organic layers were separated, dried over anhydrous $MgSO_4$ and concentrated under reduced pressure. The crude residue was

further purified via column chromatography (SiO_2, hexane/ethyl acetate) affording the desired product as an orange amorphous solid.

Compound 30: product obtained as an orange solid. **Yield:** 5.2 mg (10%).

TLC: R_f = 0.31 (hexanes / EtOAc, 7 : 1 V/V)

^{1}H NMR ($CDCl_3$, 500 MHz): δ = 7.46 (d, J = 8.8 Hz, 4H); 6.87 (d, J = 8.7 Hz, 4H); 6.80-6.74 (m, 6H); 6.64 (m, 2H); 3.81 (s, 6H) ppm.

^{13}C NMR ($CDCl_3$, 126 MHz): δ = 159.7, 148.3, 145.0, 134.2, 133.7, 133.3, 127.6, 127.5, 123.4, 123.1, 122.3, 115.2, 55.6 ppm.

HRMS (APCI-TOF): *m/z* calcd. for $[C_{30}H_{23}O_2S_2]^+$ ($[M+H]^+$) 479.1134, found 479.1126.

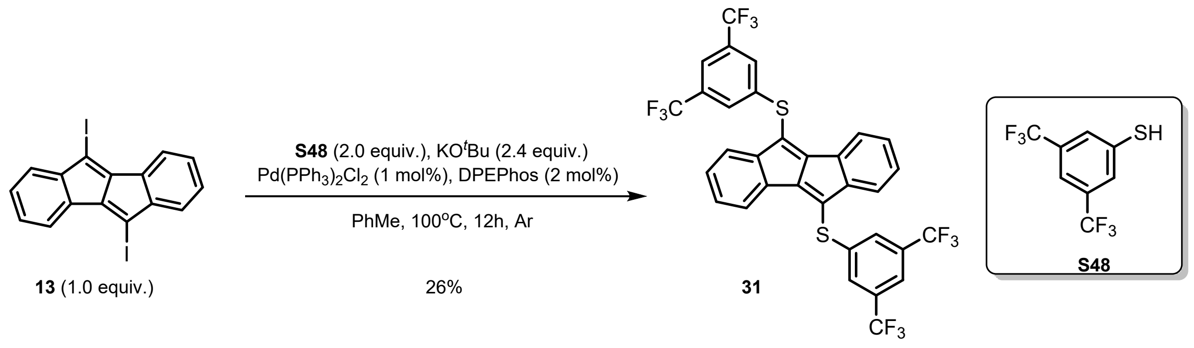

Procedure: Under an argon atmosphere tris(dibenzylideneacetone)dipalladium(0) (1.0 mg, 1.1 μmol, 1 mol%) and DPEPhos (1.2 mg, 2.2 μmol, 2 mol%) was dissolved in anhydrous toluene (1 mL) and stirred for 10 minutes at ambient temperature. To the resulting solution were added **13** (50.0 mg, 0.11 mmol, 1.0 equiv.), 3,5-bis(trifluoromethyl)benzenethiol (**S48**) (54.2 mg, 0.22 mmol, 2.0 equiv.), and potassium *tert*-butoxide (29.7 mg, 0.26 mmol, 2.4 equiv.) and the reaction was heated at 100 °C overnight. After cooling, the crude mixture was diluted with DCM, filtered over a pad of celite and washed with 1 M NaOH solution. The organic layers were separated, dried over anhydrous $MgSO_4$ and concentrated under reduced pressure. The crude residue was further purified via column chromatography (SiO_2, hexane/ethyl acetate) affording the desired product as a red amorphous solid.

Compound 31: product obtained as a red-orange solid. **Yield:** 20.0 mg (26%).

TLC: R_f = 0.82 (hexanes / EtOAc, 9 : 1 V/V)

^{1}H NMR ($CDCl_3$, 500 MHz): δ = 7.84 (s, 4H); 7.72 (s, 2H); 6.99 (d, J = 6.2 Hz, 2H); 6.91-6.84 (m, 4H); 6.61 (d, J = 6.6 Hz, 2H) ppm.

^{13}C NMR ($CDCl_3$, 126 MHz): δ = 149.1, 147.4, 137.3, 133.2, 132.9, 132.7 ,132.3, 130.1, 129.2, 129.0, 128.6, 124.0, 123.9, 122.5, 121.9, 120.7, 119.7 ppm.

^{19}F NMR ($CDCl_3$, 282 MHz): δ = -63.16 ppm.

HRMS (APCI-TOF): *m/z* calcd. for $[C_{32}H_{14}F_{12}S_2]^+$ ($[M]^+$) 690.0339, found 690.0337.

S3.5 Thianthrenation

S3.5.1 Optimization of reaction conditions

The aryl-thianthrenation protocol described by the Ritter group was utilized.[9] This method leverages aryl-thianthrenium salts (Ar-TT^+) to achieve selective C-H functionalization.[10] To prepare these special sulfonium salts we optimized conditions using TTO (5-thianthrene-*S*-oxide), an activator and a base as an additive. The latter was especially crucial in our case to prevent the loss of the TIPS group during thianthrenation. As highlighted below (Scheme S4.) the main challenge was to suppress the formation of side-product **B**, which was detected via LC-MS measurements. Notably, by subjecting **11** to excess reagents, bisthianthrenation was detected, however product **C** could not be isolated.

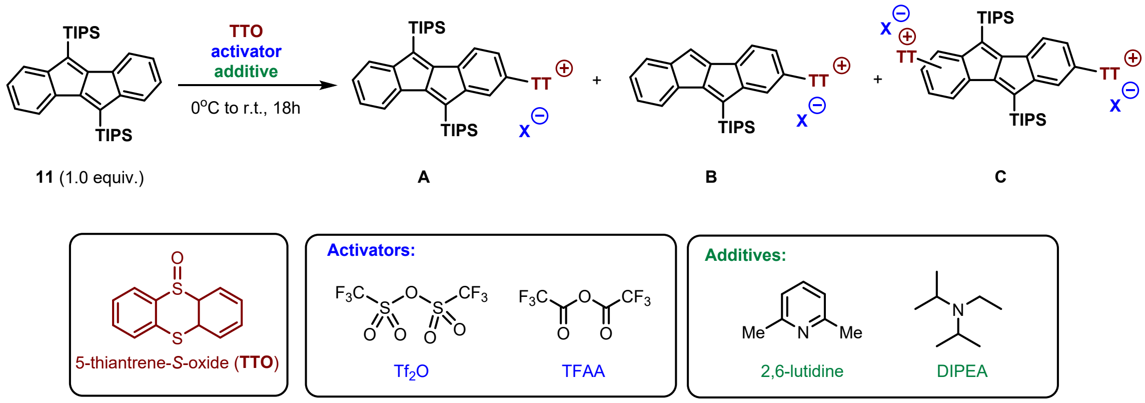

Scheme S4. Thianthrenation of **11** and the key components of the transformation explored during optimization.

In our optimization studies we examined activator reagents, a range of different bases, as well as reaction times and reagent equivalents (Table S10).

Table S10. Conditions explored for thianthrenation of **11**.

Entry	Conditions	Products[Note 1], [Note 2]
1	Tf_2O (1.3 eq.)	Mixture of **A** and **B**
2	Tf_2O (1.3 eq.), K_2CO_3 (2 eq.)	Mixture of **A** and **B**
3	Tf_2O (1.3 eq.), DIPEA (2 eq.)	n.d.
4	Tf_2O (1.3 eq.), 2,6-lutidine (2 eq.)	Formation of **A**: (**45 % NMR yield**; amount of **B** under 10 % based on NMR analysis)
5	Tf_2O (1.3 eq.), 2,6-lutidine (10 eq.)	Formation of **A**: (**50 % NMR yield**; amount of **B** under 10 % based on NMR analysis)
6	TFAA (1.3 eq.)	Formation of **A**: (**25 % NMR yield**; amount of **B** under 10 % based on NMR analysis)
7	TFAA (1.3 eq.), DIPEA (2 eq.)	n.d.
8	TFAA (1.3 eq.), 2,6-lutidine (2 eq.)	n.d.
9	TTO (3 eq.), Tf_2O (3 eq.),	Mixture of **A**, **B** and **C**
10	TTO (3 eq.), Tf_2O (3 eq.), 2,6-lutidine (10 eq.)	Mixture of **A**, and **C**
11	TTO (5 eq.), Tf_2O (5 eq.), 2,6-lutidine (5 eq.)	Complex mixture (**C** was detected as a side product)
12	TTO (10 eq.), Tf_2O (10 eq.), 2,6-lutidine (20 eq.)	Complex mixture (**C** was detected as a side product)

[Note 1]: In-situ analysis of the reaction mixture was carried out using LC-MS to identify components of the reaction mixture; yields were approximated by ^1H NMR spectroscopy.
[Note 2]: In most cases, complete conversion could not be achieved.

*S3.5.1.1 Formation of regioisomeric products upon thianthrenation of **11** and subsequent functionalizations*

Using the optimized conditions for the thianthrenation step, detailed analysis of the analytical data revealed the formation of a small amount of side-product that was inseparable from **34** (Scheme S5). As the NMR signals were largely overlapping, unambiguous assignment was not possible at this stage, although the formation of a regioisomer of **34** was suspected (**34-minor**). Further transformations of **34** to various derivatives (see below) provided evidence for the presence of the two isomers (**34** and **34-minor**), which was especially clear in the formation of **36** and **36-minor**. Due to the inseparable nature of **34** and **34-minor**, their mixture was used in the subsequent coupling reactions, which led to the formation of a minor product (<10%) in each case.

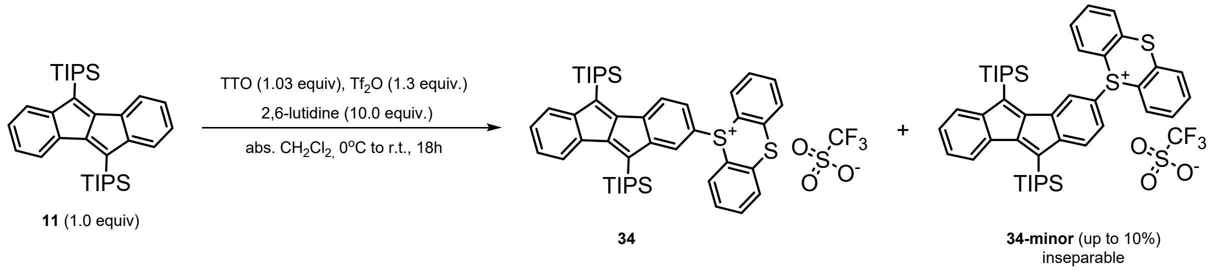

Scheme S5. Formation of regioisomeric products in the thianthrenation of compound **11**.

Interestingly, thianthrenation of dihalogenated DBPs **12** and **13** provided single isomers.

S3.5.2 Experimental procedures and product characterization

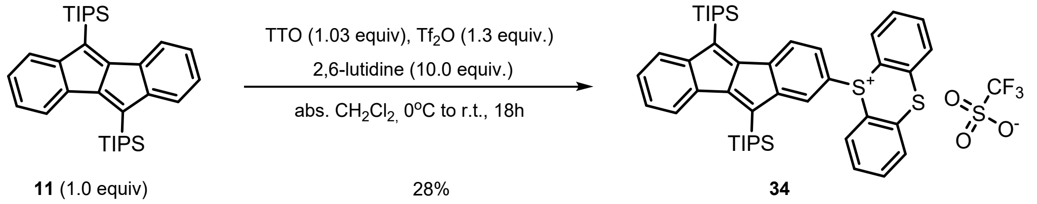

General Procedure G: A 100 mL flask was charged with a magnetic stir bar, followed by the addition of thianthrene-S-oxide (465 mg, 2.00 mmol, 1.03 equiv.) and compound **11** (1.00 g, 1.94 mmol, 1.00 equiv.). The mixture was dissolved in anhydrous dichloromethane (19.5 mL, 0.1 M)[Note 1], after which 2,6-lutidine (2.25 mL, 19.4 mmol, 10.0 equiv.)[Note 2] was added to the solution. The mixture was then cooled to 0 °C using an ice bath. At this temperature, triflic anhydride (427 µL, 2.25 mmol, 1.3 equiv.) was added dropwise while maintaining the internal temperature between 0 - 2 °C. After the addition was complete, the reaction mixture was stirred at 0 °C for 1 h and then allowed to warm to room temperature (22 °C), where it was stirred for an additional 17 h.[Note 3] After completion, hexanes (200 mL) was added slowly to the reaction mixture and stirred for 10 min. The solution was then decanted, and the remaining thick oil/waxy solid was washed twice with hexanes (200 mL portions).[Note 4] The crude product was purified by flash column chromatography (SiO_2, 0% methanol in dichloromethane grading to 10% methanol in dichloromethane). The resulting brown oil was dissolved in THF (10 mL), and then, while sonicating the mixture in an ultrasonic bath, hexanes (200 mL) was added dropwise to precipitate the product. After 10 min, the mixture was cooled to 0 °C and kept at this temperature for another 10 min. The resulting brown solid was collected by filtration and dried under vacuum to afford the product **34** (480 mg, 0.546 mmol, 28%).

[Note 1]: Dichloromethane, stabilized with amylene, was freshly distilled from calcium hydride.
[Note 2]: Anhydrous 2,6-lutidine was prepared by distillation from potassium hydroxide.
[Note 3]: At this point, the NMR yield was measured as follows: ethylene carbonate was added as an internal standard to the reaction mixture and a 200 µL aliquot was diluted with deuterated dichloromethane and analyzed by ^{1}H NMR spectroscopy to determine the NMR yield, which was determined to be 50%.

[Note 4]: This anhydrous work-up is crucial to prevent the formation of side product **B** during isolation and to remove excess 2,6-lutidine, which otherwise makes column chromatography and product separation more challenging.

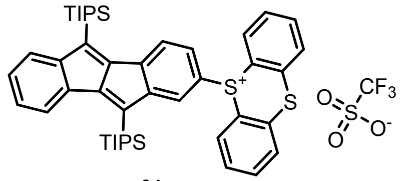

34

Compound 34: product obtained as a brown amorphous solid. **Yield:** 480 mg (28%). (*See also section S3.6.1.1.*)

TLC: R_f = 0.29 (5 % methanol in dichloromethane, CAM or 254 nm)

^{1}H NMR (CDCl$_3$, 600 MHz): δ = 8.59 (d, J = 7.9 Hz, 2H); 7.86-7.78 (m, 4H); 7.77-7.73 (m, 2H); 7.28-7.25 (m, 1H); 7.20 (d, J = 7.4 Hz, 1H); 7.12 (d, J = 7.5 Hz, 1H); 6.86-6.85 (m, 2H); 6.84-6.81 (m, 1H); 6.60-6.55 (m, 1H); 1.57-1.45 (m, 3H); 1.22-1.16 (m, 3H); 1.14-1.12 (m, 18H); 0.99-0.97 (m, 18H) ppm.

^{13}C NMR (CDCl$_3$, 151 MHz): δ = 164.42, 158.64, 157.99, 155.48, 148.43, 141.05, 137.05, 136.58, 135.84, 136.65, 134.78, 130.28, 130.10, 129.01, 127.63, 126.88, 126.21, 124.25, 124.10, 121.90, 121.72, 119.03, 19.34, 19.16, 13.44, 13.14 ppm.

^{19}F NMR (CDCl$_3$, 282 MHz): δ = -78.2 (s) ppm.

HRMS (APCI-TOF): *m/z* calcd. for $[C_{46}H_{57}Si_2S_2]^+$ ($[M+H]^+$) 729.3434, found 729.3438. (HRMS mass detected with loss of anion.)

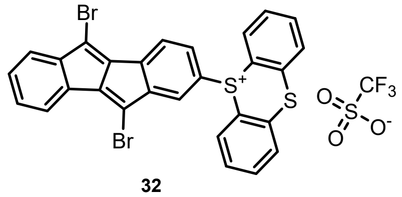

32

Compound **32** was prepared according to **General Procedure G** with the following modifications: The reaction was carried out on a 0.11 mmol scale (40 mg, 1.0 equiv.); thianthrene-S-oxide (27 mg, 0.11 mmol, 1.03 equiv.) triflic anhydride (24 µL, 0.14 mmol, 1.3 equiv.). Purification by precipitation with the mixture of hexanes / THF (10 mL, V/V = 10 : 1, 2 cycles) afforded the title compound **32** (40 mg, 55 mmol, 50%).

Compound 32: product obtained as a brown amorphous solid. **Yield:** 40.0 mg (50%).

TLC: R_f = 0.25 (5 % methanol in dichloromethane, CAM or 254 nm)

^{1}H NMR (CDCl$_3$, 600 MHz): δ = 8.77-8.66 (m, 2H); 7.84-7.83 (m, 2H); 7.81-7.80 (m, 2H); 7.80-7.79 (m, 2H); 7.36 (d, J = 8.1 Hz, 1H); 7.33-7.30 (m, 1H); 7.11-7.08 (m, 1H); 7.07-7.05 (m, 1H); 7.00-6.98 (m, 1H); 6.95 (dd, J = 8.1, 2.0 Hz, 1H); 6.61 (d, J = 1.9 Hz, 1H) ppm.

^{13}C NMR (CDCl$_3$, 151 MHz): δ = 149.7, 147.1, 146.9, 142.1, 137.2, 136.6, 136.1, 134.9, 132.6, 130.5, 130.2, 130.1, 129.3, 126.4, 124.0, 123.4, 123.0, 122.9, 120.0, 122.9, 120.3, 119.2, 117.4 ppm.
(Due to low solubility some ^{13}C signals are assigned based on HSQC and HMBC correlations.)

^{19}F NMR (CDCl$_3$, 282 MHz): δ = -78.2 (s) ppm.

HRMS (APCI-TOF): *m/z* calcd. for $[C_{28}H_{16}S_2Br_2]^+$ ($[M+H]^+$) 573.9054, found 573.9080.

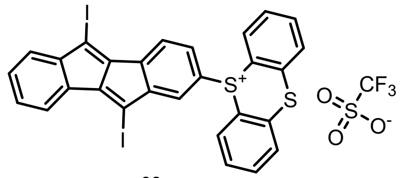

33

Compound **33** was prepared according to **General Procedure G** with the following modifications: The reaction was carried out on a 0.13 mmol scale (57 mg, 1.0 equiv.); thianthrene-S-oxide (30 mg, 0.13 mmol, 1.03 equiv.) triflic anhydride (28 µL, 0.16 mmol, 1.3 equiv.). Purification by precipitation with the mixture of hexanes and THF (10 mL, V/V = 10 : 1, 2 cycles) afforded the title compound **33** (40.0 mg, 55 mmol, 50%).

Compound 33: product obtained as a brown amorphous solid. **Yield:** 41.mg (40 %).

TLC: R_f = 0.25 (5 % methanol in dichloromethane, CAM or 254 nm)

^{1}H NMR (CDCl$_3$, 600 MHz): δ = 8.73 (dd, *J* = 6.8, 2.3 Hz, 2H); 7.83 (m, 2H); 7.81-7.81 (m, 2H); 7.80-7.79 (m, 2H); 7.40 (d, *J* = 8.1 Hz, 1H); 7.32 (d, *J* = 7.4 Hz, 1H); 7.08 (t, *J* = 7.5 Hz, 1H); 7.03 (t, *J* = 7.5 Hz, 1H); 6.96 (dd, *J* = 8.1, 2.0 Hz, 1H); 6.81 (d, *J* = 7.5 Hz, 1H); 6.46 (d, *J* = 2.0 Hz, 1H) ppm.

^{13}C NMR (CDCl$_3$, 151 MHz): δ = 154.5, 152.3, 149.9, 149.8, 138.1, 136.6, 136.1, 134.9, 133.4, 130.2, 130.1, 130.0, 129.1, 125.2, 121.6, 119.1, 101.4, 90.9 ppm.

^{19}F NMR (CDCl$_3$, 282 MHz): δ = -78.1 (s) ppm.

HRMS (APCI-TOF): *m/z* calcd. for $[C_{28}H_{16}S_2Br_2]^+$ ($[M+H]^+$) 573.9054, found 573.9080.

S3.5.3 Thianthrenium salt functionalization

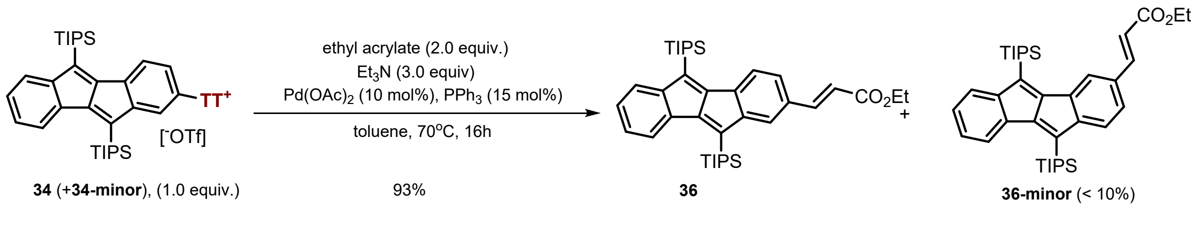

Procedure: A 4 mL scintillation vial was charged with **34** (50.0 mg, 57 µmol, 1.0 equiv.), triphenylphosphine (2.2 mg, 8.5 µmol, 15 mol%), $Pd(OAc)_2$ (1.3 mg, 5.7 µmol, 10 mol%) and was purged with argon. The solid components were dissolved in anhydrous toluene (1.0 mL)

and triethylamine (17.3 mg, 0.17 mmol, 24 µL, 3.0 equiv.) and ethyl acrylate (11.4 mg, 0.11 mmol, 12 µL, 2.0 equiv.) was added using a Hamilton syringe. The resulting mixture was stirred at 70 °C overnight. After cooling to room temperature, the mixture was diluted with DCM and filtered through a pad of celite. The filtrate was concentrated under reduced pressure, and the remaining crude residue was further purified via preparative thin layer chromatography (pTLC, eluent: hexanes / EtOAc, 9 : 1 V/V).

Compound 36: product obtained as a red-orange amorphous solid. **Yield:** 32.6 mg (93%). (*See also section S3.6.1.1*)

TLC: R_f = 0.67 (hexanes / EtOAc, 9 : 1 V/V)

Compound **36**

^{1}H NMR ($CDCl_3$, 500 MHz): δ = 7.54 (d, J = 15.9 Hz, 1H); 7.28-7.25 (m, 3H); 7.14 (m, 1H); 7.03-6.98 (m, 1H); 6.89-6.81 (m, 2H); 6.26 (d, J = 15.9 Hz, 1H); 4.27 (m, 2H); 1.71-1.61 (m, 6H); 1.35 (t, J = 7.1 Hz, 3H); 1.21 (m, 36H) ppm.

^{13}C NMR ($CDCl_3$, 126 MHz): δ = 167.4, 162.6, 160.8, 156.9, 156.1, 145.0, 142.4, 139.8, 138.6, 136.3, 133.9, 128.2, 126.9, 126.6, 125.4, 124.1, 123.8, 123.5, 116.9, 60.7, 19.54, 19.49, 14.5, 13.5 ppm.

Compound **36-minor**

^{1}H NMR ($CDCl_3$, 600 MHz): δ = 7.54 (d, J = 15.9 Hz , 1H) , 7.44 (d , J = 1.7 Hz , 1H) , 7.27 (m, 1H) , 7.1 3 (m, 2H) , 7.02 (dd , J = 8.0, 1.6 Hz , 1H) , 6.87 (m, 1H) , 6.84 (m, 1H) , 6.25 (d , J = 16.1 Hz , 1H) , 4.27 (m, 2H) , 1.71 – 1.60 (m, 6H) , 1.34 (t , J = 7.1 Hz , 3H) , 1.23 (d , J = 7.6 Hz , 18 H) , 1.20 (d , J = 7.4 Hz , 18 H).

^{13}C NMR ($CDCl_3$, 151 MHz): δ = 167.43, 163.38, 160.69, 158.49, 156.05, 144.92, 141.90, 139.76, 137.17, 136.21, 132.34, 128.56, 128.29, 126.50, 125.38, 125.08, 123.81, 122.47, 116.76, 60.60, 19.54, 19.49, 14.51, 13.47.

HRMS (APCI-TOF): *m/z* calcd. for $[C_{39}H_{57}O_2Si_2]^+$ ($[M]^+$) 613.3891, found 613.3899.

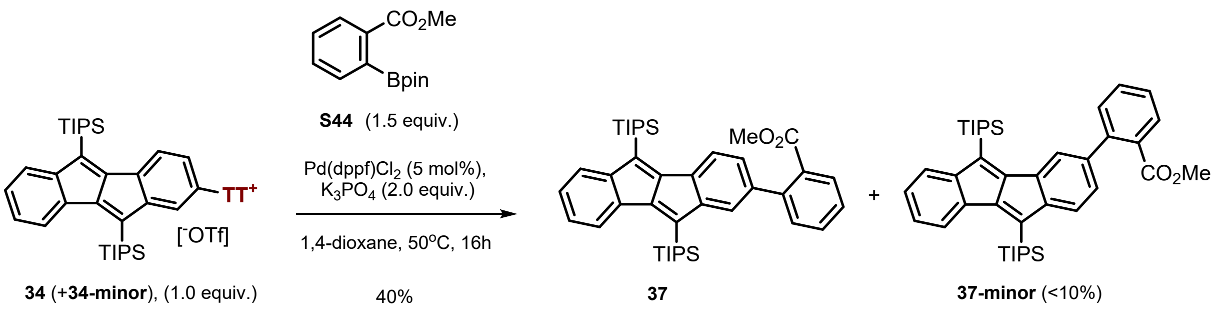

Procedure: A 4 mL scintillation vial was charged with **34** (50.0 mg, 57 µmol, 1.0 equiv.), boronic acid pinacol ester **S44** (22.4 mg, 85.3 µmol, 1.5 equiv.), Pd(dppf)Cl_2 (2.1 mg, 2.8 µmol, 5 mol%), potassium phosphate (24.1 mg, 0.11 mmol, 2.0 equiv.) and was purged with argon. The solid components were dissolved in anhydrous 1,4-dioxane (0.5 mL) and the resulting

mixture was stirred at 50 °C overnight. After cooling to room temperature, the mixture was diluted with DCM and filtered through a pad of celite. The filtrate was concentrated under reduced pressure, and the remaining crude residue was further purified via preparative thin layer chromatography (pTLC, eluent: hexanes / EtOAc, 9 : 1 V/V).

Compound 37: product obtained as a red amorphous solid. **Yield:** 15.0 mg (40%). (*See also section S3.6.1.1.*)

TLC: R_f = 0.67 (hexanes / EtOAc, 9 : 1 V/V)

^{1}H NMR (CD_2Cl_2, 500 MHz): δ = 7.78 (d, J = 7.8 Hz, 1H); 7.53 (td, J = 7.6, 1.4 Hz, 1H); 7.40 (td, J = 7.6, 1.4 Hz, 1H); 7.35 (dd, J = 7.7, 1.3 Hz, 1H); 7.31-7.27 (m, 2H); 7.15 (d, J =7.5 Hz, 1H); 7.06 (s, 1H); 6.89-6.80 (m, 2H); 6.74 (dd, J =7.7, 1.6 Hz, 1H); 3.68 (s, 3H); 1.73-1.58 (m, 6H); 1.24-1.17 (m, 36H) ppm.

^{13}C NMR ($CDCl_3$, 126 MHz): δ = 169.3, 162.4, 161.6, 156.3, 156.2, 142.8, 141.1, 140.3, 140.2, 136.5, 135.4, 131.43, 131.41, 131.0, 130.2, 130.1, 130.0, 128.0, 127.2, 126.18, 126.16, 125.4, 125.1, 123.6, 123.0, 52.1, 19.54, 19.53, 13.4 ppm.

HRMS (APCI-TOF): *m/z* calcd. for $[C_{42}H_{57}O_2Si_2]^+$ ($[M+H]^+$) 649.3891, found 649.3862.

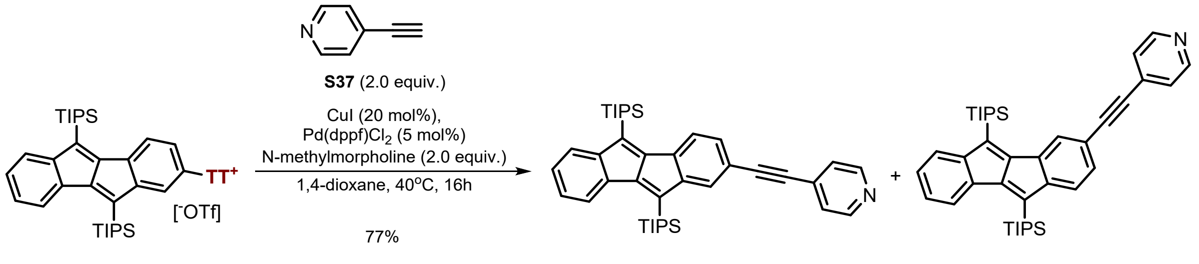

Procedure: A 4 mL scintillation vial was charged with **34** (50.0 mg, 57 µmol, 1.0 equiv.), CuI (2.1 mg, 11 µmol, 20 mol%), $Pd(dppf)Cl_2$ (2.1 mg, 2.8 µmol, 5 mol%), 4-ethynylpyridine (11.7 mg, 0.11 mmol, 2.0 equiv.) and was purged with argon. The solid components were dissolved in anhydrous 1,4-dioxane (1.0 mL) and N-methylmorpholine (11.5 mg, 0.11 mmol, 13 µL, 2.0 equiv.) was added using a Hamilton syringe. The resulting mixture was stirred at 40 °C overnight. After cooling to room temperature, the mixture was diluted with DCM and filtered through a pad of celite. The filtrate was concentrated under reduced pressure, and the remaining crude residue was further purified via preparative thin layer chromatography (pTLC, eluent: hexanes / EtOAc, 7 : 1 V/V).

Compound 38: product obtained as a red amorphous solid. **Yield:** 27 mg (77%). (*See also section S3.6.1.1.*)

TLC: R_f = 0.18 (hexanes / EtOAc, 9 : 1 V/V)

^{1}H NMR (CD_2Cl_2, 500 MHz): δ = 8.60 (bs, 2H); 7.37 (d, J = 5.8 Hz, 2H); 7.28-7.23 (m, 3H); 7.13 (m, 1H); 7.07-7.02 (m, 1H); 6.89-6.81 (m, 2H); 1.65 (m, 6H); 1.22 (m, 36H) ppm.

^{13}C NMR (CDCl$_3$, 126 MHz): δ = 163.2, 161.2, 156.7, 156.4, 149.7, 143.1, 139.8, 137.8, 136.6, 130.6, 128.7, 128.6, 128.1, 127.0, 126.0, 125.9, 125.8, 124.2, 123.6, 121.3, 95.6, 87.2, 19.55, 19.51, 13.73, 13.67 ppm.

HRMS (ESI-TOF): *m/z* calcd. for $[C_{41}H_{54}NSi_2]^+$ ($[M+H]^+$) 616.3789, found 616.3801.

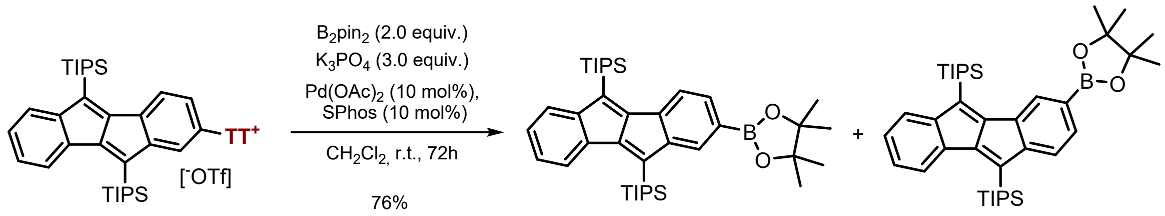

34 (+**34-minor**), (1.0 equiv.) **39** **39-minor** (< 10%)

Procedure: A 4 mL scintillation vial was charged with **34** (50.0 mg, 57 µmol, 1.0 equiv.), B_2pin_2 (28.9 mg, 0.11 mmol, 2.0 equiv.), $Pd(OAc)_2$ (1.3 mg, 5.7 µmol, 10 mol%), SPhos (2.3 mg, 5.7 µmol, 10 mol%), potassium phosphate (36.2 mg, 0.17 mmol, 3.0 equiv.) and was purged with argon. The solid components were dissolved in anhydrous DCM (1.0 mL) and the resulting mixture was stirred at room temperature for 72 hours. After completion, the mixture was diluted with DCM and filtered through a pad of celite. The filtrate was concentrated under reduced pressure, and the remaining crude residue was further purified via preparative thin layer chromatography (pTLC, eluent: hexanes / EtOAc, 7 : 1 V/V).

Compound 39: product obtained as a dark-red amorphous solid. **Yield:** 28.0 mg (76%). (*See also section S3.6.1.1.*)

TLC: R_f = 0.77 (hexanes / EtOAc, 7 : 1 V/V)

^{1}H NMR (CD$_2$Cl$_2$, 600 MHz): δ = 7.52 (s, 1H); 7.28-7.25 (m, 2H); 7.23 (dd, *J* = 7.4, 1.0 Hz, 1H); 7.15-7.13 (m, 1H); 6.87-6.84 (m, 1H); 6.84-6.81 (m, 1H); 1.74-1.62 (m, 6H); 1.30 (s, 12H); 1.21 (d, *J* = 7.5, 18H); 1.19 (d, *J* = 7.5 Hz, 18H) ppm.

^{13}C NMR (CD$_2$Cl$_2$, 151 MHz): δ = 161.9, 161.4, 156.3, 155.5, 141.8, 141.5, 139.3, 136.7, 133.6, 131.3, 129.1 (m), 128.1, 126.7, 125.6, 123.9, 123.2, 84.0, 25.0, 19.6, 19.5, 13.7, 13.6 ppm.

HRMS (APCI-TOF): *m/z* calcd. for $[C_{40}H_{61}BO_2Si_2]^+$ ($[M+H]^+$) 640.4297, found 640.4292.

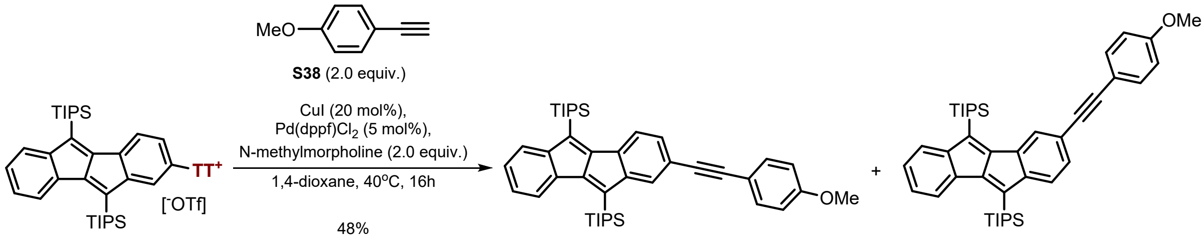

34 (+**34-minor**), (1.0 equiv.) **40** **40-minor** (< 10%)

Procedure: A 4 mL scintillation vial was charged with **34** (50.0 mg, 57 µmol, 1.0 equiv.), CuI (2.1 mg, 11 µmol, 20 mol%), $Pd(dppf)Cl_2$ (2.1 mg, 2.8 µmol, 5 mol%), 1-ethynyl-4-

methoxybenzene (15.0 mg, 0.11 mmol, 2.0 equiv.) and was purged with argon. The solid components were dissolved in anhydrous 1,4-dioxane (1.0 mL) and *N*-methylmorpholine (11.5 mg, 0.11 mmol, 13 µL, 2.0 equiv.) was added using a Hamilton syringe. The resulting mixture was stirred at 50 °C overnight. After cooling to room temperature, the mixture was diluted with DCM and filtered through a pad of celite. The filtrate was concentrated under reduced pressure, and the remaining crude residue was further purified via preparative thin layer chromatography (pTLC, eluent: hexanes / EtOAc, 9 : 1 V/V).

Compound 40: product obtained as a red amorphous solid. **Yield:** 17.8 mg (48%). (*See also section S3.6.1.1.*)

TLC: $R_f = 0.77$ (hexanes / EtOAc, 9 : 1 V/V)

^{1}H NMR ($CDCl_3$, 600 MHz): δ = 7.48-7.46 (m, 2H); 7.27-7.25 (m, 1H); 7.21 (m, 2H); 7.12 (dd, J = 7.4, 1.3 Hz, 1H); 6.98 (dd, J = 7.8, 1.3 Hz, 1H); 6.89-6.86 (m, 2H); 6.86 (m, 1H); 6.83-6.82 (m, 1H); 3.83 (s, 3H); 1.68 (m, 3H); 1.65 (m, 3H); 1.22 (d, J = 7.5 Hz, 18H); 1.20 (d, J = 7.5 Hz, 18H) ppm.

^{13}C NMR ($CDCl_3$, 151 MHz): δ = 162.5, 161.3, 159.7, 156.3, 156.2, 141.2, 139.9, 136.4, 136.0, 133.2, 129.6, 128.1, 127.7, 126.4, 125.2, 123.7, 123.2, 122.7, 115.8, 114.1, 89.7, 89.1, 55.5, 19.6, 19.5, 13.43, 13.41 ppm.

HRMS (APCI-TOF): *m/z* calcd. for $[C_{43}H_{57}OSi_2]^+$ ($[M+H]^+$) 645.3942, found 645.3925.

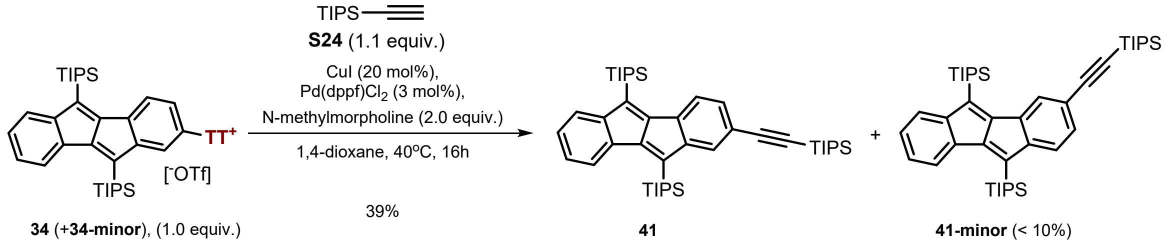

Procedure: A 4 mL scintillation vial was charged with **34** (45.0 mg, 51 µmol, 1.0 equiv.), CuI (1.9 mg, 10 µmol, 20 mol%), $Pd(dppf)Cl_2$ (1.1 mg, 1.5 µmol, 3 mol%) and was purged with argon. The solid components were dissolved in anhydrous 1,4-dioxane (0.4 mL) and *N*-methylmorpholine (10.4 mg, 0.1 mmol, 11 µL, 2.0 equiv.), triisopropyl(silylacetlyene) (10.3 mg, 56.3 µmol, 13 µL, 1.1 equiv.) were added using a Hamilton syringe. The resulting mixture was stirred at 40 °C overnight. After cooling to room temperature, the mixture was diluted with DCM and filtered through a pad of celite. The filtrate was concentrated under reduced pressure, and the remaining crude residue was further purified via preparative thin layer chromatography (pTLC, eluent: hexanes).

Compound 41: product obtained as a red amorphous solid. **Yield:** 14.1 mg (39%). (*See also section S3.6.1.1.*)

TLC: $R_f = 0.64$ (hexanes)

^{1}H NMR (CD_2Cl_2, 500 MHz): δ = 7.29-7.19 (m, 3H); 7.14 (d, J = 7.2 Hz, 1H); 6.94 (d, J = 7.7 Hz, 1H); 6.89-6.80 (m, 2H); 1.70-1.60 (m, 6H); 1.20 (t, J = 7.5 Hz, 36H); 1.13 (s, 21H) ppm.

^{13}C NMR (CD_2Cl_2, 126 MHz): δ = 162.6, 161.4, 156.5, 156.4, 142.0, 140.1, 136.7, 136.5, 129.8, 129.0, 128.5, 126.8, 125.7, 124.1, 123.5, 122.9, 108.4, 91.5, 19.53, 19.50, 18.9, 13.69, 13.66, 11.8 ppm.

HRMS (APCI-TOF): *m/z* calcd. for $[C_{45}H_{71}Si_3]^+$ ($[M+H]^+$) 695.4858, found 695.4871.

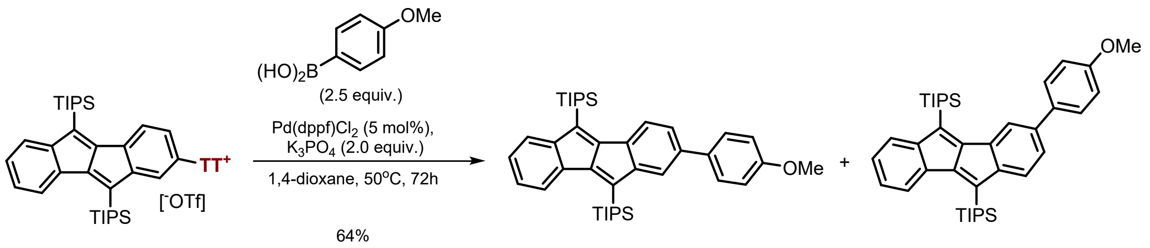

34 (+**34-minor**), (1.0 equiv.) **42** **42-minor** (< 10%)

Procedure: A 4 mL scintillation vial was charged with **34** (130.0 mg, 0.15 mmol, 1.0 equiv.), 4-methoxy(phenylboronic) acid (56.2 mg, 0.37 mmol, 2.5 equiv.), Pd(dppf)Cl_2 (5.4 mg, 7.4 µmol, 5 mol%), potassium phosphate (62.7 mg, 0.29 mmol, 2.0 equiv.) and was purged with argon. The solid components were dissolved in anhydrous 1,4-dioxane (2.5 mL) and the resulting mixture was stirred at 50 °C for 72 hours. After cooling to room temperature, the mixture was diluted with DCM and filtered through a pad of celite. The filtrate was concentrated under reduced pressure, and the remaining crude residue was further purified via column chromatography (SiO_2, hexanes). Flash chromatography was repeated twice to afford the desired product without residual thianthrene contamination.

Compound 42: product obtained as a dark-red amorphous solid. **Yield:** 58.3 mg (64%). (*See also section S3.6.1.1.*)

TLC: R_f = 0.61 (hexanes / EtOAc, 12 : 1 V/V)

^{1}H NMR ($CDCl_3$, 500 MHz): δ = 7.46 (d, J = 8.6 Hz, 2H); 7.35 (s, 1H); 7.31 (d, J = 7.9 Hz, 1H); 7.28 (d, J = 7.2 Hz, 1H); 7.14 (d, J = 7.2 Hz, 1H); 7.02 (d, J = 8.6 Hz, 1H); 6.97 (d, J = 8.7 Hz, 2H); 6.88-6.80 (m, 2H); 3.84 (s, 3H); 1.75-1.64 (m, 6H); 1.26-1.20 (m, 36H) ppm.

^{13}C NMR ($CDCl_3$, 126 MHz): δ = 162.7, 161.7, 159.8, 157.4, 156.7, 140.6, 140.5, 140.3, 136.7, 135.0, 134.0, 128.3, 127.8, 126.5, 125.4, 124.5, 124.13, 124.10, 124.0, 114.7, 55.7, 19.62, 19.60, 19.56, 13.8, 13.7 ppm.

HRMS (APCI-TOF): *m/z* calcd. for $[C_{41}H_{57}Si_2]^+$ ($[M+H]^+$) 621.3942, found 621.3923.

S3.6 Further attempted transformations of dihalo-DBPs

Several attempted transformations in the *5,10* positions of dihalo-DBPs were unsuccessful (Table S11-S13).

Table S11. Attempts of borylation of **13**.

Entry	Conditions	Product [%]
1	*n*BuLi (1.1 equiv.), O^{i}PrBpin (1.1 equiv.), THF, -78°C to r.t., 16h	n.d.
2	B_2pin_2 (3.0 equiv.), Pd(dppf)Cl_2 (5 mol%), XPhos (5 mol%), KOAc (6.0 equiv.), PEG-2000, 110°C, 16h	n.d.
3	B_2pin_2 (2.2 equiv.), Pd(PPh_3)Cl_2 (3 mol%), PPh_3 (6 mol%), K_2CO_3 (1.5 equiv.), toluene, 50°C, 16h	n.d.

Table S12. Attempts of formylation of **13**.

Entry	Conditions	Product [%]
1	*n*BuLi (1.1 equiv.), DMF (1.1 equiv.), THF, -78°C to r.t., 16h	n.d.
2	HCO_2H (8.0 equiv.), I_2 (2.4 equiv.), PPh_3 (2.4 equiv.), Pd(OAc)$_2$ (6 mol%), Et_3N (12 equiv.), toluene, 80°C	n.d.

Table S13. Attempts of *5,10*-dehalogenation of **12** and **13**.

Entry	Conditions	Product [%]
1	TTMSS (2.0 equiv.), AIBN (30 mol%), H_2O, 100°C, 4h	n.d.
2	*n*Bu_3SnH (3.0 equiv.), AIBN (10 mol%), toluene, 110°C, 16h	n.d.

S4 X-ray crystallographic data

Single crystals of $C_{17}H_{25}Si$ (**11**) obtained by recrystallization from chloroform. Single crystals of $C_{16}H_8Br_2$ (**12**) were obtained by recrystallization from chloroform/methanol. X-ray diffraction data were collected from a single crystal at cryogenic temperatures for both compounds. The crystals were mounted on MD loops with Mitegen's LV CryoOil™ and measured on a XtaLAB Synergy-R, HyPix rotating anode diffractometer using Cu Kα radiation (wavelength: 1.54184 Å) using omega scans. Data collection and data reduction were carried out using CrysAlisPro (Rigaku). Using Olex2,[11] the structure was solved with the SHELXT[12] structure solution program using Intrinsic Phasing and refined with the SHELXL[13] refinement package using Least Squares minimisation. All non-hydrogen atoms were refined anisotropically. Hydrogen atoms were generated based upon geometric evidence and their positions were refined by the riding model. Crystallographic data are summarized in Tables S14 and S15 for compounds **11** and **12**, respectively.

The structures of **11** and **12** were deposited (Cambridge Crystallographic Data Centre) and are available free of charge with CCDC deposition numbers 2503542 (**11**) and 2503560 (**12**) respectively via www.ccdc.cam.ac.uk/getstructures.[14]

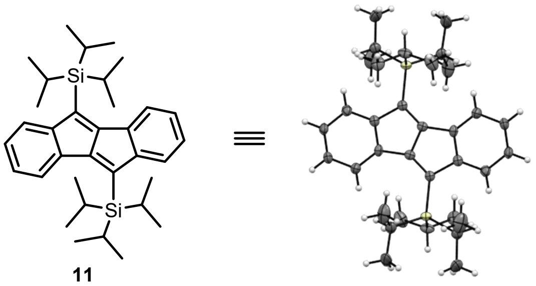

11

Table S14. Crystal data and structure refinement for compound **11**.

Identification code	Compound **11**
Empirical formula	$C_{17}H_{25}Si$
Formula weight	257.46
Temperature/K	105.88(10)
Crystal system	monoclinic
Space group	$P2_1/c$
a/Å	12.9337(2)
b/Å	7.34533(9)
c/Å	17.1434(3)
α/°	90
β/°	111.946(2)
γ/°	90
Volume/Å^3	1510.65(5)
Z	4
ρ_{calc} / gcm^{-3}	1.132
μ /mm^{-1}	1.195

F(000)	564.0
Crystal size/mm^3	0.254 × 0.116 × 0.112
Radiation	Cu Kα (λ = 1.54184)
2Θ range for data collection/°	7.368 to 151.118
Index ranges	$-15 \leq h \leq 16$, $-9 \leq k \leq 8$, $-20 \leq l \leq 21$
Reflections collected	13746
Independent reflections	2924 [R_{int} = 0.0401, R_{sigma} = 0.0310]
Data/restraints/parameters	2924/0/169
Goodness-of-fit on F^2	1.062
Final R indexes [I>=2σ (I)]	R_1 = 0.0440, wR_2 = 0.1092
Final R indexes [all data]	R_1 = 0.0493, wR_2 = 0.1134
Largest diff. peak/hole / e Å^{-3}	0.53/-0.35

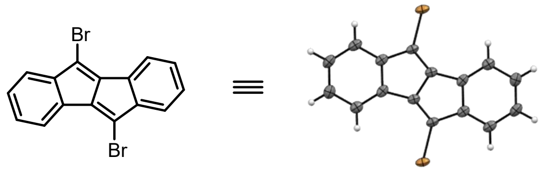

12

Table S15. Crystal data and structure refinement for compound **12**.

Identification code	Compound **12**
Empirical formula	$C_{16}H_8Br_2$
Formula weight	360.04
Temperature/K	106.15
Crystal system	monoclinic
Space group	I2/a
a/Å	18.2774(4)
b/Å	3.89392(8)
c/Å	17.2149(4)
α/°	90
β/°	94.030(2)
γ/°	90
Volume/Å^3	1222.17(4)
Z	4
ρ_{calc} / g cm^{-3}	1.957
μ/mm^{-1}	8.196
F(000)	696.0
Crystal size/mm^3	0.27 × 0.095 × 0.057
Radiation	CuKα (λ = 1.54184)
2Θ range for data collection/°	9.702 to 149.746

Index ranges	$-22 \leq h \leq 22$, $-3 \leq k \leq 4$, $-21 \leq l \leq 19$
Reflections collected	5797
Independent reflections	1212 [R_{int} = 0.0394, R_{sigma} = 0.0246]
Data/restraints/parameters	1212/0/82
Goodness-of-fit on F^2	1.123
Final R indexes [I>=2σ (I)]	R_1 = 0.0370, wR_2 = 0.0969
Final R indexes [all data]	R_1 = 0.0387, wR_2 = 0.0983
Largest diff. peak/hole / e Å^{-3}	1.80/-0.94

S5 NMR spectra

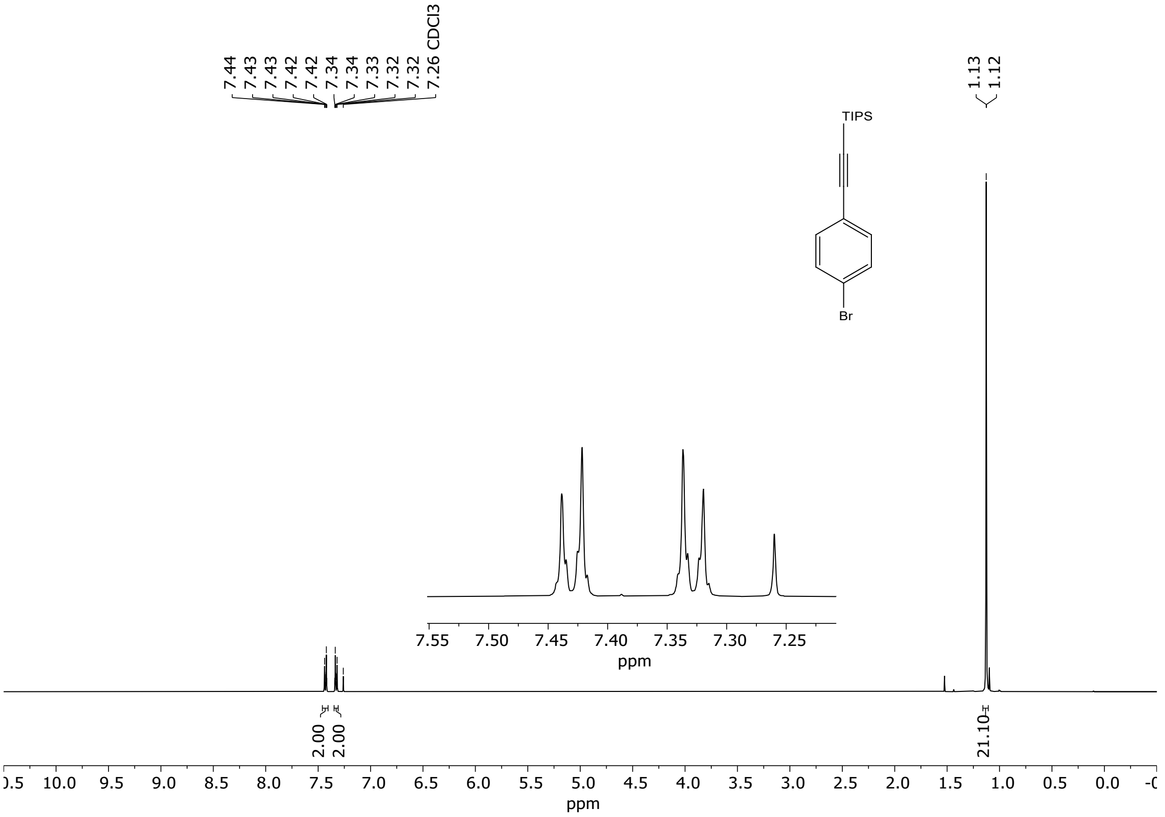

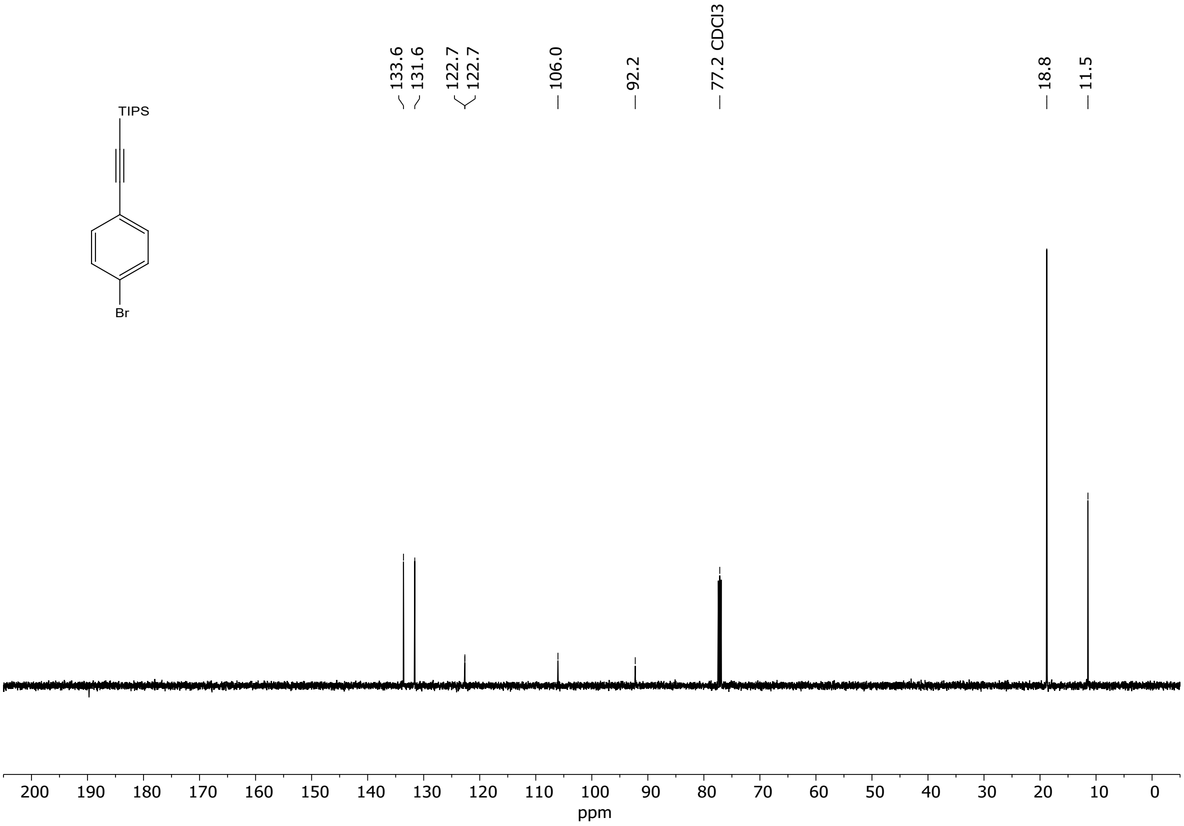

Figure S3. NMR spectra of compound **S17** – ^{1}H, $CDCl_3$, 500 MHz (top); ^{13}C, $CDCl_3$, 126 MHz (bottom).

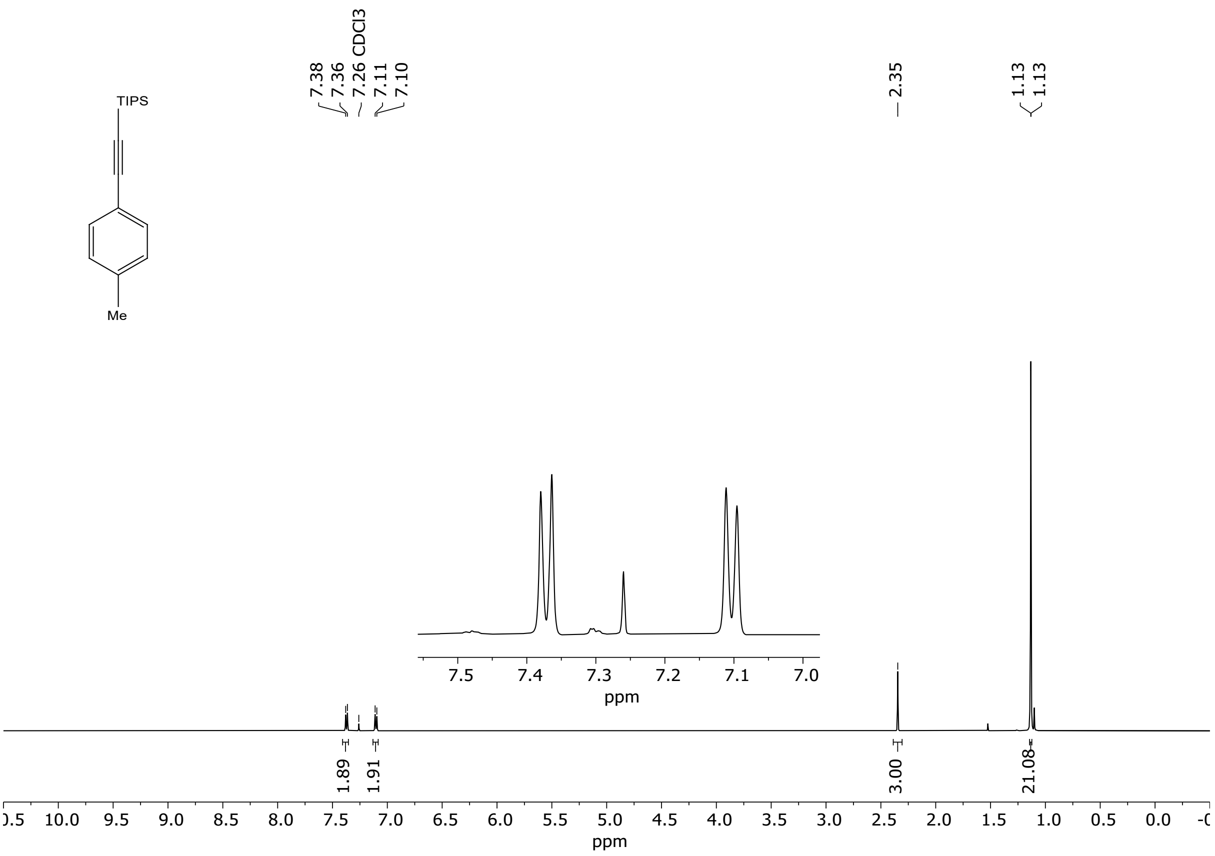

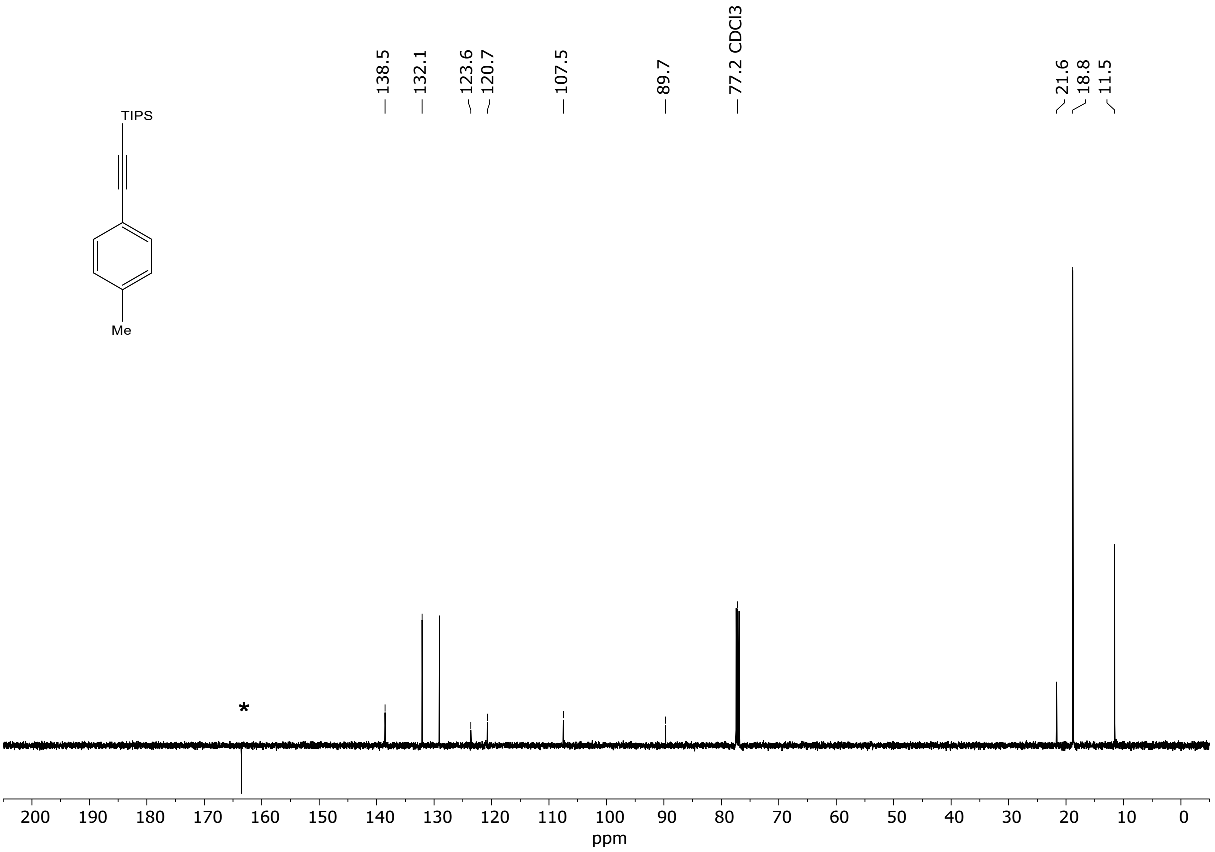

Figure S4. NMR spectra of compound **S18** – ^{1}H, $CDCl_3$, 500 MHz (top); ^{13}C, $CDCl_3$, 126 MHz (bottom). The asterisk marks a noise from the instrument.

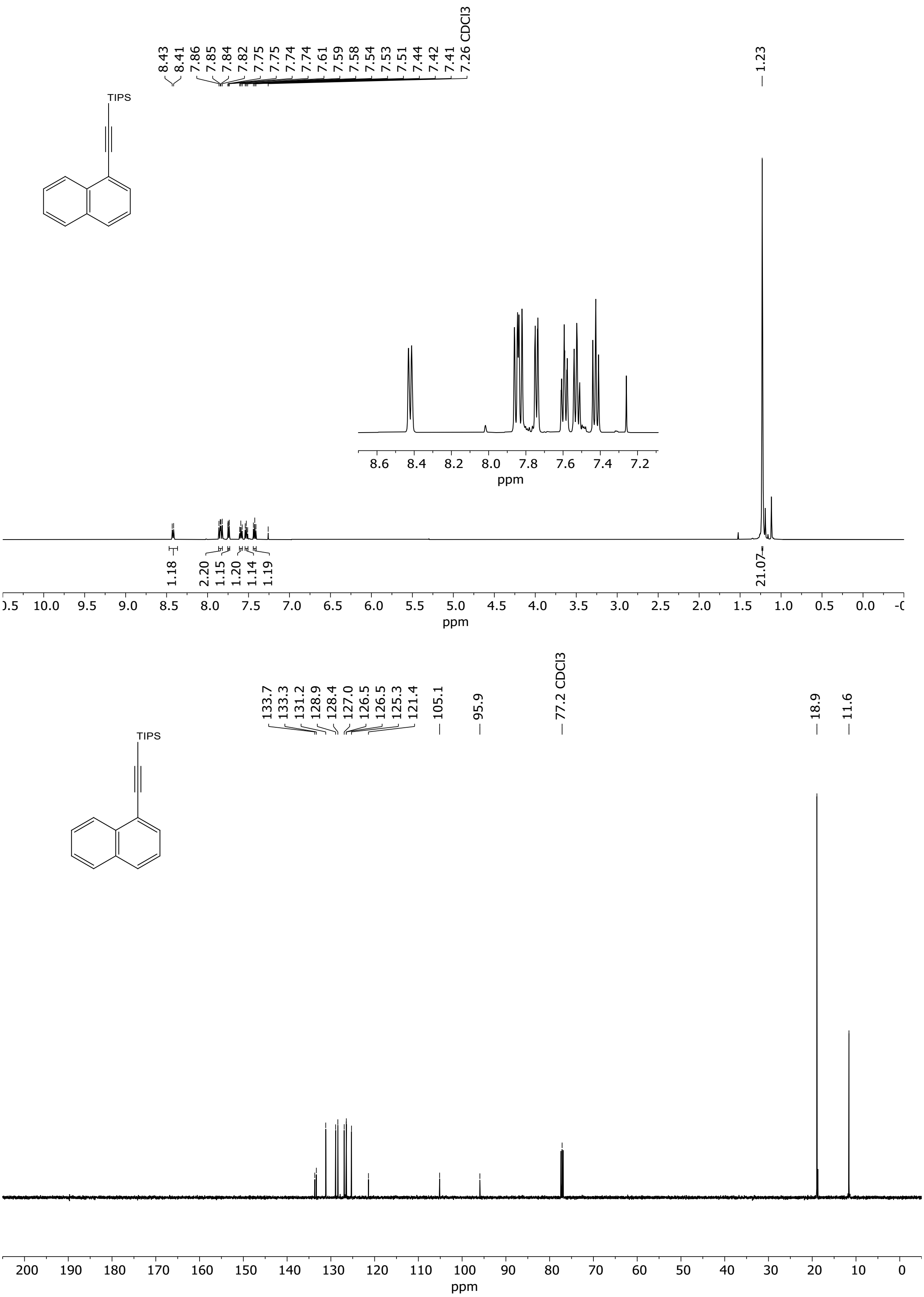

Figure S5. NMR spectra of compound **S19** – ^{1}H, $CDCl_3$, 500 MHz (top); ^{13}C, $CDCl_3$, 126 MHz (bottom).

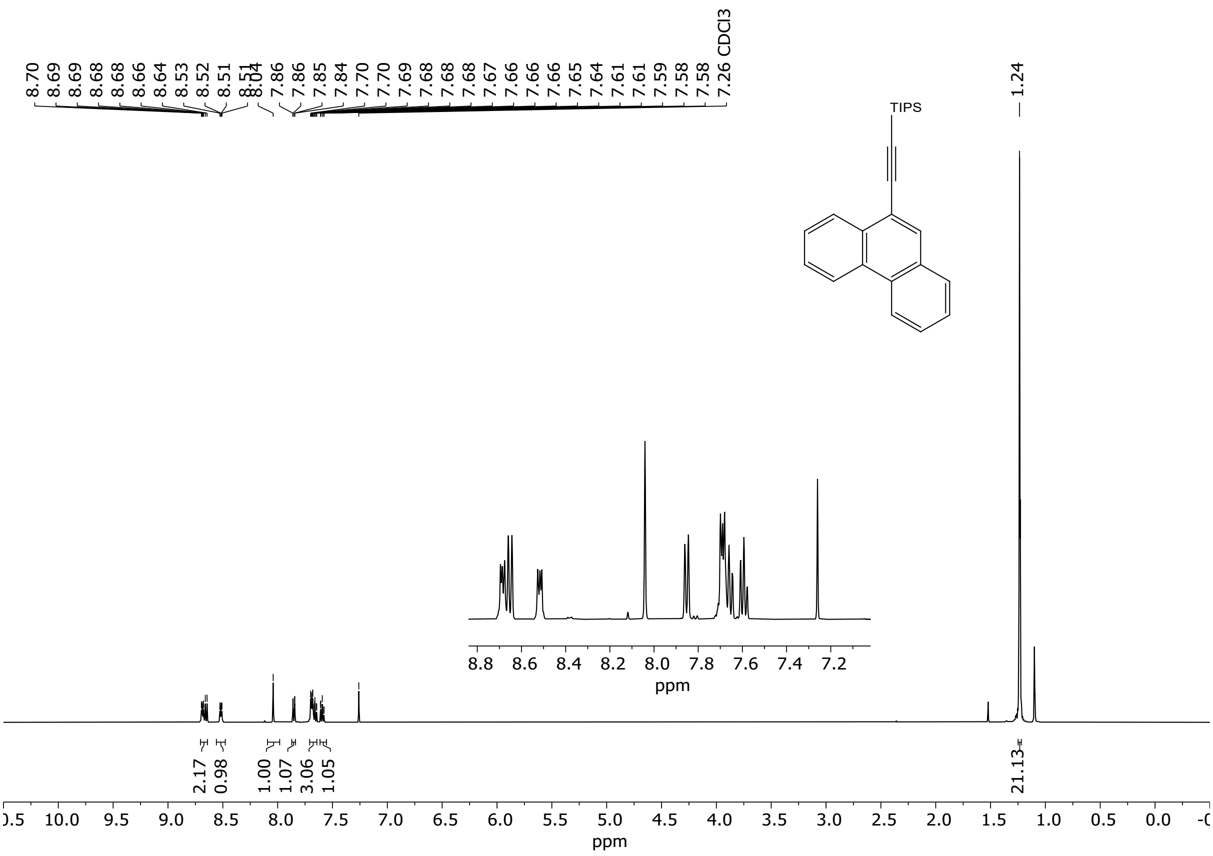

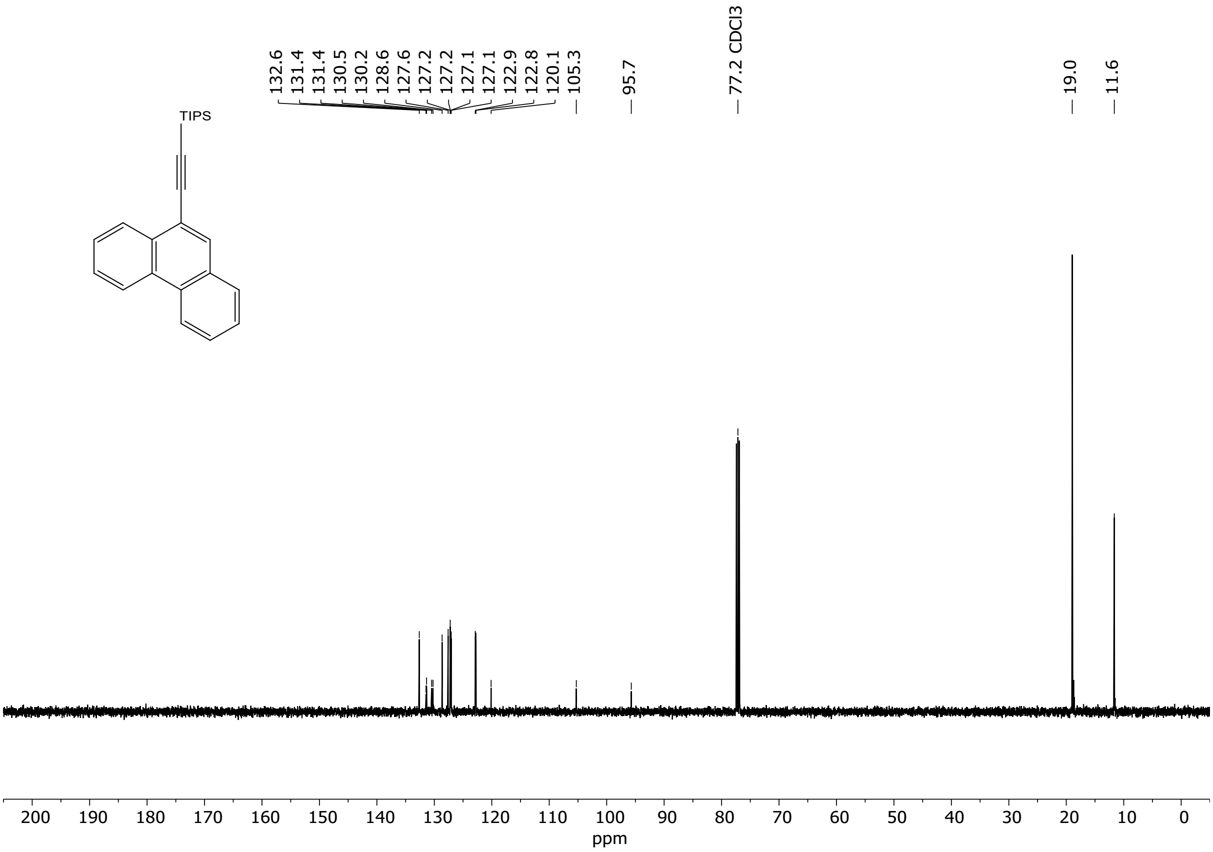

Figure S6. NMR spectra of compound **S20** – ^{1}H, $CDCl_3$, 500 MHz (top); ^{13}C, $CDCl_3$, 126 MHz (bottom).

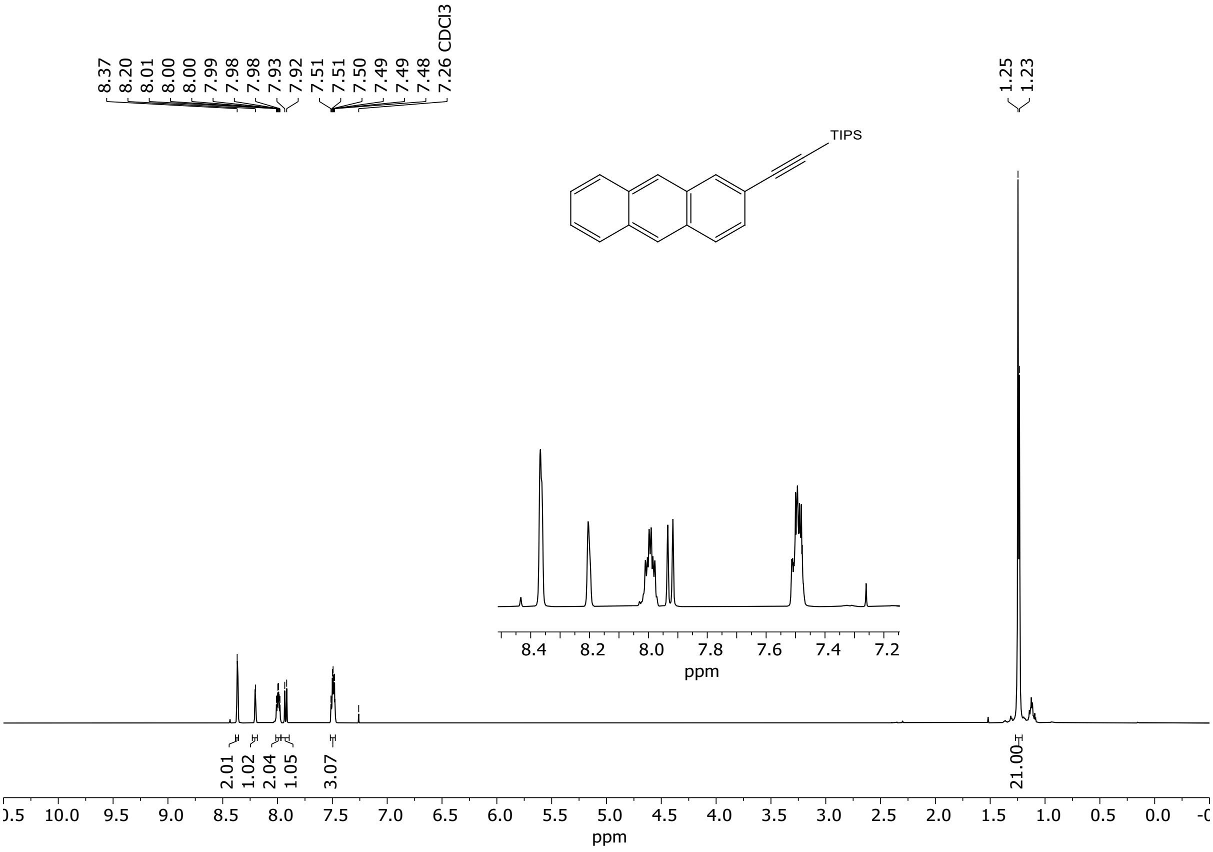

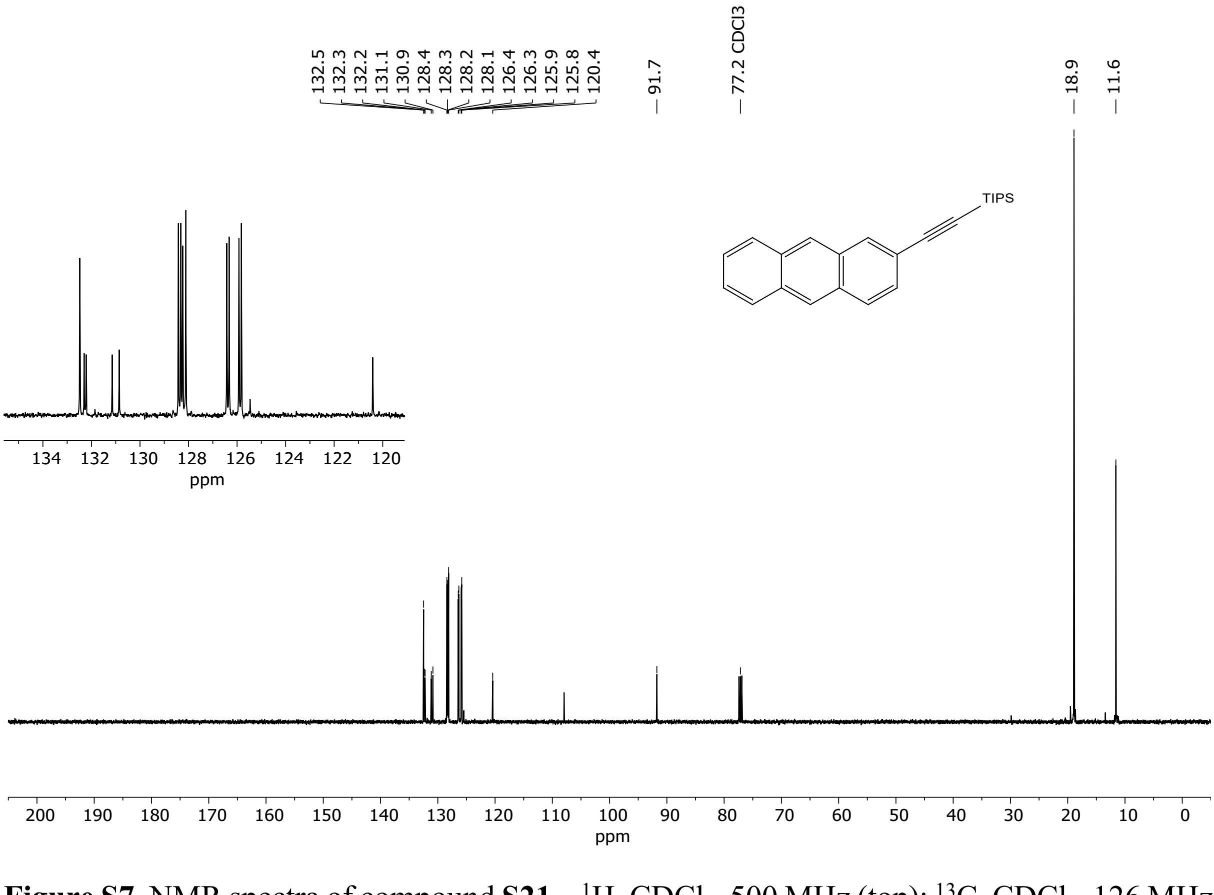

Figure S7. NMR spectra of compound **S21** – ^{1}H, $CDCl_3$, 500 MHz (top); ^{13}C, $CDCl_3$, 126 MHz (bottom).

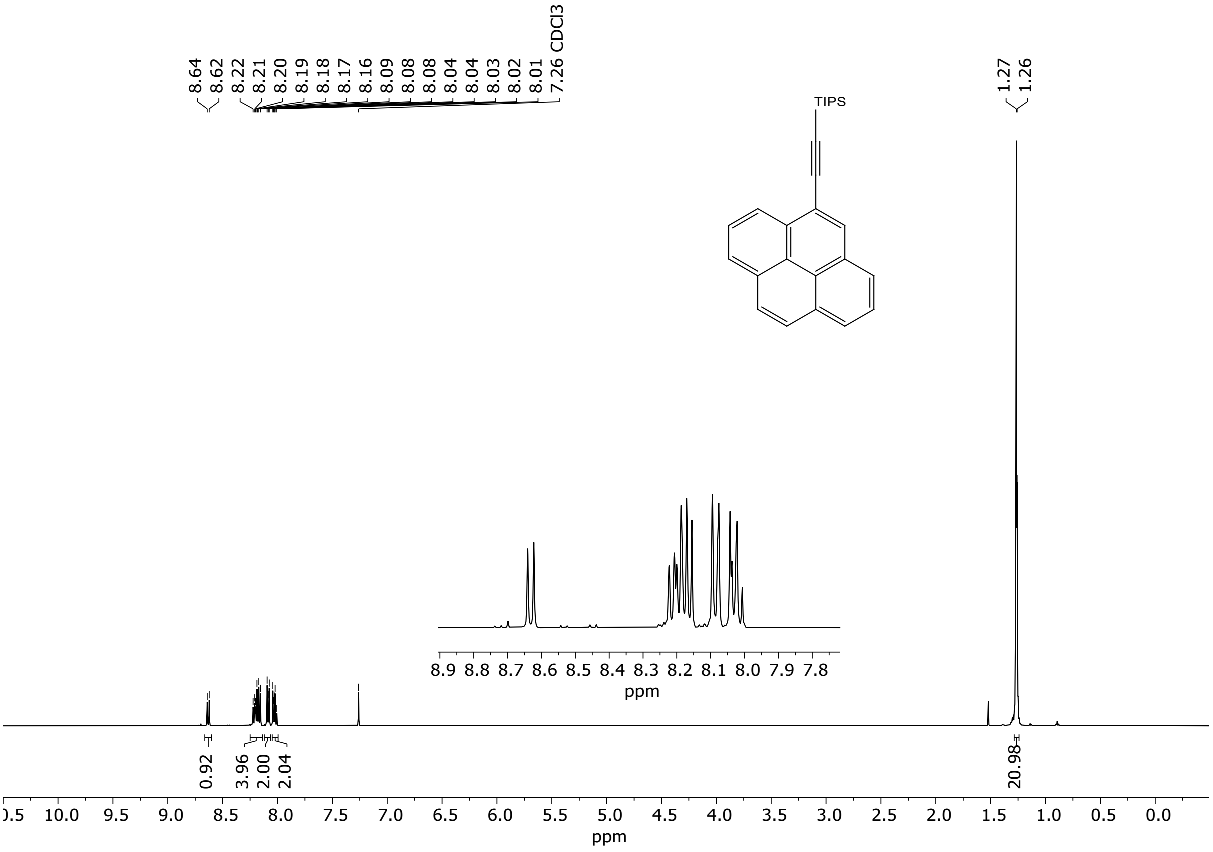

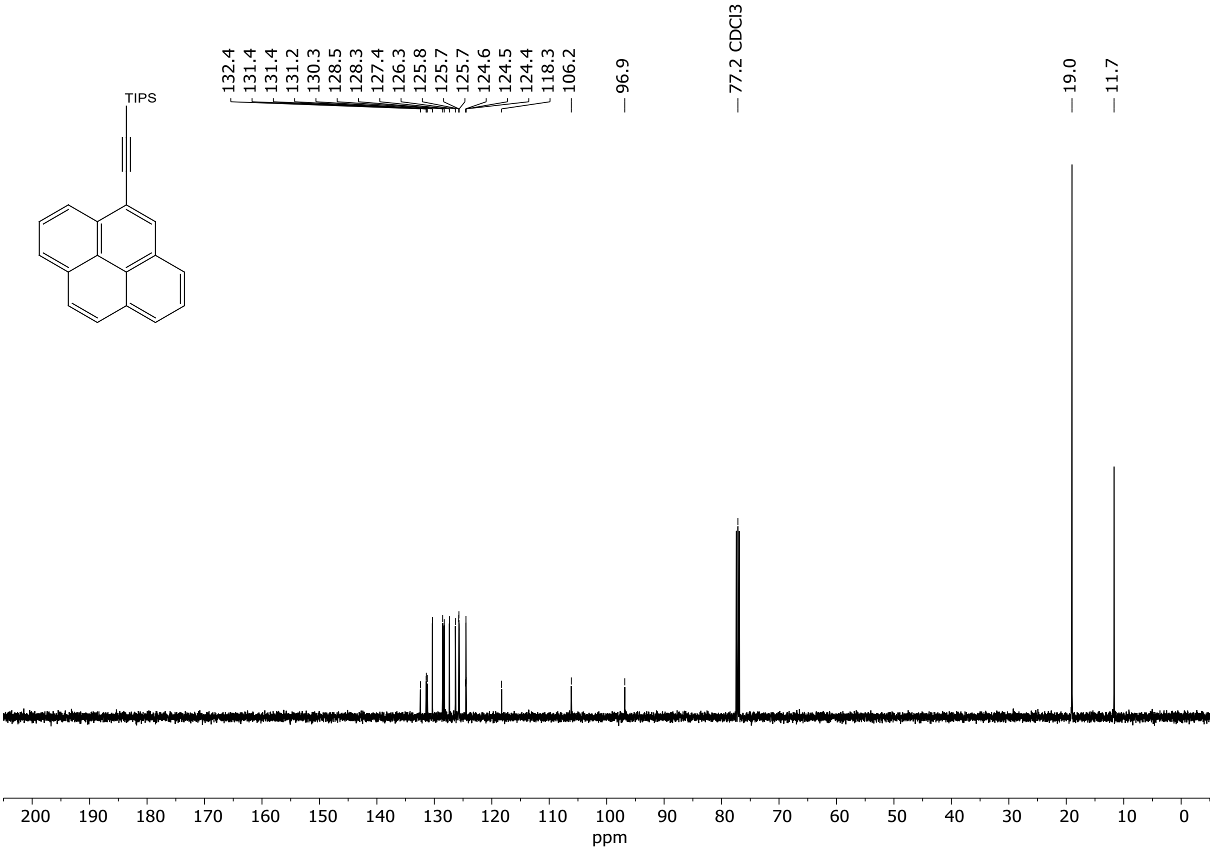

Figure S8. NMR spectra of compound **S22** – ^{1}H, $CDCl_3$, 500 MHz (top); ^{13}C, $CDCl_3$, 126 MHz (bottom).

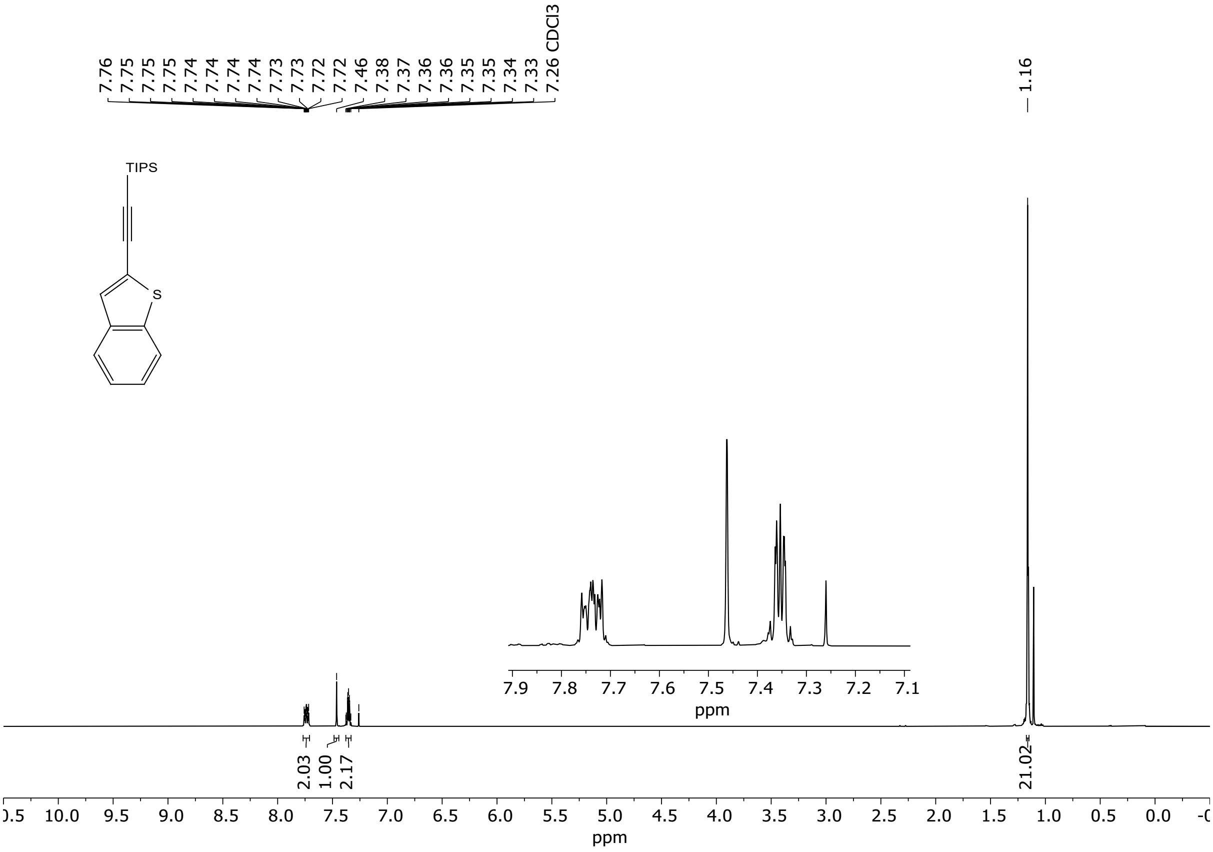

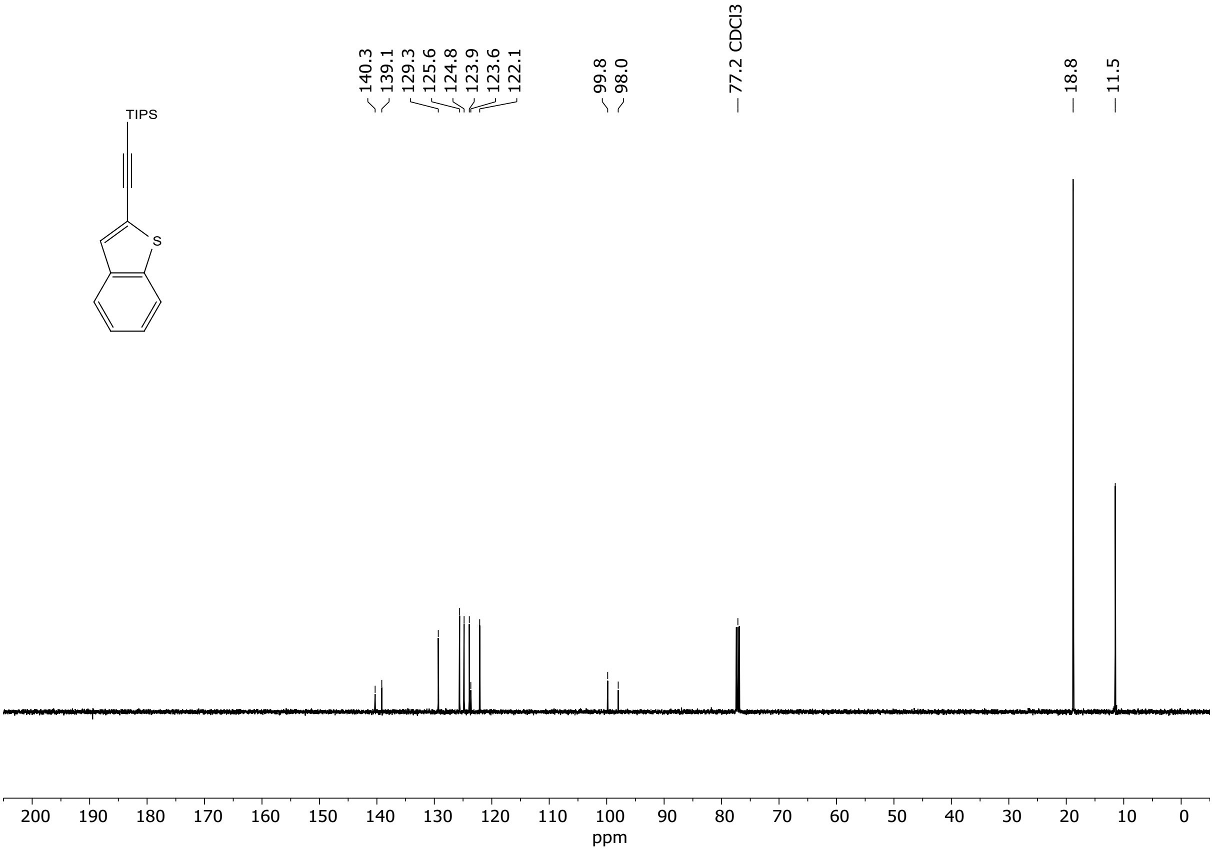

Figure S9. NMR spectra of compound **S24** – ^{1}H, $CDCl_3$, 500 MHz (top); ^{13}C, $CDCl_3$, 126 MHz (bottom).

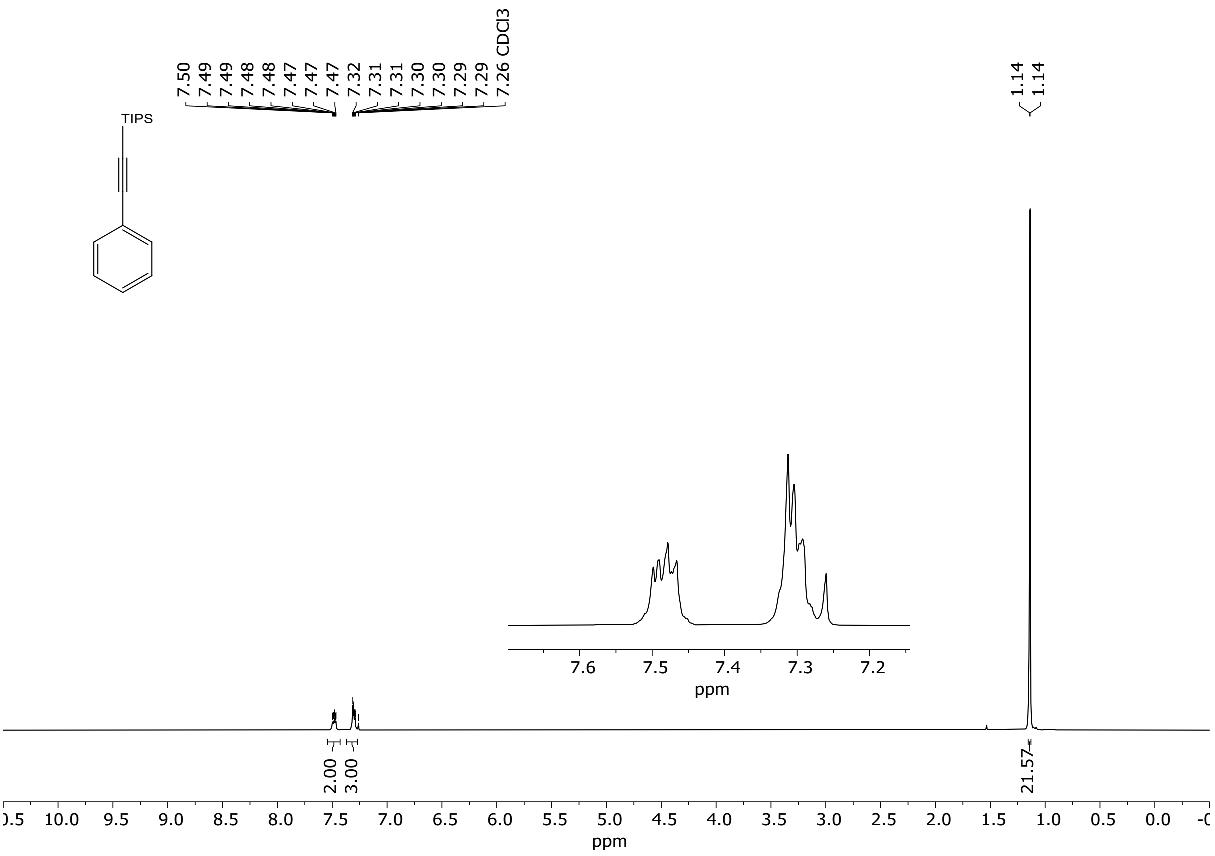

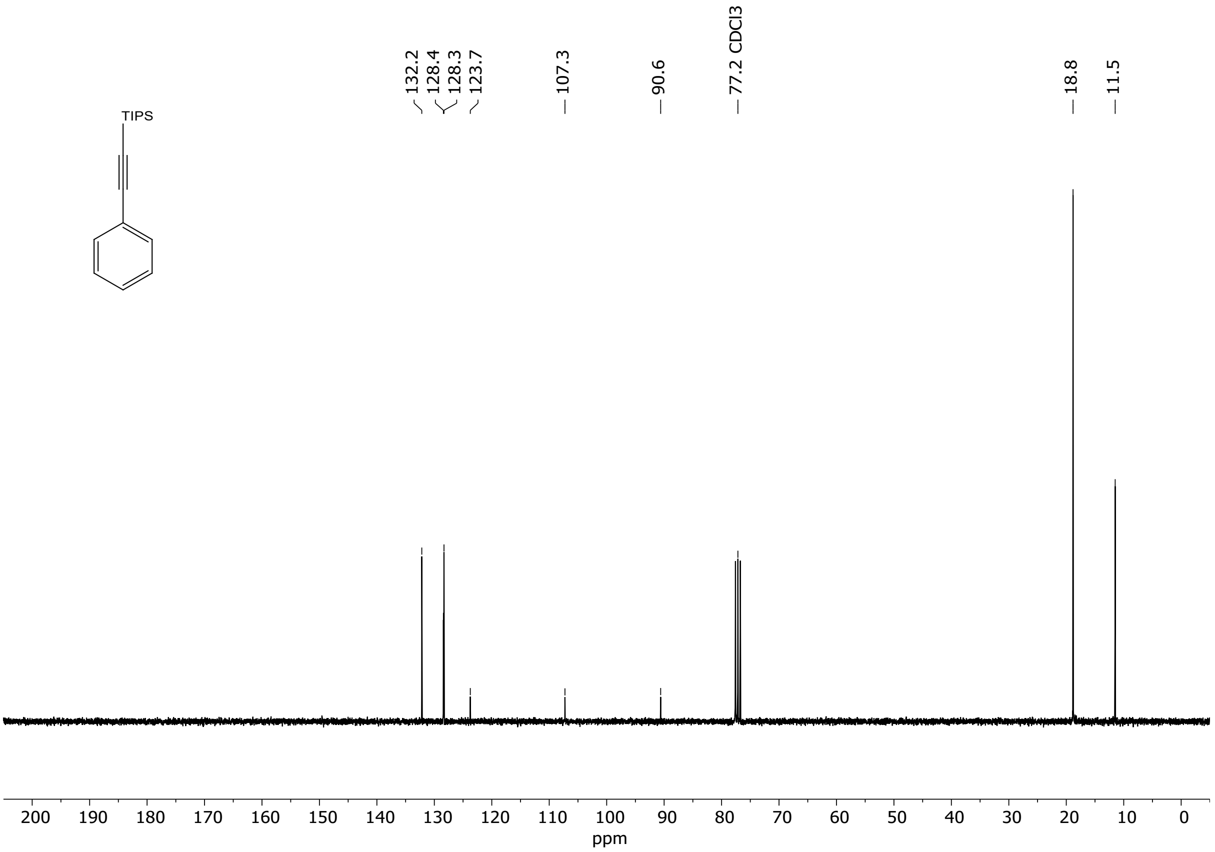

Figure S10. NMR spectra of compound **10** – ^{1}H, $CDCl_3$, 500 MHz (top); ^{13}C, $CDCl_3$, 126 MHz (bottom).

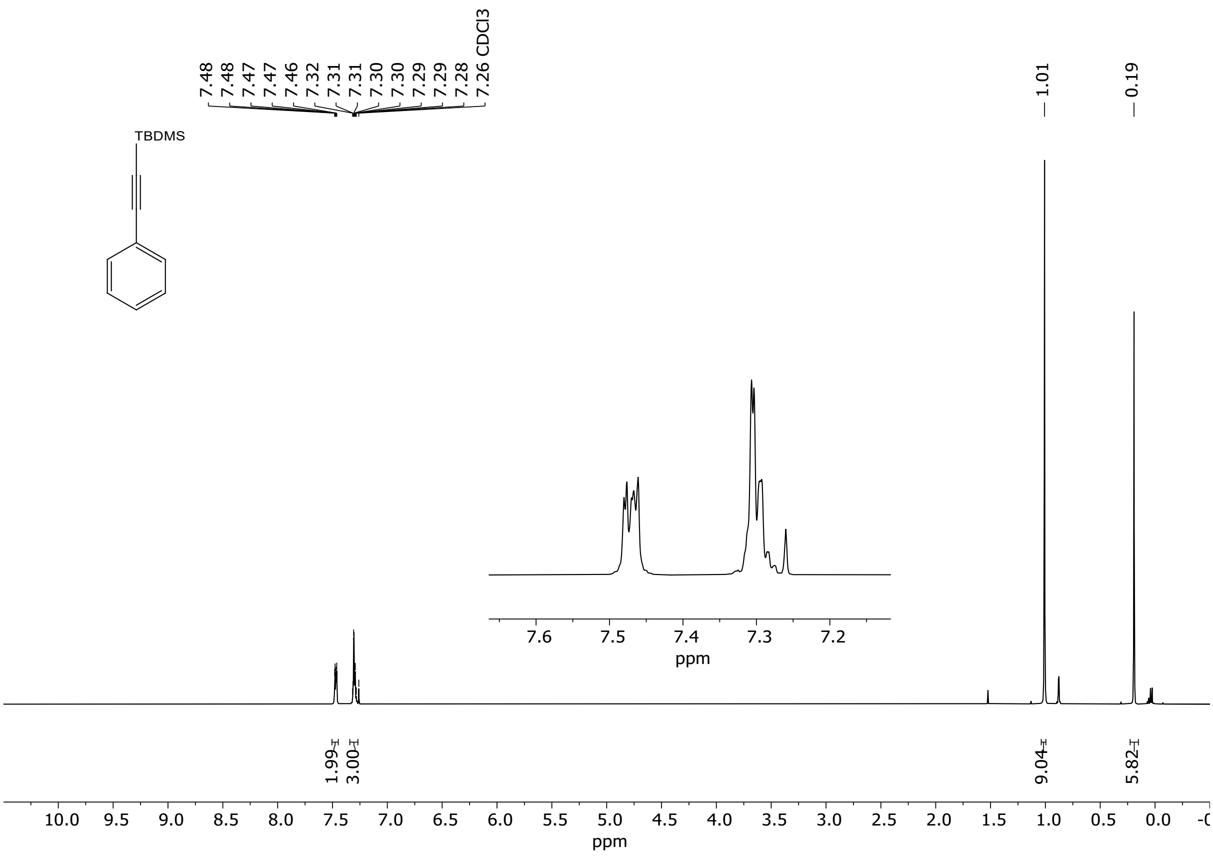

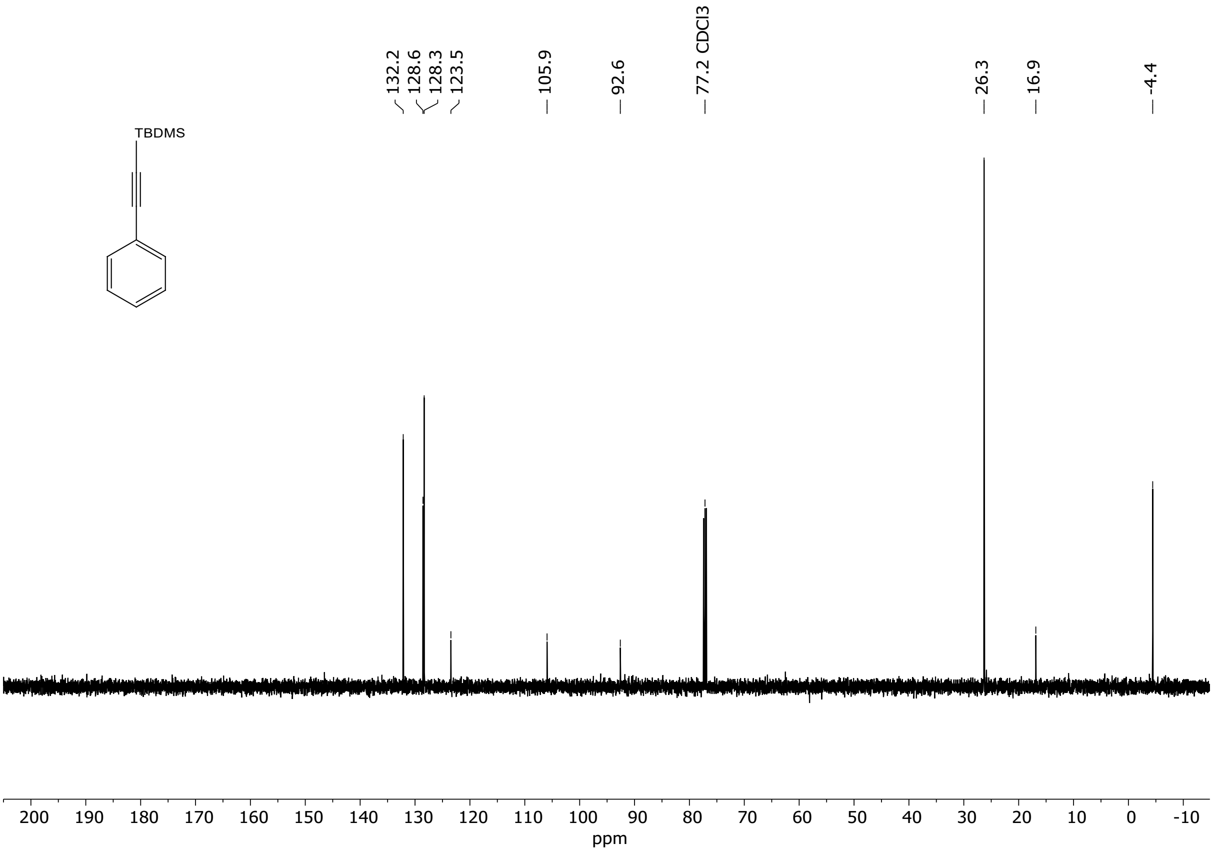

Figure S11. NMR spectra of compound **S27** – ^{1}H, $CDCl_3$, 500 MHz (top); ^{13}C, $CDCl_3$, 126 MHz (bottom).

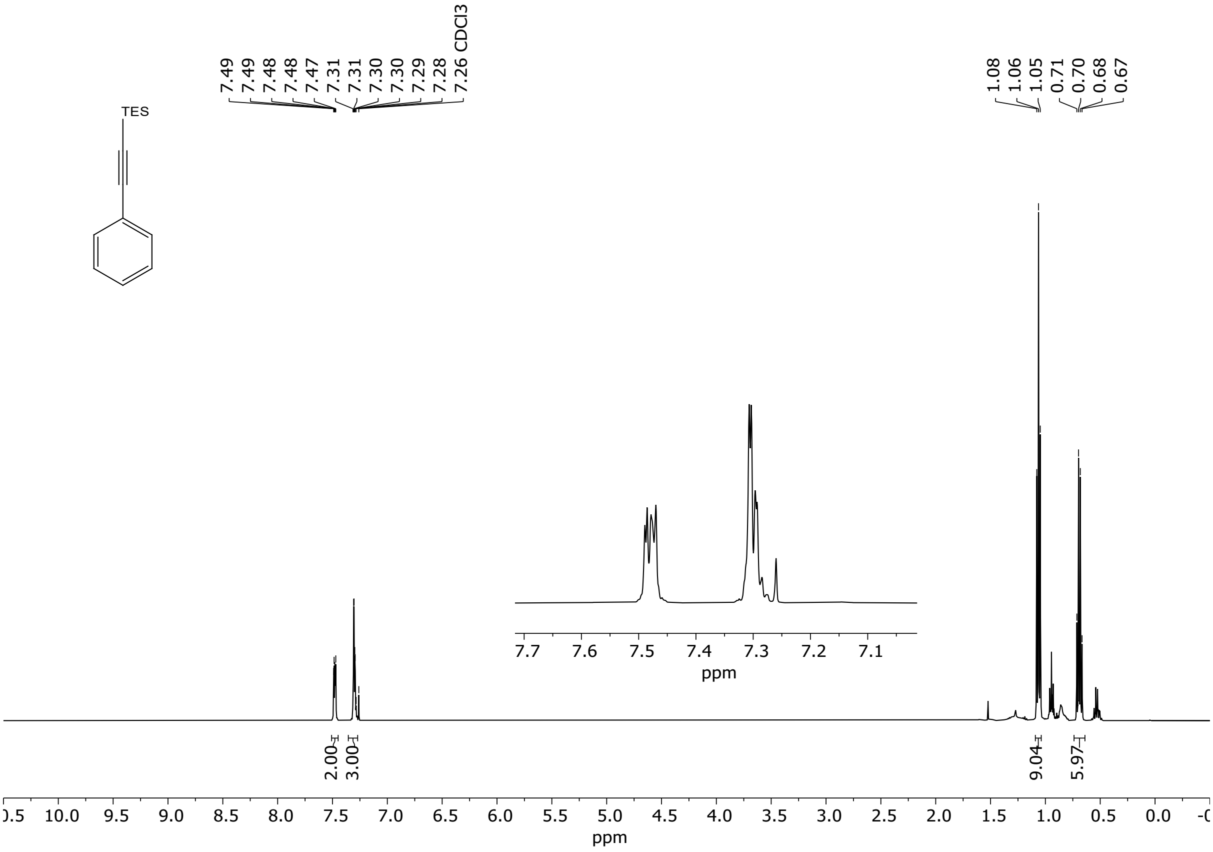

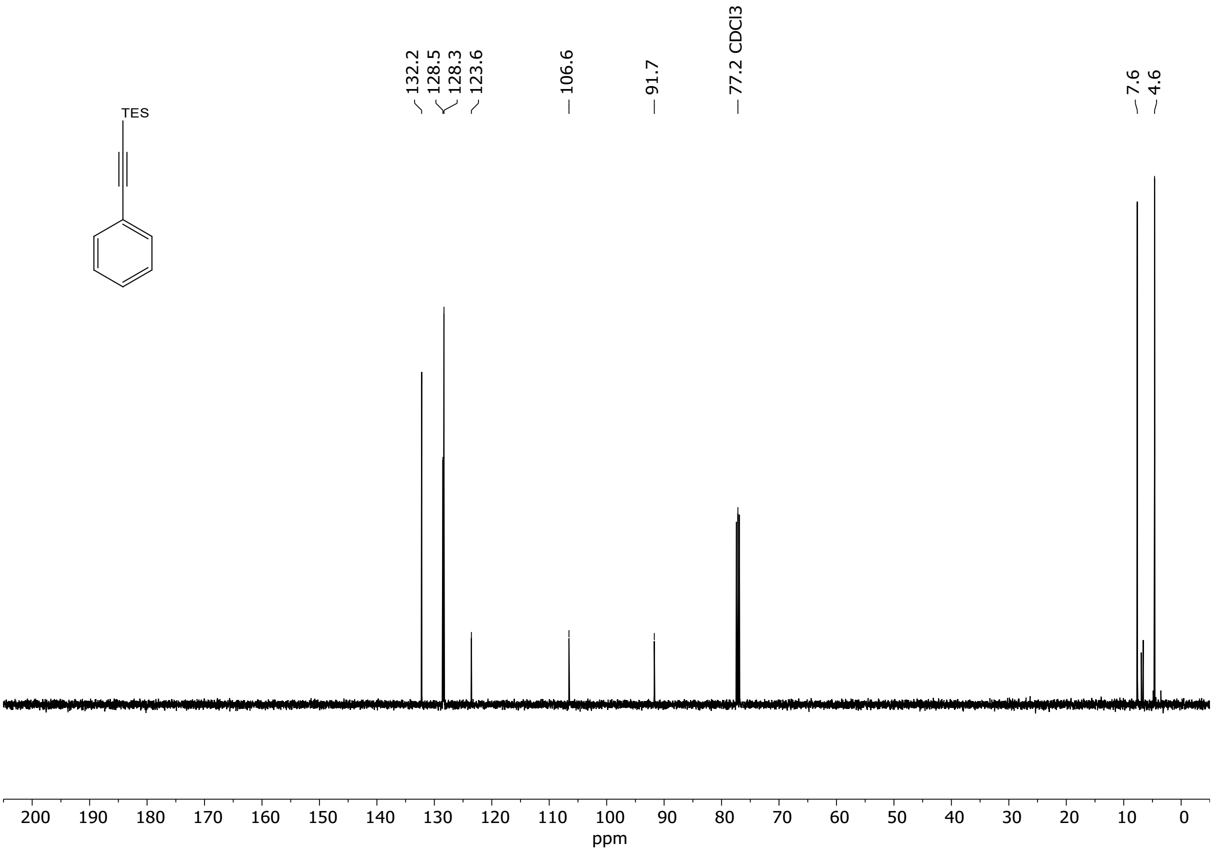

Figure S12. NMR spectra of compound **S28** – ^{1}H, $CDCl_3$, 500 MHz (top); ^{13}C, $CDCl_3$, 126 MHz (bottom).

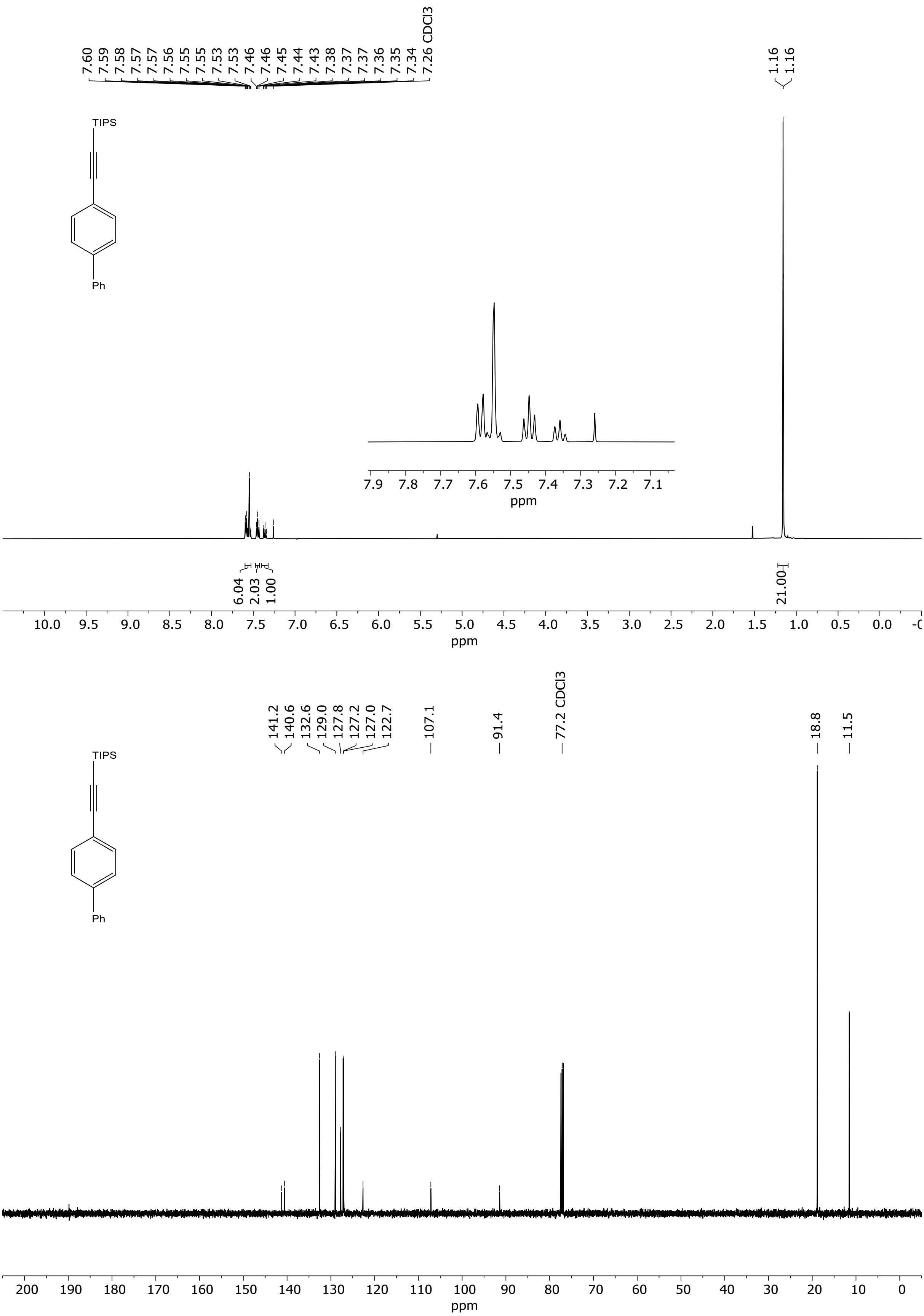

Figure S13. NMR spectra of compound **S29** – ^{1}H, $CDCl_3$, 500 MHz (top); ^{13}C, $CDCl_3$, 126 MHz (bottom).

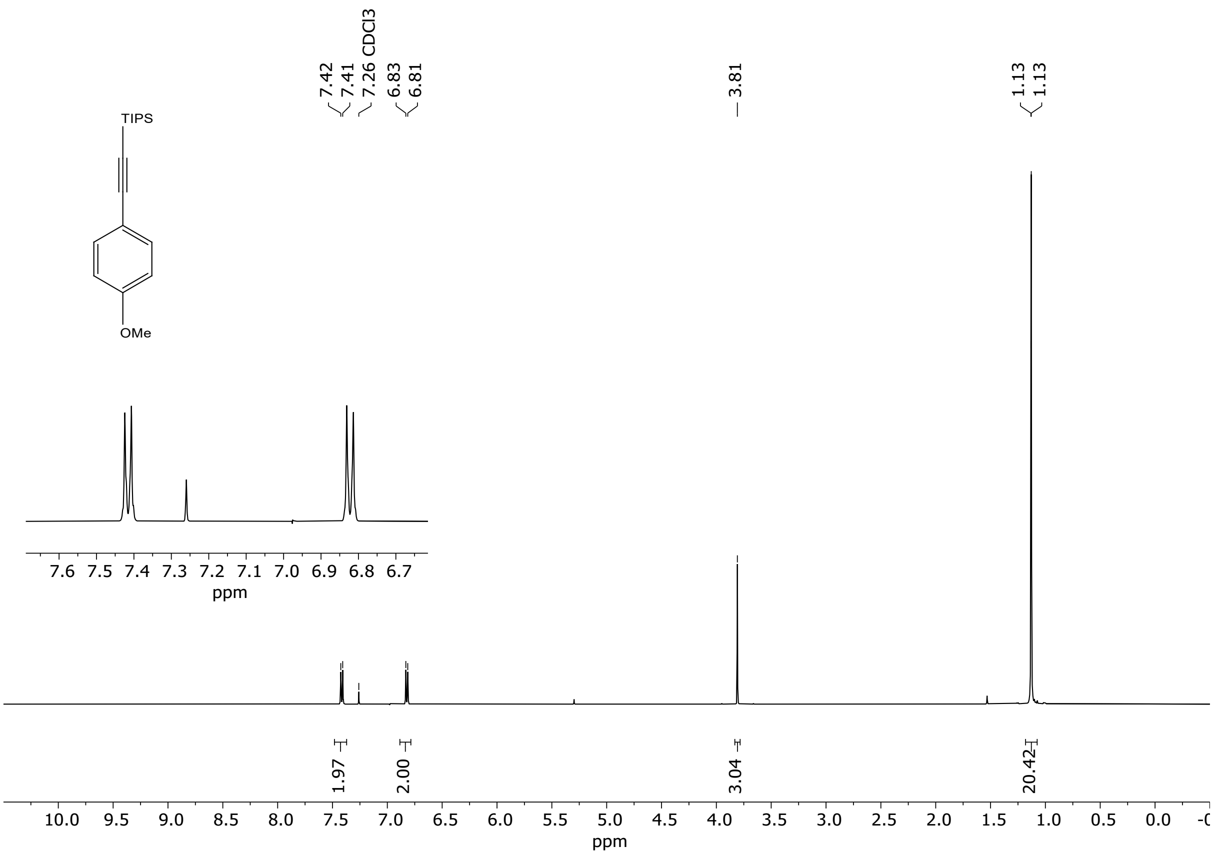

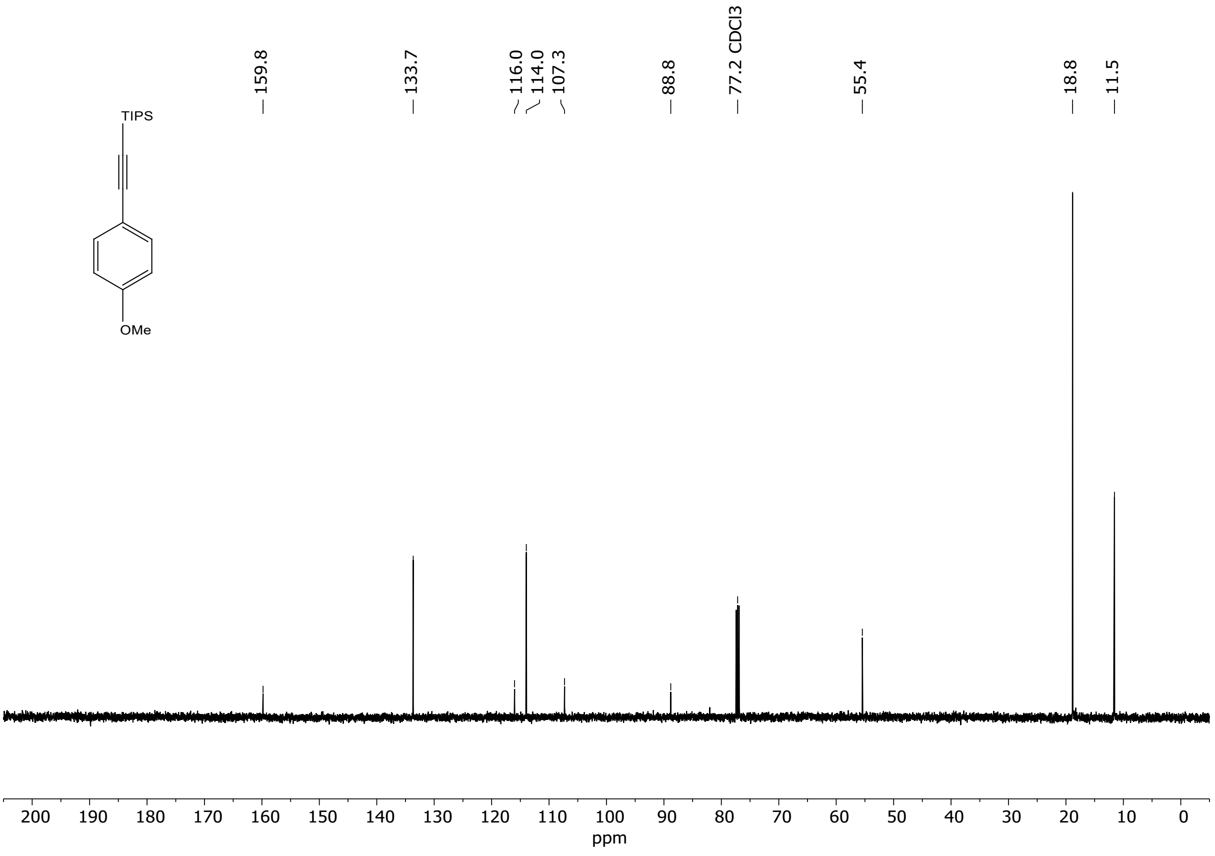

Figure S14. NMR spectra of compound **S30** – ^{1}H, $CDCl_3$, 500 MHz (top); ^{13}C, $CDCl_3$, 126 MHz (bottom).

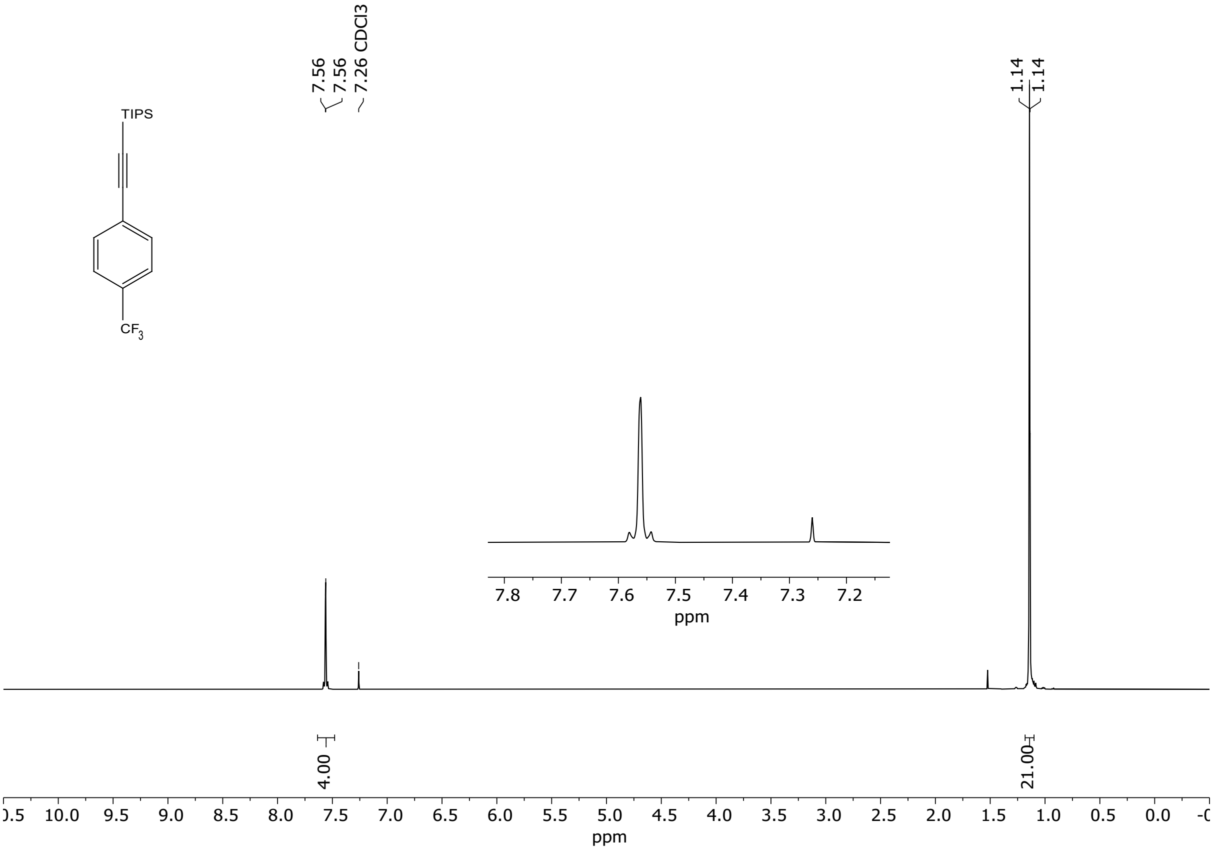

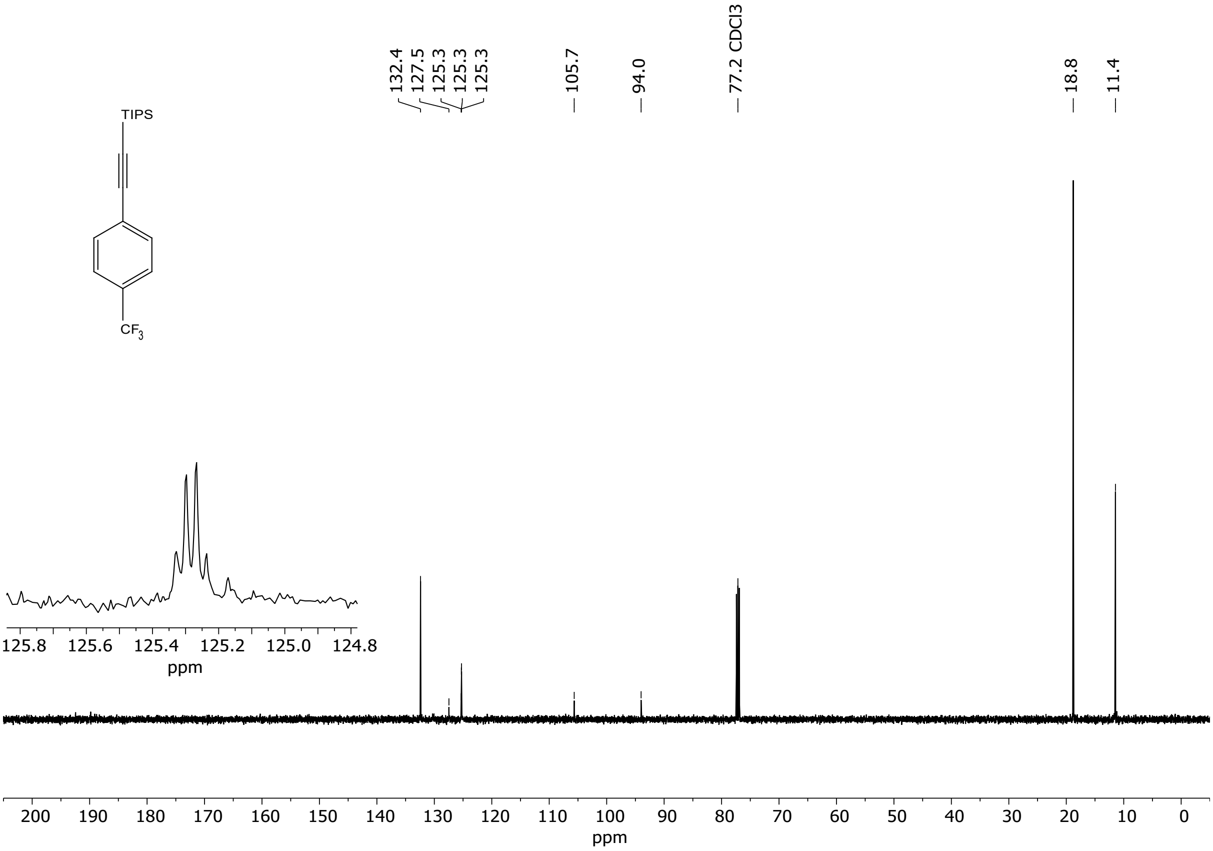

Figure S15. NMR spectra of compound **S31** – ^{1}H, $CDCl_3$, 500 MHz (top); ^{13}C, $CDCl_3$, 126 MHz (bottom).

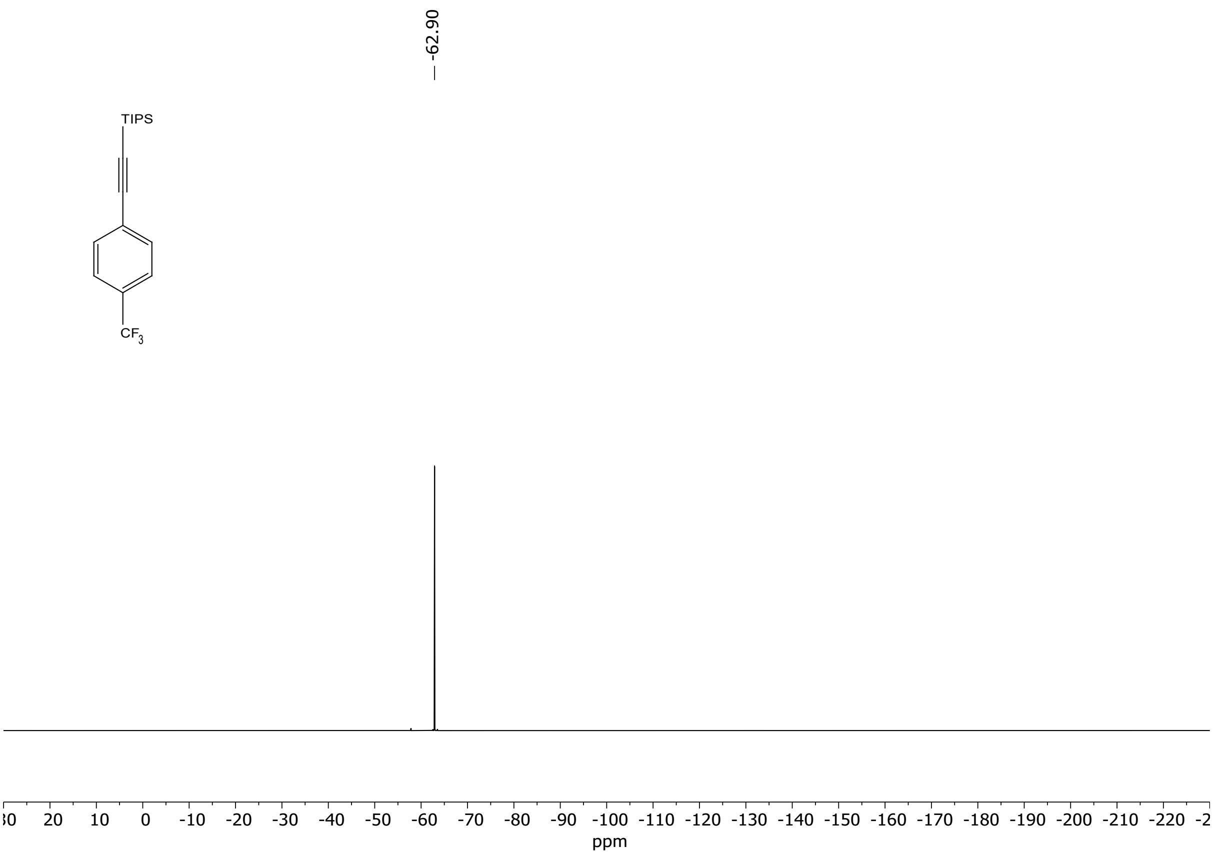

Figure S16. NMR spectrum of compound **S31** – ^{19}F, $CDCl_3$, 282 MHz.

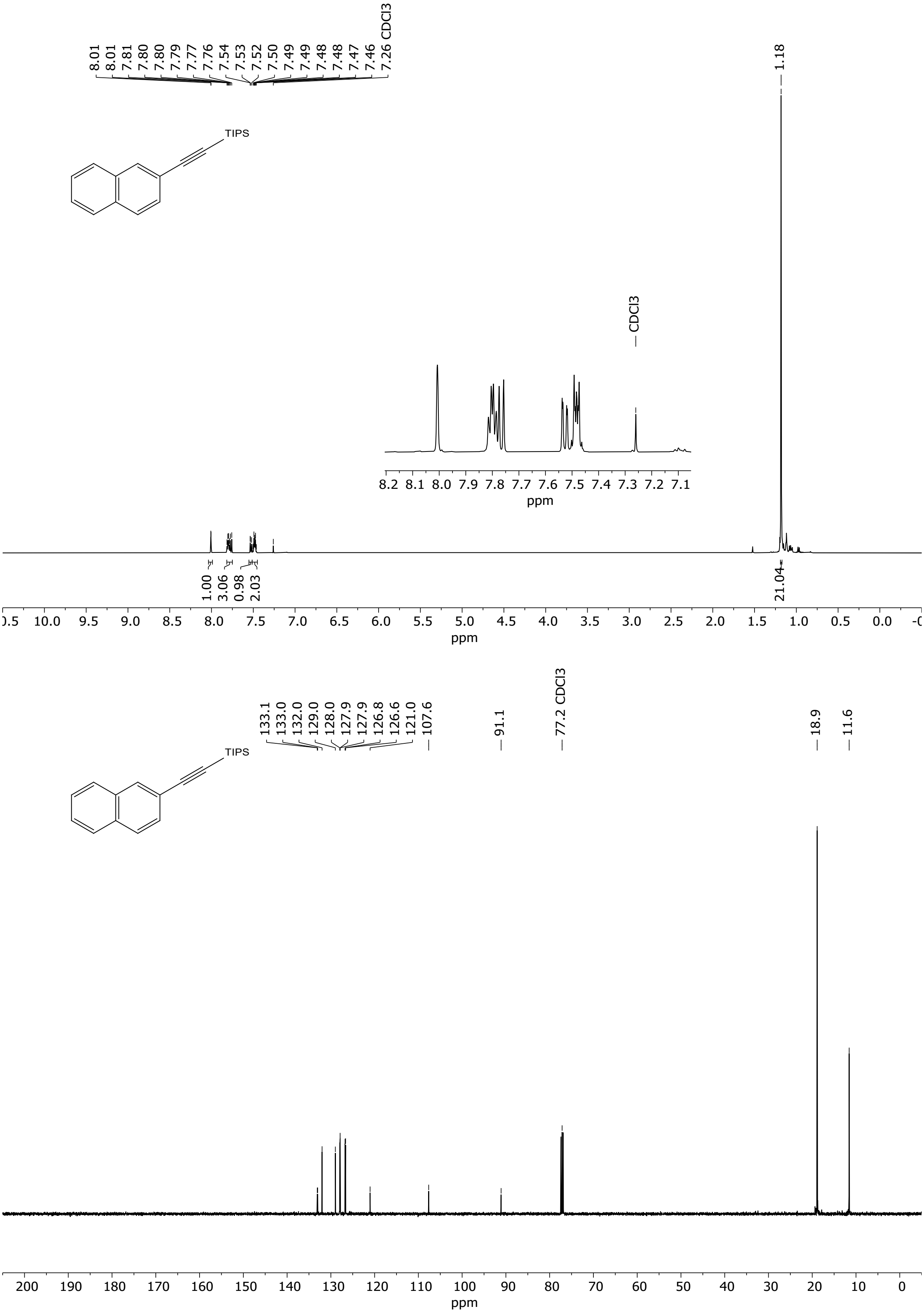

Figure S17. NMR spectra of compound **S32** – ^{1}H, $CDCl_3$, 500 MHz (top); ^{13}C, $CDCl_3$, 126 MHz (bottom).

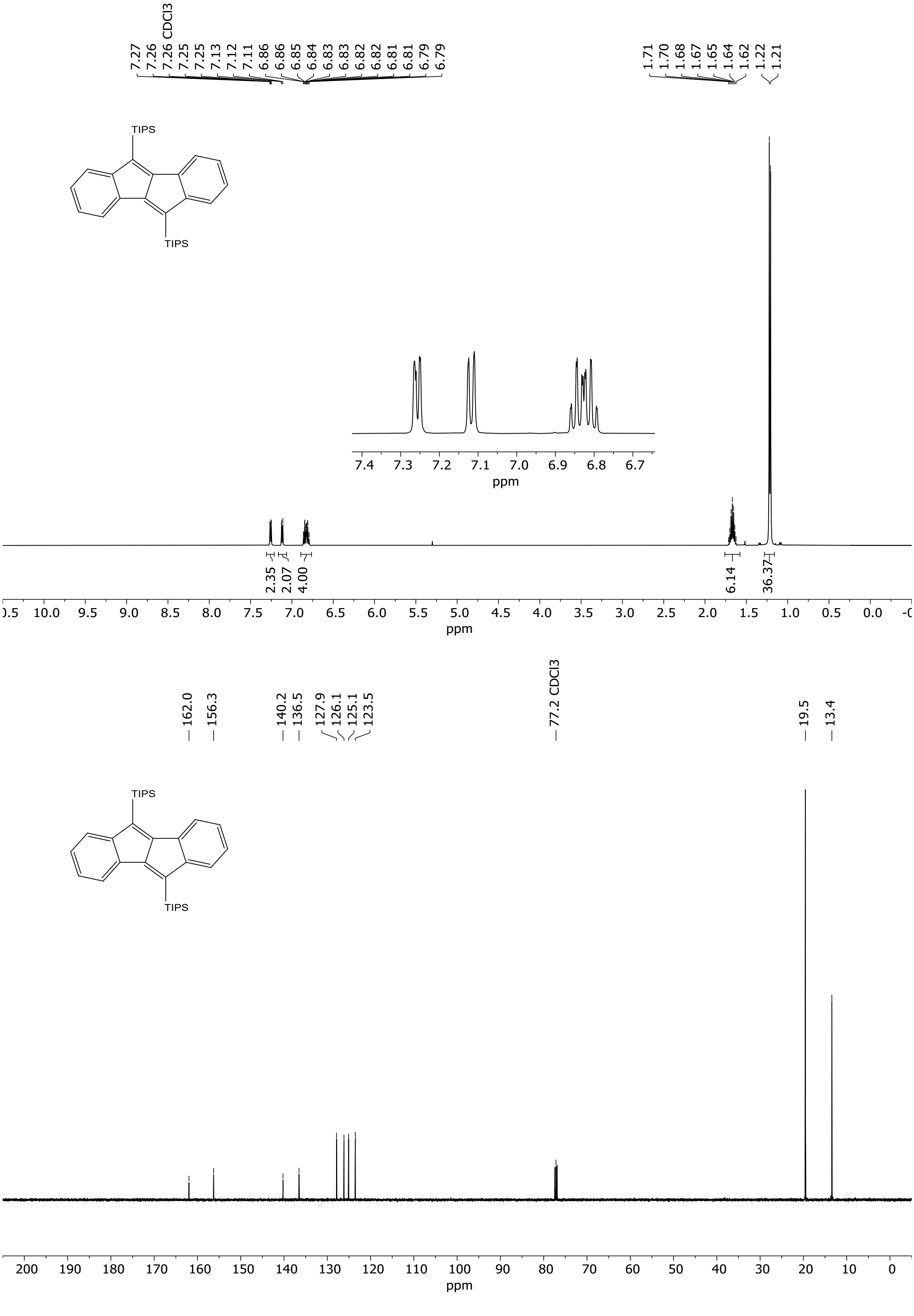

Figure S18. NMR spectra of compound **11** – ^{1}H, $CDCl_3$, 500 MHz (top); ^{13}C, $CDCl_3$, 126 MHz (bottom).

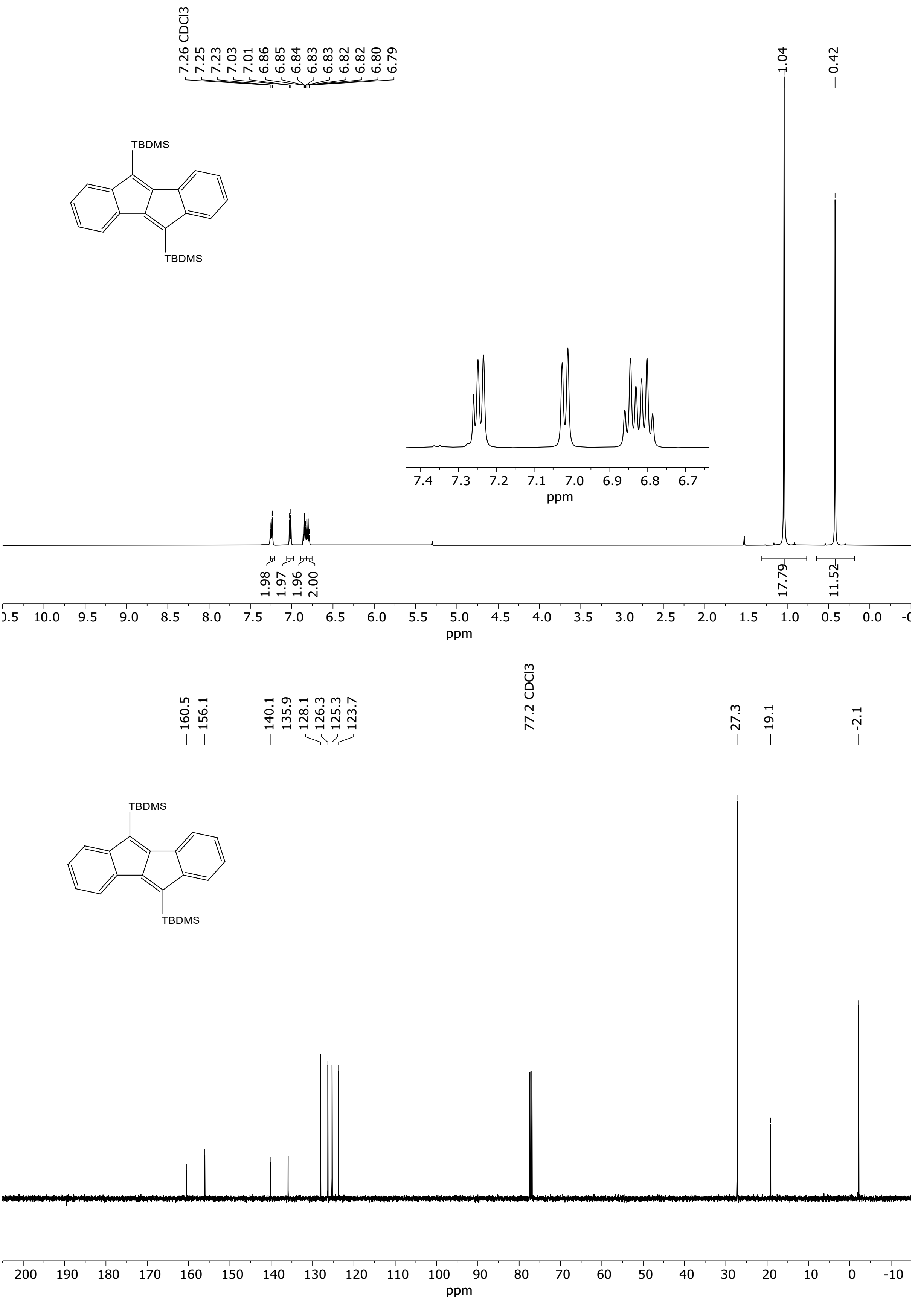

Figure S19. NMR spectra of compound **S33** – ^{1}H, $CDCl_3$, 500 MHz (top); ^{13}C, $CDCl_3$, 126 MHz (bottom).

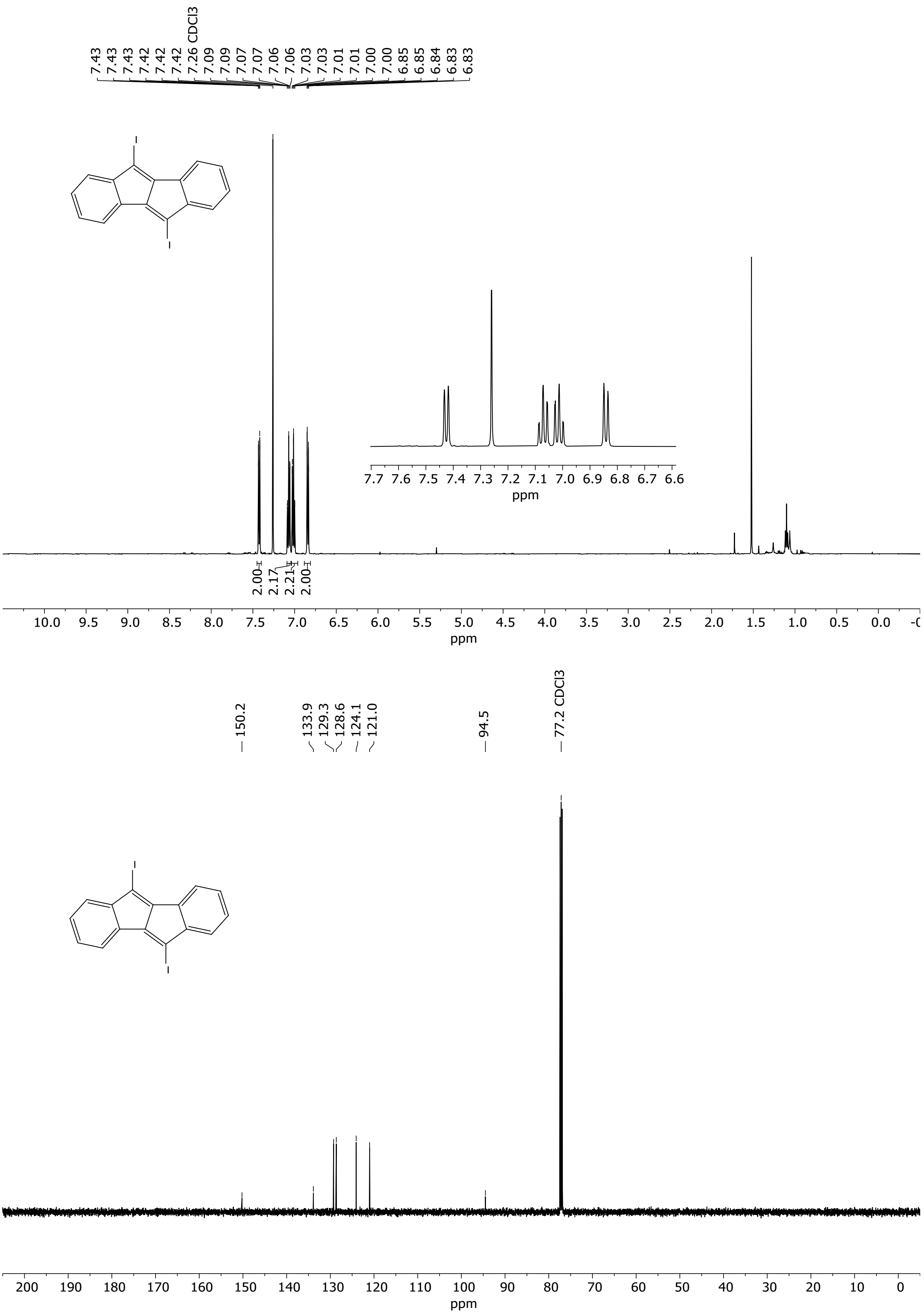

Figure S20. NMR spectra of compound **13** – ^{1}H, $CDCl_3$, 500 MHz (top); ^{13}C, $CDCl_3$, 126 MHz (bottom).

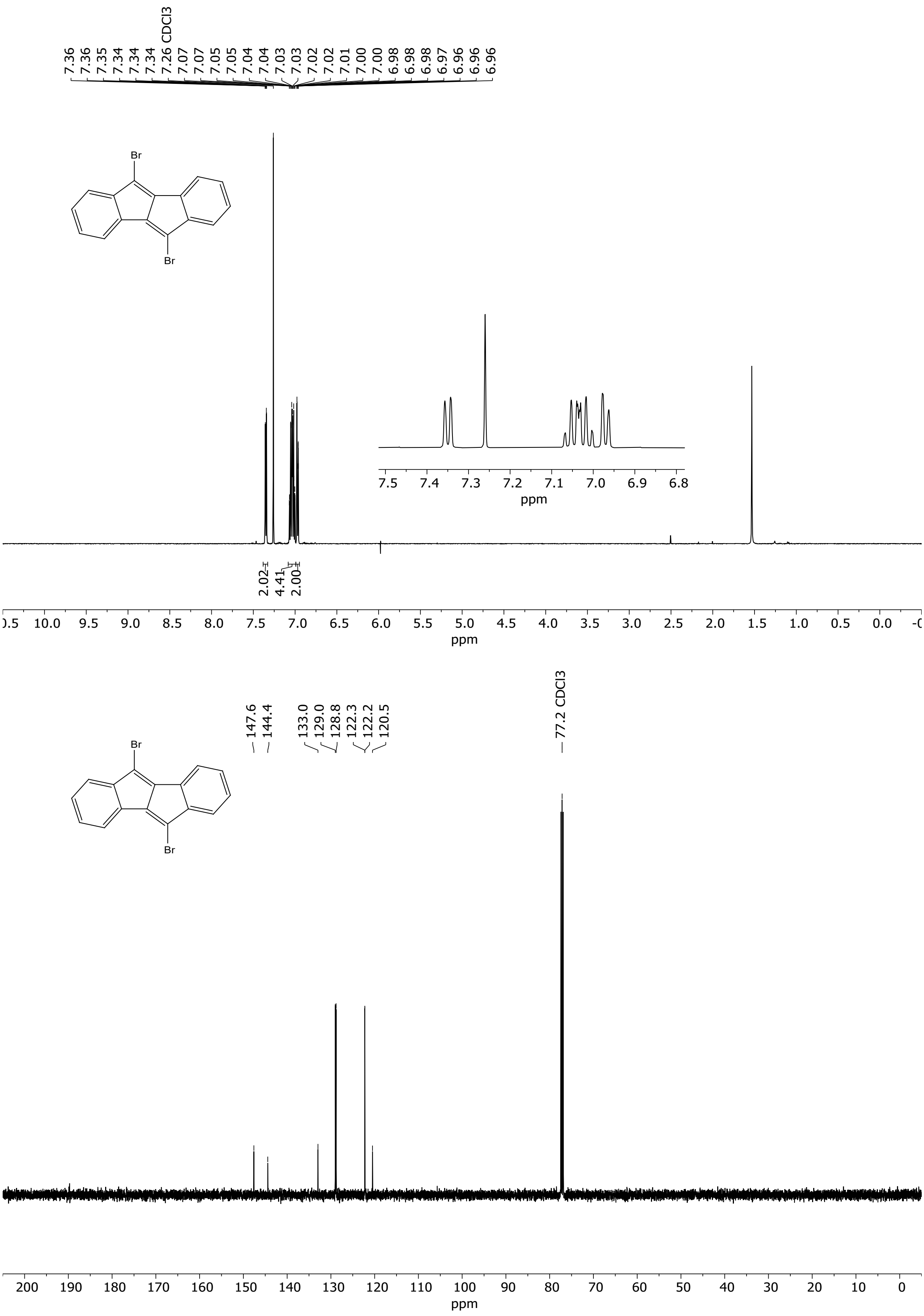

Figure S21. NMR spectra of compound **12** – ^{1}H, $CDCl_3$, 500 MHz (top); ^{13}C, $CDCl_3$, 126 MHz (bottom).

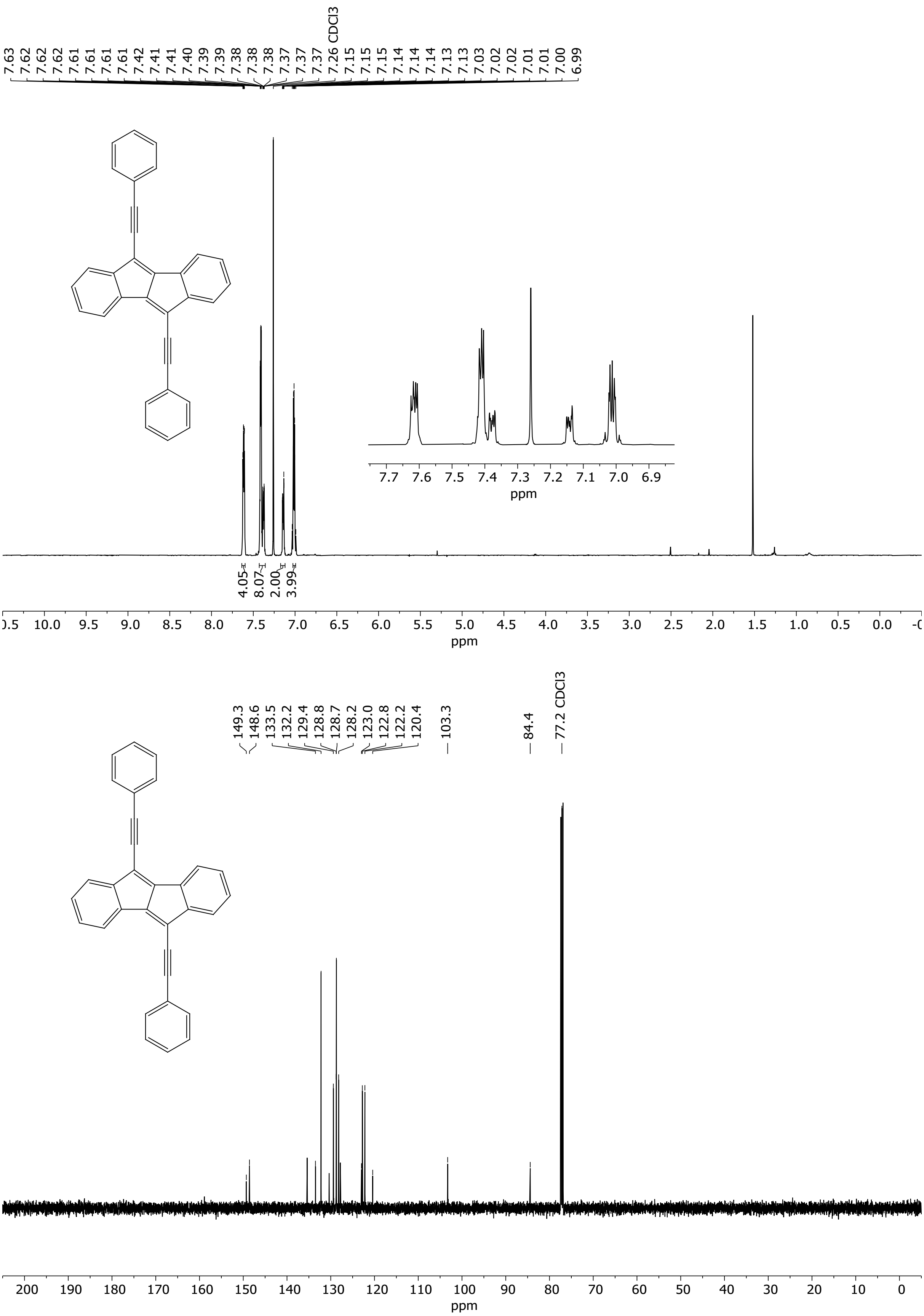

Figure S22. NMR spectra of compound **14** – ^{1}H, $CDCl_3$, 500 MHz (top); ^{13}C, $CDCl_3$, 126 MHz (bottom).

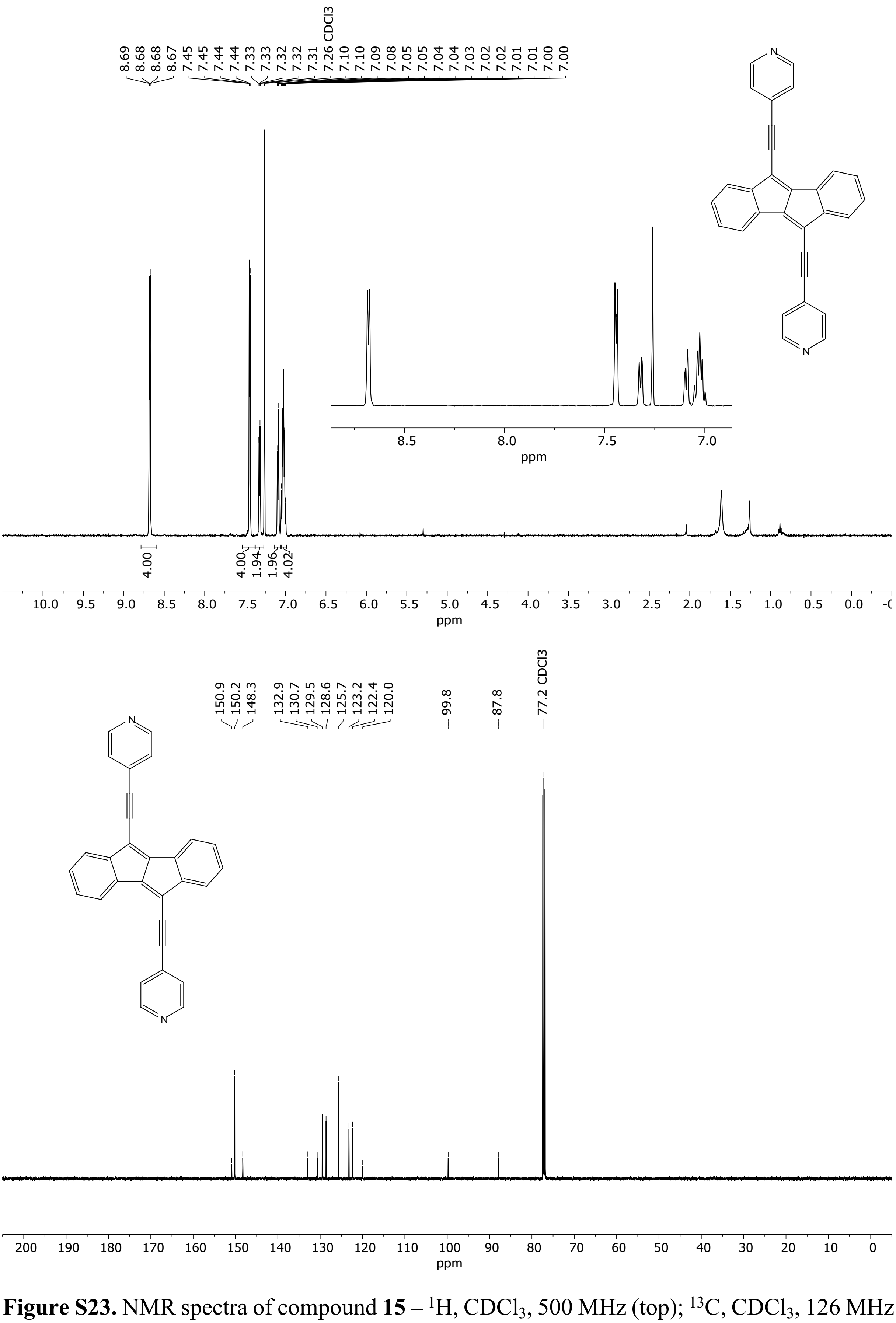

Figure S23. NMR spectra of compound **15** – ^{1}H, $CDCl_3$, 500 MHz (top); ^{13}C, $CDCl_3$, 126 MHz (bottom).

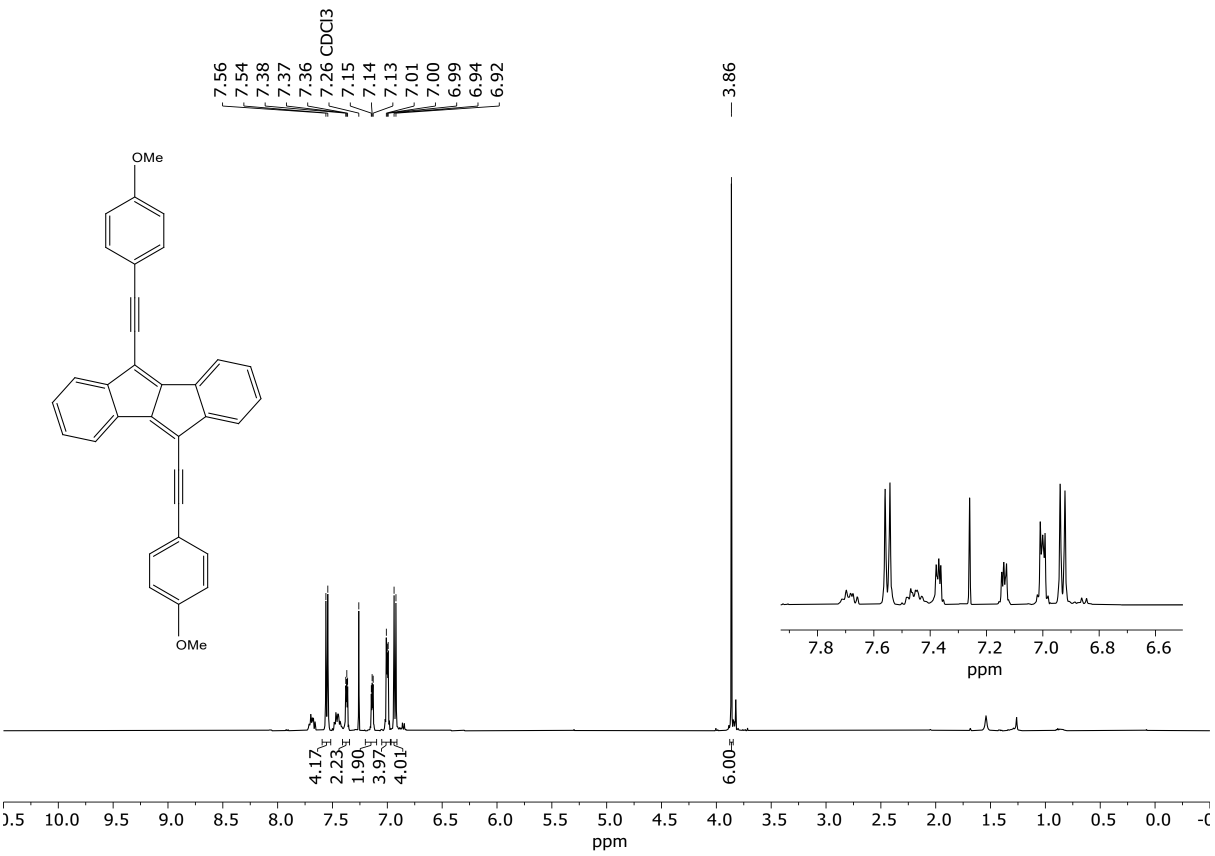

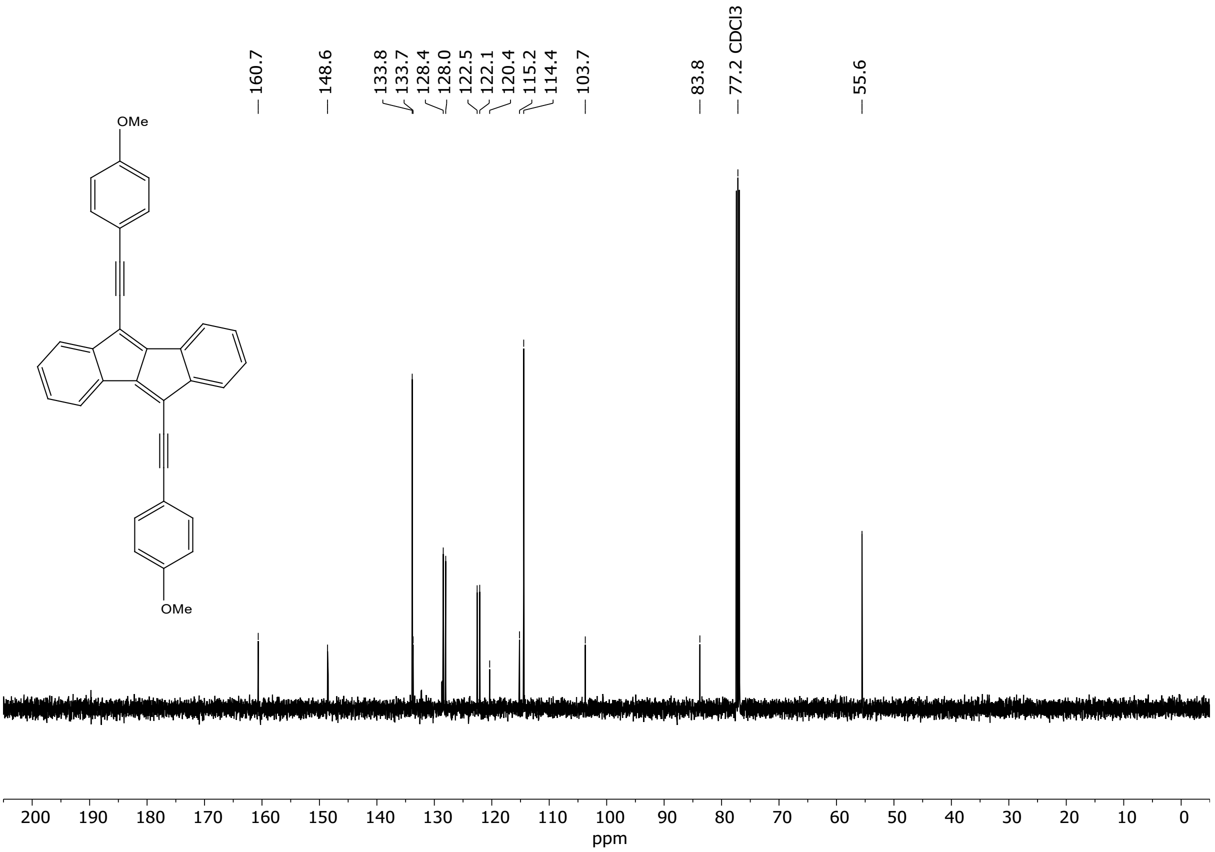

Figure S24. NMR spectra of compound **16** – ^{1}H, $CDCl_3$, 500 MHz (top); ^{13}C, $CDCl_3$, 126 MHz (bottom).

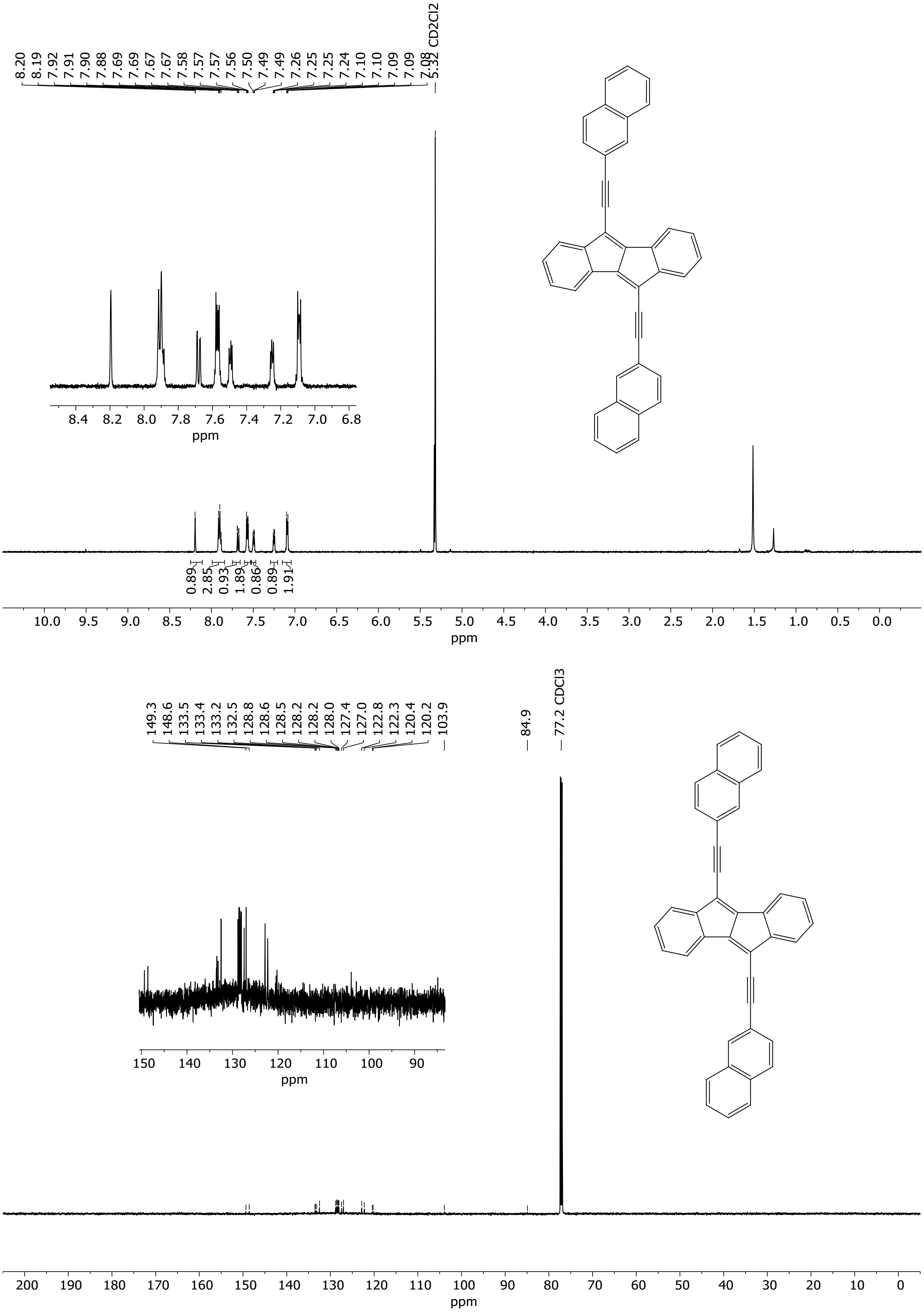

Figure S25. NMR spectra of compound **17** – ^{1}H, $CDCl_3$, 500 MHz (top); ^{13}C, $CDCl_3$, 126 MHz (bottom).

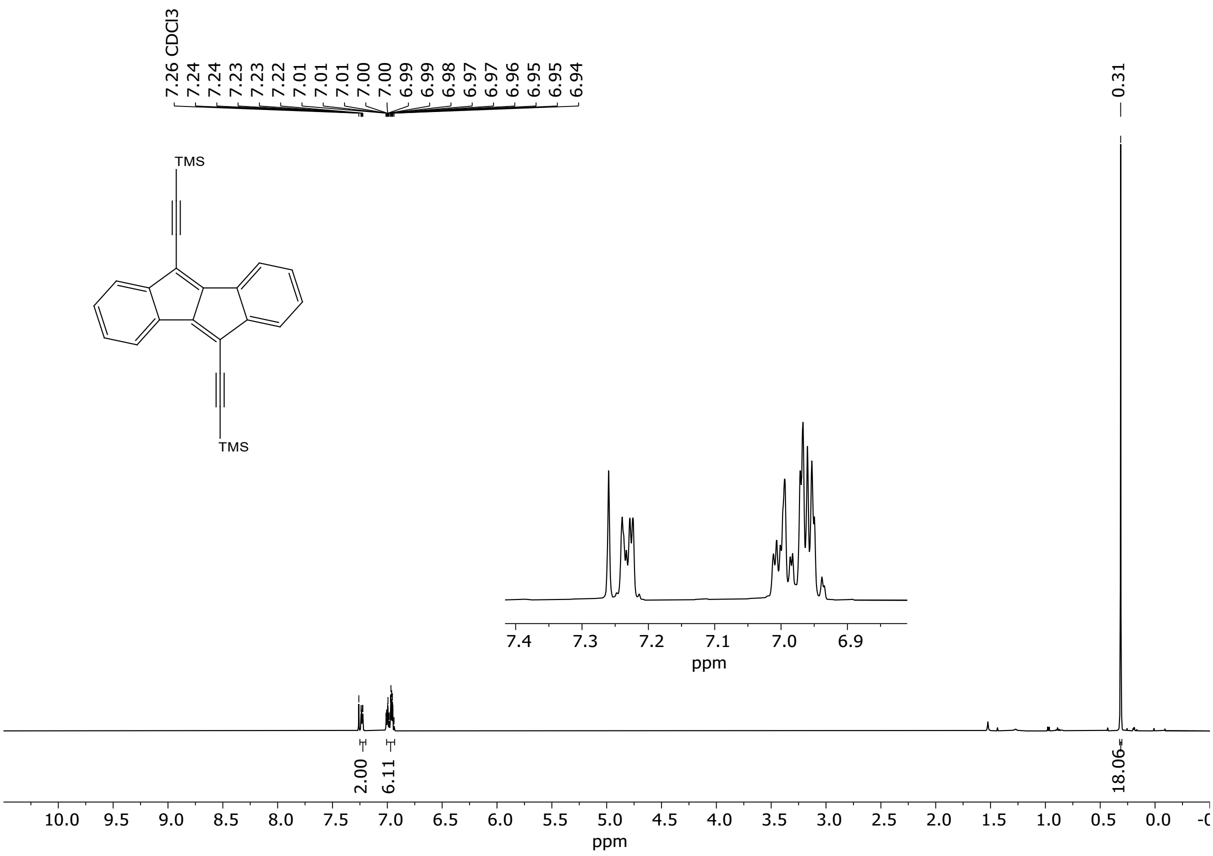

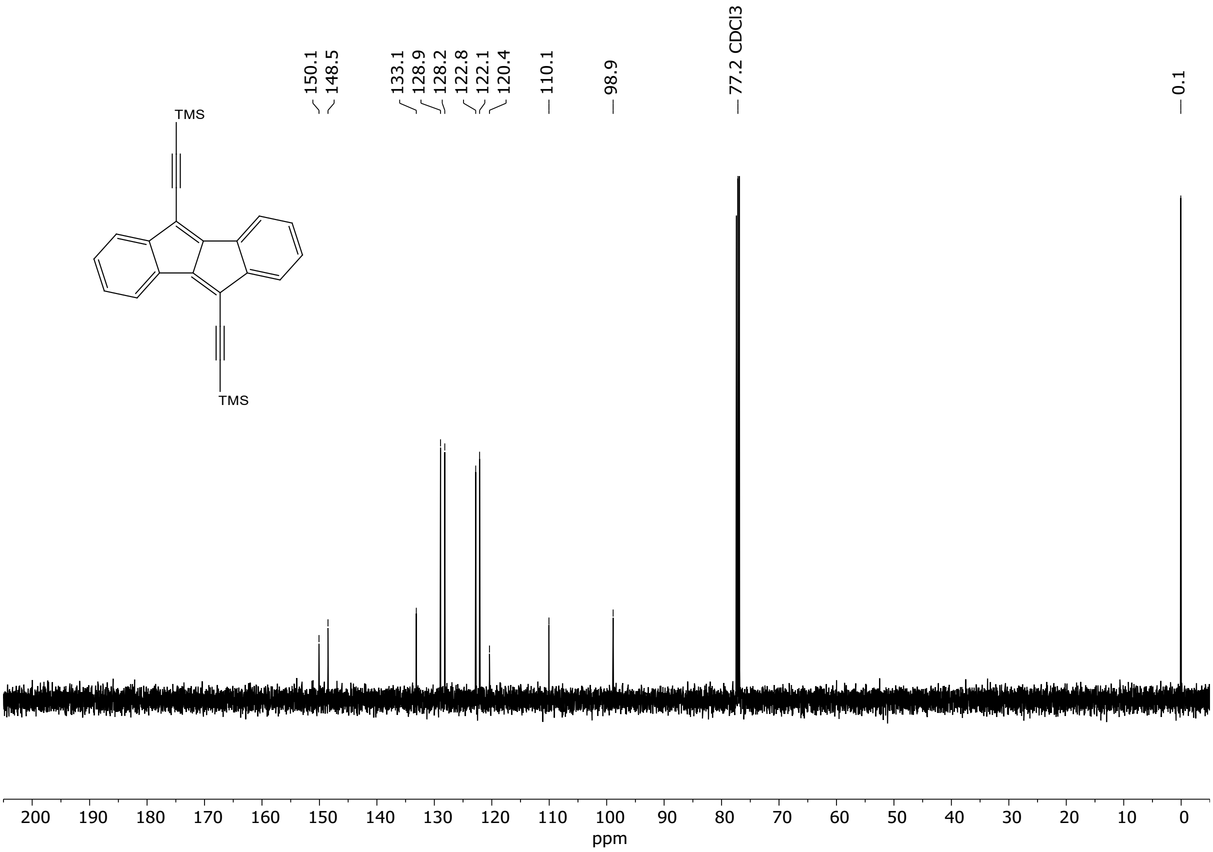

Figure S26. NMR spectra of compound **18** – ^{1}H, $CDCl_3$, 500 MHz (top); ^{13}C, $CDCl_3$, 126 MHz (bottom).

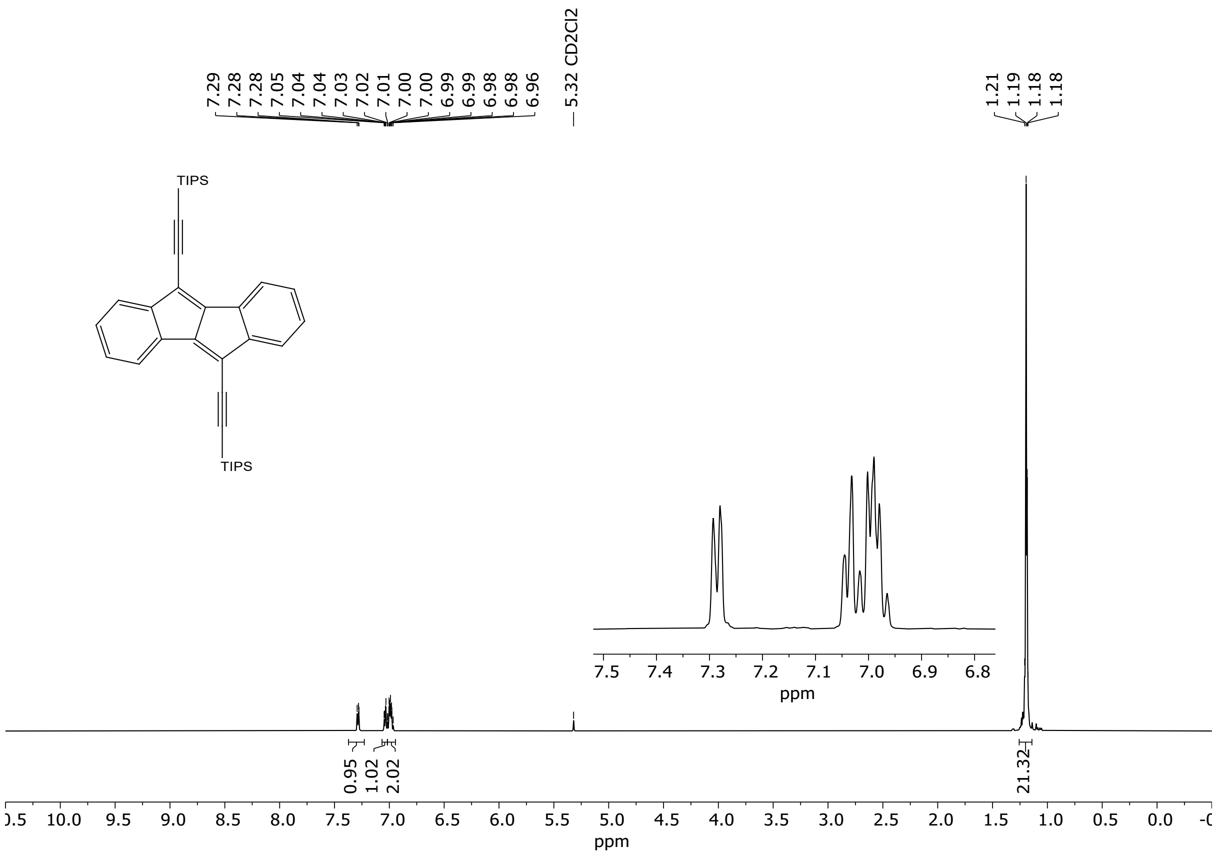

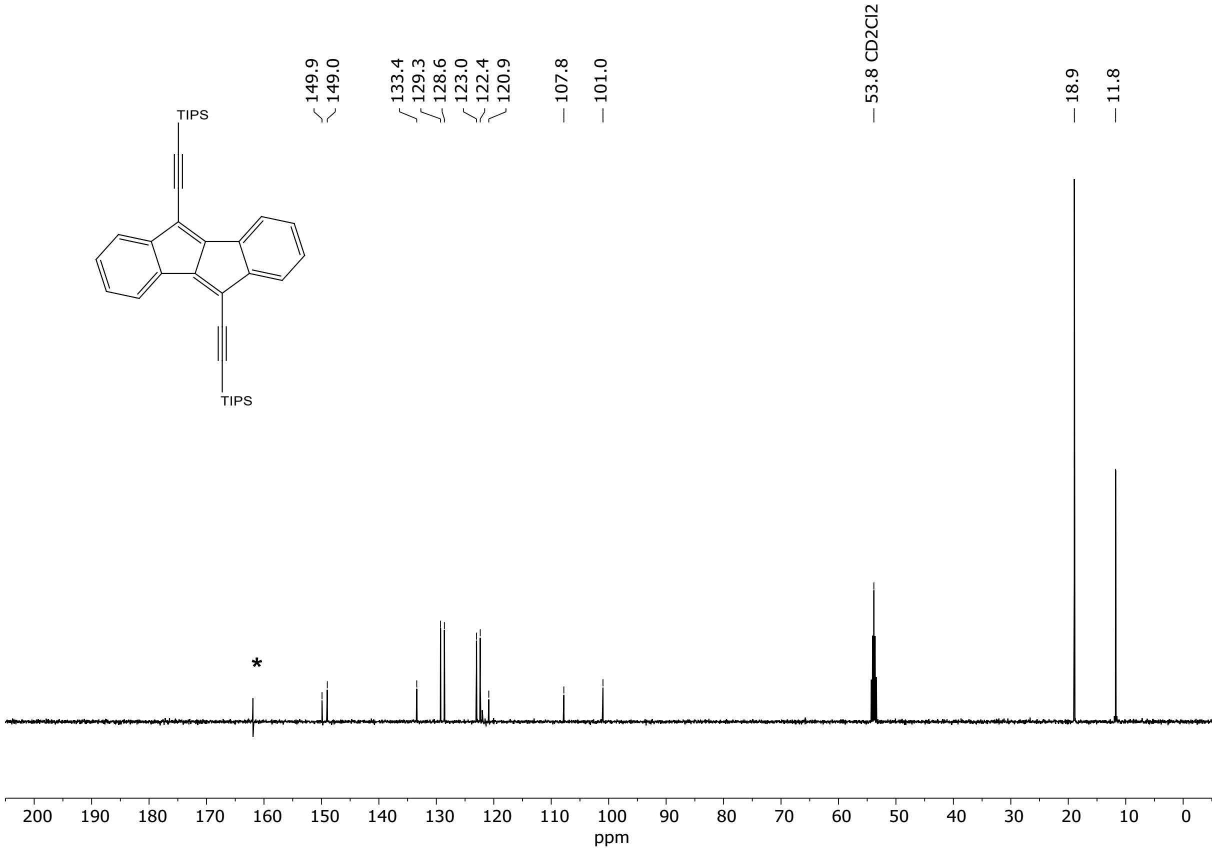

Figure S27. NMR spectra of compound **19** – ^{1}H, CD_2Cl_2, 500 MHz (top); ^{13}C, CD_2Cl_2, 126 MHz (bottom). The asterisk marks a noise from the instrument.

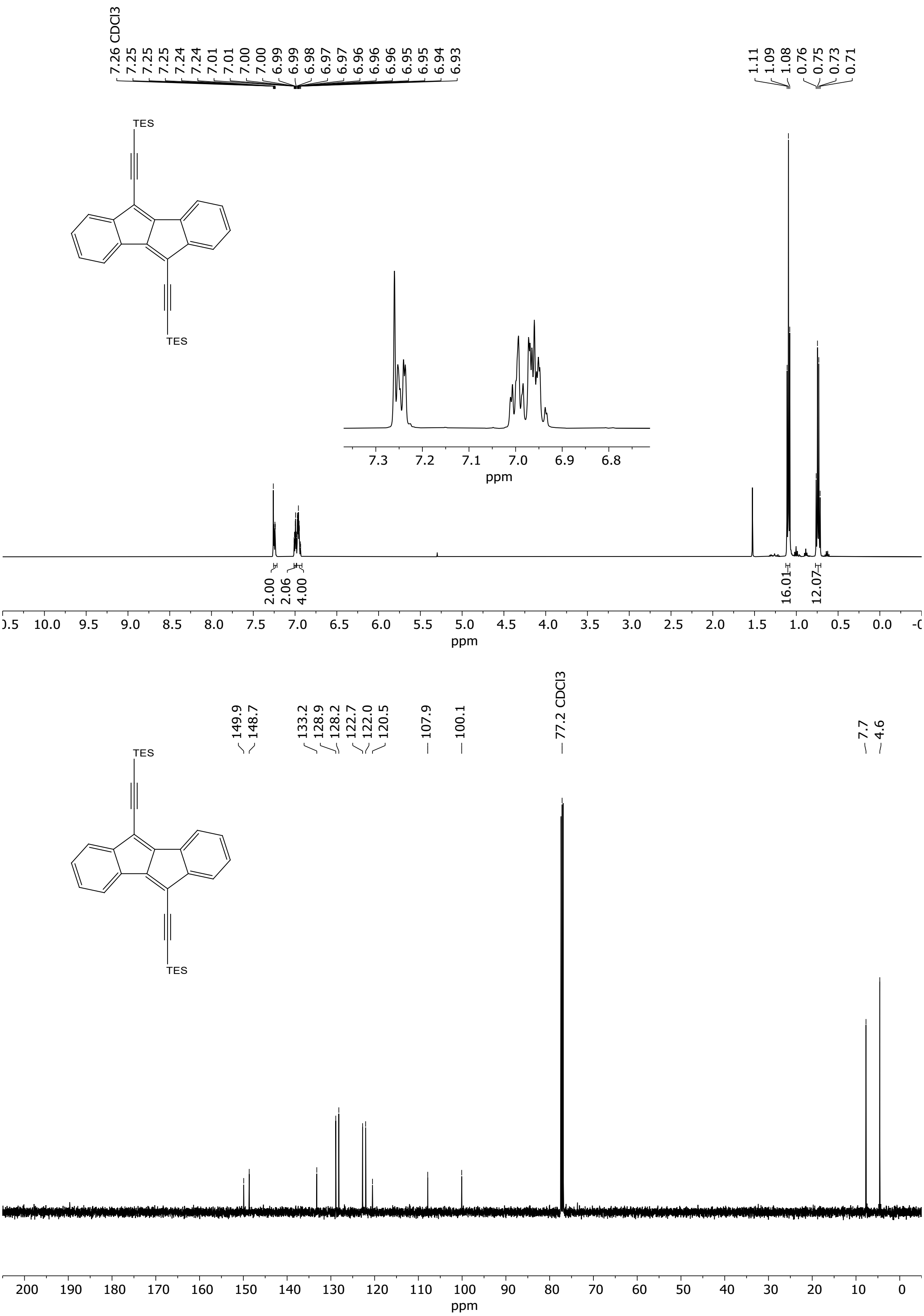

Figure S28. NMR spectra of compound **20** – ^{1}H, $CDCl_3$, 500 MHz (top); ^{13}C, $CDCl_3$, 126 MHz (bottom).

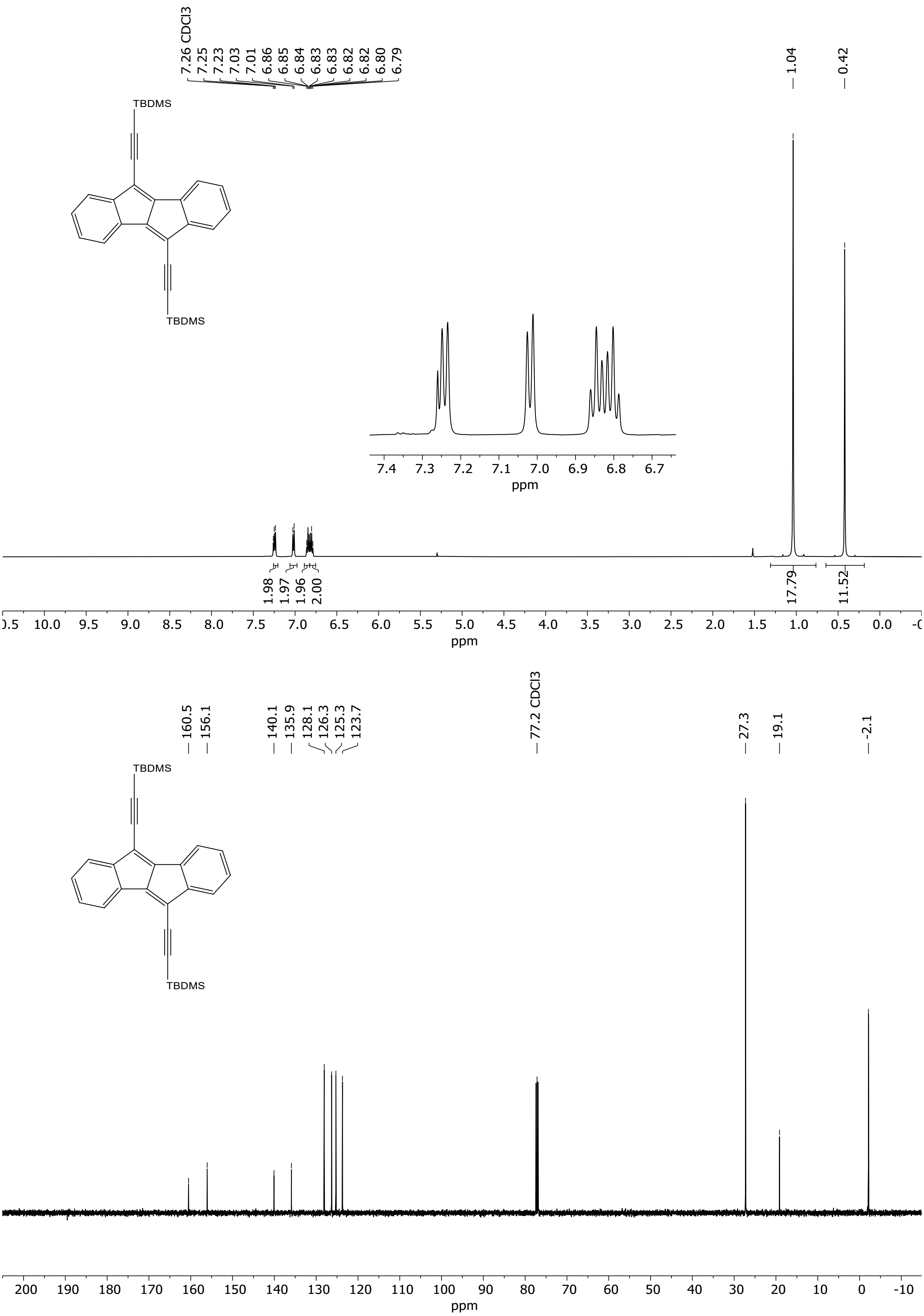

Figure S29. NMR spectra of compound **21** – ^{1}H, $CDCl_3$, 500 MHz (top); ^{13}C, $CDCl_3$, 126 MHz (bottom).

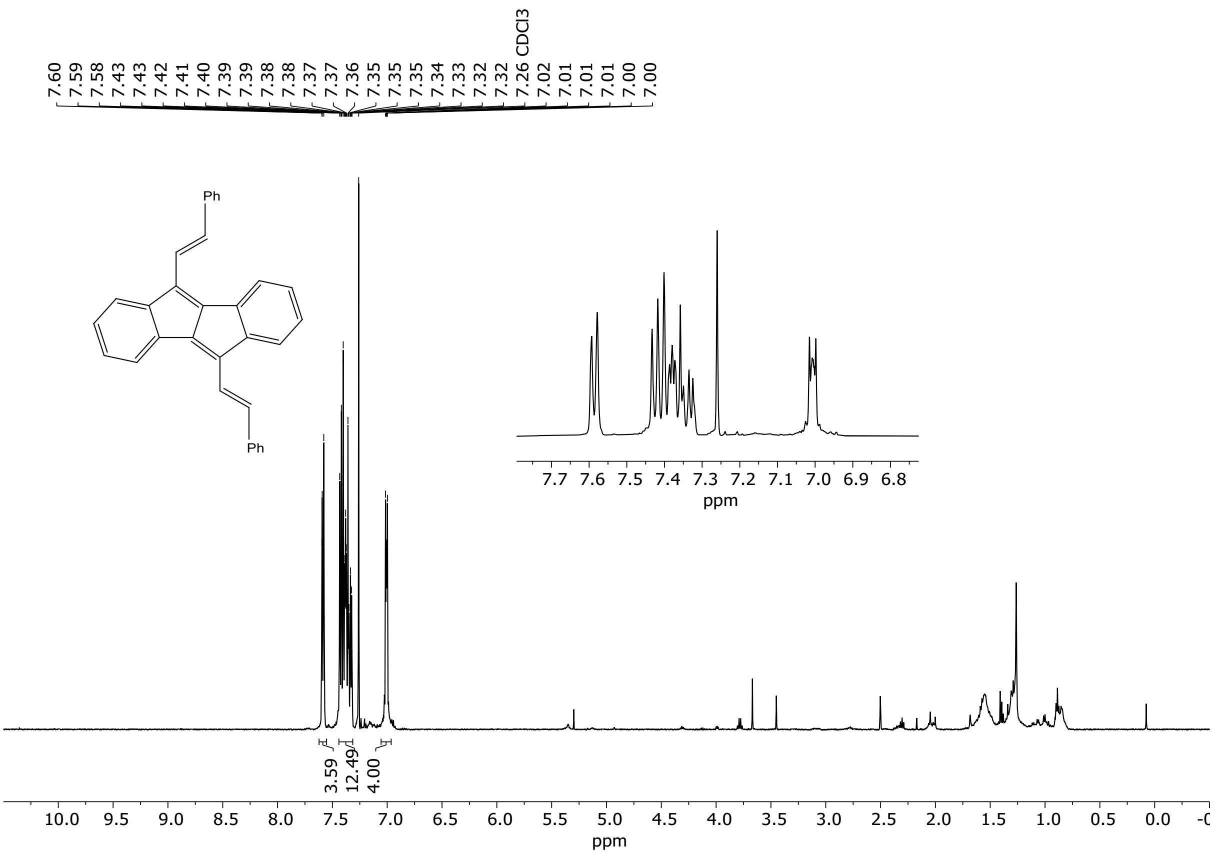

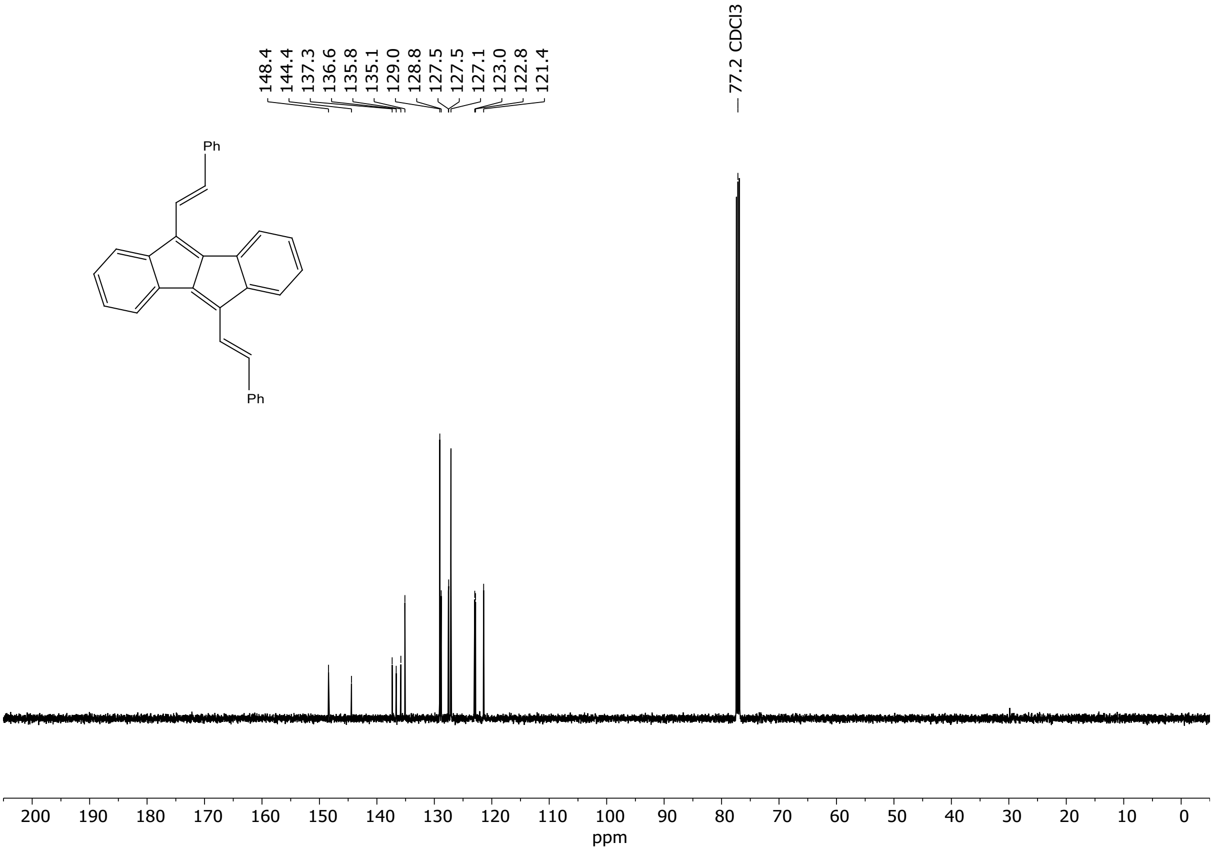

Figure S30. NMR spectra of compound **22** – ^{1}H, $CDCl_3$, 500 MHz; ^{13}C, $CDCl_3$, 126 MHz (bottom).

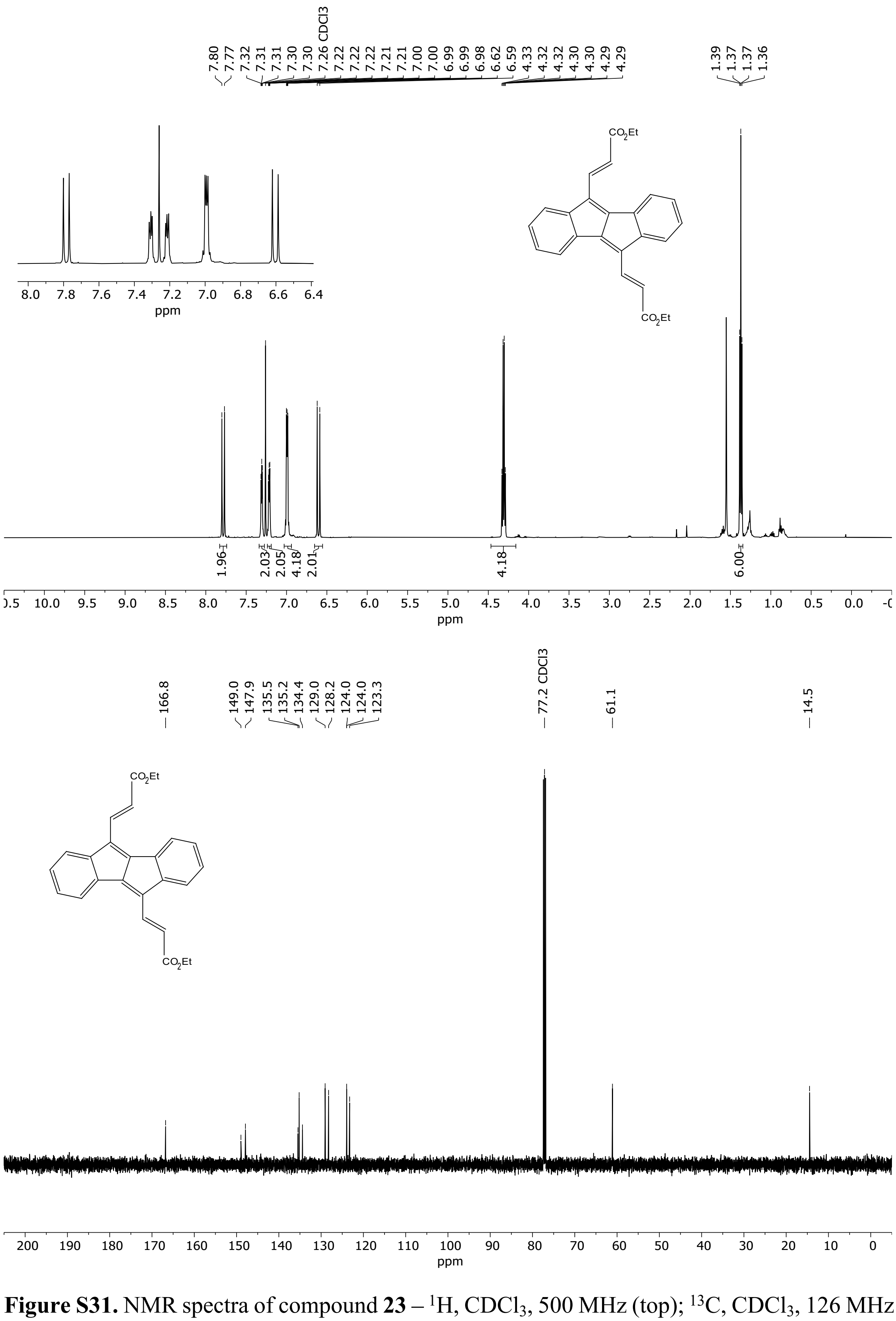

Figure S31. NMR spectra of compound **23** – ^{1}H, $CDCl_3$, 500 MHz (top); ^{13}C, $CDCl_3$, 126 MHz (bottom).

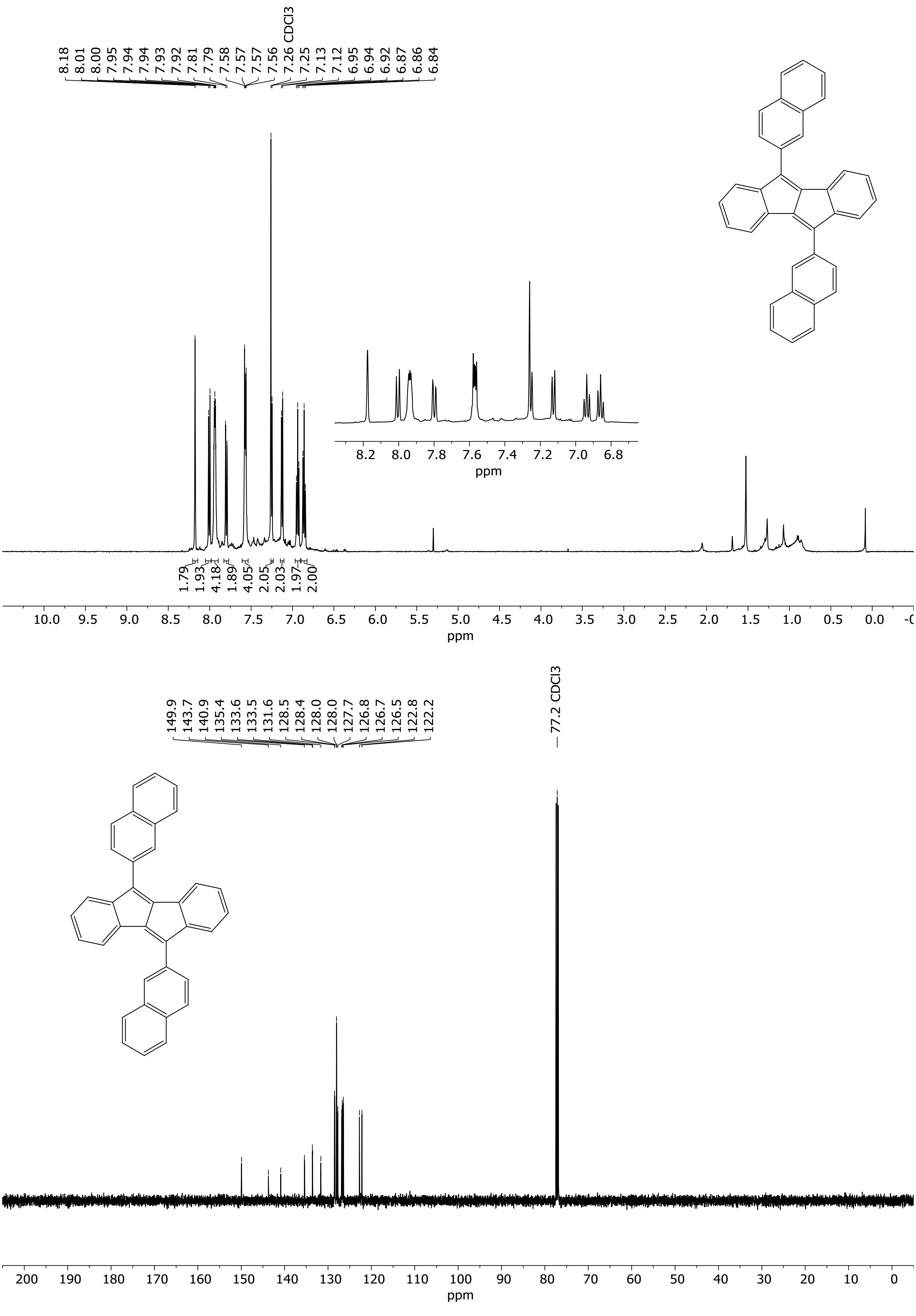

Figure S32. NMR spectra of compound **24** – ^{1}H, $CDCl_3$, 500 MHz (top); ^{13}C, $CDCl_3$, 126 MHz (bottom).

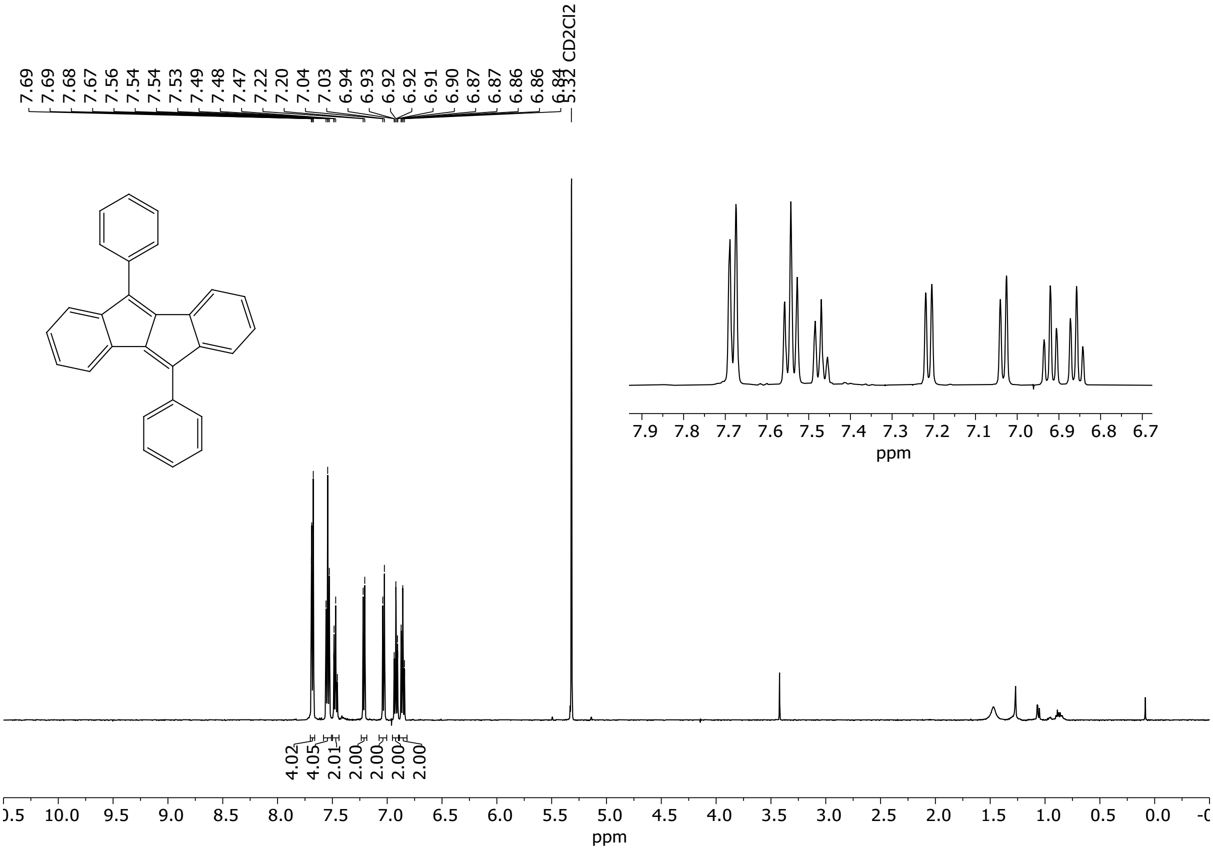

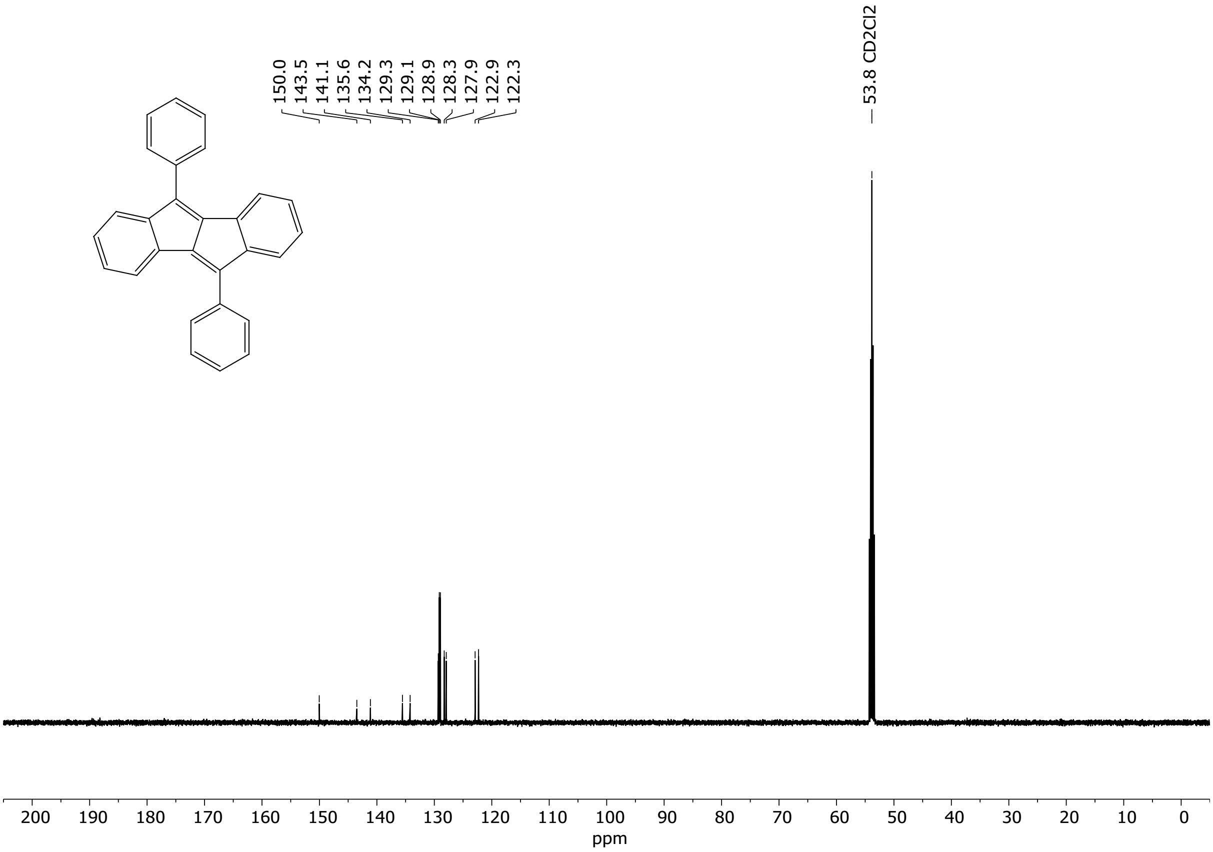

Figure S33. NMR spectra of compound **25** – ^{1}H, CD_2Cl_2, 500 MHz (top); ^{13}C, CD_2Cl_2, 126 MHz (bottom).

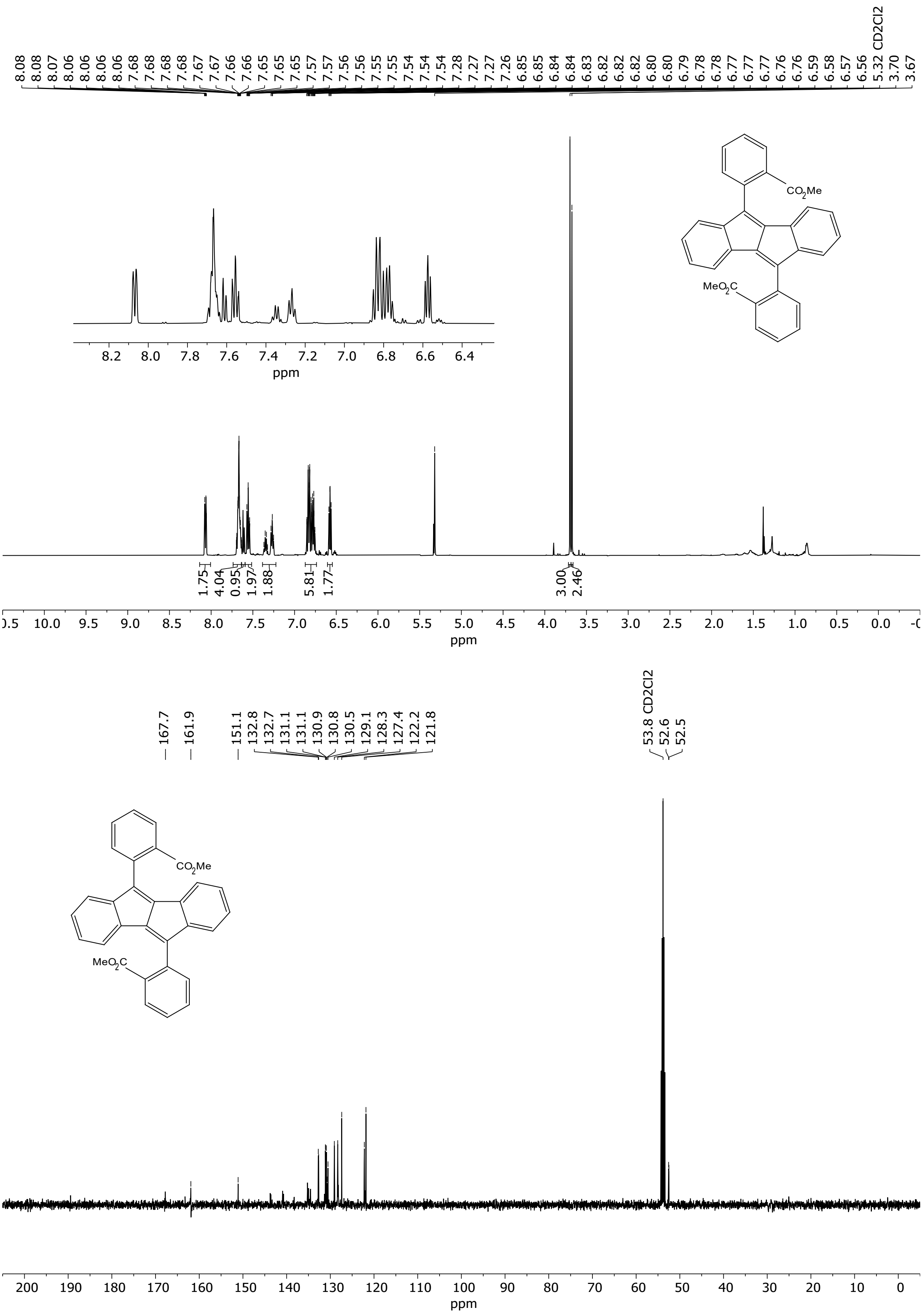

Figure S34. NMR spectra of compound **26** – ^{1}H, CD_2Cl_2, 500 MHz (top); ^{13}C, CD_2Cl_2, 126 MHz (bottom).

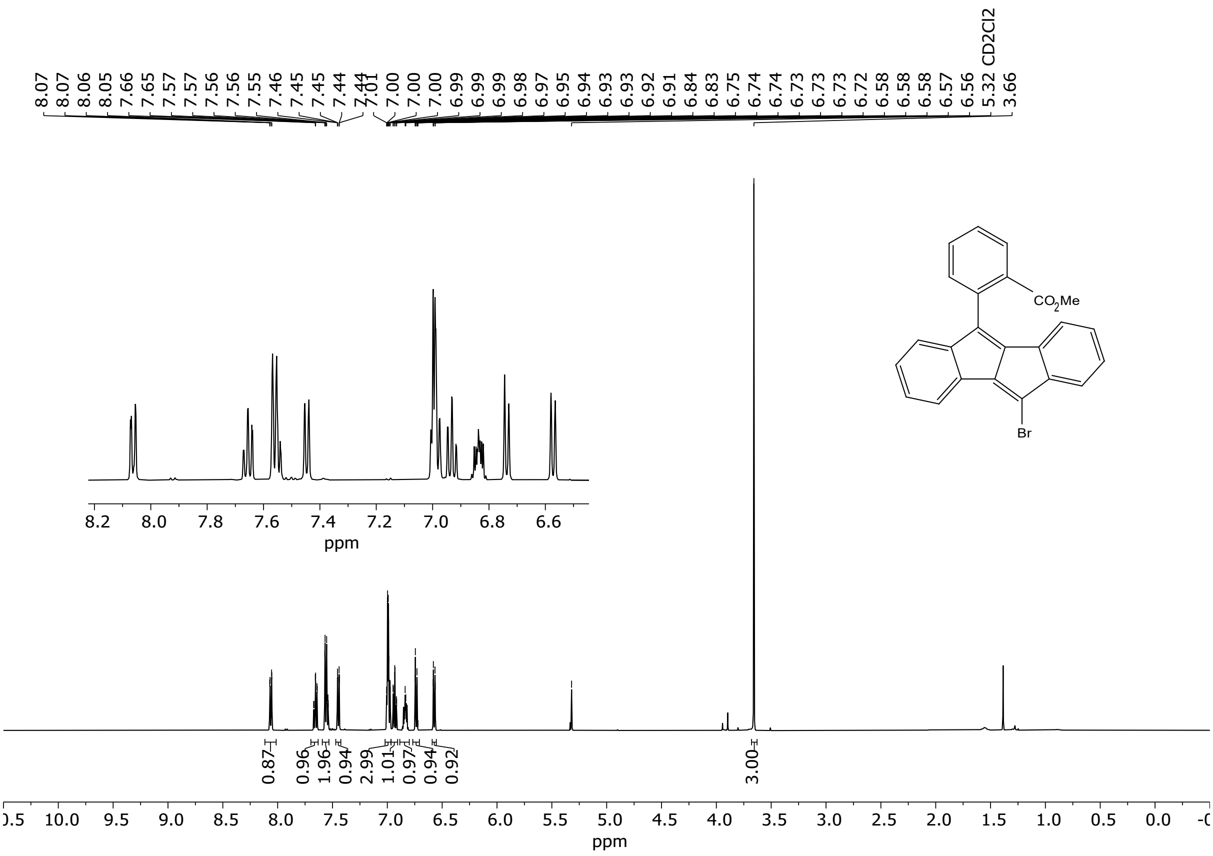

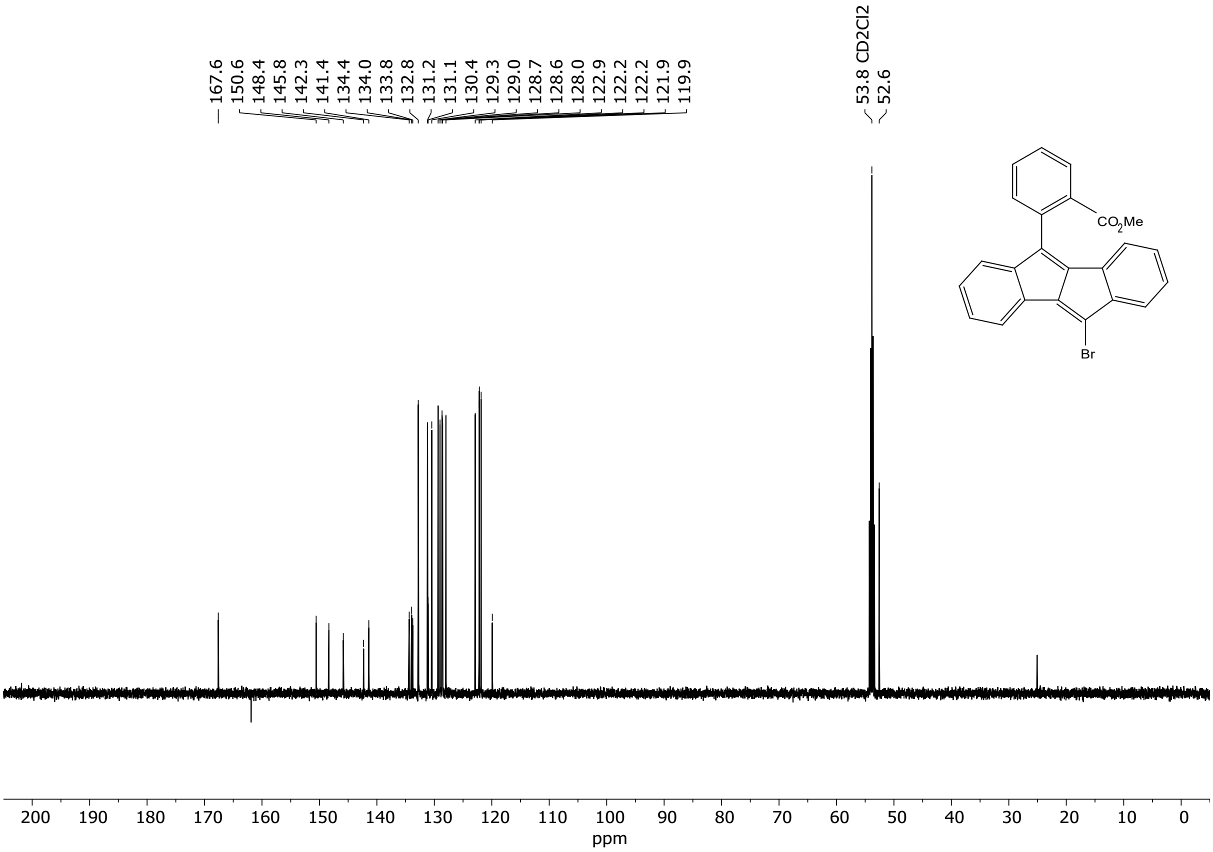

Figure S35. NMR spectra of compound **27** – ^{1}H, CD_2Cl_2, 500 MHz (top); ^{13}C, CD_2Cl_2, 126 MHz (bottom).

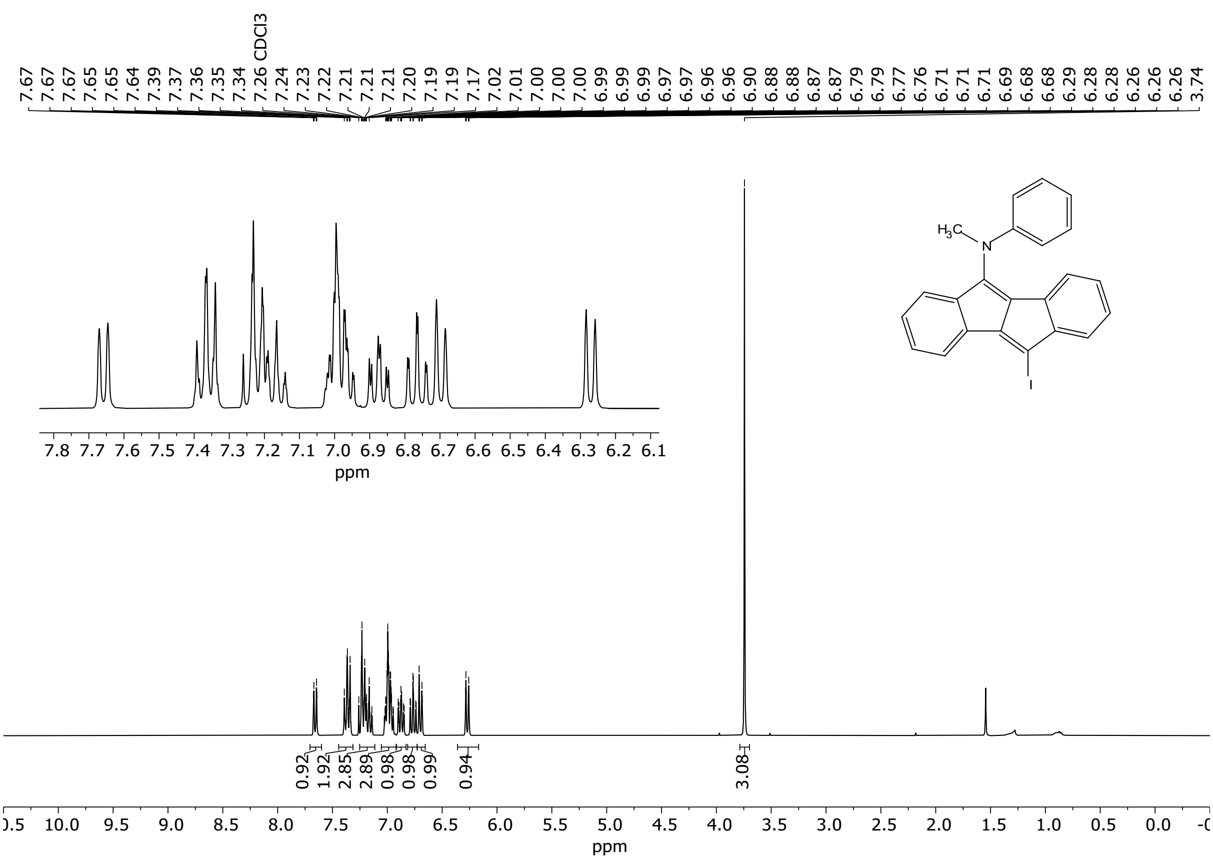

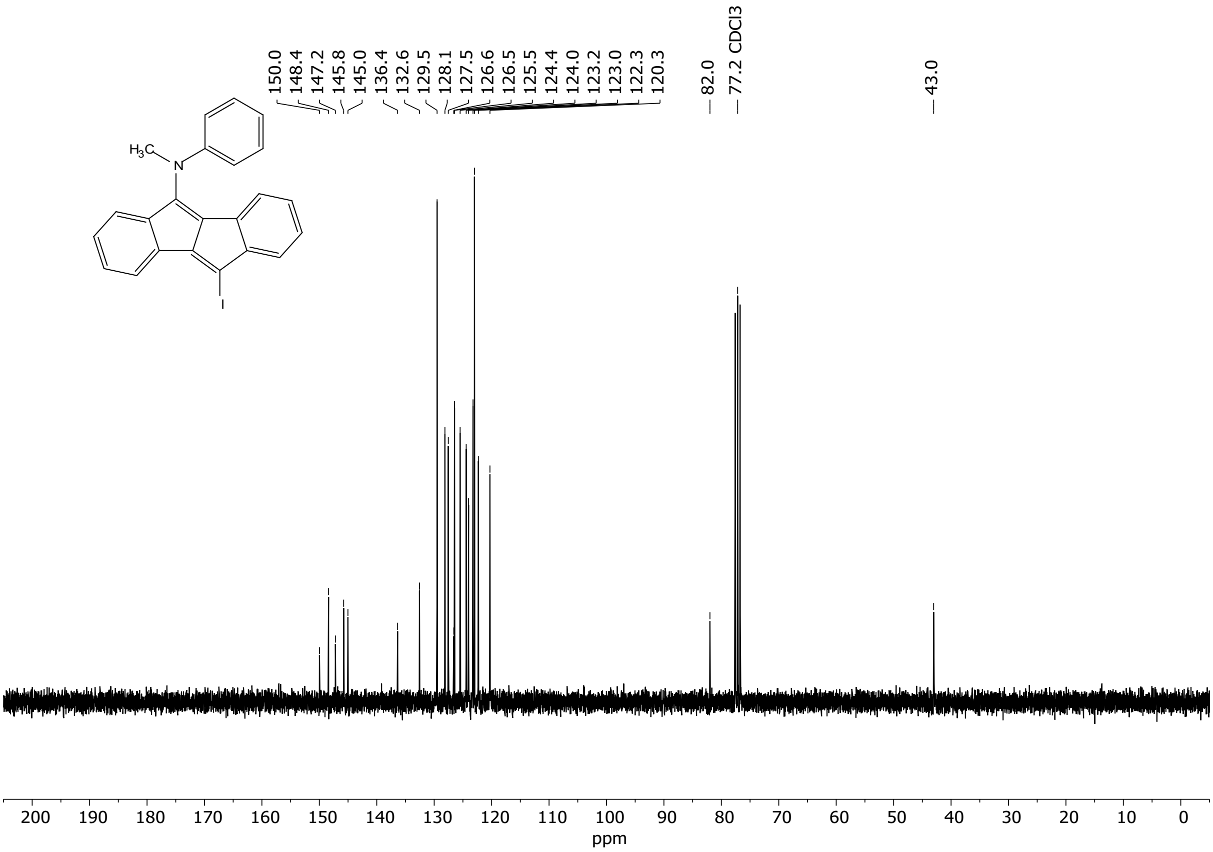

Figure S36. NMR spectra of compound **28** – ^{1}H, $CDCl_3$, 300 MHz (top); ^{13}C, $CDCl_3$, 75 MHz (bottom).

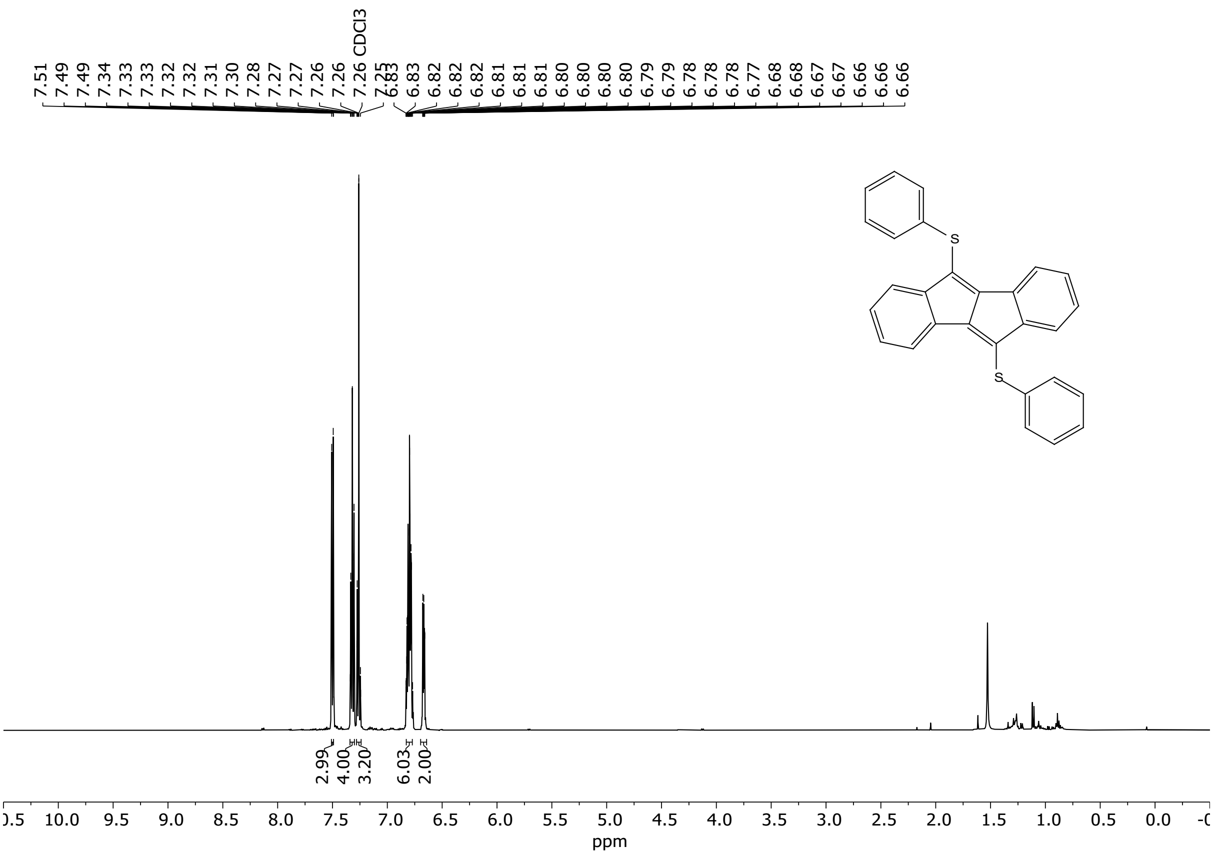

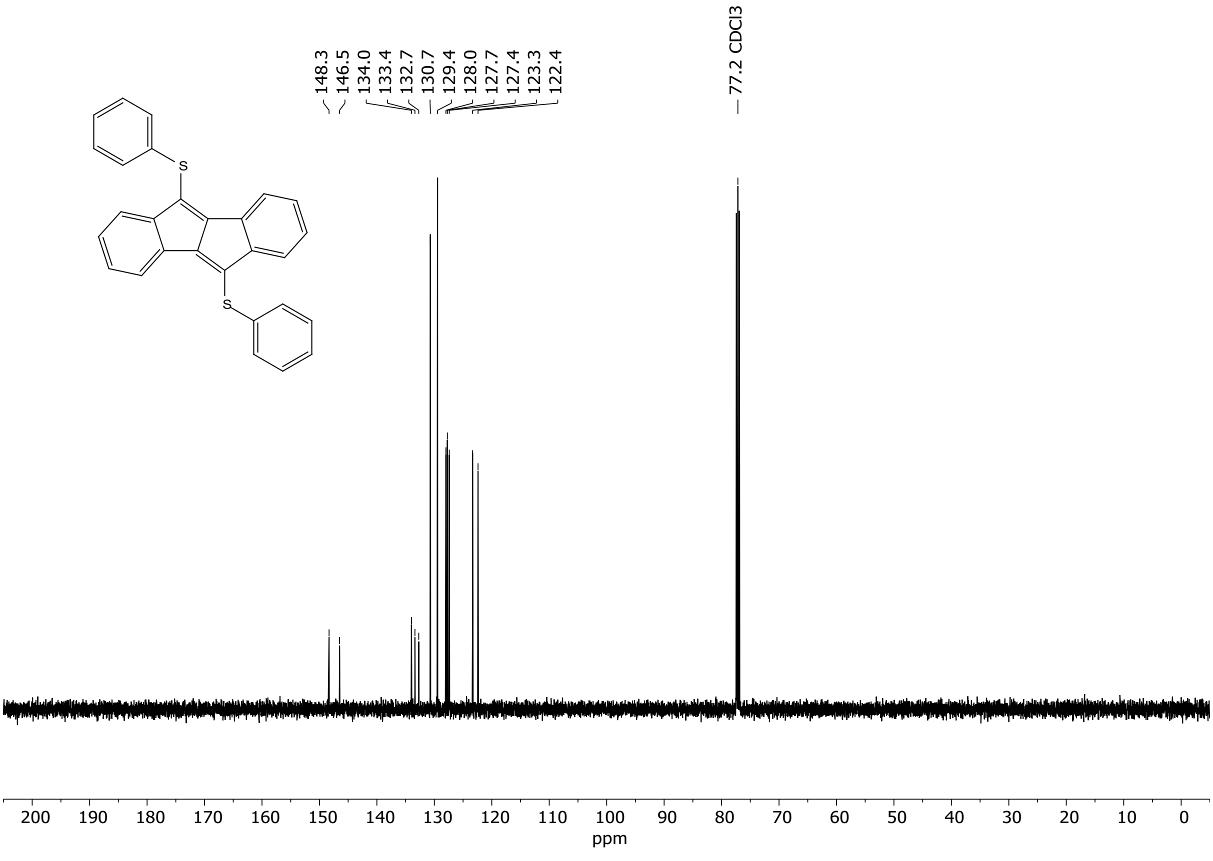

Figure S37. NMR spectra of compound **29** – ^{1}H, $CDCl_3$, 500 MHz (top); ^{13}C, CD_2Cl_2, 126 MHz (bottom).

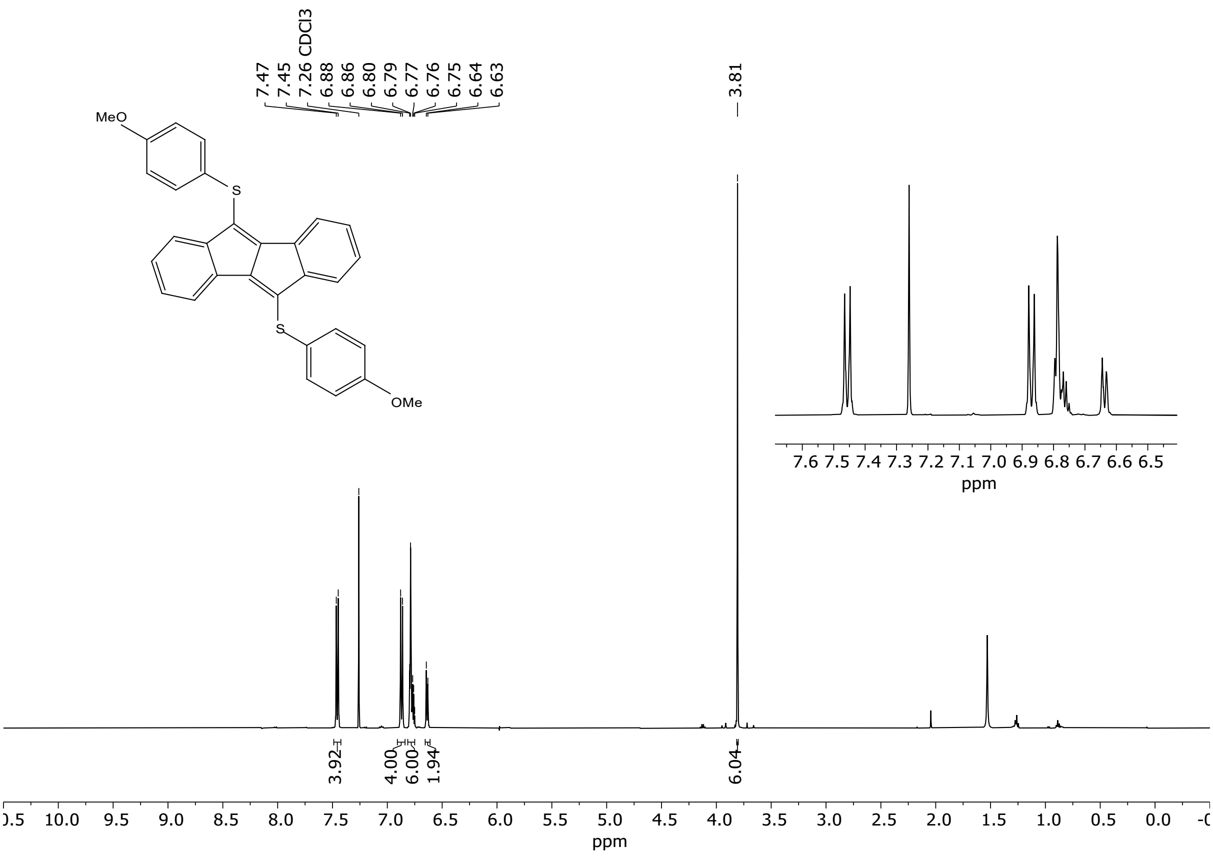

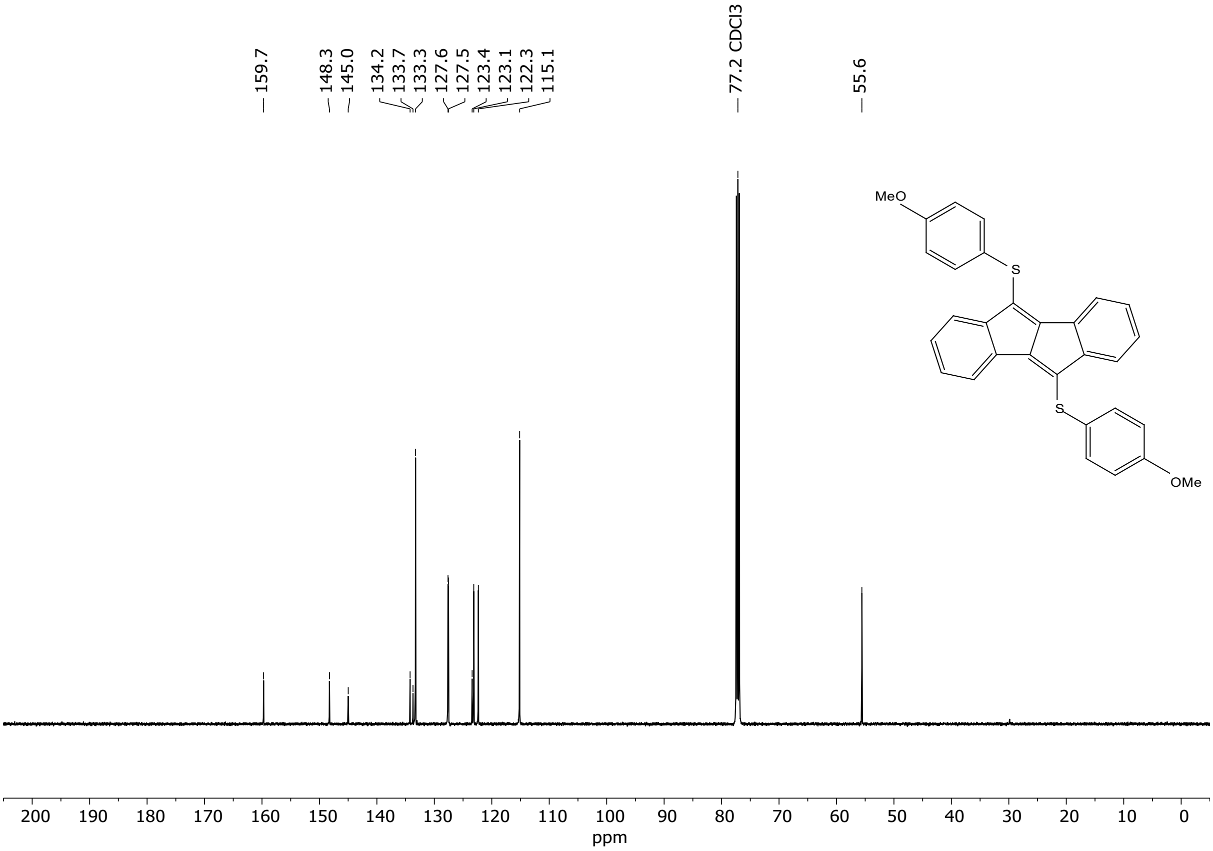

Figure S38. NMR spectra of compound **30** – ^{1}H, $CDCl_3$, 500 MHz (top); ^{13}C, $CDCl_3$, 126 MHz (bottom).

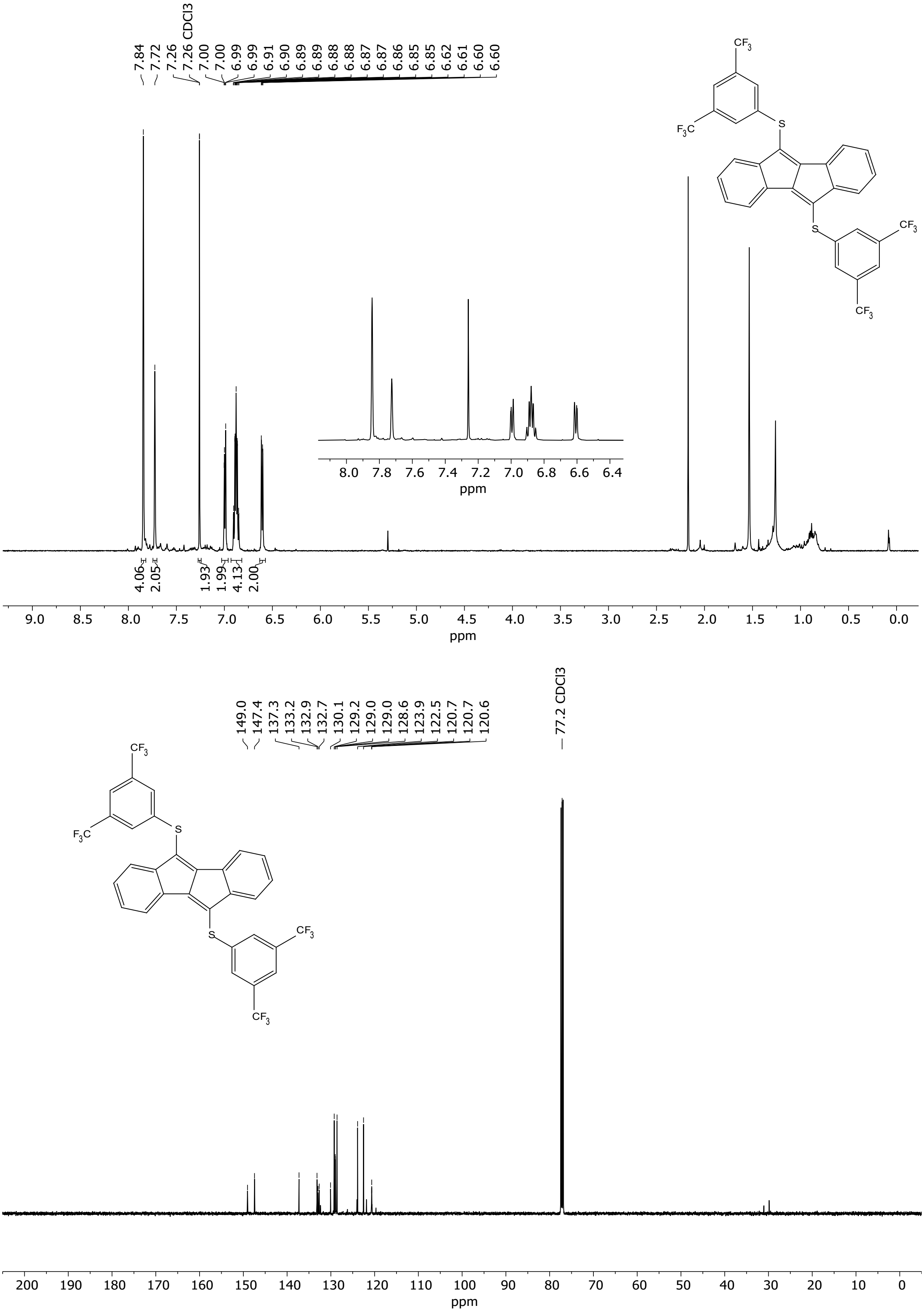

Figure S39. NMR spectra of compound **31** – ^{1}H, $CDCl_3$, 500 MHz (top); ^{13}C, $CDCl_3$, 151 MHz (bottom);

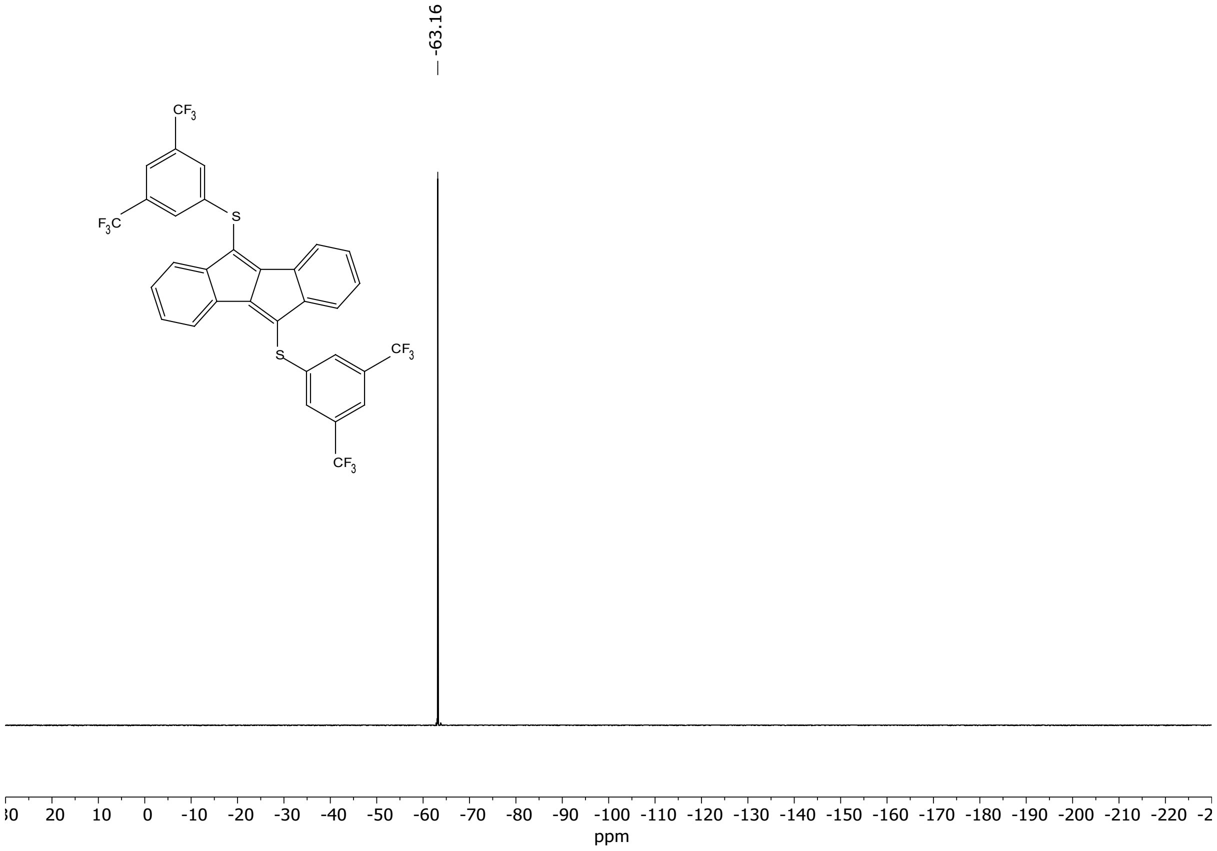

Figure S40. NMR spectrum of compound **31** – ^{19}F, $CDCl_3$, 282 MHz.

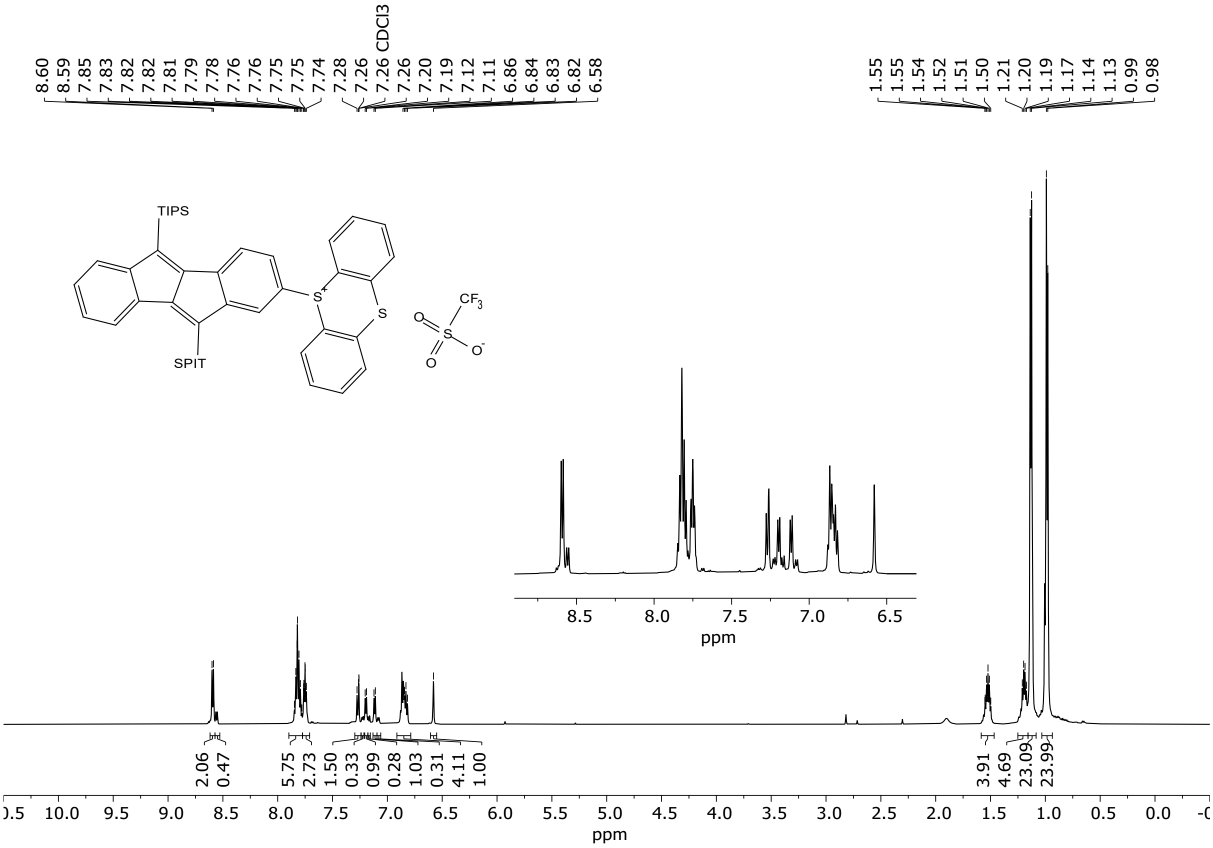

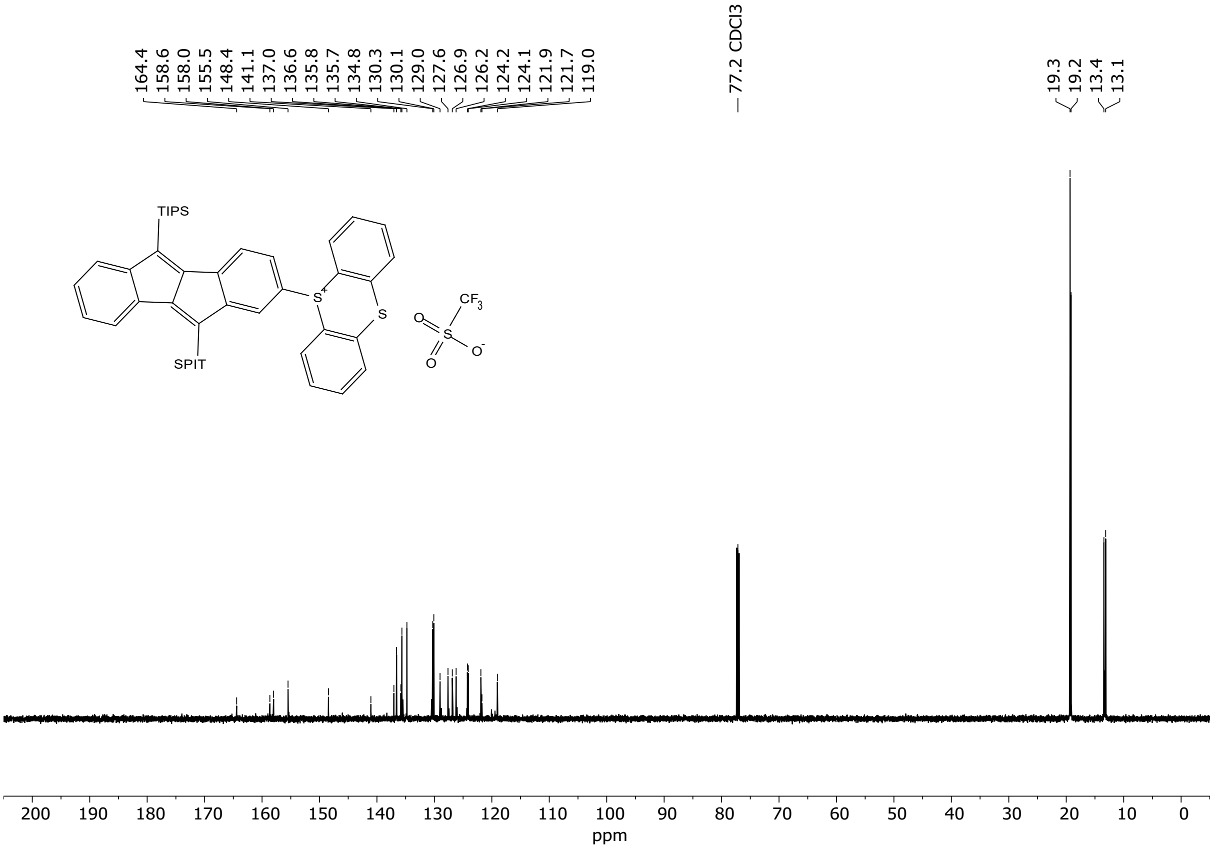

Figure S41. NMR spectra of compound **34** – ^{1}H, $CDCl_3$, 600 MHz (top); ^{13}C, $CDCl_3$, 151 MHz (bottom).

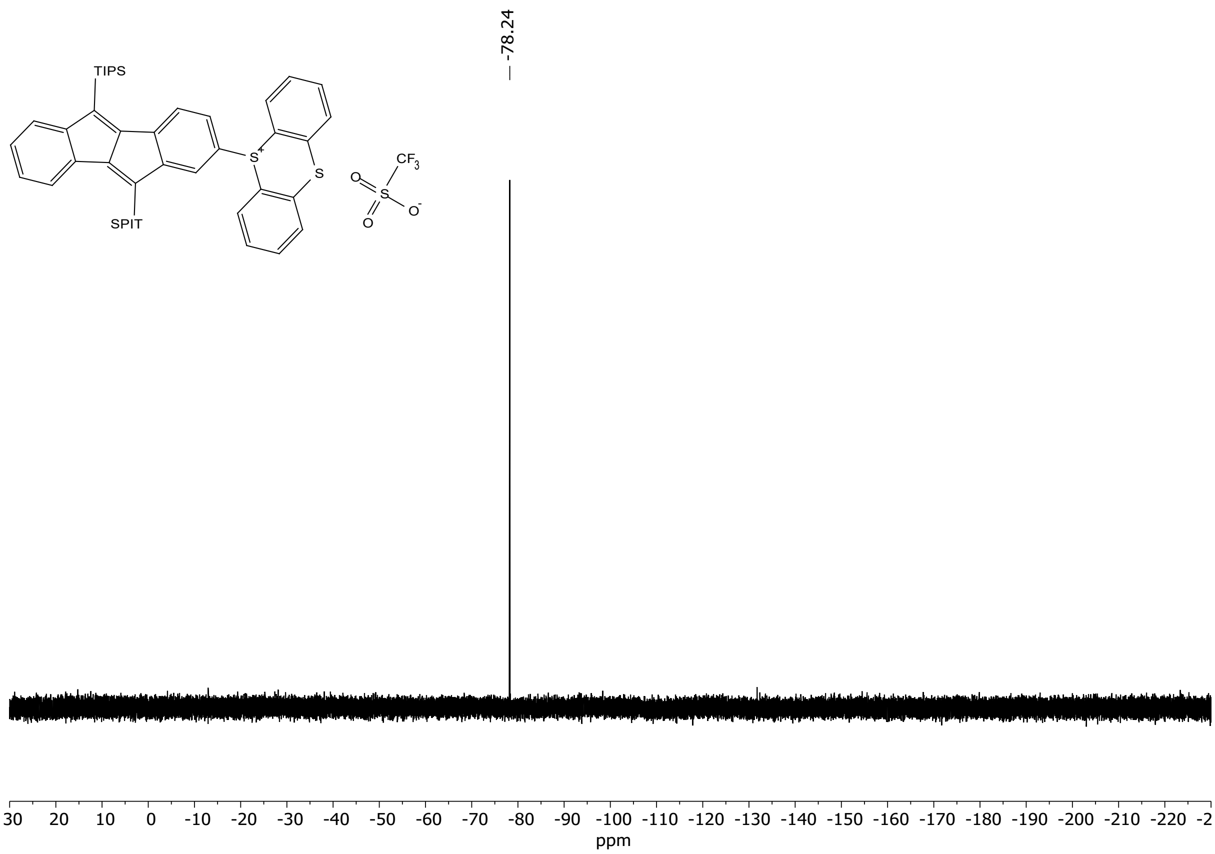

Figure S42. NMR spectra of compound **34** – ^{19}F, $CDCl_3$, 282 MHz.

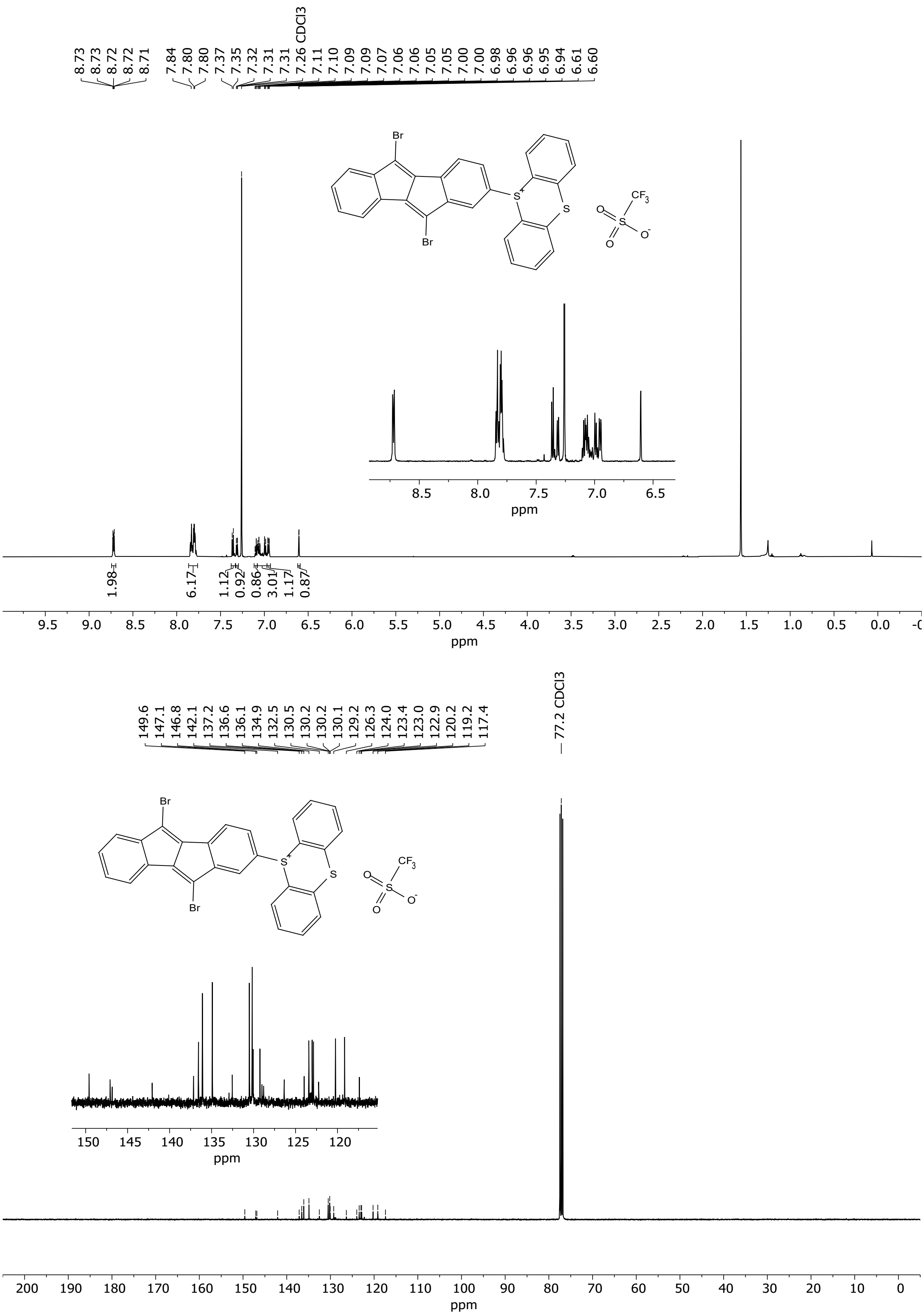

Figure S43. NMR spectra of compound **32** – ^{1}H, $CDCl_3$, 600 MHz (top); ^{13}C, $CDCl_3$, 151 MHz (bottom).

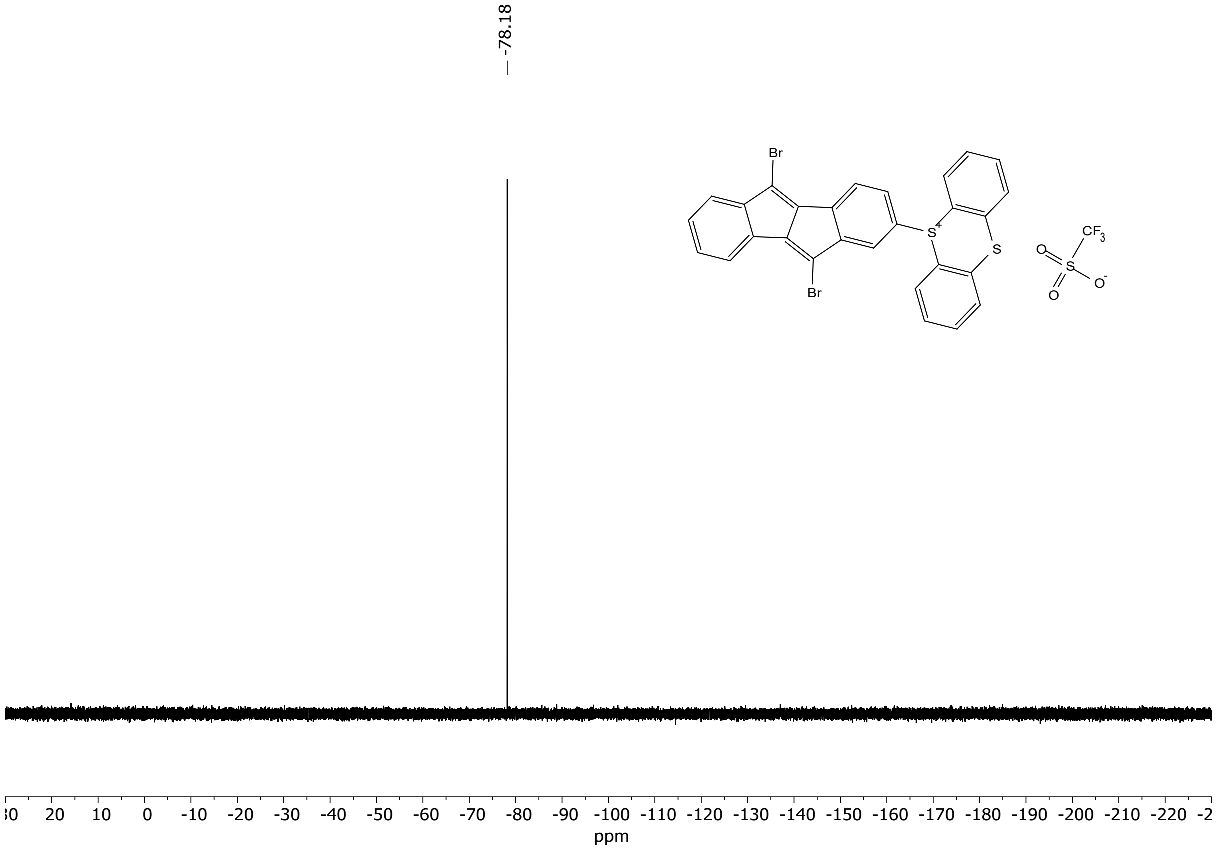

Figure S44. NMR specrum of compound **32** – ^{19}F, $CDCl_3$, 282 MHz.

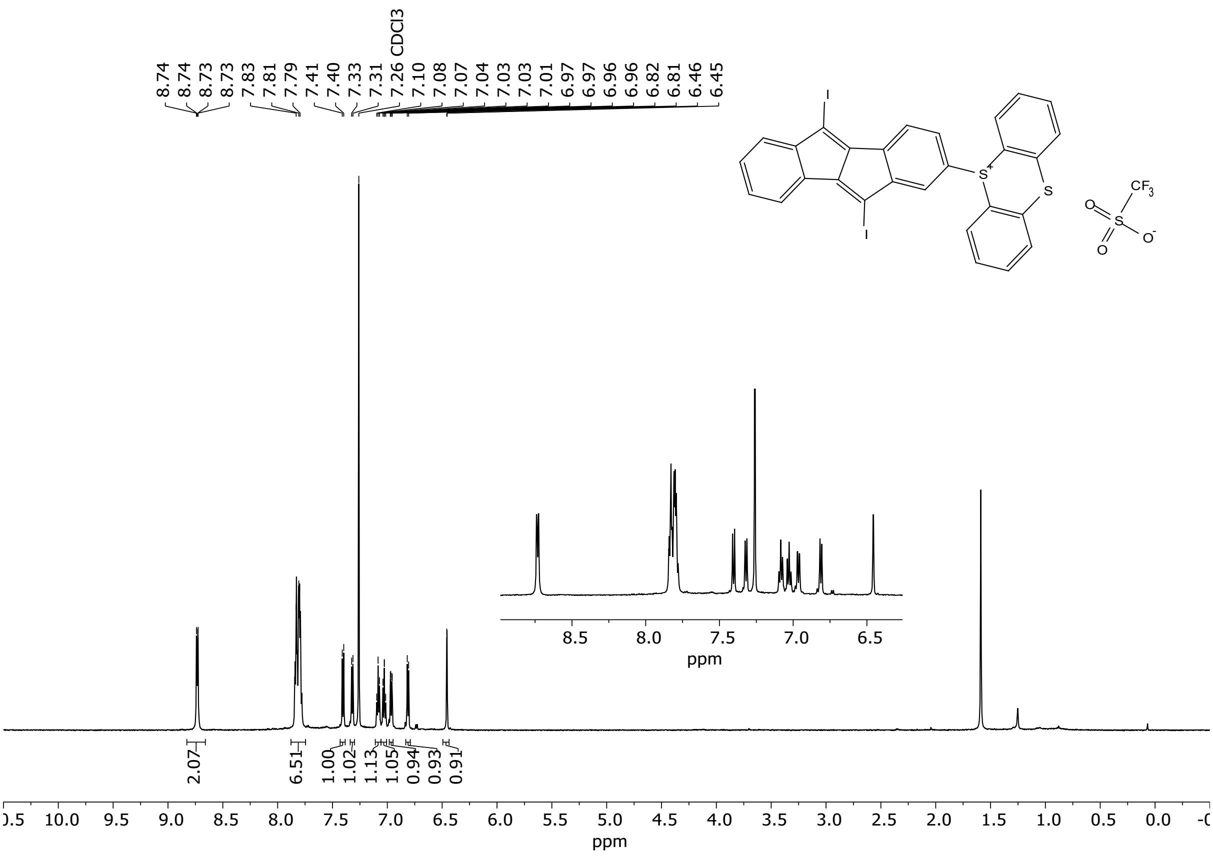

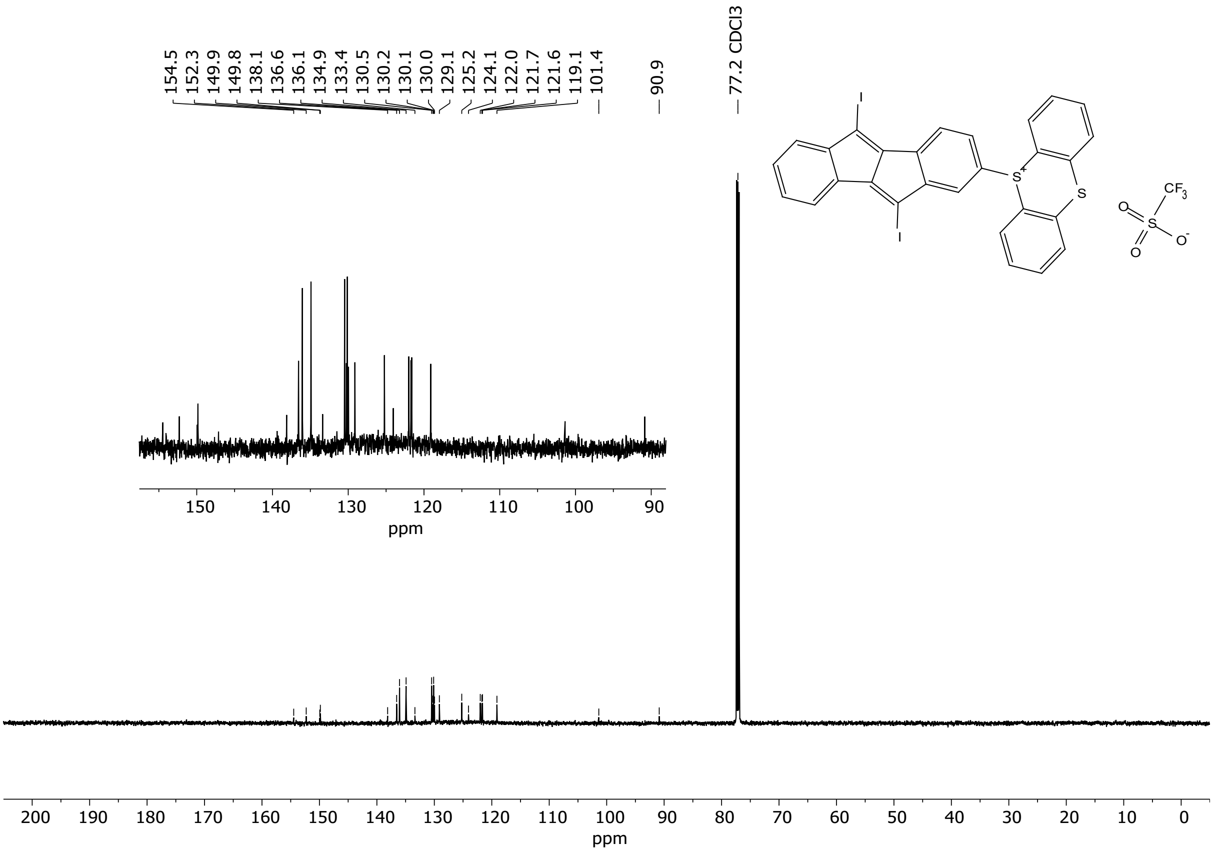

Figure S45. NMR spectra of compound **33** – ^{1}H, $CDCl_3$, 600 MHz (top); ^{13}C, $CDCl_3$, 151 MHz (bottom).

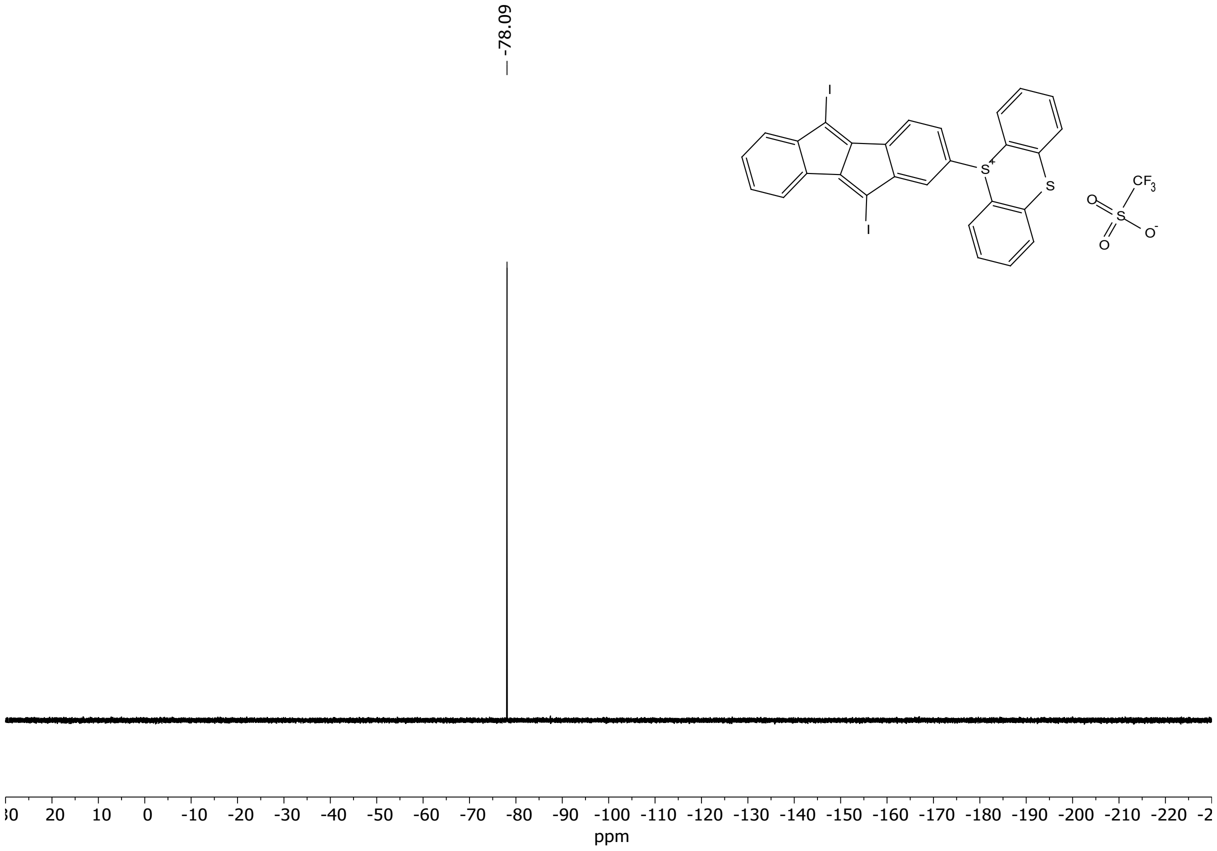

Figure S46. NMR spectrum of compound **33** – ^{19}F, $CDCl_3$, 282 MHz.

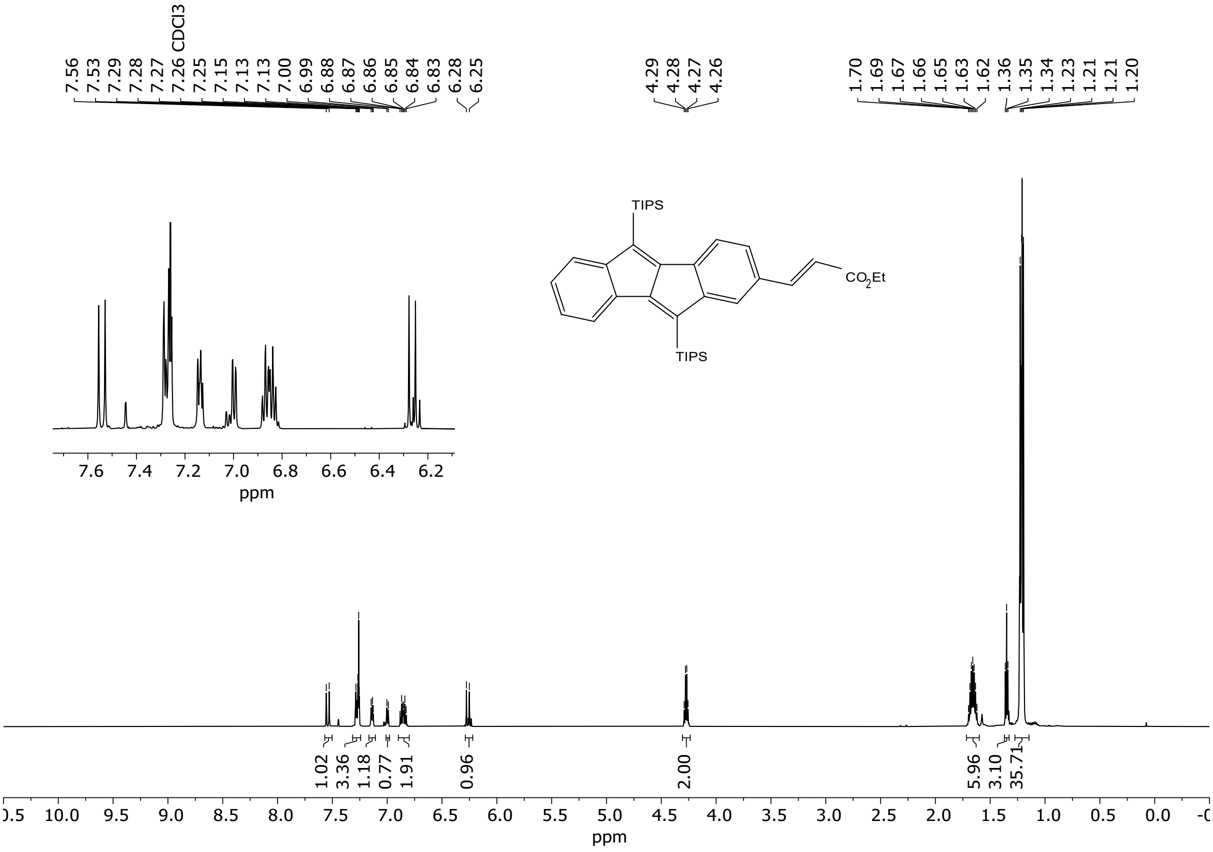

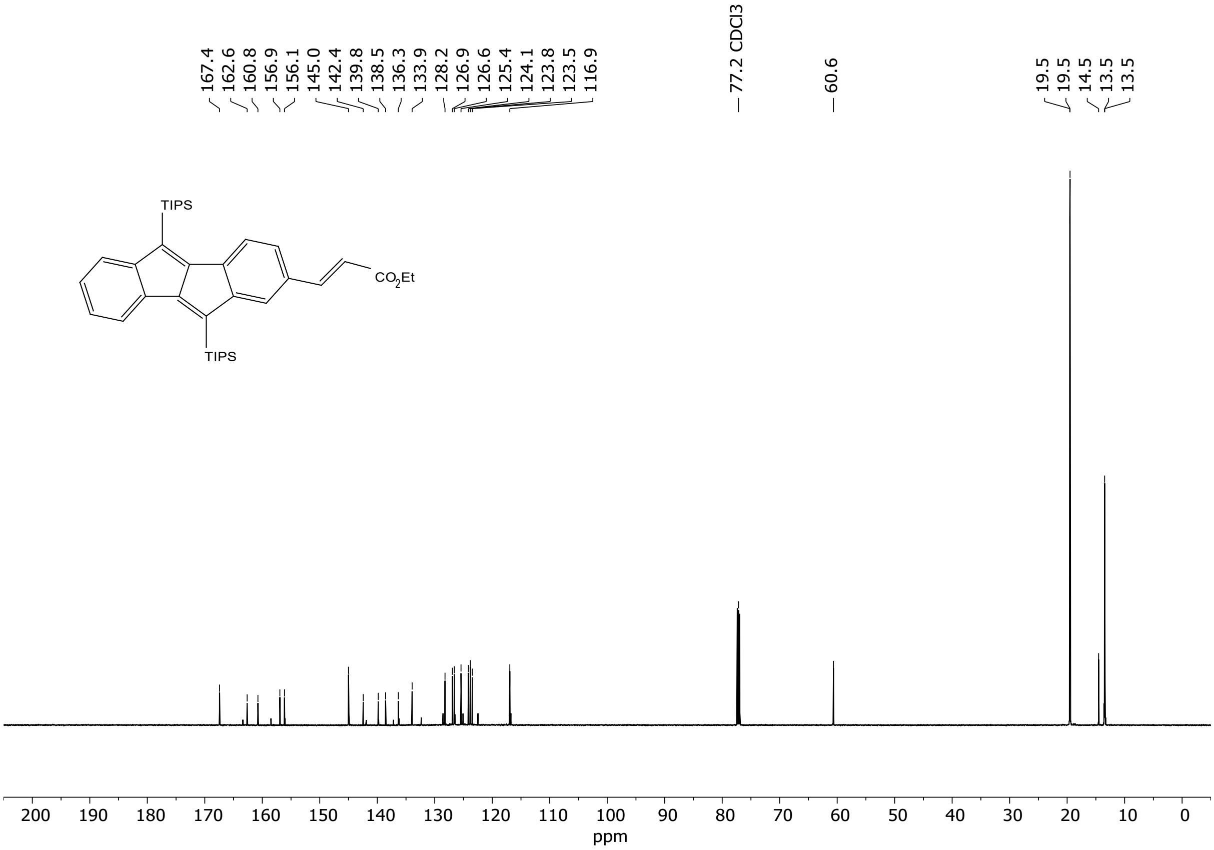

Figure S47. NMR spectra of compound **36** – ^{1}H, $CDCl_3$, 600 MHz (top); ^{13}C, $CDCl_3$, 151 MHz (bottom).

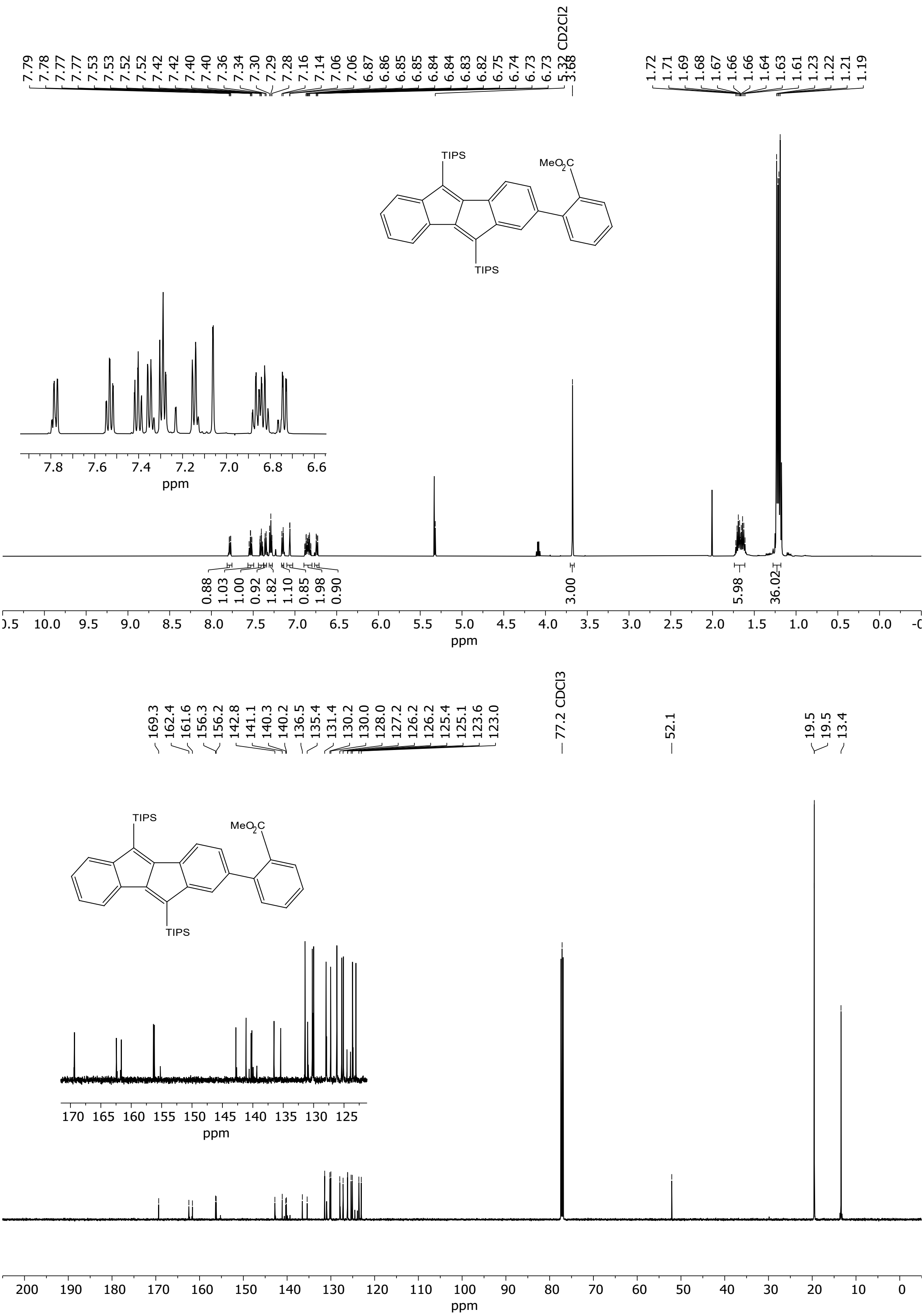

Figure S48. NMR spectra of compound **37** – ^{1}H, CD_2Cl_2, 500 MHz (top); ^{13}C, $CDCl_3$, 126 MHz (bottom).

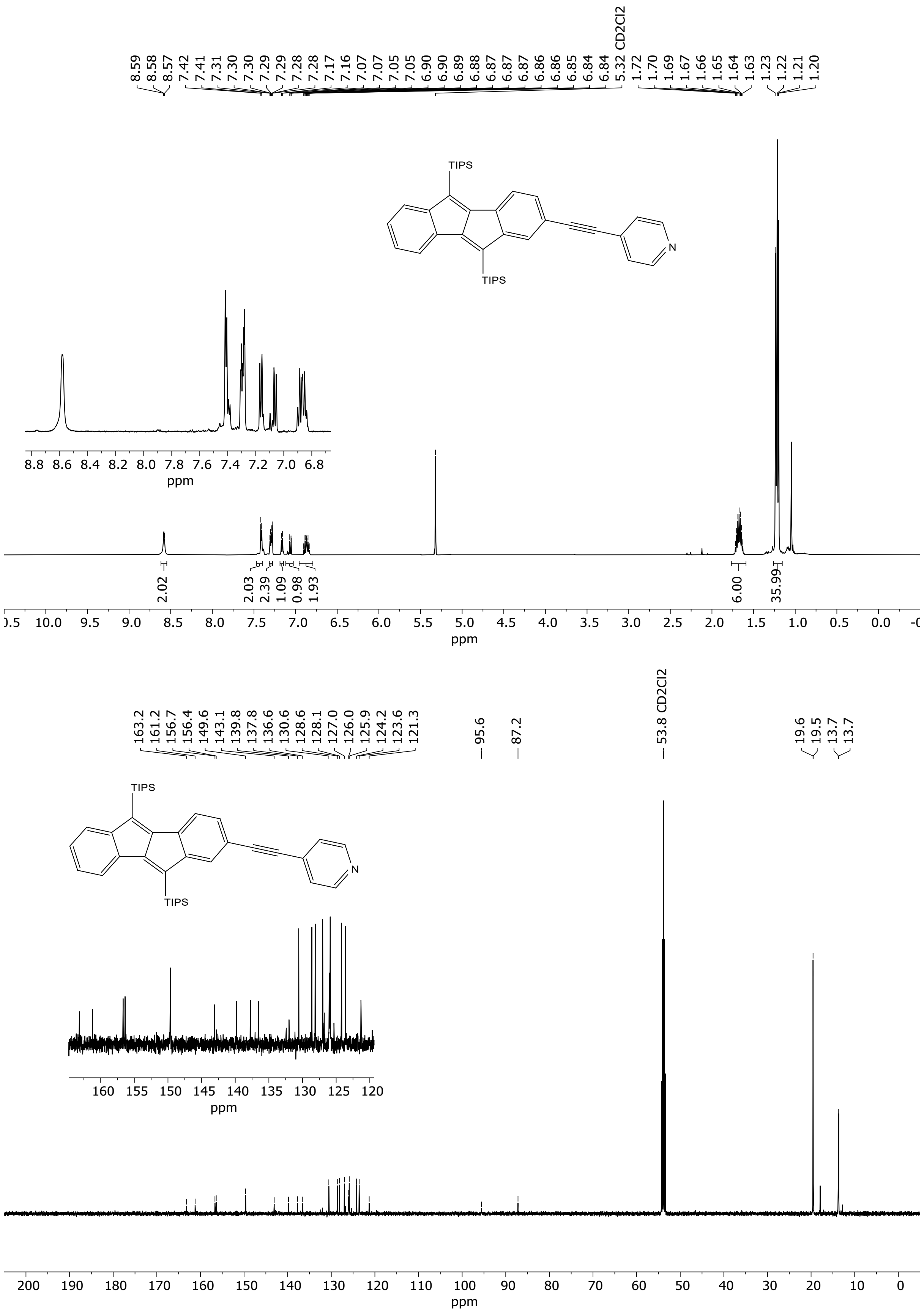

Figure S49. NMR spectra of compound **38** – ^{1}H, CD_2Cl_2, 500 MHz (top); ^{13}C, CD_2Cl_2, 126 MHz (bottom).

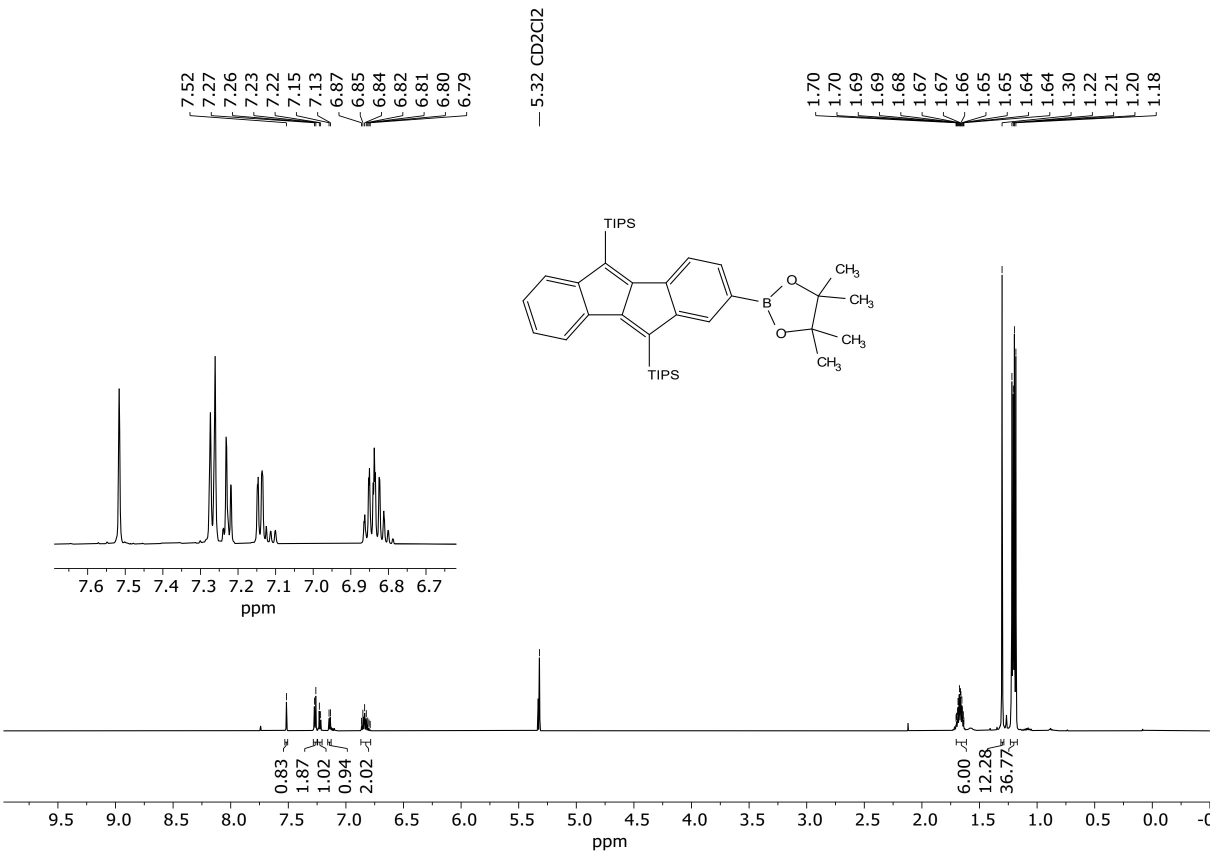

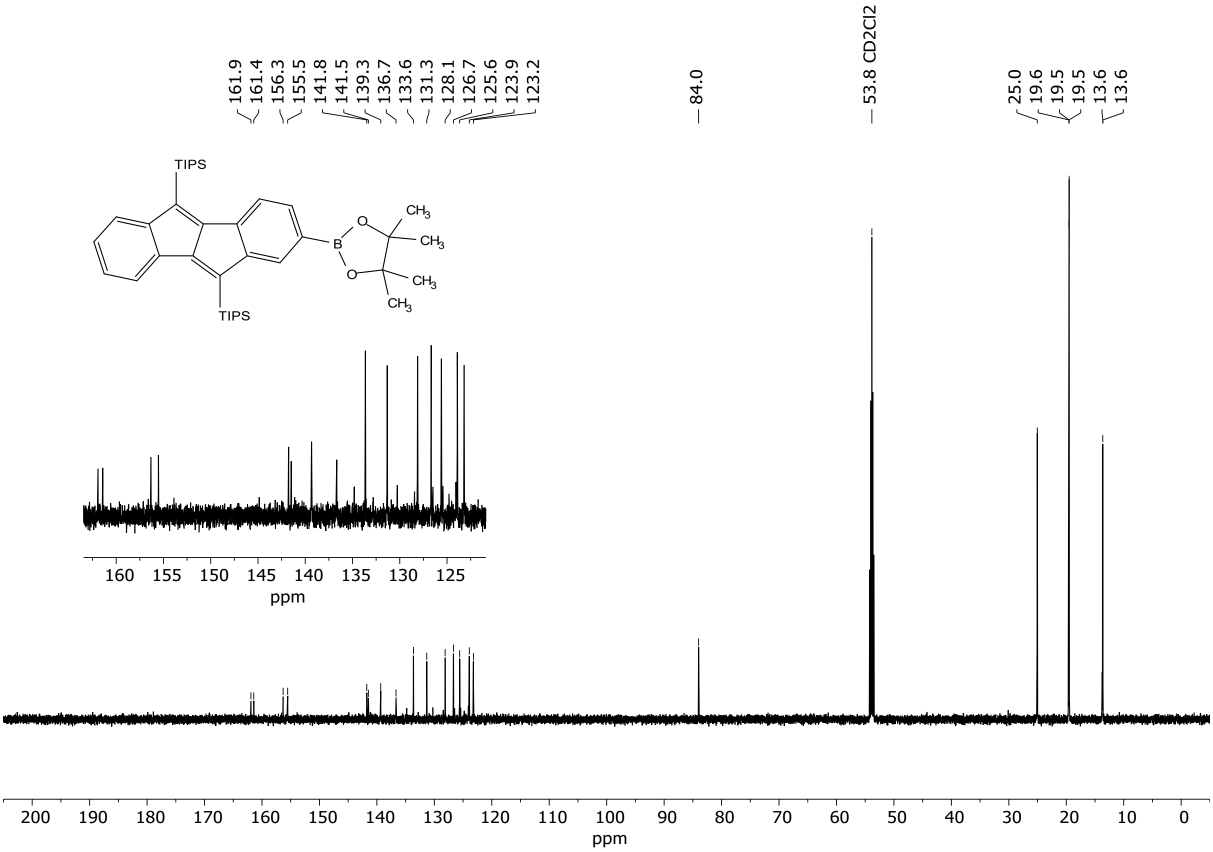

Figure S50. NMR spectra of compound **39** – ^{1}H, CD_2Cl_2, 600 MHz (top); ^{13}C, CD_2Cl_2, 151 MHz (bottom).

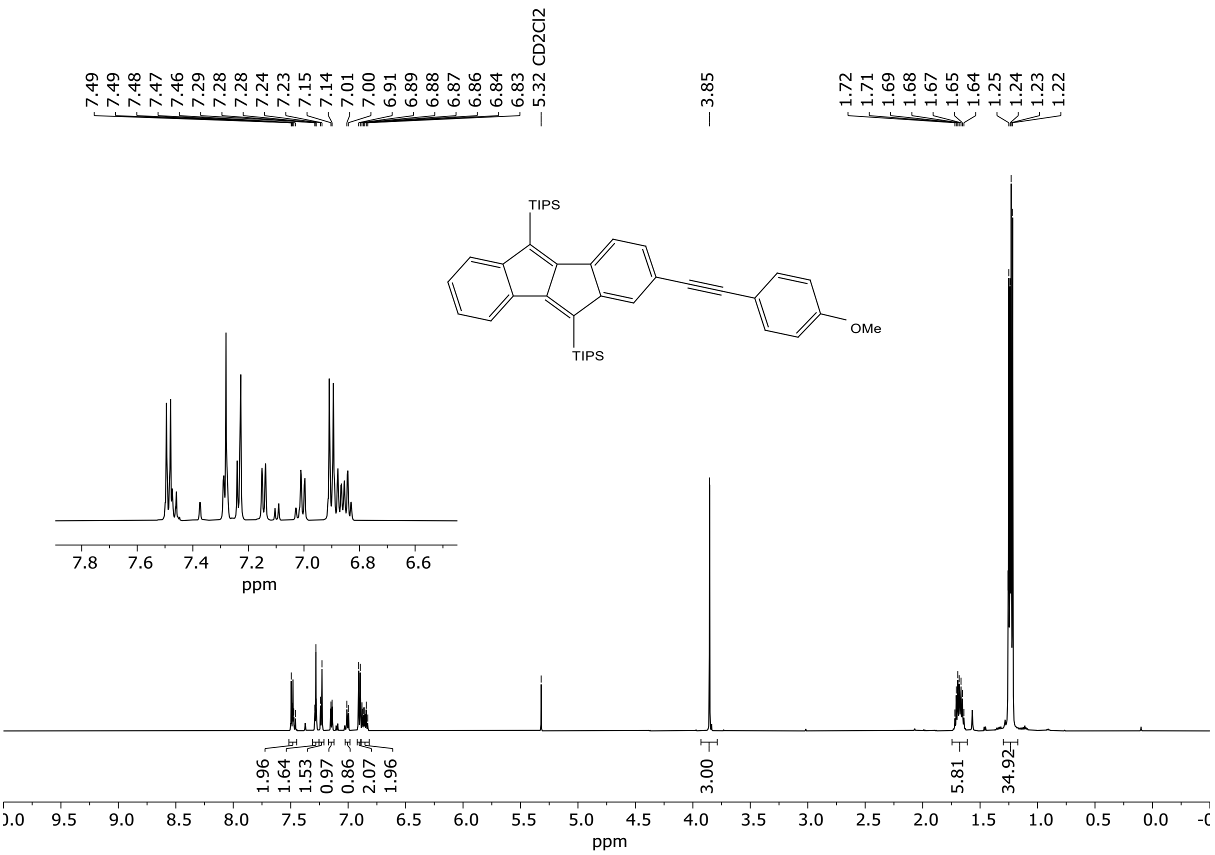

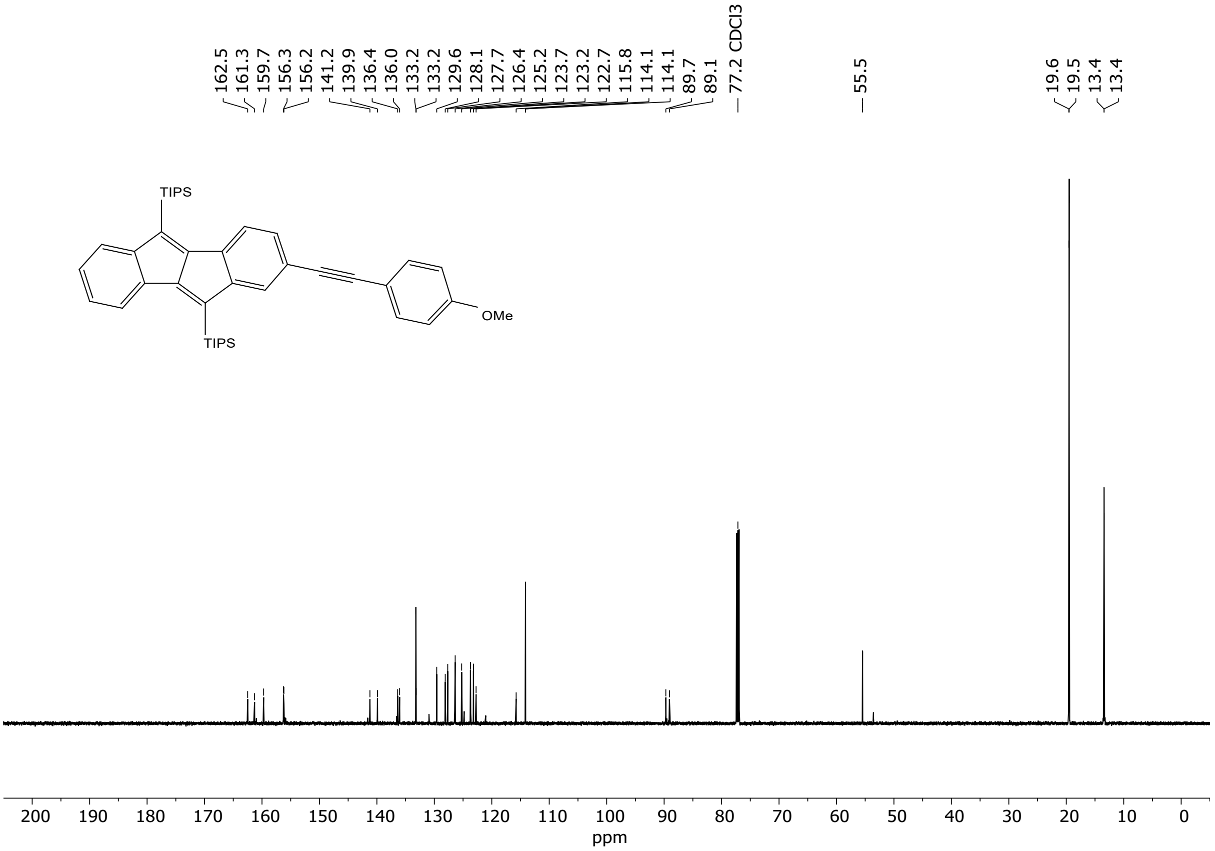

Figure S51. NMR spectra of compound **40** – ^{1}H, CD_2Cl_2, 600 MHz (top); ^{13}C, CD_2Cl_2, 151 MHz (bottom).

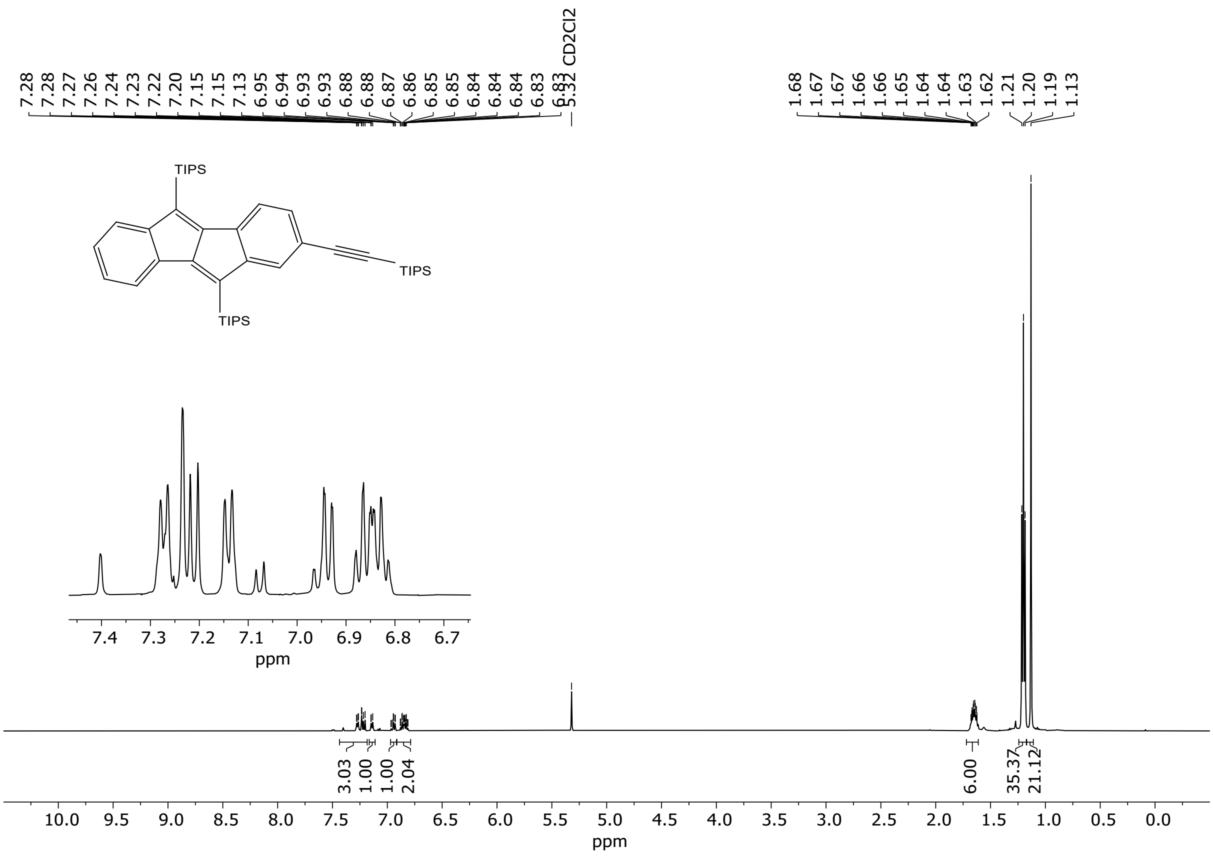

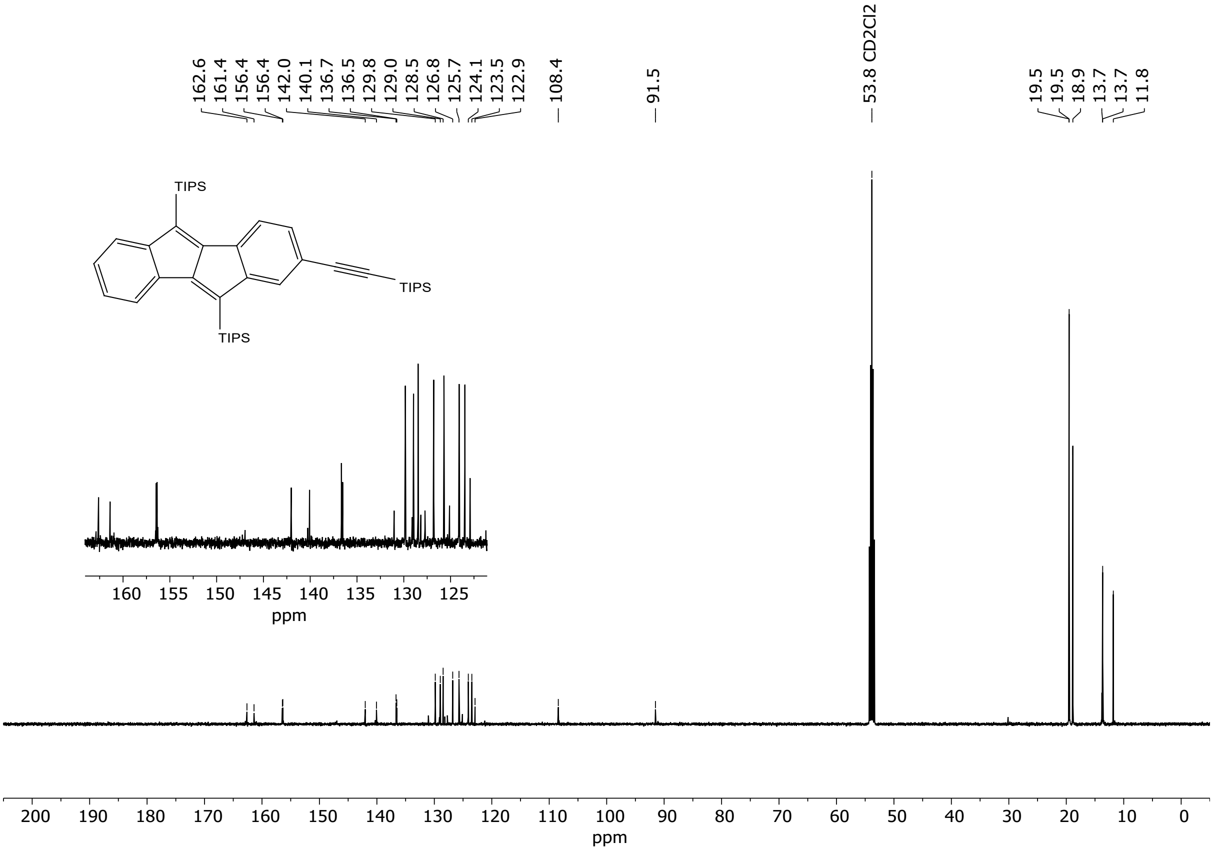

Figure S52. NMR spectra of compound **41** – ^{1}H, CD_2Cl_2, 500 MHz (top); ^{13}C, CD_2Cl_2, 126 MHz (bottom).

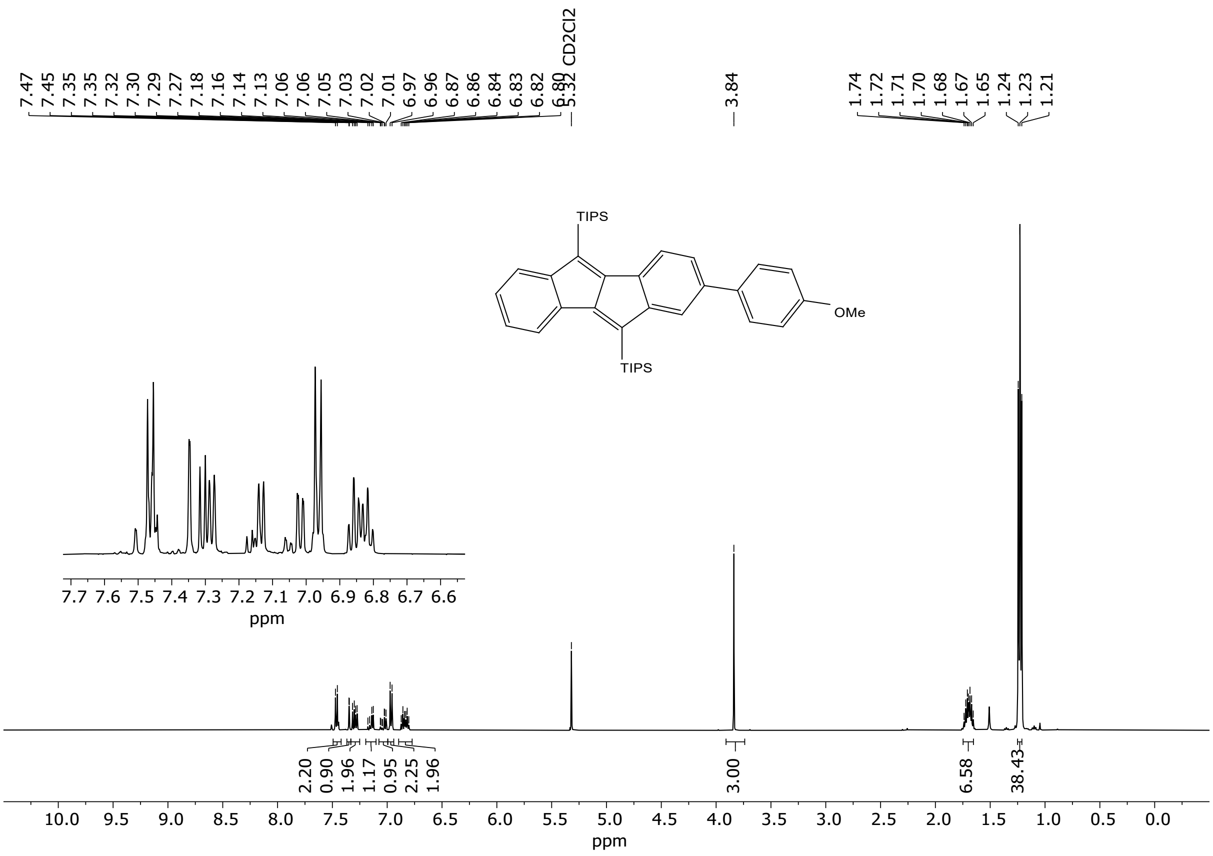

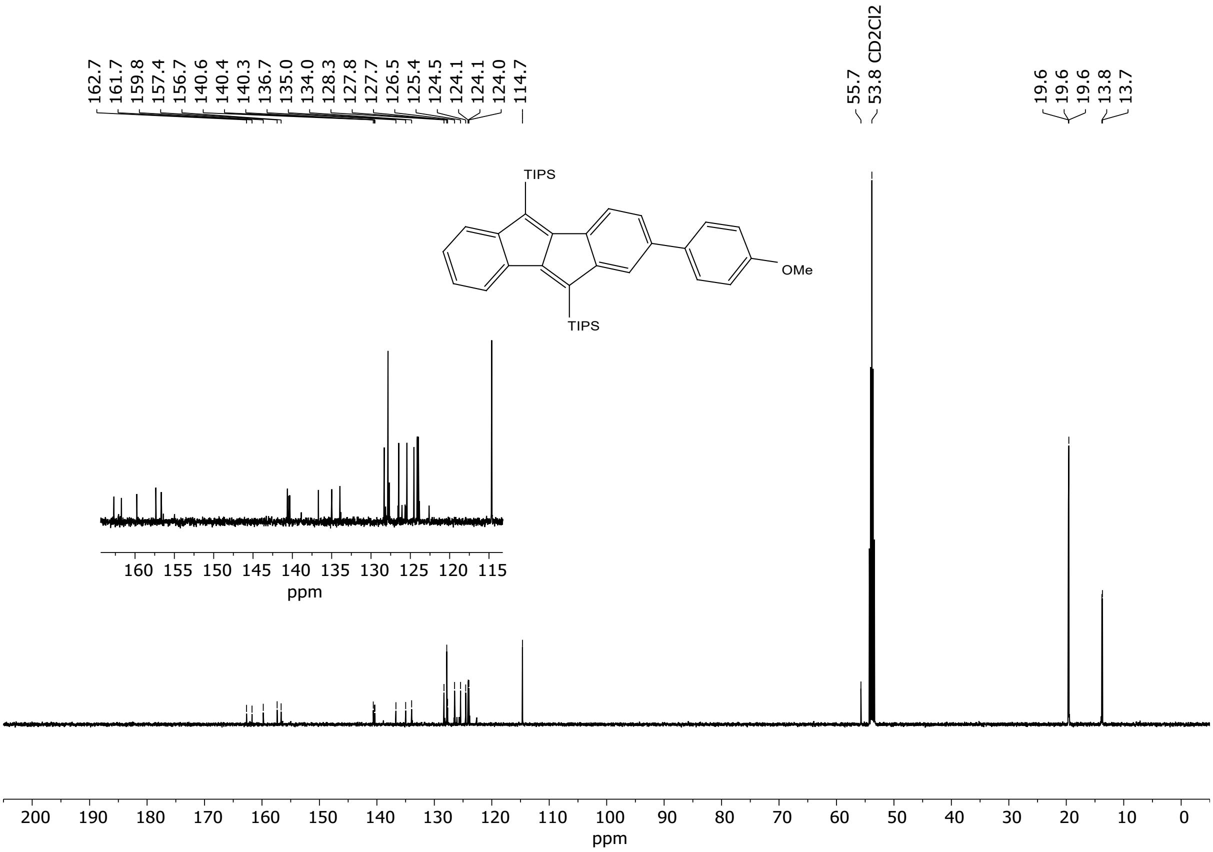

Figure S53. NMR spectra of compound **42** – ^{1}H, CD_2Cl_2, 500 MHz (top); ^{13}C, CD_2Cl_2, 126 MHz (bottom).

S6 References

1 J. Sprachmann, T. Wachsmuth, M. Bhosale, D. Burmeister, G.J. Smales, M. Schmidt, Z. Kochovski, N. Grabicki, R. Wessling, E.J.W. List-Kratochvil, B. Esser, O. Dumele, *J. Am. Chem. Soc.*, 2013, **145**, 2840-2851.

2 T. Kawase, A. Konishi, Y. Hirao, K. Matsumoto, H. Kurata, T. Kubo, *Chem. Eur. J.*, 2009, **15**, 2653-2661.

3 A.S.K. Hashmi, M. Wieteck, I. Braun, P. Nösel, L. Jongbloed, M. Rudolph, F. Rominger, *Adv. Synth. Catal.,* **2012**, *354*, 555-562.

4 F. Xu, L. Peng, A. Orita, J. Otera, *Org. Lett.*, 2012, **15**, 3970-3973.

5 S. Chaffins, M. Brettreich, F. Wudl, *Synthesis*, 2002, **9**, 1191-1194.

6 W. Srimontree, A. Chatupheeraphat, H.-H. Liao, M. Rueping, *Org. Lett.*, 2017, **19**, 3091–3094.

7 M. Saito, M. Nakamura, T. Tajima, M. Yoshioka, *Angew. Chem. Int. Ed.*, 2007, **46**, 1504-1507.

8 J. H. Smith, D. Cavlovic, L.T. Lackovic, M.M. Lopez, E. Meirzadeh, M.L. Steigerwald, X. Roy, C.P. Nuckolls, S.R. Docherty, *J. Am. Chem. Soc.*, 2025, **147**, 111-117.

9 D. Ahmadli, S. Müller, Y. Xie, T. Smejkal, S. Jaeckh, A.V. Iosub, S.R. Williams, T. Ritter, *J. Am. Chem. Soc.*, 2025, **147**, 4268-4283.

10 F. Berger, M.B. Plutschack, J. Riegger, W. Yu, S. Speicher, M. Ho, N. Frank, T. Ritter, *Nature*, 2019, **567**, 223-228.

11 O.V. Dolomanov, L.J. Bourhis, R.J. Gildea, J.A.K. Howard, H. Puschman, *J. Appl. Cryst.*, 2009, **42**, 339-341.

12 G.M. Sheldrick, *Acta Cryst.*, 2015, **A71**, 3-8.

13 G.M. Sheldrick, *Acta Cryst.*, 2015, **C71**, 3-8.

14 C.R. Groom, I.J. Bruno, M.P. Lightfoot, S.C. Ward, *Acta Cryst.*, 2016, **B72**, 171-179.
